# The structures and reactivity of NHC-supported copper(i) triphenylgermyls†

**DOI:** 10.1039/d3sc05862j

**Published:** 2023-12-01

**Authors:** Rex S. C. Charman, Nick J. Evans, Laura E. English, Samuel E. Neale, Petra Vasko, Mary F. Mahon, David J. Liptrot

**Affiliations:** a Department of Chemistry, University of Bath Bath BA2 7AY UK d.j.liptrot@bath.ac.uk; b Centre for Sustainable and Circular Technology Bath BA2 7AY UK; c Department of Chemistry, University of Helsinki A.I. Virtasen aukio 1 P.O. Box 55 FI-00014 Finland; d X-Ray Crystallography Suite, University of Bath Bath BA2 7AY UK m.f.mahon@bath.ac.uk

## Abstract

Deprotonation of triphenyl germane with NHC-supported copper alkoxides afforded four novel (NHC)CuGePh_3_ complexes. Of these, (IPr)CuGePh_3_ (IPr = :C{N(2,6-iPr_2_C_6_H_3_)CH}_2_) was selected for further investigation. Analysis by EDA-NOCV indicates it to be a germyl nucleophile and its σ-bond metathesis reaction with a range of p-block halides confirmed it to be a convenient source of [Ph_3_Ge]^−^. The Cu–Ge bond of (IPr)CuGePh_3_ underwent π-bond insertions with *t*BuNCS, CS_2_, and PhNCO to furnish a series of germyl substituted carboxylate derivatives, (IPr)CuXC(Y)GePh_3_ (X = S, NPh; Y = S, N*t*Bu, O), which were structurally characterised. (IPr)CuGePh_3_ inserted phenyl acetylene, providing both the Markovnikov and anti-Markovnikov products. The (NHC)CuGePh_3_ compounds were validated as catalytic intermediates; addition of 10 mol% of NHC-copper(i) alkoxide to a mixture of triphenyl germane and a tin(iv) alkoxide resulted in a tin/germanium cross coupling with concomitant formation of alcohol. Moreover, a catalytic hydrogermylation of Michael acceptors was developed with Ph_3_GeH adding to 7 activated alkenes in good conversions and yields in the presence of 10 mol% of NHC-copper(i) alkoxide. In all cases, this reaction provided the β-germylated substrate implicating nucleophilicity at germanium.

## Abstract 2

Four N-heterocyclic carbene (NHC) supported copper(i) germyls have been synthesised containing both normal- and ring-expanded NHCs. Their π-insertion and σ-bond metathesis chemistry has been explored in stoichiometric and catalytic regimes.

## Graphical abstract

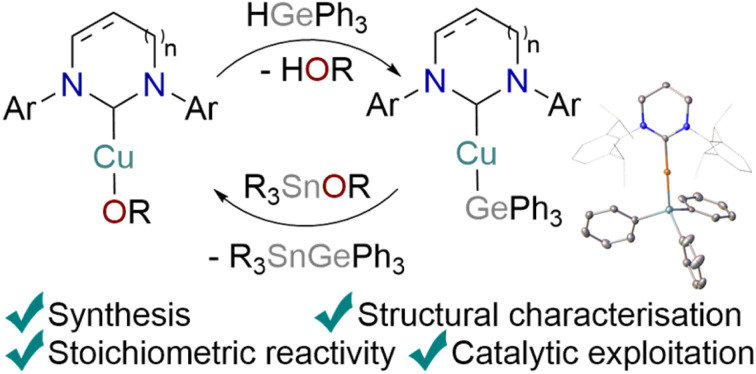

## Introduction

The structural characterisation of well-defined copper(i) silyls has been pivotal to their exploitation in catalysis. N-Heterocyclic Carbenes (NHCs) provide a tuneable coordination environment and have contributed significantly in the effort to access isolable copper(i) silyls. For example, the syntheses of (NHC)CuSi(TMS)_2_R (NHC = IMe, I*t*Bu, and R = TMS, Et) were described *via* salt metathesis of a metal silanide with copper(i) halides.^1^ The use of silylboranes as synthetic reagents to generate metal silyls *via* σ-bond metathesis has since gained significant attention.^2^ Kleeberg and co-workers reported the synthesis of (IPr)CuSiMe_2_Ph from the reaction of PhMe_2_SiBPin with (IPr)CuO*t*Bu.^3^ The same group expanded this approach to a series of NHC-copper(i) silyl compounds of formula (NHC)CuSiR_3_, (NHC = IPr, IMes, I*t*Bu, Me_2_IMe, and SiR_3_ = SiMe_2_Ph, SiPh_3_),^4^ while Van Hoveln and co-workers reported the synthesis (IPr)CuSi(OEt)_3_ from the reaction of Si_2_(OEt)_6_ with (IPr)CuO*t*Bu.^5^ NHC-copper(i) silyls have widespread utility as key intermediates in silylations of a swathe of unsaturated organic reagents, many of which feature a carbonyl group. For example, the reduction of CO_2_,^3^ as well as the silylation of Michael acceptors, dienones, dienoates, allylic chlorides, lactams, aldehydes, acyl chlorides, and others are well documented.^5–13^ This extensive applicability is, in part, driven by the consistent modes of reactivity offered by Cu–Si bonds.

Copper(i) organostannyls containing NHC ligands have also been explored; (IPr)CuSnPh_3_ was generated from the reaction of Ph_3_SnH with (IPr)CuX (X = H, O^*t*^Bu) by Sadighi.^14^ (IPr)CuSnMe_3_ and [(Me_2_IMe)CuSnMe_3_]_3_ were generated from the respective alkoxides and [CH_2_N(iPr)]_2_BSnMe_3_.^4^ (IPr)CuSnPh_3_ can act as a source of a phenyl anion^14^ upon reaction with carbon dioxide forming (IPr)CuOC(O)Ph, alongside a diphenyltin derivative. The authors proposed a number of associated reaction mechanisms^14^ and Ariafard, Yates and co-workers subsequently explored these *via* DFT calculations.^15^ We recently reported a series of ring-expanded NHC (RE-NHC) copper(i) triphenylstannyls, whose reactions with heterocumulenes also resulted in phenyl transfer and allowed us to propose an alternative mechanism associated with nucleophilic chemistry at tin in these systems.^16^

Unlike their tetrel congeners, copper(i) complexes of organogermyl anions are far less well-explored. The first triphenylphosphine supported copper(i) germyl compounds of the form (Ph_3_P)_*n*_CuGePh_3_ (*n* = 1, 3) were reported from the reaction of Ph_3_GeLi with (Ph_3_P)CuCl by Hooton.^17^ Bockarev and co-workers described the first structurally authenticated triphenylphosphine-copper(i) germyl compound, (Ph_3_P)_2_CuGe(C_6_F_5_)_3_, from the deprotonation of (C_6_F_5_)_3_GeH with *t*BuOCu in the presence of triphenylphosphine.^18^ The reaction of Yb with (Ph_3_P)_3_CuGePh_3_ was reported to yield [{Yb(THF)_6_}{(Ph_3_Ge)_2_Cu}_2_]·THF.^19^ Oshima and co-workers detailed the synthesis of two germyl cuprates, (Ph_3_Ge)_2_Cu(CN)Li_2_ and (Et_3_Ge)_2_Cu(SMe_2_)Li, which transferred the Ph_3_Ge-fragment to 1-dodecyne, providing the net hydrogermylation products as both regioisomers after hydrolysis.^20^ Germyl copper reagents have since been exploited to germylate α,β-unsaturated carbonyls, α,β-alkynic esters, and acyl chlorides.^21–23^ Beyond this, almost no exploration of copper(i) triphenylgermyls has been undertaken.

Until recently, organogermanium chemistry was significantly in the shadow of its lighter and heaver congener. Silicon and tin have strong precedent in organic chemistry, for example in the Hiyama and Stille couplings respectively. The high cost of germanium and a misperception of its character as being isolated to that of “big silicon” contributed to this oversight. In recent years, however, organogermanium systems have received growing acclaim as useful, orthogonal transmetallation group in a large and growing swathe of cross coupling reactions.^24,25^

Given the limited number of copper(i) triphenylgermyl complexes thus described, and their potential utility in installing increasingly synthetically useful germyl groups, we set out to explore the chemistry of the well-precedented NHC ligand class with these underexplored moieties. This investigation was particularly interesting and attractive in the context of the vast dissimilitude in reactivity between copper(i) silyls and stannyls. Herein, we report the synthesis and reactivity of a range of compounds of the form (NHC)CuGePh_3_ (NHC = SIMes, :C{N(Mes)CH_2_}_2_; IPr, :C{N(Dipp)CH}_2_; 6-Mes, :C{N(Mes)CH_2_}_2_CH_2_; and 6-Dipp, :C{N(Dipp)CH_2_}_2_CH_2_. Mes = 2,3,5-Me_3_C_6_H_2_; Dipp = 2,6-iPr_2_C_6_H_3_).

## Results and discussion

### Synthesis and characterisation of (NHC)CuGePh_3_

In an initial experiment, (SIMes)CuO*t*Bu was reacted with an equimolar amount of Ph_3_GeH in C_6_D_6_. Interrogation of the ^1^H NMR spectrum showed complete disappearance of the resonance associated with the germane hydride at 5.85 ppm within three days at 40 °C, as well as the formation of *t*BuOH. We interpreted these data to indicate the targeted formation of (SIMes)CuGePh_3_, compound 1. In contrast, extension of this approach to larger NHCs was unsuccessful, providing sluggish reactions and incomplete conversion to the anticipated products, even with extended heating. In the cases of IPr and 6-Dipp, replacement of the *tert*-butoxide leaving group by its smaller methoxide analogue afforded success, yielding (NHC)CuGePh_3_ (NHC = IPr, 2; 6-Dipp, 4). Attempts to extend this methodology to 6-Mes were hampered by significant challenges in the clean synthesis of (6-Mes)CuOMe, but reaction of (6-Mes)CuMes with Ph_3_GeH provided (6-Mes)CuGePh_3_ (compound 3).

**Fig. 1:**
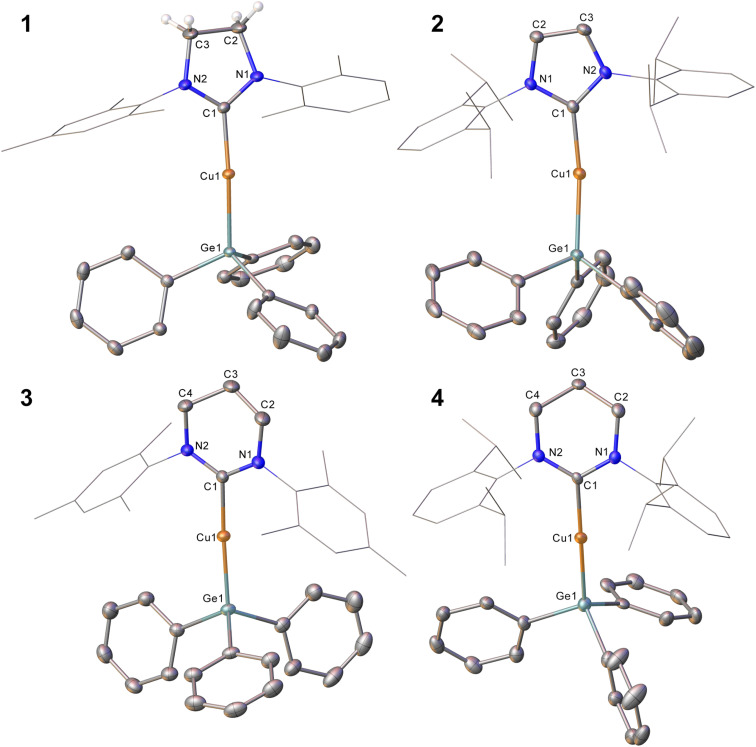
Molecular structures of compound 1–4. Hydrogen atoms (those attached to C2 and C3 excepted in 1) have been omitted for clarity, and the carbene substituents are represented in wireframe view. Ellipsoids are represented at 30% probability. Only one of the two molecules present in the asymmetric unit is shown for compound 2.

Compound 1 was crystallised from slow cooling of a saturated toluene solution, while crystals of compounds 2–4 were obtained from diffusion of hexane into saturated toluene solutions. The structures derived from SC-XRD of each of these species are shown in Fig. 1, with metric parameters in Table 1. Compounds 1–4 crystallise as monomers with C–Cu–Ge geometries that are close to linear (C–Cu–Ge angle (°): 1, 169.49(6); 2, 171.24(5), 171.36(5); 3, 173.48(5); 4, 171.77(16)), which slightly increase upon going from the 5- to 6-membered carbene ligands. A concomitant increasing trend in the C–Cu bond length is apparent (C–Cu distance (Å): 1, 1.9185(18); 2, 1.9126(16), 1.9182(16); 3, 1.9340(15); 4, 1.949(6)). These metrics are similar to those previously observed for (NHC)CuEPh_3_ systems (E = Si, Sn); in particular, Kleeberg^4^ reported a corresponding silicon system for IPr (*i.e.* the lighter congener of 2), its tin analogue was reported by Sadighi^14^ and the heavier congeners of 3 and 4 were described by us^16^ (relevant bond lengths (Å) and angles (°): (IPr)CuSiPh_3_, C–Cu, 1.9333(1); C–Cu–Si, 170.53(4); (IPr)CuSnPh_3_, C–Cu, 1.914(2); C–Cu–Sn, 169.6(8); (6-Mes)CuSnPh_3_, C–Cu, 1.927(3); C–Cu–Sn, 172.87(10); (6-Dipp)CuSnPh_3_, C–Cu, 1.934(3); C–Cu–Sn, 171.27(9)).^4,14,16^ Comparison of the Cu–Ge bond distances between 1–4 show only limited variations as a consequence of the identity of the NHC, whereas comparison of 2 to (IPr)CuSiPh_3_ and (IPr)CuSnPh_3_ (Cu–E bond length (Å): 2, 2.3038(3), 2.3085(3); (IPr)CuSiPh_3_, 1.9333(1); (IPr)CuSnPh_3_, 2.469(5)) exhibit unsurprising trends in the Cu–E bond lengths. A similar, predictable, lengthening of the Cu–E bond length when comparing 3 or 4 to their corresponding tin congeners (6-Dipp)CuSnPh_3_ and (6-Mes)CuSnPh_3_ is also observed (Cu–E bond length (Å): 3, 2.3045(3); (6-Mes)CuSnPh_3_, 2.4567(4); 4, 2.3456(12); (6-Dipp)CuSnPh_3_, 2.4742(4)).

**Table 1:** Selected bond lengths (Å) and angles (°) for (NHC)CuEPh_3_ and compounds 1–4

	1	2	3	4	(IPr)CuSiPh_3_ (ref. 4)	(IPr)CuSnPh_3_ (ref. 14)		(6-Mes)CuSnPh_3_ (ref. 16)	(6-Dipp)CuSnPh_3_ (ref. 16)
C–Cu	1.9185(18)	1.9126(16)	1.9340(15)	1.949(6)	1.9333(1)	1.914(2)	1.927(3)		1.934(3)
Cu–E	2.3078(3)	2.3038(3)	2.3045(3)	2.3456(12)	1.9333(1)	2.469(5)	2.4567(4)		2.4742(4)
C–Cu–E	169.49(6)	171.24(5)	173.48(5)	171.77(16)	170.53(4)	169.6(8)	172.87(10)		171.27(9)

### Computational analysis of (IPr)CuGePh_3_

Based on prior reports of both its silicon and tin congeners, and its trivial synthesis from easily accessible compounds, we selected compound 2 as a platform to investigate the reactivity of the Cu–Ge bond. In preparation for these reactions, we sought to characterise the nature of the Cu–Ge bond in 2*via* DFT calculations. Inspection of the structure optimised at the PBE0-D3BJ/BS2(C_6_H_6_)//BP86/BS1 level of theory provided a good match with the crystallographically characterised structure, albeit with some elongation of the bond parameters which likely reflects the effects of packing in the crystal structure (selected bonds (Å) and angles (°): computed; Cu–Ge, 2.363; C–Cu, 1.920; C–Cu–Ge, 177.77; derived from X-ray; Cu–Ge, 2.3038(3); C–Cu, 1.9126(16); C–Cu–Ge, 171.24(5)). The Wiberg bond index for the Cu–Ge bond (0.56) reflects a significantly polarised covalent interaction. To understand the bonding interactions in this species we analysed the compound further using the EDA-NOCV approach. The analysis indicated that the bonding between the copper and germyl fragments is most appropriately described as [(IPr)Cu]^+^ and [GePh_3_]^−^ moieties as this had the smallest orbital interaction energy of the studied fragment combinations (see ESI†). Further interrogation of the calculated orbital interaction energies by the NOCV method shows that the largest contribution, comprising 66.6% of the overall orbital interaction energies, originates from donation of electron density from germanium to copper (see ESI†).

### Reactions with electrophilic p-block halides

**Scheme 1:**
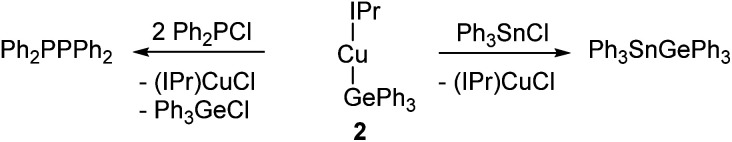
σ-Bond metathesis reactions of (IPr)CuGePh_3_, 2.

Confirming this analysis, compound 2 acted as a germyl nucleophile in σ-bond metathesis reactions (Scheme 1). Addition of 2 to Ph_3_SnCl resulted in the formation of (IPr)CuCl^26^ which could be observed in the ^1^H NMR spectrum alongside a new resonance in the ^119^Sn NMR spectrum at 160.3 ppm which we attributed to Ph_3_SnGePh_3_.^27^

When compound 2 was reacted with diphenyl chlorophosphine, instantaneous formation of resonances associated with (IPr)CuCl was observed in the ^1^H NMR spectrum. The ^31^P NMR spectrum, however, showed evidence of starting material, the expected σ-bond metathesis product, Ph_2_PGePh_3_, in the form of a resonance at −51.7 ppm, and a resonance at −15 ppm. This was attributed to Ph_2_PPPh_2_ based on literature precedent, and reinterpretation of the ^1^H NMR allowed assignments of resonances in the phenyl region associated with Ph_3_GeCl. Addition of more Ph_2_PCl resulted in consumption of the resonance at −51.7 ppm in the ^31^P NMR spectrum and an increase in intensity of the peak associated with Ph_2_PPPh_2_. We interpret these results to imply that the reaction between 2 and Ph_2_PCl does in fact form Ph_2_PGePh_3_, but that this compound readily undergoes a dehalogermylation with another equivalent of Ph_2_PCl to generate Ph_2_PPPh_2_ and Ph_3_GeCl. This reaction is analogous to well-precedented dehalosilylation reactions to generate P–P bonds.

### Reactions with heterocumulenes

**Scheme 2:**
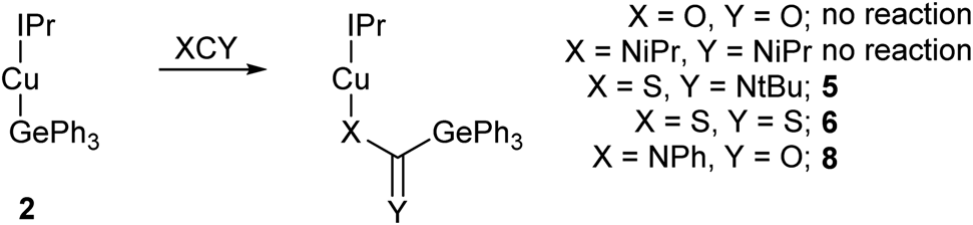
The reaction of (IPr)CuGePh_3_, 2, with a range of heterocumulenes.

Reactions with heterocumulenes were then investigated (Scheme 2) to assess the propensity of the Cu–Ge bond towards insertion reactions. Addition of carbon dioxide, or di-iso-propyl carbodiimide, to 2, provided no evidence of reactivity and even with extended reaction times only starting materials were isolable. This behaviour contrasts sharply with (IPr)CuSnPh_3_ which was shown to transfer a phenyl group to both of these reagent classes to generate (IPr)CuX_2_CPh (X = N(*p*-tol), O)^14,16^ with concurrent formation of “Ph_2_Sn” and with the observation that NHC-copper(i) silyls have been reported to deoxygenate CO_2_ to generate CO.^3^

**Fig. 2:**
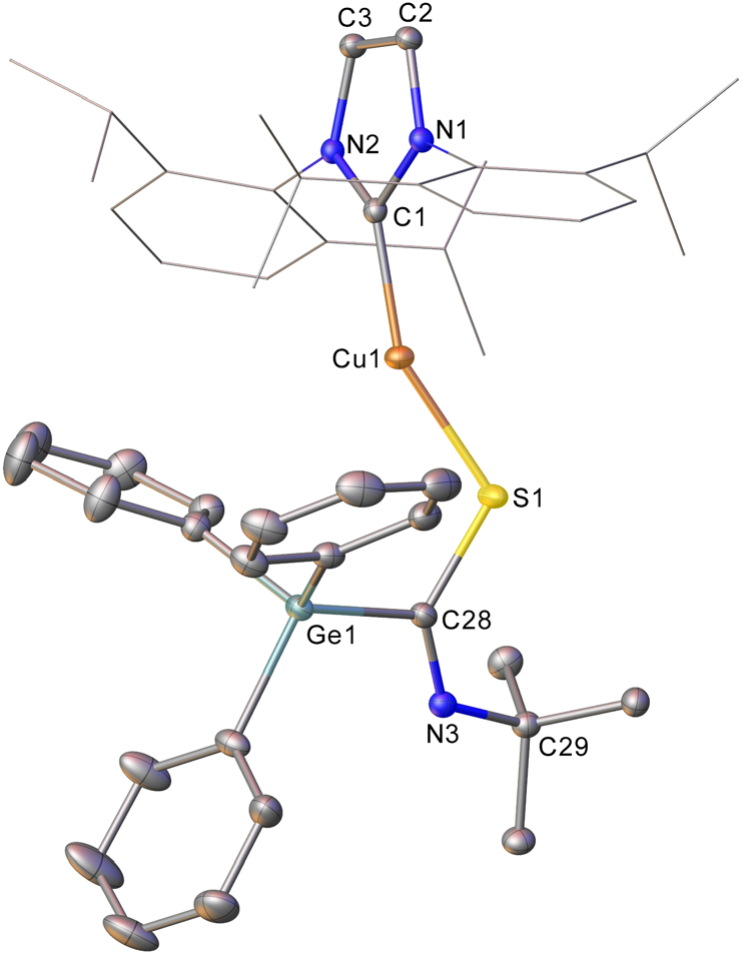
Molecular structure of compound 5. Hydrogen atoms have been omitted and the carbene substituents are represented in wireframe view, for clarity. Ellipsoids are represented at 30% probability. Selected bond length (Å) and angle (°) data: C1–Cu1, 1.9070(14); Cu1–S1, 2.1538(4); S1–C28, 1.7827(15); C28–N3, 1.276(2); C28–Ge1, 1.9699(15); Cu1–S1–C28, 115.06(5); S1–C28–N3, 128.61(12).

In contrast, reacting sulfur containing heterocumulene with 2 provided more success. Addition of one equivalent of *tert*-butyl isothiocyanate to a solution of 2 provided ^1^H NMR data consistent with the formation of a new compound containing both the IPr ligand and a *tert*-butyl group. Diffusion of hexane into a saturated toluene solution provided crystals suitable for single crystal X-ray diffraction, which revealed the identity of the product to be (IPr)CuSC(=N*t*Bu)GePh_3_ (compound 5, Fig. 2), thereby confirming the nucleophilicity of the germanium centre in 2. Compound 5 arises from insertion of the isothiocyanate C=S bond into the Cu–Ge bond. As such, the GePh_3_ fragment is transferred onto the carbon of *t*BuNCS with a corresponding new Cu–S bond being formed alongside a C–S single bond (1.7827(15) Å). The N=C bond remains intact, with a bond length of 1.276(2) Å and a S–C–N angle of 128.61(12)°. The Ge–C28 distance (1.9699(15) Å) is also consistent with a single bond. To our knowledge, 5 constitutes the first report of a heavy tetrel substituted thioamidate, albeit as the κ_1_-sulfur linkage isomer. The heavier analogue of 3, (6-Mes)CuSnPh_3_ was found to generate (6-Mes)CuSC(NPh)Ph when reacted with phenyl isothiocyanate. During this reaction, we observed ^119^Sn NMR spectroscopy and mass spectrometry data consistent with the formation of the tin analogue of 5, (6-Mes)CuSC(NPh)SnPh_3_, but proposed that it was unstable with respect to extrusion of “SnPh_2_”. We thus investigated the thermolysis of compound 5 which provided no evidence of “Ph_2_Ge” extrusion after heating a benzene-*d*_6_ solution thereof in a sealed tube at 120 °C overnight.

**Fig. 3:**
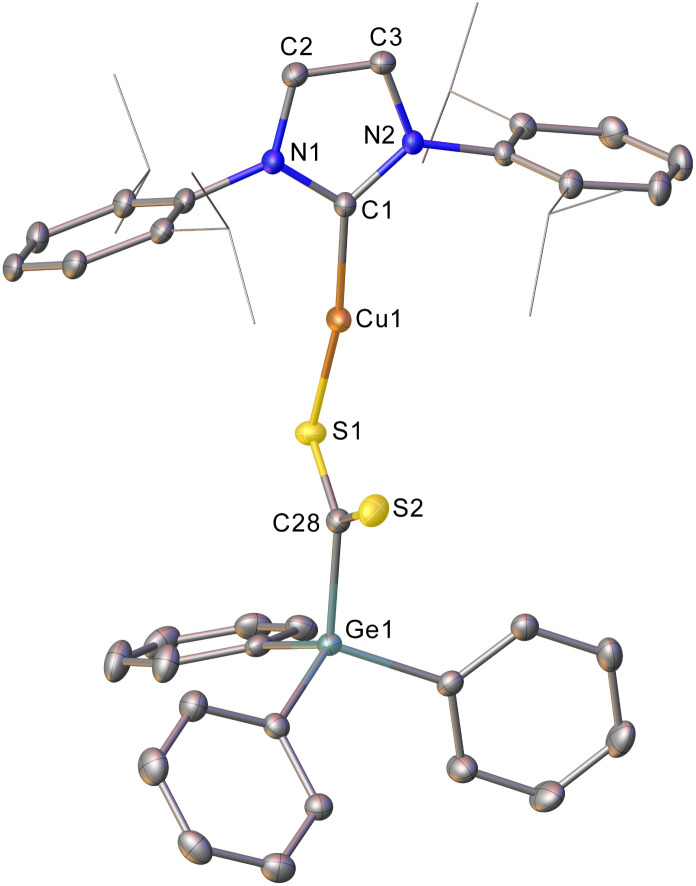
One of the two molecules in the asymmetric unit of compound 6. Hydrogen atoms have been omitted and the carbene substituents are represented in wireframe view, for clarity. Ellipsoids are represented at 30% probability. Selected bond length (Å) and angle (°) data: C1–Cu1, 1.903(2); Cu1–S1, 2.1944(7); S1–C28, 1.698(2); C28–S2, 1.651(2); C28–Ge1, 1.983(2); Cu1–S1–C28, 99.97(8); S1–C28–S2, 124.69(14).

Addition of CS_2_ to a C_6_D_6_ solution of 2 was similarly successful, providing a ^1^H NMR spectrum consistent with consumption of 2 and the formation of a new compound. Once again, slow diffusion of hexane into a saturated toluene solution provided material appropriate for SC-XRD which indicated the product of this reaction to be (IPr)CuSC(S)GePh_3_ (compound 6, Fig. 3).

Compound 6 reflects nucleophilic transfer of the intact germyl moiety, once again *via* the addition of the Cu–Ge bond across a C=S bond. This generates a rare example of a heavy tetrel substituted dithiocarboxylate,^28^ which binds to the copper in a κ_1_ fashion. The terminal sulfur shows a bond distance consistent with some multiple bonding (1.651(2) Å), whilst the coordinated sulfur atom shows a longer bond (1.698(2) Å) reflecting the cleavage of the π-component of the carbon sulfur bond. The geometry at C28 is approximately trigonal planar (S1–C28–S2, 124.69(14)°) and the C–Ge bond distance (1.983(2) Å) is consistent with that in compound 5.

**Fig. 4:**
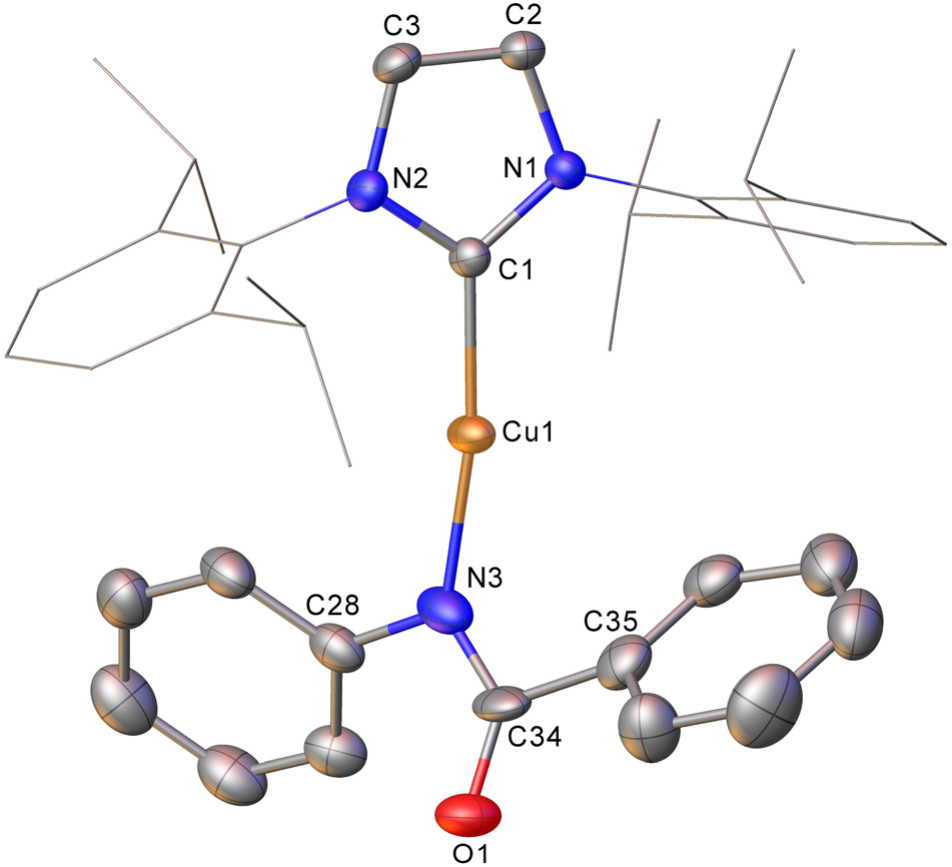
Molecular structure of compound 7. Hydrogen atoms have been omitted and the carbene substituents are represented in wireframe view, for clarity. Ellipsoids are represented at 30% probability.

In contrast to these reactions involving intact triphenyl germyl transfer, addition of phenyl isocyanate to 2 provided crystals of a product, characterised by SC-XRD as (IPr)CuN(Ph)C(O)Ph (compound 7, Fig. 4). Compound 7 is a two-coordinate copper(i) benzamidinate with a κ_1_–N binding mode and while the chemical characterisation is unambiguous, the data quality preclude extensive interpretation of the associated metrics (see ESI†). We previously proposed compound 7 formed from the reaction of (IPr)CuSnPh_3_ with PhNCO with concomitant generation of “SnPh_2_”. In the case of the reaction of 2 with phenyl isocyanate, no evidence of “GePh_2_” was observed, but the precipitation of a small amount of grey material we interpreted to be germanium metal and the presence of GePh_4_ in both the ^1^H NMR and mass spectra of the reaction medium were taken to imply its transient formation followed by disproportionation.

**Fig. 5:**
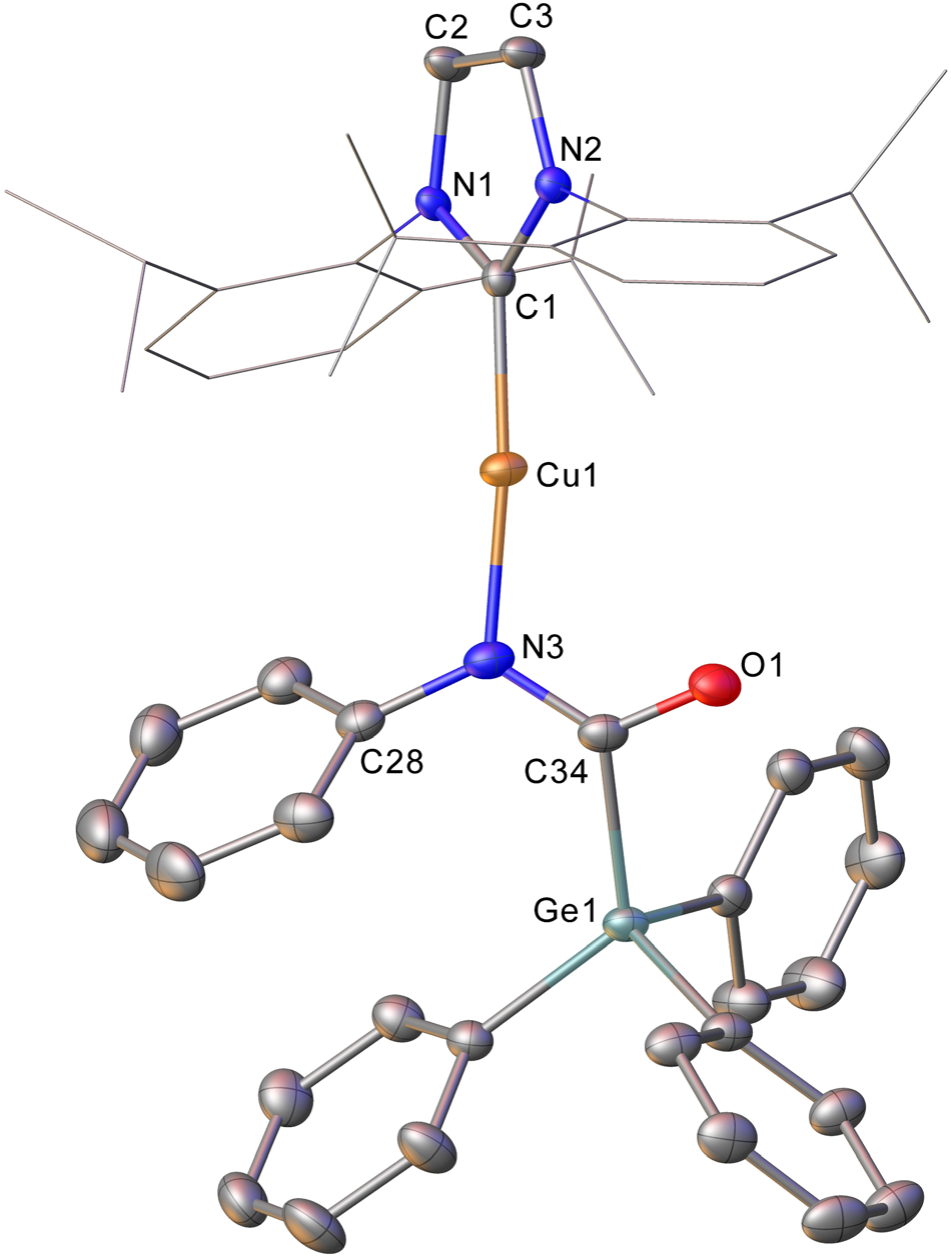
Molecular structure of compound 8. Hydrogen atoms have been omitted and the carbene substituents are represented in wireframe view, for clarity. Ellipsoids are represented at 30% probability. Selected bond length (Å) and angle (°) data: C1–Cu1, 1.864(3); Cu1–N3, 1.872(3); N3–C34, 1.328(4); C34–O1, 1.237(4); C34–Ge1, 2.005(3); C1–Cu1–N3, 172.62(13); N3–C34–Ge1 125.1(2).

Intrigued by this first example of germanium behaviour that was similar to its heavy congener, we reinterrogated the reaction *via in situ*^1^H NMR spectroscopy. Addition of PhNCO to 2 in C_6_D_6_ provided evidence of starting material consumption, which over the course of a week was complete. The ^1^H NMR spectrum of the arising solution, however, indicated the major product to be the germaamidate compound (IPr)CuN(Ph)C(O)GePh_3_, 8, as opposed to compound 7, which was structurally characterised (Fig. 5). Compound 8 affords another example of a polar π-bond insertion into the Cu–Ge bond which results in the formation of two new σ-bonds. Transfer of the GePh_3_ moiety onto the isocyanate carbon gives a C–Ge single bond (C34–Ge1, 2.005(3) Å) while the isocyanate C=O double bond is retained (C34–O1, 1.237(4) Å). This comes at a cost of C=N cleavage to yield a formally anionic nitrogen atom (N3–C34, 1.328(4) Å) and an approximately trigonal planar germaamide carbon (N3–C34–Ge1 125.1(2)). In fact, compound 8 is, to the best of our knowledge, the first solid-state, structurally characterised germanium substituted amide derivative.

Heating compound 8 in C_6_D_6_ at 120 °C over four days, surprisingly, provided no evidence of the formation 7, Ge, and GePh_4_. Instead, reformation of 2 alongside triphenyl isocyanurate [PhNC(O)]_3_ was observed. This suggests that (IPr)CuN(Ph)C(O)GePh_3_ is capable of undergoing a deinsertion to regenerate 2 and PhNCO. Trimerisation of PhNCO is well known, and may be mediated by any free IPr ligand present, or *via* successive insertions into the Cu–N bond of 8 followed by germyl elimination to reform 2. Some evidence for this latter mechanism was procured from an attempt to react phenyl isocyanate with 1 which, despite an equimolar stoichiometry of these reagents, consistently yielded (SIMes)CuN(Ph)C(O)N(Ph)C(O)GePh_3_ (see ESI†) suggesting that the nucleophilicity of the anionic nitrogen in the germaamidate exceeds that of the germanium atom in the copper(i) germyl. These observations, alongside significant difficulty in isolating anything but trace amounts of 7 from such reactions, were deduced to reflect that, while germanium chemistry can parallel that of tin (*i.e.*, 2 can undertake phenyl transfer reactions analogous to its heaver congener), such reactivity is disfavoured with respect to other pathways and only contributes a small amount of the reactivity of 2.

These findings also complicate the significance of (IPr)CuX_2_CEPh_3_ (X = NR, O; E = Ge, Sn) as intermediates in the mechanism of phenyl transfer from copper(i) triphenyltetranides to heterocumulenes which we previously proposed. While their relevance cannot be ruled out at this stage, such systems may be off-route towards the phenyl transfer reaction. Reactions may then proceed *via* a deinsertion of the heterocumulene. In the case of tin, these may be phenyl transfer reactions occurring *via* the pathways previously proposed (and investigated by DFT), whereas in the case of germanium, competing trimerisation of the heterocumulene can occur. Alternatively, however, these data may simply reflect the difference in the Ge–C *versus* Sn–C bond strengths. In the tin case, weaker bonding allows much more facile, and consistent transfer of the phenyl moiety under mild conditions. In contrast, the strong Ge–C bonding demands more forcing conditions for phenyl transfer which also open up other reaction pathways.

### Reactions with carbon–carbon multiple bonds

**Scheme 3:**
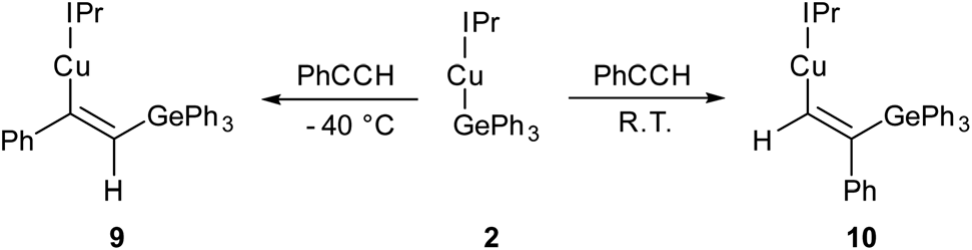
The outcomes of the reaction of (IPr)CuGePh_3_, 2, with phenyl acetylene at two temperatures, with possible transition states inset.

**Fig. 6:**
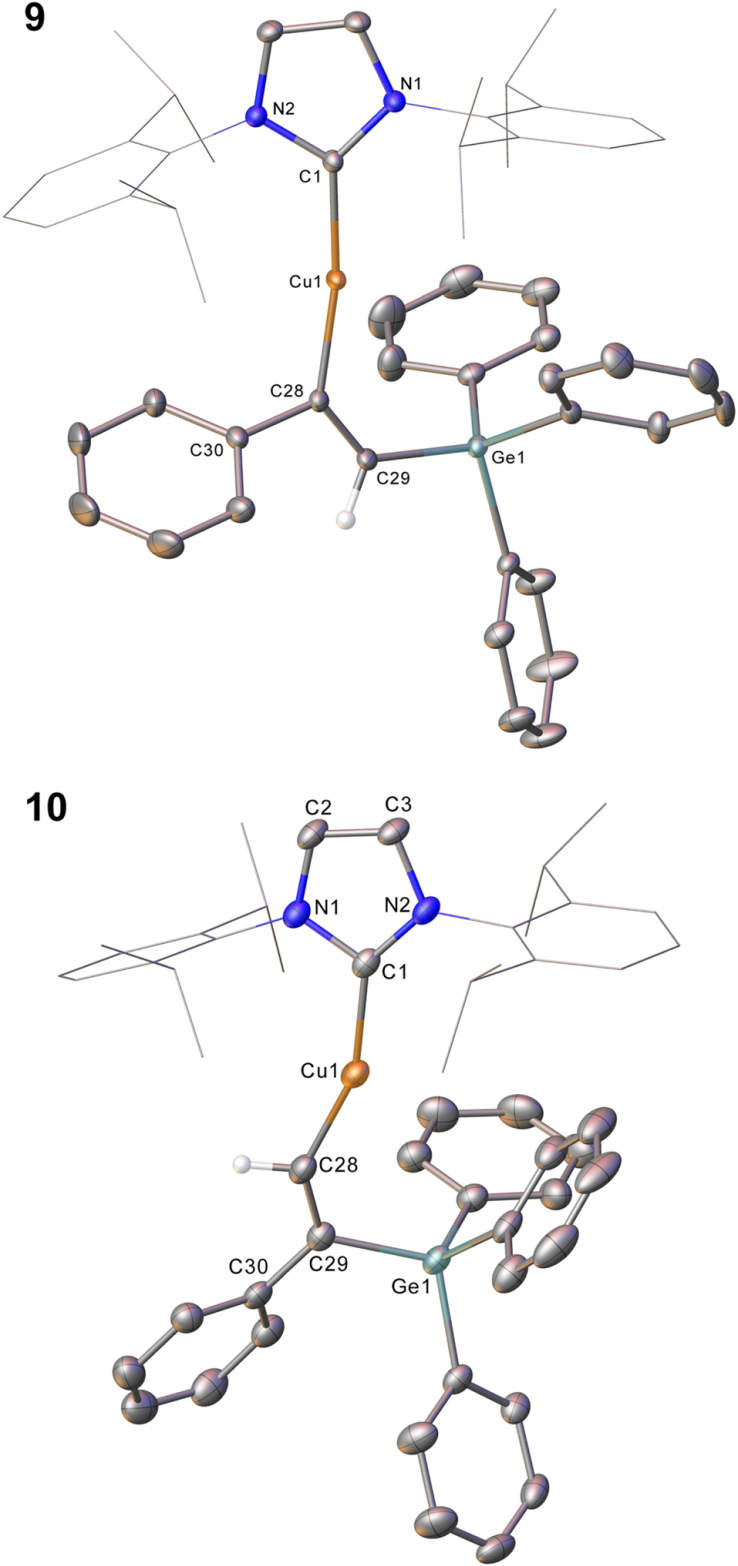
Molecular structure of compound 9 and 10. Hydrogen atoms (H29 excepted) have been omitted for clarity, and the carbene substituents are represented in wireframe view, also for perspicuity. Ellipsoids are represented at 30% probability. Selected bond length (Å) and angle (°) data: 9, C1–Cu1, 1.9177(16); Cu1–C28, 1.9262(16); C28–C29, 1.345(2); C29–Ge1, 1.9241(16); C1–Cu1–C28, 169.64(7); Cu1–C28–C29, 133.66(13); C28–C29–Ge1, 130.44(13); 10, C1–Cu1, 1.923(3); Cu1–C28, 1.916(3); C28–C29, 1.350(4); C29–Ge1, 1.944(3); C1–Cu1–C28, 158.52(12); Cu1–C28–C29, 140.9(2); C28–C29–Ge1, 119.3(2).

Compound 2 showed no reaction with ethylene, or internal alkynes, however reactivity towards a terminal alkyne (Scheme 3) was observed. Addition of one equivalent of phenyl acetylene to 2 in C_6_D_6_ provided ^1^H NMR spectroscopic data consistent with the formation of a number of products. We thus repeated the reaction at low temperature (*ca.* −40 °C) and isolated a crystalline material which we found to be (IPr)CuC(Ph)=C(H)GePh_3_ (compound 9, Fig. 6).

Compound 9 is the product of an insertion of the C–C triple bond of phenyl acetylene into the Cu–Ge bond. The product retains a C–C double bond (C28–C29, 1.345(2) Å) with Cu–C and Ge–C single bonds (Cu1–C28, 1.9262(16); C29–Ge1, 1.9241(16) Å). The angles at the carbons bonded to copper Cu1–C28–C29 (133.66(13)°) and C28–C29–Ge1 (130.44(1)°) further reflect the persistence of multiple bonding at the C–C fragment, and the copper and germanium atoms are *cis*-oriented with respect to one another.

A repeat of this reaction, at room temperature and with a slight deficit of phenyl acetylene (0.8 equivalents) allowed isolation of an isomer of 9, (IPr)CuC(H)=C(Ph)GePh_3_ (compound 10). While strenuous attempts to acquire any more than trace quantities of 10 were unsuccessful, it was characterised by single crystal X-ray crystallography (Fig. 6). Metric parameters associated with 10 largely parallel those of 9, with the exception of those in the Cu–C–C–Ge fragment, where the decreased steric clash at the formally anionic C28 produces larger angles (Cu1–C28–C29: 10, 140.9(2); 9, 133.66(13)°), and the corresponding steric crowding at C29 results in decreased angles (C28–C29–Ge1: 10, 119.3(2); 9, 130.44(13)°).

**Scheme 4:**
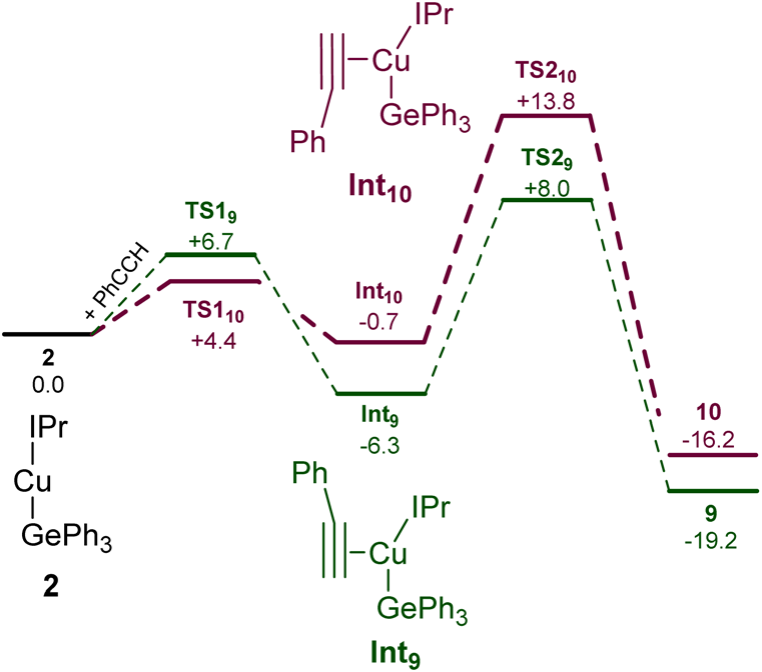
The calculated pathways for the reaction of (IPr)CuGePh_3_, 2, with phenyl acetylene at the BP86-D3BJ/BS2(C_6_H_6_)//BP86/BS1 level of theory.

Surprised by the formation of both isomers of insertion, we turned to computational methods to provide greater insight into this observation. The energies of compounds 2, 9, and 10 were determined by DFT (BP86-D3BJ/BS2(C_6_H_6_)//BP86/BS1), and we interrogated the barriers for the conversion of 2 into 9 and 10 respectively (Scheme 4). Both 9 and 10 are more stable than the starting materials, the former by −19.2 kcal mol^−1^ and the latter by −16.2 kcal mol^−1^. The marginally higher relative stability of 9 is unsurprising based on the effect of a phenyl substituent adjacent to the formally carbanionic carbon on copper. The formation of each species was calculated to occur *via* the formation of two intermediate π-complexes where the alkyne coordinates the copper centre,^29,30^Int_9_ and Int_10_. The formation of these occurs with low barriers in each case, with access to Int_10_ being marginally more facile (towards Int_9_, 6.7 kcal mol^−1^; towards Int_10_, 4.4 kcal mol^−1^). In both cases, the π-complexes are slightly more stable with respect to the starting materials but this effect is more pronounced in the case of Int_9_*versus*Int_10_, (−6.3 and −0.7 kcal mol^−1^ respectively). Migratory insertion of the alkyne into the Cu–Ge bond then occurs to produce 9 and 10. In both cases this insertion is associated with an accessible barrier, albeit higher in the case of the reaction towards 10 (Int_9_ → 9, 8.0 kcal mol^−1^; Int_10_ → 10 13.6 kcal mol^−1^). These data indicate that under all regimes, 9 is expected to be the dominant product as it is both kinetically and thermodynamically favoured.

Attempts to characterise the ratio of 9 and 10 in solution at a range of temperatures, however, proved challenging. At low temperature, 9 is the dominant species in solution, alongside small amounts of 10, (IPr)CuCCPh and PhC(GePh_3_)=CH_2_. At room temperature, however, the proportions of (IPr)CuCCPh and PhC(GePh_3_)=CH_2_ are much higher. Based on these data, and the DFT results we propose that the insertion to generate 9 is somewhat reversible, and small amounts of 10 form *via* the less favoured pathway. Once formed, 10 however is a competent base towards phenyl acetylene and at room temperature this reaction proceeds rapidly. As 9 and 10 are in equilibrium, small amounts of 10 thus form at room temperature and *via* repeated equilibration/deprotonation, significant generation of (IPr)CuCCPh and PhC(GePh_3_)=CH_2_ is observed. In contrast, we did not observe any data suggesting the formation of PhC(H)=C(H)GePh_3_, which we attribute to the stabilising effect of the phenyl substituent on the formally carbanionic carbon in 9 which renders it insufficiently basic to deprotonate phenyl acetylene.

### Catalytic reactions of copper(i) germyls

**Scheme 5:**
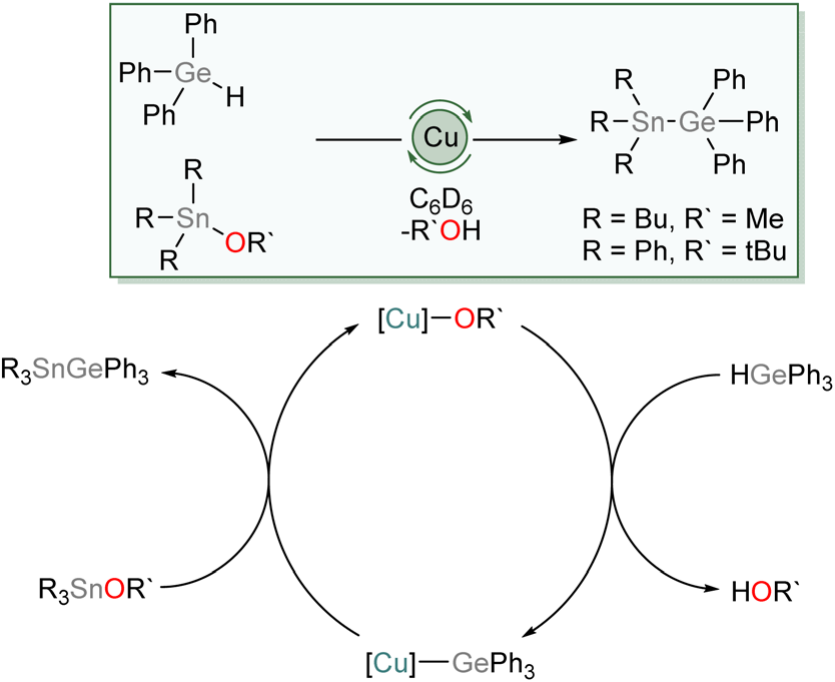
The proposed mechanism of tin/germanium cross coupling catalysed by (SIMes)CuO*t*Bu.

We then investigated the possibility of catalytic exploitation of NHC-supported copper(i) germyls. Initially, we focussed on σ-bond metathesis reactions in the hope of generating new cross-coupling reactions. We selected 1 as a possible intermediate hoping that its reduced steric demand might enhance reactivity. Addition of 10 mol% of (SIMes)CuO*t*Bu to an equimolar mixture of Ph_3_GeH and Ph_3_SnCl provided no catalytic turnover. Inspired by the synthesis of 1–4 by reaction of Ph_3_GeH with copper(i) alkoxides and our prior work,^31^ we instead turned our attention to the exploitation of tin alkoxides as coupling partners. Addition of an equivalent of Ph_3_GeH to (SIMes)CuO*t*Bu in the presence of Bu_3_SnOMe in C_6_D_6_ provided evidence of formation of Ph_3_GeSnBu_3_ and (SIMes)CuOMe. Moreover, addition of 10 mol% of (SIMes)CuO*t*Bu to an equimolar mixture of Ph_3_GeH and Ph_3_SnO*t*Bu provided spectroscopic evidence of quantitative formation of Ph_3_GeSnPh_3_ and *t*BuOH overnight at 100 °C. Similarly, Bu_3_SnOMe coupled quantitatively with Ph_3_GeH in the presence of 10 mol% of (SIMes)CuO*t*Bu when the side product, methanol, was removed *in vacuo* (Scheme 5).

We propose this reaction occurs *via* a series of σ-bond metathesis steps, and can be considered a sp^3^–sp^3^ heavy tetrel cross-coupling. Attempts to extend this reactivity towards coupling of Ph_3_GeH with tin diesters or oxides in a 2 : 1 ratio (Bu_2_Sn(OMe)_2_, Bu_2_SnO, Ph_2_SnO); to equimolar reactions lighter tetrel esters (Ph_3_GeOMe, Me_3_GeOMe, Ph_3_SiOMe); or to phosphinites (Ph_2_POEt) provided no evidence of cross-coupling.

Work in insertion reactions was initially less productive. Attempts to catalytically hydrogermylate heterocumulenes were unproductive, and under no conditions could evidence of deprotonation of triphenyl germane by 5, 6 or 8 be observed. Work with alkynes was also disappointing; when an excess of phenyl acetylene was added to compound 2 and triphenyl germane, no catalytic turnover was observed. Instead, ^1^H NMR spectroscopic data was consistent with the formation of (IPr)CuCCPh and PhC(GePh_3_)=CH_2_. We recently reported that the deprotonation of phenyl acetylene by a copper(i) boryl imidinate produced (IPr)CuCCPh,^32^ and we propose a similar mechanism is operant here, with 9 deprotonating phenyl acetylene. (IPr)CuCCPh is, unfortunately from the perspective of productive catalysis, inert and shows no ability to deprotonate triphenyl germane thus precluding catalytic turnover. Such unproductive side reactivity could be obviated by removing the acidic proton of the terminal acetylene. Given the reluctance of 2 to react with internal alkynes, it was further unsurprising to see that no hydrogermylation of diphenyl acetylene was observed in the presence of (SIMes)CuO*t*Bu.

**Scheme 6:**
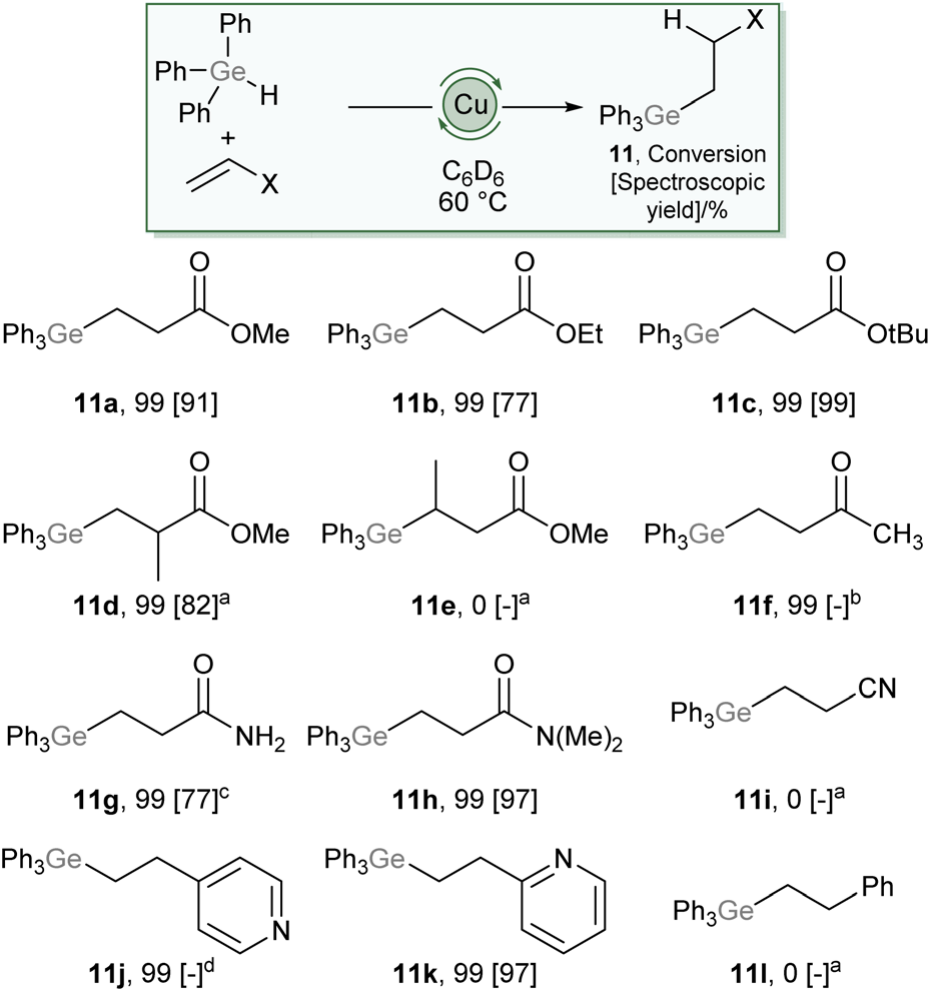
Scope of the hydrogermylation of Michael-acceptors catalysed by 10 mol% (SIMes)CuO*t*Bu. For full details see ESI,† values in brackets are spectroscopic yields relative to an internal standard. (a) 100 °C, (b) 80 °C, provided an intractable mixture with no evidence of desired product, (c) reaction performed in *d*_8_-THF at 100 °C, (d) provided an intractable mixture including desired product.

Compound 2 also showed no ability to insert ethylene, and correspondingly no conditions were found to proffer catalytic hydrogermylation of ethene. However, addition of 10 mol% (SIMes)CuO*t*Bu to an equimolar mixture of Ph_3_GeH and methyl acrylate showed complete consumption of the acrylate overnight at 60 °C and ^1^H NMR data consistent with hydrogermylation. This data contained, alongside resonances associated with phenyl and –OMe groups, a pair of complex multiplets at 2.44 and 1.83 ppm which integrated in a 2 : 2 ratio. These data allowed us to unambiguously assign the product as Ph_3_GeCH_2_CH_2_C(O)OMe (11a), which forms *via* a conjugate addition of a germyl nucleophile to the Michael acceptor.^22,23^ This provides a remarkable contrast to reactions of silanes, R_3_SiH, which consistently provide reactivity associated with addition of the hydride nucleophile under copper catalysis. The scope of this reaction was then explored (Scheme 6), and found to tolerate a range of carboxylate derivatives including acrylic esters and amides (11a–c, 11g–h), methyl methacrylate (11d) as well as a vinyl pyridine (11k). Substitution of the β-position had a detrimental effect on reactivity (11e) and less electrophilic alkenes, such as methyl vinyl ketone (11f), acrylonitrile (11i), and styrene (11l) provided undesired or no reactivity. Given the widespread exploitation of Ge–C bonds as carbon-nucleophiles in cross-coupling,^24,25^ this reaction provides synthons for β-nucleophilic Michael acceptor fragments, an interesting example of Umpolung character conferred by the ambiphilicity of germanium.

## Conclusions

These results allow us to begin to comprehend the reactivity of Cu–Ge bonds compared to their lighter and heavier analogues. The formation of the germyl anion by deprotonation of the corresponding germane indicates a degree of ambiphilicity of the Ge–H bond, reminiscent of Ph_3_SnH which can be deprotonated by copper alkoxides. In contrast, once installed the Cu–GePh_3_ fragment showed reactivity much more consistent with its silyl analogue. (IPr)CuGePh_3_ (2) is a source of the germyl nucleophile towards a range of substrates. In the reaction with p-block halides it is competent in the formation of new Ge–Sn and Ge–P bonds *via* σ-bond metathesis. The general nucleophilicity of the germyl fragment extends to the reaction with sulfur-containing heterocumulenes, allowing the isolation of two previously unreported heavy atom substituted analogues of organic functional groups. The reaction with phenyl isocyanate was more complex, somewhat reflecting behaviour reminiscent of its tin congener, with phenyl transfer occurring. This, however, did not appear to be the dominant mode of reactivity, and thermolysis did not enhance the amount of phenyl transfer. The reaction of phenyl acetylene with 2 allowed us to investigate the previously noted unselective insertion of alkynes into Cu–Ge bonds. This remarkably diverse range of reactions show that copper(i) triphenylgermyls are like neither their lighter nor heavier congeners, but show a unique chemistry. Finally, we were able to validate the potential of NHC-copper(i) germyls as catalytic intermediates, describing a tin/germanium cross coupling between triphenyl germane and two tin(iv) alkoxides. This reaction occurred with concomitant formation of an equivalent of alcohol, and was proposed to occur *via* deprotonation of triphenyl germane by a copper(i) alkoxide. Moreover, catalytic hydrogermylation of Michael acceptors was also viable and the product isomer indicated the intermediacy of a copper(i) germyl. These results show that NHC-supported copper(i) germyls are convenient synthons for installing the GePh_3_ group into a range of molecules and we expect these observations to inspire catalytic and stoichiometric exploitation of these systems in the coming years, bringing them towards a prominence approaching that of their silicon congeners.

## Data availability

Full synthetic and characterisation data (NMR, MS, CHN, SCXRD) and full details of computational analysis available in ESI.† Crystallographic data for all compounds have been deposited with the Cambridge Crystallographic Data Centre as the following supplementary CCDC publications: 2221285–2221292 for compound 1 (2221285), compound 2 (2221286), compound 3 (2221288), compound 4 (2221287), compound 5 (2221289), compound 6 (2221290), compound 7 (2221291), compound 8 (2249130), compound 9 (2221292), compound 10 (2249131) and (SIMes)CuN(Ph)C(O)N(Ph)C(O)GePh_3_ (2249132).

## Author contributions

RSCC; synthetic investigation, data curation, writing – original draft; NJE; synthetic investigation; LEE; computational investigation, writing – original draft; SEN, writing – review & editing; PV, funding acquisition, methodology, resources, supervision, writing – review & editing; MFM, data curation, formal analysis, supervision, visualisation, writing – review & editing; DJL, conceptualisation, funding acquisition, methodology, resources, project administration, supervision, writing – original draft.

## Conflicts of interest

There are no conflicts to declare.

## Supplementary Material

SC-015-D3SC05862J-s001

SC-015-D3SC05862J-s002
