# Supplementary material for: The structures and reactivity of NHC-supported copper(i) triphenylgermyls
Source: Chem Sci. 2023 Dec 1;15(2):584–93. doi: 10.1039/d3sc05862j (PMC10763552; doi:10.1039/d3sc05862j)
Supplement: SC-015-D3SC05862J-s001 [file SC-015-D3SC05862J-s001.pdf]

Electronic Supplementary Material (ESI) for Chemical Science.
This journal is © The Royal Society of Chemistry 2023

**Supplementary Information for**

# The Structures and Reactivity of NHC-supported Copper(I) Triphenylgermyls

Rex S. C. Charman, Nick J. Evans, Laura E. English, Samuel E. Neale, Petra Vasko, Mary F. Mahon, David J. Liptrot

**Experimental** 4

General Considerations and Starting Materials 4
Synthesis of (6-Dipp)CuOMe 4
Synthesis of $Ph_3SnOtBu$ 5
Synthesis of compound **1**, (SIMes)$CuGePh_3$ 5
Synthesis of compound **2**, (IPr)$CuGePh_3$ 5
Synthesis of compound **3**, (6-Mes)$CuGePh_3$ 6
Synthesis of compound **4**, (6-Dipp)$CuGePh_3$ 6
Synthesis of compound **5**, (IPr)CuSC(=NtBu)$GePh_3$ 7
Synthesis of compound **6**, (IPr)CuSC(S)$GePh_3$ 7
Synthesis of compound **7**, (IPr)CuN(Ph)C(O)Ph 7
Synthesis of compound **8**, (IPr)CuN(Ph)C(O)$GePh_3$ 8
Synthesis of compound **9**, (IPr)CuC(Ph)C(H)$GePh_3$ 8
Synthesis of compound **10**, (IPr)CuC(H)=C(Ph)$GePh_3$ 8
Reaction of compound **2** and $Ph_3SnCl$ 9
Reaction of compound **2** and $Ph_2PCl$ 9
Reaction of compound **2** and carbon dioxide 9
$^1$H VT-NMR study of the reaction of compound **2** and carbon dioxide 9
Reaction of compound **2** and di-iso-propyl carbodiimide 9
Thermolysis of compound **5** 10
Reaction of compound **2** and phenyl isocyanate 10
Thermolysis of compound **8** 10
Reaction of compound **1** and phenyl isocyanate 10
Reaction of compound **2** and phenyl acetylene at -40 °C 10
Reaction of compound **2** and of phenyl acetylene at room temperature 10
Reaction of 10 mol% compound **2** with $Ph_3GeH$ and phenyl acetylene 11
Reaction of 10 mol% (SIMes)CuOtBu with $Ph_3GeH$ and $Ph_3SnCl$ 11
Reaction of (SIMes)CuOtBu with $Ph_3GeH$ and $Bu_3SnOMe$ 11
General Procedure for the Catalytic Cross Coupling Reactions 11
Catalytic Cross Coupling Reactions 11
General Procedure for the Catalytic Hydrogermylation of Activated Alkenes 12
Catalytic Hydrogermylation of Activated Alkenes 12

**X-ray Crystallography** 15

X-ray data discussion 15
Figure S1. Molecular structure of (SIMes)CuN(Ph)C(O)N(Ph)C(O)$GePh_3$ 16
Table S1. Crystal data and structure refinement details (compounds **1**-**4**) 17
Table S2. Crystal data and structure refinement details (compounds **5**-**8**) 18
Table S3. Crystal data and structure refinement details (compounds **9**-**10** and (SIMes)CuN(Ph)C(O)N(Ph)C(O)$GePh_3$) 19

***In situ* reaction monitoring by NMR spectroscopy** 20

Figures S2: Reaction of (SIMes)CuOtBu and $Ph_3GeH$ 20

Figure S3: Reaction of (IPr)CuOMe and $Ph_3GeH$ **20**
Figure S4: Reaction of (6-Mes)CuMes and $Ph_3GeH$ **21**
Figure S5: Reaction of (6-Dipp)CuOMe and $Ph_3GeH$ **21**
Figure S6: Reaction of (IPr)$CuGePh_3$ and tBuNCS **22**
Figure S7: Reaction of (IPr)$CuGePh_3$ and $CS_2$ **22**
Figure S8: Reaction of (IPr)CuPh and PhNCO **23**
Figure S9-10: Reaction of compound **2** and $Ph_3SnCl$ **23**
Figures S11-12: Reaction of compound **2** and $Ph_2PCl$ **24**
Figure S13: Reaction of compound **2** and carbon dioxide **25**
Figure S14: $^{1}H$ VT-NMR spectra of the reaction of compound **2** and carbon dioxide **26**
Figure S15: Reaction of compound **2** and di-iso-propyl carbodiimide **26**
Figure S16: Thermolysis of compound **5** **27**
Figure S17: Reaction of compound **2** and phenyl isocyanate **27**
Figure S18: Thermolysis of compound **8** **28**
Figure S19: Reaction of compound **1** and phenyl isocyanate **28**
Figure S20: Reaction of compound **2** and phenyl acetylene at -40 °C **29**
Figure S21: Reaction of compound **2** and phenyl acetylene at room temperature **29**
Figure S22: The $^{1}H$–$^{13}C$ HSQC NMR spectrum of the reaction of compound **2** and two equivalents of phenyl acetylene at room temperature **30**
Figure S23: Reaction of 10 mol% compound **2** with phenyl acetylene and $Ph_3GeH$ **30**
Figures S24-25: Reaction of 10 mol% (SIMes)CuOtBu with $Ph_3GeH$ and $Ph_3SnCl$ **31**
Figures S26-27: Reaction of (SIMes)CuOtBu with $Ph_3GeH$ and $Bu_3SnOMe$ **32**
Figures S28-29: Reaction of 10 mol% (SIMes)CuOtBu with $Ph_3GeH$ and $Bu_3SnOMe$ **33**
Figures S30-31: Reaction of 10 mol% (SIMes)CuOtBu with $Ph_3GeH$ and $Ph_3SnOtBu$ **34**
Figure S32: Reaction of 10 mol% (SIMes)CuOtBu with $Ph_3GeH$ and methyl vinyl ketone **35**
Figure S33: Reaction of 5 mol% (SIMes)CuOtBu with $Ph_3GeH$ and acrylonitrile **35**
Figure S34: Reaction of 10 mol% (SIMes)CuOtBu with $Ph_3GeH$ and 4-vinyl pyridine **36**

**NMR Spectra of Compounds** **37**

Figures S35-36: NMR spectra of (6-Dipp)CuOMe **37**
Figures S37-39: NMR spectra of $Ph_3SnOtBu$ **38**
Figures S40-42: NMR spectra of $Ph_3GeSnBu_3$ **39**
Figures S43-44: NMR spectra of compound **1**, (SIMes)$CuGePh_3$ **41**
Figures S45-46: NMR spectra of compound **2**, (IPr)$CuGePh_3$ **42**
Figures S47-48: NMR spectra of compound **3**, (6-Mes)$CuGePh_3$ **43**
Figures S49-50: NMR spectra of compound **4**, (6-Dipp)$CuGePh_3$ **44**
Figures S51-52: NMR spectra of compound **5**, (IPr)CuSC(=NtBu)$GePh_3$ **45**
Figures S53-54: NMR spectra of compound **6**, (IPr)CuSC(S)$GePh_3$ **46**
Figures S55-56: NMR spectra of compound **7**, (IPr)CuN(Ph)C(O)Ph **47**
Figures S57-58: NMR spectra of compound **8**, (IPr)CuN(Ph)C(O)$GePh_3$ **48**
Figures S59-60: NMR spectra of compound **9**, (IPr)CuC(Ph)C(H)$GePh_3$ **49**
Figures S61-62: NMR spectra of compound **11a**, $Ph_3GeCH_2CH_2C(O)OCH_3$ **50**
Figures S63-64: NMR spectra of compound **11b**, $Ph_3GeCH_2CH_2C(O)OCH_2CH_2$ **51**

Figures S65-66: NMR spectra of compound **11c**, $Ph_3GeCH_2CH_2C(O)OC(CH_3)_3$ **52**
Figures S67-68: NMR spectra of compound **11d**, $Ph_3GeCH_2(CH_3)CHC(O)OCH_3$ **53**
Figures S69-70: NMR spectra of compound **11g**, $Ph_3GeCH_2CH_2C(O)NH_2$ **54**
Figures S71-72: NMR spectra of compound **11h**, $Ph_3GeCH_2CH_2C(O)NMe_2$ **55**
Figures S73-74: NMR spectra of compound **11k**, $Ph_3GeCH_2CH_2(2\text{-}C_5H_5N)$ **56**

**Computational details** **57**

**References** **68**

# Experimental

## General Considerations and Starting Materials

All reactions dealing with air- and moisture-sensitive compounds were carried out under argon atmosphere using standard Schlenk line and glovebox techniques. NMR experiments using air sensitive compounds were conducted in J. Young's tap NMR tubes prepared and sealed in a glovebox under argon. Hexane, toluene, and pentane were purified using an MBraun Solvent Purification System and stored over 4Å molecular sieves. Benzene, diethyl ether, and THF were dried with a sodium benzophenone suspension and distilled. $C_6D_6$ and toluene-$d_8$ was dried over a potassium mirror prior to vacuum transfer into a sealed ampoule and stored in a glove box under argon. Methanol was dried over magnesium turnings and distilled. All NMR data, unless otherwise stated, were acquired at 298 K on an Agilent ProPulse or Bruker Avance instrument for $^1$H (500 MHz), $^{13}$C{$^1$H} (126 MHz), and $^{119}$Sn (186 MHz) and a Bruker Avance 400 for $^1$H (400 MHz), $^{13}$C{$^1$H} (101 MHz) and $^{31}$P{$^1$H} (162 MHz). $^1$H and $^{13}$C NMR spectra were referenced using residual solvent resonances. Elemental analyses were performed by Elemental Microanalysis Ltd., Okehampton, Devon, U.K. $Ph_3GeH$ and $Ph_3SnCl$ were purchased from Merck and was used as received. Dibutyldimethoxytin was purchased from TCI and was used as received. Sodium methoxide and tributyltin methoxide were purchased from Fisher and were used as received. Phenyl isocyanate, *tert*-butyl isothiocyanate, $Ph_2POEt$, and $Ph_2PCl$ were purchased from Merck and distilled before use. Carbon disulfide was purchased from Merck and dried over calcium hydride prior to vacuum transfer into reactions. Carbon dioxide was purchased from Boc and atmospheres were applied through an Agilent 1/4 inch OT3-4 oxygen/moisture trap. Phenylacetylene was purchased from Fluorochem and was used as received. Diphenyltin oxide was purchased from Strem and was used as received. Methyl acrylate, ethyl acrylate, tertbutyl acrylate, methyl methacrylate, methyl crotonate, acrylonitrile, and 4-vinyl pyridine were purchased from Fisher and were distilled before use. Acrylamide, N,N-dimethylacrylamide, styrene, methyl vinyl ketone, and 2-vinyl pyridine were purchased from Merck and were distilled before use. (SIMes)CuOtBu, (IPr)CuPh, (IPr)CuOMe, (IPr)CuOtBu, (6-Mes)CuMes, (6-Dipp)CuCl, $Ph_3GeLi$, $Me_3GeOMe$, $Ph_3GeOMe$, and $Ph_3SiOMe$ were prepared according to literature procedures.[1]

## Synthesis of (6-Dipp)CuOMe

In a glovebox, (6-Dipp)CuCl (1.254 g, 2.49 mmol) and NaOMe (0.135 g, 2.50 mmol) were added to a Schlenk flask. Methanol (40 mL) was added forming a yellow suspension which was left stirring overnight at room temperature. Volatiles were removed *in vacuo* precipitating a beige powder. Benzene (20 mL) was added and resulting suspension was filtered forming a pale yellow solution. Volatiles were removed *in vacuo* precipitating an off-white powder which was washed with hexane (3 x 10 mL) yielding the product, (6-Dipp)CuOMe (0.475 g, 0.95 mmol, 38 %).

$^1$H NMR (500 MHz, $C_6D_6$) δ 7.19 – 7.17 (m, 2H, *para-H*), 7.08 (d, *J* = 7.8 Hz, 4H, *meta-H*), 3.92 (s, 3H, O$CH_3$), 3.03 (hept, *J* = 6.8 Hz, 4H, C*H*($CH_3$)$_2$), 2.73 (t, *J* = 5.9 Hz, 4H, N$CH_2$), 1.52 – 1.47 (m, 14H, CH($CH_3$)$_2$ and N$CH_2CH_2$), 1.19 (d, *J* = 7.0 Hz, 12H, CH($CH_3$)$_2$). $^{13}$C NMR (126 MHz, $C_6D_6$) δ 203.2 (*C*Cu), 145.7 (Ar-*C*), 142.5 (Ar-*C*), 129.3 (Ar-*C*), 124.8 (Ar-*C*), 46.1 (N$CH_2$), 28.9 (*C*H($CH_3$)$_2$), 25.0 (CH($CH_3$)$_2$), 24.6 (CH($CH_3$)$_2$), 20.4 (N$CH_2CH_2$). Analysis calculated for $C_{29}H_{43}CuN_2O$ (MW = 499.21 g/mol): Expected: C, 69.77; H, 8.68; N, 5.61. Found: C, 69.37; H, 8.88; N, 5.26. See figures S35-36 for full NMR spectra of (6-Dipp)CuOMe.

**Synthesis of $Ph_3SnOtBu$**

In a glovebox, $Ph_3SnCl$ (0.900 g, 2.33 mmol) and KOtBu (0.262 g, 2.33 mmol) were added to a Schlenk flask. Hexane (30 mL) was added forming a colourless suspension which was left stirring overnight at room temperature. The resulting suspension was filtered, and volatiles were removed *in vacuo* precipitating a colourless powder of the product, $Ph_3SnOtBu$ (0.814 g, 1.92 mmol, 82 %). $^1$H NMR (500 MHz, $C_6D_6$) δ 7.78 – 7.63 (m, 6H, Ar-*H*), 7.19 – 7.07 (m, 11H, Ar-*H* and residual $C_6H_6$), 1.37 (s, 9H, SnO*tBu*). 13C NMR (126 MHz, $C_6D_6$) δ 140.3 (Ar-*C*), 136.9 (Ar-*C*), 129.9 (Ar-*C*), 129.0 (Ar-*C*), 72.4 (O*C*($CH_3$)$_3$), 34.1 (OC(*C*$H_3$)$_3$). $^{119}$Sn NMR (186 MHz, $C_6D_6$) δ -114.3. See figures S37-39 for full NMR spectra of $Ph_3SnOtBu$.

**Synthesis of compound 1, (SIMes)CuGePh$_3$**

In a glovebox, (SIMes)CuOtBu (0.300 g, 0.68 mmol) and $Ph_3GeH$ (0.206 g, 0.68 mmol) were added to a Schlenk flask. Diethyl ether (30 mL) was added forming a white suspension which was left stirring overnight at room temperature. Volatiles were removed *in vacuo* precipitating a white powder which was washed with cold hexane (3 x 10 mL) yielding the product, compound **1** (0.355 g, 0.53 mmol, 78 %). Single crystals of compound **1** that were suitable for characterisation by SC-XRD were grown from the slow cooling of a saturated toluene solution overnight.

$^1$H NMR (500 MHz, $C_6D_6$) δ 7.57 – 7.54 (m, 6H, Ar-*H*), 7.20 – 7.17 (m, 4H, Ar-*H*), 7.17 – 7.12 (m, 9H, Ar-*H* and residual benzene), 6.73 – 6.72 (m, 4H, *meta-H*), 2.97 (s, 4H, NC*H*$_2$), 2.11 (s, 6H, *para*-C*H*$_3$), 2.10 (s, 12H, *ortho*-C*H*$_3$). $^{13}$C NMR (126 MHz, $C_6D_6$) δ 204.5 (*C*Cu), 150.3 (Ar-*C*), 138.5 (Ar-*C*), 136.8 (Ar-*C*), 135.7 (Ar-*C*), 135.5 (Ar-*C*), 130.0 (Ar-*C*), 127.5 (Ar-*C*), 126.3 (Ar-*C*), 50.5 (N*C*H$_2$), 21.1 (*para*-*C*H$_3$), 18.1 (*ortho*-*C*H$_3$). Analysis calculated for $C_{39}H_{41}CuGeN_2$ (MW = 673.95 g/mol): Expected: C, 69.51; H, 6.13; N, 4.16. Found: C, 69.04; H, 6.15; N, 4.02. See figure S2 for the in-situ reaction $^1$H NMR spectrum and figures S43-44 for full NMR spectra of **1**.

**Synthesis of compound 2, (IPr)CuGePh$_3$**

In a glovebox, (IPr)CuOMe (0.780 g, 1.61 mmol) and $Ph_3GeH$ (0.492 g, 1.61 mmol) were added to a Schlenk flask. Toluene (30 mL) was added forming a colourless solution which was stirred overnight at room temperature. Volatiles were removed *in vacuo* precipitating a white powder which was washed with hexane (3 x 10 mL). The white powder was dissolved in the minimum amount of toluene and filtered forming a colourless solution. The solution was cooled to -5 °C overnight precipitating a colourless crystalline material of the product, compound **2** (0.440 g, 0.58 mmol, 36 %). Single crystals of compound **2** were that were suitable for characterisation by SC-XRD were grown from the slow diffusion of hexane into a saturated toluene solution.

$^1$H NMR (500 MHz, $C_6D_6$) δ 7.51 – 7.49 (m, 6H, Ar-*H*), 7.26 – 7.21 (m, 2H, Ar-*H*), 7.20 – 7.13 (m, 11H, Ar-*H* and residual benzene), 7.05 (d, *J* = 7.9 Hz, 4H, *meta-H*), 6.21 (s, 2H, NC*H*), 2.53 (hept, *J* = 6.9 Hz, 4H, C*H*(CH$_3$)$_2$), 1.27 (d, *J* = 7.0 Hz, 12H, CH(C*H*$_3$)$_2$), 1.05 (d, *J* = 7.0 Hz, 12H, CH(C*H*$_3$)$_2$). $^{13}$C NMR (126 MHz, $C_6D_6$) δ 183.6 (*C*Cu), 150.3 (Ar-*C*), 145.9 (Ar-*C*), 136.8 (Ar-*C*), 134.8 (Ar-*C*), 130.7 (Ar-*C*), 127.5 (Ar-*C*), 126.2 (Ar-*C*), 124.3 (Ar-*C*), 122.2 (N*C*H), 29.0 (*C*H(CH$_3$)$_2$), 25.3 (CH(*C*H$_3$)$_2$), 23.6 (CH(*C*H$_3$)$_2$). Analysis calculated for $C_{45}H_{51}CuGeN_2$ (MW = 756.09 g/mol): Expected: C, 71.49; H, 6.80; N, 3.71. Found: C, 70.96; H, 6.82; N, 3.69. See figure S3 for the in-situ reaction $^1$H NMR spectrum and figures S45-46 for full NMR spectra of **2**.

**Synthesis of compound 3, (6-Mes)CuGePh$_3$**

In a glovebox, (6-Mes)CuMes (111 mg, 0.220 mmol) and Ph$_3$GeH (67.2 mg, 0.220 mmol) were added to a Schlenk flask. Toluene (10 mL) was added forming a colourless solution which was stirred for 3 days at 60 °C. Volatiles were removed *in vacuo* forming a white powder which was washed with hexane (3 x 10 mL) yielding the product, compound **3** (75.5 mg, 0.110 mmol, 50 %). Single crystals of compound **3** that were suitable for characterisation by SC-XRD were grown from the slow diffusion of hexane into a saturated toluene solution.

$^{1}$H NMR (500 MHz, C$_6$D$_6$) δ 7.45 – 7.44 (m, 6H, Ar-*H*), 7.23 – 7.12 (m, 11H, Ar-*H* and residual benzene), 6.75 (s, 4H, *meta-H*), 2.46 (t, *J* = 5.9 Hz, 4H, NC*H*$_2$), 2.18 (s, 6H, *para*-C*H*$_3$), 2.06 (s, 12H, *ortho*-C*H*$_3$), 1.32 – 1.27 (m, 2H, NCH$_2$C*H*$_2$). $^{13}$C NMR (126 MHz, C$_6$D$_6$) δ 202.2 (*C*Cu), 150.8 (Ar-*C*), 141.8 (Ar-*C*), 138.1 (Ar-*C*), 136.8 (Ar-*C*), 134.9 (Ar-*C*), 130.0 (Ar-*C*), 127.3 (Ar-*C*), 126.1 (Ar-*C*), 43.4 (N*C*, 21.2 (*para*-*C*H$_3$), 20.6 (NCH$_2$*C*H$_2$), 18.1 (*ortho*-*C*H$_3$). MS (ESI) [M+] C$_{40}$H$_{43}$CuGeN$_2$: 685.1998, found: 685.2069 (err [ppm] = 4.63). Despite repeated attempts, we were unable to obtain satisfactory elemental analysis for this compound. This is most likely due to the compound's high sensitivity towards air and moisture. Analysis calculated for C$_{40}$H$_{43}$CuGeN$_2$ (MW = 687.97 g/mol): Expected: C, 69.83; H, 6.3; N, 4.07. Found: C, 68.22; H, 6.17; N, 3.88. See figure S4 for the in-situ reaction $^{1}$H NMR spectrum and figures S47-48 for full NMR spectra of **3**.

**Synthesis of compound 4, (6-Dipp)CuGePh$_3$**

(6-Dipp)CuOMe route:

In a vial in a glovebox, (6-Dipp)CuOMe (30 mg, 0.060 mmol) and Ph$_3$GeH (27 mg, 0.089 mmol) were combined. Benzene (0.5 mL) was added forming a yellow solution which was transferred into a J Young's NMR tube. The reaction was heated to 40 °C for 4 days and volatiles were removed *in vacuo* precipitating a yellow oil. The oil was triturated with hexane (0.5 mL) precipitating a beige powder. The powder was redissolved in toluene (0.5 mL) and filtered forming a colourless solution. Crystals of compound **4** that were suitable for characterisation by SC-XRD were grown from the slow diffusion of hexane. Despite repeated attempts at purification, this route yielded material that was impure by NMR spectroscopy. Crystalline yield: (6-Dipp)CuGePh$_3$ (8.5 mg, 0.011 mmol, 18 %). See figure S5 for the in-situ reaction $^{1}$H NMR spectrum and figures S49-50 for full NMR spectra of **4**.

(6-Dipp)CuCl route:

In a vial in a glovebox, (6-Dipp)CuCl (30 mg, 0.060 mmol) and Ph$_3$GeLi (19 mg, 0.060 mmol) were combined. Diethyl ether (0.5 mL) was added forming a white suspension which was transferred into a J Young's NMR tube. The reaction was heated to 40 °C overnight and volatiles were removed *in vacuo* precipitating a white powder. The powder was redissolved in toluene (0.5 mL) and filtered forming a colourless solution. Crystals of compound **4** were grown from the slow diffusion of hexane with a 5% impurity of (6-Dipp)CuCl, (6-Dipp)CuGePh$_3$ (4.4 mg, 0.006 mmol, 10 %).

$^{1}$H NMR (500 MHz, C$_6$D$_6$) δ 7.36 – 7.32 (m, 6H, Ar-*H*), 7.29-7.26 (m, 2H, Ar-*H*), 7.22 – 7.17 (m, 8H, Ar-*H*), 7.09 (d, *J* = 7.8 Hz, 4H, *meta-H*), 2.99 – 2.90 (m, 4H, C*H*(CH$_3$)$_2$), 2.66 (t, *J* = 5.9 Hz, 4H, NC*H*$_2$), 1.47 – 1.41 (m, 2H, NCH$_2$C*H*$_2$), 1.29 – 1.24 (m, 14H, NCH$_2$C*H*$_2$ and CH(C*H*$_3$)$_2$), 1.14 (d, *J* = 7.0 Hz, 12H, CH(C*H*$_3$)$_2$). $^{13}$C NMR (126 MHz, C$_6$D$_6$) δ 202.3 (*C*Cu), 150.5 (Ar-*C*), 145.9 (Ar-*C*), 141.4 (Ar-*C*), 136.8 (Ar-*C*), 129.5 (Ar-*C*), 127.2 (Ar-*C*), 126.0 (Ar-*C*), 125.0 (Ar-*C*), 45.9 (N*C*H$_2$), 28.9 (*C*H(CH$_3$)$_2$), 25.2 (CH(*C*H$_3$)$_2$), 24.6 (CH(*C*H$_3$)$_2$), 20.2 (NCH$_2$*C*H$_2$). MS (ESI) [M+K] C$_{46}$H$_{55}$CuGeN$_2$K: 811.2544, found: 811.2502 (err [ppm] = -1.19).

Analysis calculated for $C_{46}H_{55}CuGeN_2$ (MW = 772.14 g/mol): Expected: C, 71.56; H, 7.18; N, 3.63. Found: C, 71.20; H, 7.09; N, 3.55. No in-situ NMR spectra were collected for **4** via this synthetic pathway.

**Synthesis of compound 5, (IPr)CuSC(=NtBu)GePh$_3$**

In a vial in a glovebox, (IPr)CuGePh$_3$ (30 mg, 0.040 mmol) was dissolved in benzene (0.5 mL). *tert*-Butyl isothiocyanate (5.0 µL, 0.040 mmol) was added forming a pale yellow solution. The reaction mixture was transferred into a J Young's NMR tube and placed at 40 °C for 10 days. Volatiles were removed *in vacuo* precipitating a beige powder. The powder was dissolved in the minimum amount of toluene and single crystals of compound **5** that were suitable for characterisation by SC-XRD were grown from the slow diffusion of hexane, (IPr)CuSC(=NtBu)GePh$_3$ (8 mg, 0.009 mmol, 20 %).

$^1$H NMR (500 MHz, $C_6D_6$) δ 7.85 – 7.81 (m, 6H, Ar-*H*), 7.27 – 7.23 (m, 2H, Ar-*H*), 7.15 – 7.09 (m, 13H, Ar-*H*), 6.37 (s, 2H, NC*H*), 2.77 (hept, *J* = 6.8 Hz, 4H, C*H*(CH$_3$)$_2$), 1.36 (d, *J* = 6.8 Hz, 12H, CH(C*H*$_3$)$_2$), 1.08 (d, *J* = 6.8 Hz, 12H, CH(C*H*$_3$)$_2$), 0.60 (s, 9H, NC(C*H*$_3$)$_3$). $^{13}$C NMR (126 MHz, $C_6D_6$) δ 187.0 (*C*Cu), 146.0 (Ar-*C*), 144.4 (Ar-*C*), 135.8 (Ar-*C*), 135.3 (Ar-*C*), 130.4 (Ar-*C*), 127.7 (Ar-*C*), 124.2 (Ar-*C*), 122.5 (Ar-*C*), 29.7 (NC(*C*H$_3$)$_3$), 29.0 (*C*H(CH$_3$)$_2$), 24.5 (CH(*C*H$_3$)$_2$), 24.4 (CH(*C*H$_3$)$_2$). MS (ESI) [M-H]$^-$ $C_{50}H_{59}CuGeN_3S$: 869.2984, found: 869.3023 (err [ppm] = 0.75). Analysis calculated for $C_{50}H_{60}CuGeN_3S$ (MW = 871.29 g/mol): Expected: C, 68.93; H, 6.94; N, 4.82. Found: C, 69.22; H, 6.90; N, 3.98. See figure S6 for the in-situ reaction $^1$H NMR spectrum and figures S51-52 for full NMR spectra of **5**.

**Synthesis of compound 6, (IPr)CuSC(S)GePh$_3$**

In a vial in a glovebox, (IPr)CuGePh$_3$ (30 mg, 0.040 mmol) was dissolved in benzene (0.5 mL) and was transferred into a J Young's NMR tube. $CS_2$ (4.7 µL, 0.079 mmol) was added via vacuum transfer forming a pale green solution. Volatiles were removed *in vacuo* yielding the product, (IPr)CuSC(S)GePh$_3$ (19.7 mg, 0.024 mmol, 60 %). Single crystals of compound **6** were that were suitable for characterisation by SC-XRD were grown from the slow diffusion of hexane into a saturated toluene solution.

$^1$H NMR (500 MHz, $C_6D_6$) δ 7.83 – 7.78 (m, 6H, Ar-*H*), 7.21 – 7.18 (m, 2H, Ar-*H*), 7.14 – 7.08 (m, 9H, Ar-*H*), 7.06 (d, *J* = 7.8 Hz, 4H, *meta-H*), 6.32 (s, 2H, NC*H*), 2.62 (hept, *J* = 6.9 Hz, 4H, C*H*(CH$_3$)$_2$), 1.37 (d, *J* = 6.8 Hz, 12H, CH(C*H*$_3$)$_2$), 1.07 (d, *J* = 6.8 Hz, 12H, CH(C*H*$_3$)$_2$). $^{13}$C NMR (126 MHz, $C_6D_6$) δ 192.7 (*C*S$_2$), 184.4 (*C*Cu), 145.8 (Ar-*C*), 138.0 (Ar-*C*), 136.1 (Ar-*C*), 135.3 (Ar-*C*), 130.6 (Ar-*C*), 128.9 (Ar-*C*), 128.1 (Ar-*C*), 124.2 (Ar-*C*), 122.4 (N*C*H), 29.0 (*C*H(CH$_3$)$_2$), 24.8 (CH(*C*H$_3$)$_2$), 24.1 (CH(*C*H$_3$)$_2$). Analysis calculated for $C_{46}H_{51}CuGeN_2S_2$ (MW = 832.22 g/mol): Expected: C, 66.39; H, 6.18; N, 3.37. Found: C, 66.26; H, 6.11; N, 3.25. See figure S7 for the in-situ reaction $^1$H NMR spectrum and figures S53-54 for full NMR spectra of **6**.

**Synthesis of compound 7, (IPr)CuN(Ph)C(O)Ph**

In a vial in a glovebox, (IPr)CuPh (30 mg, 0.057 mmol) was dissolved in benzene (0.5 mL) and PhNCO (9.2 µL, 0.085 mmol) was added forming a colourless suspension. The reaction was left overnight at room temperature before the suspension was transferred into a J Young's NMR tube. Volatiles were removed *in vacuo* precipitating a colourless powder which was washed with hexane (3 x 1 mL) yielding compound **7**, (IPr)CuN(Ph)C(O)Ph (14.6 mg, 0.023 mmol, 40 %).

$^1$H NMR (400 MHz, $C_6D_6$) δ 7.98 – 7.96 (m, 2H, Ar-*H*), 7.23 – 7.19 (m, 2H, Ar-*H*), 7.16 – 7.11 (m, 16H, Ar-*H* and residual benzene), 7.05 – 7.00 (m, 2H, Ar-*H*), 6.99 – 6.97 (m, 4H, *meta-H*), 6.94 – 6.88 (m, 2H, Ar-*H*), 6.75 – 6.71 (m, 2H, Ar-*H*), 6.16 (s, 2H, NC*H*), 2.41 (hept, *J* = 6.9 Hz, 4H, C*H*(CH$_3$)$_2$), 1.06 (d, *J* = 6.9 Hz, 12H, CH(C*H*$_3$)$_2$), 0.99 (d, *J* = 6.9 Hz, 12H, CH(C*H*$_3$)$_2$).

$^{13}C$ NMR (101 MHz, $C_6D_6$) δ 182.0 (*C*(O)), 173.5 (*C*Cu), 151.4 (Ar-*C*), 145.4 (Ar-*C*), 144.4 (Ar-*C*), 135.1 (Ar-*C*), 130.8 (Ar-*C*), 128.4 (Ar-*C*), 128.2 (Ar-*C*), 127.9 (Ar-*C*), 127.7 (Ar-*C*), 126.3 (Ar-*C*), 124.6 (*meta*-*C*), 123.0 (N*C*H), 121.0 (Ar-*C*), 28.9 (*C*H(CH$_3$)$_2$), 24.4 (CH(*C*H$_3$)$_2$), 23.8 (CH(*C*H$_3$)$_2$). Analysis calculated for $C_{40}H_{46}CuN_3O$ (MW = 648.37 g/mol): Expected: C, 74.10; H, 7.15; N, 6.48. Found: C, 73.72; H, 6.82; N, 6.12. See figure S8 for the in-situ reaction $^1H$ NMR spectrum and figures S55-56 for full NMR spectra of **7**.

**Synthesis of compound 8, (IPr)CuN(Ph)C(O)GePh$_3$**

In a vial in a glovebox, (IPr)CuGePh$_3$ (30 mg, 0.040 mmol) was dissolved in benzene (0.5 mL) and PhNCO (5.4 µL, 0.050 mmol) was added forming a colourless solution. The reaction was left for 7 days at room temperature and was then transferred into a J Young's NMR tube. Volatiles were removed *in vacuo* precipitating a beige powder which was dissolved in the toluene (0.5 mL). Single crystals of compound **8** that were suitable for characterisation by SC-XRD were grown from the slow diffusion of hexane, (IPr)CuN(Ph)C(O)GePh$_3$ (4.4 mg, 0.005 mmol, 13 %).

$^1H$ NMR (400 MHz, $C_6D_6$) δ 7.68 – 7.64 (m, 6H, Ar-*H*), 7.24 – 7.20 (m, 2H, Ar-*H*), 7.09 – 7.05 (m, 13H, Ar-*H*), 6.59 – 6.52 (m, 3H, Ar-*H*), 6.42 – 6.39 (m, 2H, Ar-*H*), 6.28 (s, 2H, NC*H*), 2.59 (hept, *J* = 6.8 Hz, 4H, C*H*(CH$_3$)$_2$), 1.35 (d, *J* = 6.9 Hz, 12H, CH(C*H*$_3$)$_2$), 1.07 (d, *J* = 6.9 Hz, 12H, CH(C*H*$_3$)$_2$). $^{13}C$ NMR (101 MHz, $C_6D_6$) δ 184.6 (*C*(O)), 183.0 (*C*Cu), 148.9 (Ar-*C*), 145.9 (Ar-*C*), 140.1 (Ar-*C*), 135.9 (Ar-*C*), 135.2 (Ar-*C*), 130.6 (Ar-*C*), 128.2 (Ar-*C*), 127.9 (Ar-*C*), 127.8 (Ar-*C*), 126.2 (Ar-*C*), 124.4 (Ar-*C*), 122.6 (N*C*H), 122.3 (Ar-*C*), 29.1 (*C*H(CH$_3$)$_2$), , 24.8 (CH(*C*H$_3$)$_2$), 24.0 (CH(*C*H$_3$)$_2$). MS (ESI) $[M+H]^+$ $C_{52}H_{57}CuGeN_3S$: 876.3000, found: 876.2999 (err [ppm] = -2.20). Despite repeated attempts, we were unable to obtain satisfactory yield of this compound for suitable elemental analysis. See figure S16 for the in-situ reaction $^1H$ NMR spectrum and figures S57-58 for full NMR spectra of **8**.

**Synthesis of compound 9, (IPr)CuC(Ph)C(H)GePh$_3$**

In a vial in a glovebox, (IPr)CuGePh$_3$ (30 mg, 0.040 mmol) was dissolved in cold toluene (0.5 mL) and the solution was cooled to -40 °C. Phenyl acetylene (3.5 µL, 0.032 mmol) was then added and the reaction mixture was left at -40 °C for three days. The reaction mixture was then transferred into J Young's NMR tube and volatiles were removed *in vacuo* precipitating a white powder. The powder was redissolved in toluene (0.5 mL) and filtered forming a colourless solution. Single crystals of compound **8** that were suitable for characterisation by SC-XRD were grown from the slow diffusion of hexane, (IPr)CuC(Ph)C(H)GePh$_3$ (11.4 mg, 0.013 mmol, 33 %).

$^1H$ NMR (500 MHz, $C_6D_6$) δ 7.96 – 7.62 (m, 7H, Ar-*H*), 7.22 – 7.19 (m, 3H, Ar-*H*), 7.18 – 7.13 (m, 10H, Ar-*H* and residual benzene), 7.09 – 7.03 (m, 4H, Ar-*H*), 6.99 – 6.97 (m, 5H, Ar-*H*), 6.88 (s, 1H, C*H*GePh$_3$), 6.74 – 6.70 (m, 2H. Ar-*H*), 6.16 (s, 2H, NC*H*), 2.47 (hept, *J* = 7.0 Hz, 4H, C*H*(CH$_3$)$_2$), 1.02 (d, *J* = 6.8 Hz, 12H, CH(C*H*$_3$)$_2$), 0.98 (d, *J* = 6.8 Hz, 12H, CH(C*H*$_3$)$_2$). $^{13}C$ NMR (126 MHz, $C_6D_6$) δ 198.9 (Cu*C*Ph), 184.7 (*C*Cu), 159.6 (Ar-*C*), 145.7 (Ar-*C*), 145.3 (Ar-*C*), 141.5 (Ar-*C*), 135.9 (Ar-*C*), 135.3 (Ar-*C*), 132.5 (*C*HGePh$_3$), 132.3 (Ar-*C*), 130.6 (Ar-*C*), 128.4 (Ar-*C*), 128.2 (Ar-*C*), 128.1 (Ar-*C*), 128.0 (Ar-*C*), 128.0 (Ar-*C*), 125.9 (Ar-*C*), 124.4 (*meta*-*C*), 122.9 (N*C*H), 122.7 (Ar-*H*), 28.9 (*C*H(CH$_3$)$_2$), 24.4 (CH(*C*H$_3$)$_2$), 24.0 (CH(*C*H$_3$)$_2$). MS (ESI) [M+] $C_{53}H_{57}CuGeN_2$: 859.3054, found: 859.3057 (err [ppm] = -0.37). Analysis calculated for $C_{53}H_{57}CuGeN_2$ (MW = 858.23 g/mol): Expected: C, 74.17; H, 6.69; N, 3.26. Found: C, 74.76; H, 7.05; N, 3.58. See figures S59-60 for full NMR spectra of **9**.

**Synthesis of compound 10, (IPr)CuC(H)=C(Ph)GePh$_3$**

In a vial in a glovebox, (IPr)CuGePh$_3$ (30 mg, 0.04 mmol) was dissolved in toluene (0.5 mL) and phenyl acetylene (3.5 µL, 0.03 mmol) was then added forming a colourless solution.

The reaction was left overnight at room temperature and was then transferred into J Young's NMR tube. Volatiles were removed *in vacuo* precipitating a white powder. The powder was redissolved in toluene (0.5 mL) and filtered forming a colourless solution. Single crystals of compound **10** that were suitable for characterisation by SC-XRD were grown from the slow diffusion of pentane into a saturated THF solution at -40 °C. Despite repeated attempts at purification, we were unable to isolate compound **10** from a mixture containing compound **9,** (IPr)CuCCPh, and PhC($GePh_3$)=$CH_2$. See figures S19-S20 for the in-situ reaction $^1$H NMR spectrum.

**Reaction of compound 2 and $Ph_3SnCl$**

In a vial in a glovebox, (IPr)Cu$GePh_3$ (20 mg, 0.026 mmol) was dissolved in $C_6D_6$ (0.5 mL) and $Ph_3SnCl$ (11 mg, 0.03 mmol) was then added forming a colourless solution. The reaction was then transferred into a J Young's NMR tube and was interrogated by $^1$H and $^{119}$Sn NMR spectroscopy. An aliquot of the reaction mixture was dissolved in THF (1 mL) for investigation by mass spectrometry. MS (ESI) expected for $Ph_3GeSnPh_3$ $[M+K]^+$ $C_{36}H_{30}CuGeSnK$: 693.0212, found: 693.0217 (err [ppm] = -1.52).

$^1$H NMR (500 MHz, $C_6D_6$) δ 7.71 – 7.50 (m, 15H, Ar-*H*), 7.11 – 7.06 (m, 15H, Ar-*H*); $^{119}$Sn NMR (186 MHz, $C_6D_6$) δ 160.3 (s, $Ph_3GeSnPh_3$). Resonances correspond to known literature data for $Ph_3GeSnPh_3$.[5] See figures S8-S9 for the in-situ reaction NMR spectra.

**Reaction of compound 2 and $Ph_2PCl$**

In a vial in a glovebox, (IPr)Cu$GePh_3$ (30 mg, 0.040 mmol) was dissolved in $C_6D_6$ (0.5 mL) and $Ph_2PCl$ (7.7 µL, 0.042 mmol) was then added forming a colourless suspension. The reaction was then transferred into a J Young's NMR tube and was interrogated by $^1$H and $^{31}$P NMR spectroscopy. A second equivalent of $Ph_2PCl$ (7.7 µL, 0.042 mmol) was then added and the reaction was interrogated by $^1$H and $^{31}$P NMR spectroscopy. See figures S10-S11 for the in-situ reaction NMR spectra.

**Reaction of compound 2 and carbon dioxide**

In a vial in a glovebox, (IPr)Cu$GePh_3$ (20 mg, 0.026 mmol) was dissolved in $C_6D_6$ (0.5 mL) and the resulting solution was transferred into a J Young's NMR tube. The solution was degassed, and an atmosphere of $CO_2$ was applied. The reaction was then interrogated by $^1$H NMR spectroscopy at room temperature showing no reaction. See figure S12 for the in-situ reaction $^1$H NMR spectrum.

**$^1$H VT-NMR study of the reaction of compound 2 and carbon dioxide**

In a vial in a glovebox, (IPr)Cu$GePh_3$ (30 mg, 0.040 mmol) was dissolved in toluene-$d_8$ (0.5 mL) and the resulting solution was transferred into a J Young's NMR tube. The solution was degassed, and an atmosphere of $CO_2$ was applied. The reaction was then and was interrogated by $^1$H NMR spectroscopy at room temperature. The resulting reaction mixture was monitored at different temperatures (298 K, 273K, and 248 K) within an NMR spectrometer showing no reaction. See figure S13 for the in-situ reaction $^1$H NMR spectra.

**Reaction of compound 2 and di-iso-propyl carbodiimide**

In a vial in a glovebox, (IPr)Cu$GePh_3$ (19.5 mg, 0.026 mmol) was dissolved in $C_6D_6$ (0.5 mL) and di-iso-propyl carbodiimide (4.0 µL, 0.026 mmol) was then added. The reaction was then transferred into a J Young's NMR tube and was interrogated by $^1$H NMR spectroscopy.

The reaction displayed no evidence of a reaction even after heating at 80 °C overnight. See figure S14 for the in-situ reaction $^1$H NMR spectrum.

**Thermolysis of compound 5**

In a vial in a glovebox, (IPr)CuSC(=NtBu)GePh$_3$ (35 mg, 0.040 mmol) was dissolved in $C_6D_6$ (0.5 mL) and the solution was transferred into a J Young's NMR tube. The solution was heated at 120 °C for overnight and was monitored by $^1$H NMR spectroscopy. No evidence of decomposition was observed. See figure S15 for the in-situ reaction $^1$H NMR spectrum.

**Reaction of compound 2 and phenyl isocyanate**

In a vial in a glovebox, (IPr)CuGePh$_3$ (15.5 mg, 0.021 mmol) was dissolved in $C_6D_6$ (0.5 mL) and phenyl isocyanate (2.0 µL, 0.019 mmol) was then added forming a colourless solution. The reaction was then transferred into a J Young's NMR tube and was interrogated by $^1$H NMR spectroscopy. The reaction mixture was left for 2 days at room temperature and a grey material precipitated out during the course of the reaction. An aliquot of the reaction mixture was dissolved in THF (1 mL) for investigation by mass spectrometry. MS (ESI) expected for Ph$_4$Ge [M+K]$^+$ $C_{24}H_{20}GeK$: 420.0437, found: 420.0408 (err [ppm] = -4.00). See figure S16 for the in-situ reaction $^1$H NMR spectrum.

**Thermolysis study of compound 8**

In a vial in a glovebox, (IPr)CuN(Ph)C(O)GePh$_3$ (4.4 mg, 0.005 mmol) was dissolved in $C_6D_6$ (0.5 mL) and was then transferred into a J Young's NMR tube. The resulting solution was heated at 120 °C for 4 days and was monitored by $^1$H NMR spectroscopy. An aliquot of the reaction mixture was dissolved in THF (1 mL) for investigation by mass spectrometry. MS (ESI) expected for [PhNC(O)]$_3$ [M+H]$^+$ $C_{21}H_{16}N_3O_3$: 358.1192, found: 358.1187 (err [ppm] = 0.31). See figure S17 for the in-situ reaction $^1$H NMR spectrum.

**Reaction of compound 1 and phenyl isocyanate**

In a vial in a glovebox, (SIMes)CuGePh$_3$ (12 mg, 0.018 mmol) was dissolved in $C_6D_6$ (0.5 mL) and phenyl isocyanate (2.0 µL, 0.018 mmol) was then added forming a colourless solution. The reaction was then transferred into a J Young's NMR tube and was interrogated by $^1$H NMR spectroscopy. Colourless crystals of (SIMes)CuN(Ph)C(O)N(Ph)C(O)GePh$_3$, crystallised out of solution that were suitably single for characterisation by SC-XRD.

$^1$H NMR (500 MHz, $C_6D_6$) δ 8.09 – 8.03 (m, 6H, Ar-*H*), 7.27 – 7.19 (m, 9H, Ar-*H*), 6.98 – 6.86 (m, 6H, Ar-*H*), 6.72 – 6.59 (m, 4H, Ar-*H*), 6.56 – 6.42 (m, 2H, Ar-*H*), 6.42 – 6.39 (m, 2H, Ar-*H*), 2.84 (s, 4H, NC*H*$_2$), 2.03 (s, 6H, *para*-C*H*$_3$), 1.87 (s, 12H, *ortho*-C*H*$_3$). See figure S18 for the in-situ reaction $^1$H NMR spectrum.

**Reaction of compound 2 and phenyl acetylene at -40 °C**

In a vial in a glovebox, (IPr)CuGePh$_3$ (30 mg, 0.040 mmol) was dissolved in cold toluene-d$_8$ (0.5 mL) and the solution was cooled to -40 °C. Phenyl acetylene (3.5 µL, 0.032 mmol) was then added and the reaction mixture was left at -40 °C for three days. The reaction mixture was then transferred into a J Young's NMR tube and was monitored by $^1$H NMR spectroscopy. For corresponding $^1$H NMR spectrum, see Figure S13. See figure S19 for the in-situ reaction $^1$H NMR spectrum.

**Reaction of compound 2 and of phenyl acetylene at room temperature**

In a vial in a glovebox, (IPr)CuGePh$_3$ (30 mg, 0.040 mmol) was dissolved in toluene-d$_8$ (0.5 mL) and phenyl acetylene (3.5 µL, 0.032 mmol) was added. The reaction was then transferred into a J Young's NMR tube and was monitored by $^1$H NMR spectroscopy. Another equivalent of phenyl acetylene (3.5 µL, 0.032 mmol) was then added and the reaction mixture was interrogated by $^1$H and $^1$H–$^{13}$C HSQC NMR spectroscopy.

An aliquot of the reaction mixture was dissolved in THF (1 mL) for investigation by mass spectrometry. MS (ESI) expected for $PhC(GePh_3)=CH_2$ $[M+H]^+$ $C_{26}H_{23}Ge$: 409.1012 , found: 409.1018 (err [ppm] = 2.75). Selected $^1H$ NMR resonances (400 MHz, Toluene-$d_8$) δ 6.18 (m, 1H, C*H*), 5.65 (m, 1H, C*H*). For corresponding $^1H$–$^{13}C$ HSQC NMR spectrum and full $^1H$ NMR spectral data, see Figures S20-S21.

**Reaction of 10 mol% compound 2 with $Ph_3GeH$ and phenyl acetylene**

In a vial in a glovebox, (IPr)$CuGePh_3$ (5.0 mg, 0.007 mmol) was dissolved in $C_6D_6$ (0.5 mL) and $Ph_3GeH$ (20.0 mg, 0.070 mmol) was added. Phenyl acetylene (7.3 µL, 0.070 mmol) was then added and the reaction mixture was transferred into a J Young's NMR tube. The reaction was heated at 60 °C overnight and was monitored by $^1H$ NMR spectroscopy showing generation of N,N'-bis(2,6-diisopropylphenyl)imidazol-2-ylidene(phenylacetylido)copper(I) and no catalytic turnover. See figure S22 for the in-situ reaction $^1H$ NMR spectrum.

**Reaction of 10 mol% (SIMes)CuOtBu with $Ph_3GeH$ and $Ph_3SnCl$**

In a vial in a glovebox, (SIMes)CuOtBu (5 mg, 0.007 mmol) was dissolved in $C_6D_6$ (0.5 mL) forming a colourless solution. $Ph_3GeH$ (22.6 mg, 0.074 mmol) and $Ph_3SnCl$ (28.6 mg, 0.074 mmol) was then added. The reaction mixture was transferred into a J Young's NMR tube and was monitored by $^1H$ and $^{119}Sn$ NMR spectroscopy overnight at 60 °C showing no reaction. See figures S23-S24 for the in-situ reaction NMR spectra.

**Reaction of (SIMes)CuOtBu with $Ph_3GeH$ and $Bu_3SnOMe$**

In a vial in a glovebox, (SIMes)CuOtBu (30 mg, 0.068 mmol) was dissolved in $C_6D_6$ (0.5 mL) forming a colourless solution. $Ph_3GeH$ (21 mg, 0.068 mmol) and $Bu_3SnOMe$ (19 µL, 0.068 mmol) was then added. The reaction mixture was transferred into a J Young's NMR tube and was interrogated by $^1H$ and $^{119}Sn$ NMR spectroscopy. See figures S25-S26 for the in-situ reaction NMR spectra.

$Ph_3GeSnBu_3$:

1H NMR (500 MHz, $C_6D_6$) δ δ 7.66 – 7.61 (m, 6H, Ar-*H*), 7.22 – 7.18 (m, 6H, Ar-*H*), 7.15 – 7.11 (m, 3H, Ar-*H*), 1.59 – 1.50 (m, 6H, C$H_2$), 1.30 (s, 6H, C$H_2$), 1.20 (m, 15H, C$H_2$ with overlapped tBuOH), 0.81 (t, *J* = 7.3 Hz, 9H, C$H_3$). $^{119}Sn$ NMR spectrum (186 MHz, $C_6D_6$) δ -86.3 (m, $Ph_3GeSnBu_3$).

(SIMes)CuOMe:

1H NMR (500 MHz, $C_6D_6$) δ 6.74 (s, 4H, *meta-H*), 3.45 (s, 3H, CuOC$H_3$), 3.08 (s, 4H, NC$H_2$), 2.14 – 2.10 (m, 18H, *ortho*-C$H_3$ and *para*-C$H_3$).

**General Procedure for the Catalytic Cross Coupling Reactions**

In a vial in a glovebox, $Ph_3GeH$ (20.6 mg, 0.068 mmol) and the corresponding alkoxide (0.068 mmol) were combined in $C_6D_6$ (0.5 mL). (SIMes)CuOtBu (3 mg, 0.007 mmol) was then added, and the reaction mixture was transferred into a J Young's NMR tube. The reaction was monitored by $^1H$ (and $^{119}Sn$ NMR spectroscopy where relevant) until full conversion was reached. Spectroscopic yields were measured using 50 µL of a 1,3,5-trimethoxybenzene stock solution.

**$Ph_3GeSnBu_3$**

The reaction was conducted on a 2.33 mmol scale. Reaction was allowed to proceed at room temperature overnight before volatiles were removed *in vacuo* in order to push the reaction to completion.

$^{1}H$ NMR (500 MHz, $C_6D_6$) δ 7.68 – 7.60 (m, 6H, Ar-*H*), 7.21 – 7.17 (m, 6H, Ar-*H*), 7.15 – 7.12 (m, 3H, Ar-*H*), 1.60 – 1.51 (m, 6H, C*H*$_2$), 1.31 – 1.25 (s, 6H, C*H*$_2$), 1.24 – 1.18 (, 6H, C*H*$_2$), 0.83 – 0.80 (t, *J* = 7.3 Hz, 9H, C*H*$_3$). $^{119}Sn$ NMR (186 MHz, $C_6D_6$) δ -86.3. $^{13}C$ NMR (126 MHz, $C_6D_6$) δ 140.0 (Ar-*C*), 135.7 (Ar-*C*), 128.8 (Ar-*C*), 128.7 (Ar-*C*), 30.43 (Sn*Bu*$_3$), 27.77 (Sn*Bu*$_3$), 13.82 (Sn*Bu*$_3$), 10.22 (Sn*Bu*$_3$). MS (ESI) $[M+Cl]^-$ $C_{30}H_{42}GeSnCl$: Expected: 627.1204, found: 627.1237 (err [ppm] = -1.31). Yield (79 mg, 0.13 mmol, 59 %). See figures S27-S28 for the in-situ reaction NMR spectra and figures S40-S42 for full NMR spectra $Ph_3GeSnBu_3$.

**$Ph_3GeSnPh_3$**

NMR data matched those in literature (see figures S29-S30 for the in-situ reaction NMR spectra). Yield (29.3 mg, 0.045 mmol, 66 %).

**$Bu_2Sn(OMe)_2$**

0.034 mmol used. No conversion was observed even after heating at 60 °C overnight.

**$Ph_2SnO$**

0.034 mmol used. No conversion was observed at room temperature.

**$Ph_3GeOMe$**

5 mol% (SIMes)CuOtBu was used in this reaction. No conversion was observed even after heating to 60 °C overnight.

**$Me_3GeOMe$**

No conversion was observed even after heating at 80 °C overnight.

**$Ph_3SiOMe$**

No conversion was observed even after heating at 100 °C overnight.

**$Ph_2POEt$**

No conversion was observed even after heating at 80 °C overnight.

**General Procedure for the Catalytic Hydrogermylation of Activated Alkenes**

In the glovebox, $HGePh_3$ (20.6 mg, 0.068 mmol) and 1,3,5-trimethoxybenzene (~2 eq) were dissolved in 400 µL $C_6D_6$ (unless otherwise stated) and the corresponding alkene (0.068 mmol) was then added. The solution was transferred to a J. Young's NMR tube and a $^{1}H$ NMR spectroscopy experiment (using a delay of 25 seconds) was performed to obtain initial ratios of $HGePh_3$, 1,3,5-trimethoxybenzene, and the unsaturated substrate. The sample was then returned to the glovebox where 10 mol% of (SIMes)CuOtBu (100 µL of a stock solution) was added. The reaction was then heated at 60 °C overnight unless overwise stated. $^{1}H$ NMR spectroscopy was then taken (using a delay of 25 seconds) which provided a spectroscopic conversion and yield of the reaction.

**$Ph_3GeCH_2CH_2C(O)OCH_3$, 11a**

Compound was contaminated with *ca.* 15 mol% **11c** caused by competing transesterification of **11a**.

$^{1}H$ NMR (500 MHz, $C_6D_6$) δ 7.47 – 7.44 (m, 6H, Ar-*H*), 7.19 – 7.11 (m, 18H, Ar-*H*, and residual $C_6H_6$ solvent), 3.24 (s, 3H, OC*H*$_3$), 2.45 – 2.42 (m, 2H, C*H*$_2$), 1.85 – 1.80 (m, 2H, C*H*$_2$).

$^{13}C$ NMR (126 MHz, $C_6D_6$) δ 174.0 (*C*O), 135.6 (Ar-*C*), 135.3 (Ar-*C*), 129.3 (Ar-*C*), 128.6 (Ar-*C*), 51.0 (O*C*$H_3$), 29.9 (*C*$H_2$), 9.3(*C*$H_2$). MS (ESI) expected for $C_{22}H_{22}GeO_2Na$ [M+Na]$^+$: 415.0729, found: 415.0725 (err [ppm] = 0.7). Conversion, >99 %; Yield, 91 %. See figures S61-62 for NMR spectra of **11a**.

**$Ph_3GeCH_2CH_2C(O)OCH_2CH_2$, 11b**

Compound was contaminated with *ca*. 11 mol% **11c** caused by competing transesterification of **11b**.

$^{1}H$ NMR (400 MHz, $C_6D_6$) δ 7.49 – 7.45 (m, 6H, Ar-*H*), 7.19 – 7.10 (m, 18H, Ar-*H* and residual $C_6H_6$ solvent), 3.86 (q, 2H, *J* = 7.1 Hz, OC*H*$_2$), 2.50 – 2.46 (m, 2H, C*H*$_2$), 1.89 – 1.85 (m, 2H, C*H*$_2$), 0.89 (t, *J* = 7.1 Hz, 3H, C*H*$_3$). $^{13}C$ NMR (101 MHz, $C_6D_6$) δ 173.7 (*C*O), 136.8 (Ar-*C*), 135.3 (Ar-*C*), 129.3 (Ar-*C*), 128.6 (Ar-*C*), 60.2 (O*C*$H_2$), 30.2 (*C*$H_2$), 14.2 (C*H*$_3$), 9.3 (*C*$H_2$). MS (ESI) expected for $Ph_3GeCH_2CH_2C(O)OCH_3$ [M+Na]$^+$ $C_{23}H_{24}GeO_2Na$: 429.0886, found: 429.0910 (err [ppm] = -5.8). Conversion, >99 %; Yield, 77 %. See figures S63-64 for NMR spectra of **11b**.

**$Ph_3GeCH_2CH_2C(O)OC(CH_3)_3$, 11c**

$^{1}H$ NMR (500 MHz, $C_6D_6$) δ 7.51 – 7.48 (m, 6H), 7.19 – 7.12 (m, 10H, Ar-*H* and residual $C_6H_6$ solvent), 2.54 – 2.51 (m, 2H, C*H*$_2$), 1.94 – 1.90 (m, 2H, C*H*$_2$), 1.33 (s, 9H, OC(C*H*$_3$)$_3$). $^{13}C$ NMR (126 MHz, $C_6D_6$) δ 173.3 (*C*O), 136.9 (Ar-*C*), 135.4 (Ar-*C*), 129.2 (Ar-*C*), 128.6 (Ar-*C*), 79.7 (O*C*(CH$_3$)$_3$), 31.3 (*C*$H_2$), 28.1 (OC(*C*H$_3$)$_3$), 9.3 (C*H*$_2$). MS (ESI) expected for $Ph_3GeCH_2CH_2C(O)OC(CH_3)_3$ [M+Na]$^+$ $C_{25}H_{28}GeO_2Na$: 457.1199, found: 457.1192 (err [ppm] = 1.5). Conversion, >99 %; Yield, >99 %. See figures S65-66 for NMR spectra of **11c**.

**$Ph_3GeCH_2(CH_3)CHC(O)OCH_3$, 11d**

Reaction was heated at 100 °C for two days when full conversion was reached. Compound was contaminated with *ca*. 8 mol% Ph3GeCH$_2$(CH$_3$)CHC(O)OC(CH$_3$)$_3$ from competing transesterification of **11d**. MS (ESI) expected for $Ph_3GeCH_2(CH_3)CHC(O)OC(CH_3)_3$ [M+Na]$^+$ $C_{26}H_{30}GeO_2Na$: 471.1355, found: 471.1353 (err [ppm] = -0.16).

$^{1}H$ NMR (500 MHz, $C_6D_6$) δ 7.56 – 7.52 (m, 6H, Ar-*H*), 7.17 – 7.13 (m, 12H, Ar-*H* and residual $C_6H_6$ solvent), 3.06 (s, 3H, OC*H*$_3$), 2.80 – 2.73 (m, 1H, C*H*), 2.12 – 2.02 (m, 1H, C*H*$_2$), 1.61 – 1.54 (m, 1H, C*H*$_2$), 1.13 – 1.11 (m, 3H, CHC*H*$_3$). $^{13}C$ NMR (126 MHz, $C_6D_6$) δ 176.6 (*C*O), 137.2 (Ar-*C*), 135.5 (Ar-*C*), 129.2 (Ar-*C*), 128.6 (Ar-*C*), 50.9 (O*C*H$_3$), 36.6 (*C*H), 21.1 (CH*C*H$_3$), 19.4 (*C*H$_2$). MS (ESI) expected for $Ph_3GeCH_2(CH_3)CHC(O)OCH_3$ [M+Na]$^+$ $C_{23}H_{24}GeO_2Na$: 429.0886, found: 429.0899 (err [ppm] = -3.4). Conversion, >99 %; Yield, 82 %. See figures S67-68 for NMR spectra of **11d**.

**$Ph_3Ge(H_3C)CHCH_2C(O)OCH_3$, 11e**

No conversion was observed even after heating to 100 °C overnight.

**$Ph_3GeCH_2CH_2C(O)CH_3$, 11f**

Complete consumption of $CH_2CH_2C(O)CH_3$ was observed after heating to 80 °C for three days resulting in an intractable mixture. No consumption of $Ph_3GeH$ was observed (see figure S31).

**$Ph_3GeCH_2CH_2C(O)NH_2$, 11g**

Reaction was conducted in THF-d$_8$ and was heated at 100 °C for seven days when full conversion was reached. $^{1}H$ NMR (500 MHz, THF) δ 7.52 – 7.46 (m, 6H,m Ar-*H*), 7.36 – 7.29 (m, 9H, Ar-*H*), 6.42 (s, 1H, N*H*), 5.98 (s, 1H, N-*H*), 2.27 – 2.23 (m, 2H, C*H*$_2$), 1.84 – 1.79 (m, 2H, C*H*$_2$).

$^{13}C$ NMR (126 MHz, THF-$d_8$) δ 175.2 (*C*O), 138.0 (Ar-*C*), 135.9 (Ar-*C*), 129.8 (Ar-*C*), 129.1 (Ar-*C*), 31.6 (*C*$H_2$), 10.0 (*C*$H_2$). MS (ESI) expected for $Ph_3GeCH_2CH_2C(O)NH_2$ $[M+Na]^+$ $C_{21}H_{21}GeNONa$: 400.0733, found: 400.0730 (err [ppm] = -0.2). Conversion, 99 %; Yield, 77 %. See figures S69-70 for NMR spectra of **11g**.

**$Ph_3GeCH_2CH_2C(O)NMe_2$, 11h**

$^1H$ NMR (500 MHz, $C_6D_6$) δ 7.59 – 7.56 (m, 6H, Ar-*H*), 7.21 – 7.13 (m, 11H, Ar-*H* and residual $C_6H_6$ solvent), 2.56 (s, 3H, NC*H*$_3$), 2.27 – 2.24 (m, 2H, C*H*$_2$), 2.04 – 2.00 (m, 2H, C*H*$_2$), 1.96 (s, 3H, NC*H*$_3$). $^{13}C$ NMR (126 MHz, $C_6D_6$) δ 172.1 (*C*O), 137.5 (Ar-*C*), 135.5 (Ar-*C*), 129.2 (Ar-*C*), 128.6 (Ar-*C*), 35.9 (N*C*$H_3$), 35.0 (N*C*$H_3$), 28.9 (*C*$H_2$), 9.3 (*C*$H_2$). MS (ESI) expected for $Ph_3GeCH_2CH_2C(O)NMe_2$ $[M+Na]^+$ $C_{23}H_{25}GeNONa$: 428.1046, found: 428.1045 (err [ppm] = 0.33). Conversion, >99 %; Yield, 97 %. See figures S71-72 for NMR spectra of **11h**.

**$Ph_3GeCH_2CH_2CN$, 11i**

Minimal conversion of $CH_2CH_2CN$ was observed even after heating to 100 °C for three days (see figure S32).

**4-($Ph_3GeCH_2CH_2$)-$C_5H_5N$, 11j**

Complete consumption of $CH_2CH_2$(4-$C_5H_5N$) was observed after heating to 60 °C for three days resulting in an intractable mixture (see figure S33).

**2-($Ph_3GeCH_2CH_2$)-$C_5H_5N$, 11k**

Reaction was heated at 40 °C overnight when full conversion was reached.

$^1H$ NMR (500 MHz, $C_6D_6$) δ 8.50 – 8.48 (m, 1H, Ar-*H*), 7.57 – 7.54 (m, 6H, Ar-*H*), 7.19 – 7.14 (m, 13H, Ar-*H* and residual $C_6H_6$ solvent), 7.00 – 6.96 (m, 1H, Ar-*H*), 6.61 – 6.56 (m, 2H, Ar-*H*), 3.09 – 3.05 (m, C*H*$_2$), 2.10 – 2.07 (m, 2H, C*H*$_2$). $^{13}C$ NMR (126 MHz, $C_6D_6$) δ 163.9 (*C*O), 149.7 (Ar-*C*), 137.5 (Ar-*C*), 135.7 (Ar-*C*), 135.4 (Ar-*C*), 129.2 (Ar-*C*), 128.6 (Ar-*C*), 128.4 (Ar-*C*), 122.2 (Ar-*C*), 120.9 (Ar-*C*), 34.1 (*C*$H_2$), 14.4 (C*H*$_2$). MS (ESI) expected for $Ph_3GeCH_2CH_2$(2-$C_5H_5N$) $[M+Na]^+$ $C_{25}H_{23}GeNNa$: 434.0940, found: 434.0955 (err [ppm] = -3.6). Conversion, >99 %; Yield, 97 %. See figures S73-74 for NMR spectra of **11k**.

**$Ph_3GeCH_2CH_2Ph$, 11l**

No conversion was observed even after heating to 100 °C overnight.

## X-ray crystallography

Data for compounds **2**, **3**-**8**, and (SIMes)CuN(Ph)C(O)N(Ph)C(O)GePh$_3$ were obtained using an Agilent SuperNova instrument and a Cu−Kα source while those for compounds **1** and **9**-**10** were obtained garnered with Mo−Kα radiation on an Agilent Xcalibur diffractometer. All experiments were conducted at 150 K, solved using SHELXT[2] and refined using SHELXL[2] via the Olex2[3] interface. Multiscan absorption corrections were employed throughout, *via* the CrysAlisPro (Rigaku) diffractometer software. While the majority of refinements were straightforward there are some points of particular note that merit special mention herein.

In the structure of compound **2**, it was noted that there are two molecules of the copper complex in the asymmetric unit. C11 and C12 were treated for 55:45 disorder with the inclusion of minimal distance and ADP restraints.

For compound **4**, premium sample quality and a routine data collection readily afforded the solution of the structure. The conundrum lay, therefore, with the maximum residual electron density peak located at a chemically insignificant distance (0.84 Å) from the germanium centre. Initial strategies were to consider some minor disorder with the (6-Dipp)HBr adduct (a synthetic precursor) or some by−product. Spectroscopically, the dissolved crystals did not evidence either scenario.
Scrutiny of the frames indicated the presence of a second diffracting component that contributed so weakly to the pattern that it was only evident at low Bragg angles. Nonetheless, a twin integration was performed (followed by refinement) until convergence. However, this approach offered no significant change in the intensity or position of the residual electron−density maximum which was unsurprising given the paucity and intensity of the data from the second component. As a test, the data were integrated with triclinic symmetry and this illustrated that the residual electron−density maximum observed herein was also present for all four molecules in the *P*1 asymmetric unit. There was no evidence for any twinning.

The asymmetric unit in compound **6** was seen to contain two independent molecules, with S4 (in the complex based on Cu2) necessitating treatment for 60:40 disorder. Distance and ADP restraints were employed, on merit, in this disordered region to assist convergence. The largest residual electron density peak lies at a chemically insignificant distance (0.90 Å) from Cu1. Attempts to model same as a being disordered with Cu1 indicated that any putative disorder was in the region of 4%. As such, a two−component treatment of this copper centre was abandoned as it would have been artificial rather than credible.
The structural solution of compound **7** proved unexpectedly problematic beyond the fact that C23 and C24 were treated for 50:50 disorder. The primary issue relates to smearing of the electron density in the region of the amide ligand, which may be indicative of some disorder or slight rockling/pendulous behaviour of this ligand. This is very evident in the bond precision observed for this region of the molecule wherein the phenyl ring based on C35 was refined as a rigid hexagon. The C34−C35 bond distance is long relative to what one might expect for the $sp^2$-hybridised C34 and imposition of a bond−distance restraint had little impact on same. Efforts to model any disorder of the amide were not credible (< 10% occupancy of any putative second component) and, therefore, abandoned. The raw data were textbook in quality and there was no evidence of twinning. Integration in lower symmetries did nothing to improve the movement in the region of the amide, whatsoever. As such, incorrect space group assignment could be excluded. Ultimately, while this structure affords a chemical characterisation of compound **7**, no claims will be made with respect to the metrics withing the amide moiety for the aforementioned reasons.

There were two molecules present in the asymmetric unit of compound **8**, and, in the structure of compound **9**, the hydrogen atoms attached to C29 was located, and refined without restraints.

Refinement was straightforward in the structure of compound **10** with the only notable quirk being the residual electron density maximum. The location of said peak is chemically insignificant, but the location of same may point to some crystallographic disorder. Efforts to model this suggested that it would be in the region of 3% and, as such, disorder modelling was abandoned.

Finally, the asymmetric unit in (SIMes)CuN(Ph)C(O)N(Ph)C(O)GePh$_3$ comprises one molecule of the copper complex and one molecule of deuterated benzene.

Crystallographic data for all compounds have been deposited with the Cambridge Crystallographic Data Centre as the following supplementary CCDC publications: 2221285−2221292 for compound **1** (2221285), compound **2** (2221286), compound **3** (2221288), compound **4**(2221287), compound **5** (2221289), compound **6** (2221290), compound **7** (2221291), compound **8** (2249130), compound **9** (2221292), compound **10** (2249131) and (SIMes)CuN(Ph)C(O)N(Ph)C(O)GePh$_3$ (2249132). Copies of these data can be obtained free of charge on application to CCDC, 12 Union Road, Cambridge CB2 1EZ, UK [fax(+44) 1223 336033, e−mail: deposit@ccdc.cam.ac.uk.

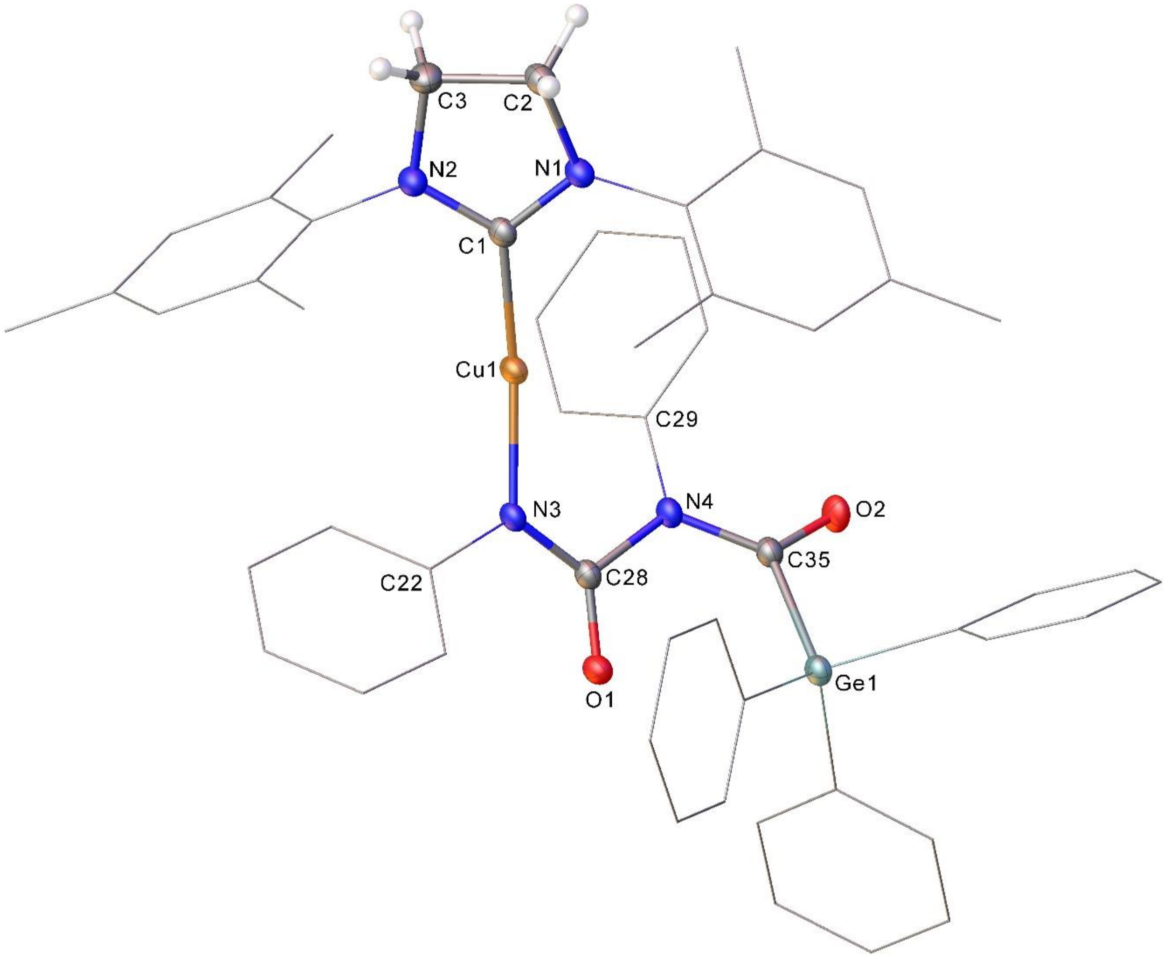


**Figure S1.** Molecular structure of (SIMes)CuN(Ph)C(O)N(Ph)C(O)GePh$_3$. Hydrogen atoms have been omitted for clarity. The phenyl and carbene substituents are represented in wireframe view, for clarity. Ellipsoids are represented at 30% probability. Selected bond length (Å) and angle (°) data: C1-Cu1, 1.891(3); Cu1-N3, 1.903(2); N3-C28, 1.332(4); C28-O1, 1.229(4); C28-N4, 1.458(3); N4-C35, 1.384(4); C35-O2, 1.224(4); C35-Ge1,2.002(3) ; C1-Cu1-N3, 165.69(11); N4-C35-Ge1, 123.5(2).

**Table S1.** Crystal data and structure refinement details

| **Identification code** | **1** | **2** | **3** | **4** |
|---|---|---|---|---|
| **Empirical formula** | $C_{39}H_{41}CuGeN_2$ | $C_{90}H_{102}Cu_2Ge_2N_4$ | $C_{40}H_{43}CuGeN_2$ | $C_{46}H_{55}CuGeN_2$ |
| **Formula weight** | 673.87 | 1512.01 | 687.89 | 772.05 |
| **Crystal system** | triclinic | triclinic | monoclinic | orthorhombic |
| **Space group** | $P$-1 | $P$-1 | $P2_1/n$ | $P2_12_12_1$ |
| ***a*/ Å** | 10.6500(4) | 11.0264(1) | 8.9616(1) | 12.0126(1) |
| ***b*/ Å** | 11.2963(4) | 19.2655(3) | 19.7494(1) | 17.1438(1) |
| ***c*/ Å** | 15.0976(5) | 20.3965(3) | 20.0400(2) | 19.7933(1) |
| ***α*/ °** | 111.368(3) | 86.283(1) | 90 | 90 |
| ***β*/ °** | 93.137(3) | 77.281(1) | 100.990(1) | 90 |
| ***γ*/ °** | 92.330(3) | 73.472(1) | 90 | 90 |
| **V/ $Å^3$** | 1685.35(11) | 4051.84(10) | 3481.76(6) | 4076.26(5) |
| ***Z*** | 2 | 2 | 4 | 4 |
| **$\rho_{calc}$/ g $cm^{-3}$** | 1.328 | 1.239 | 1.312 | 1.258 |
| **$\mu$/ $mm^{-1}$** | 1.551 | 1.785 | 2.025 | 1.784 |
| ***F*(000)** | 700.0 | 1584.0 | 1432.0 | 1624.0 |
| **Crystal size/ $mm^3$** | 0.423 × 0.267 × 0.166 | 0.255 × 0.213 × 0.114 | 0.248 × 0.167 × 0.073 | 0.212 × 0.157 × 0.09 |
| **Radiation** | Mo Kα (λ = 0.71073) | Cu Kα (λ = 1.54184) | Cu Kα (λ = 1.54184) | Cu Kα (λ = 1.54184) |
| **2θ range for data collection/°** | 5.812 to 60.584 | 8.558 to 146.696 | 8.956 to 145.086 | 6.822 to 145.22 |
| **Index ranges** | −14 ≤ h ≤ 15,<br>−15 ≤ k ≤ 15,<br>−19 ≤ l ≤ 21 | −9 ≤ h ≤ 13,<br>−23 ≤ k ≤ 23,<br>−25 ≤ l ≤ 24 | −11 ≤ h ≤ 11,<br>−24 ≤ k ≤ 16,<br>−24 ≤ l ≤ 24 | −14 ≤ h ≤ 14,<br>−21 ≤ k ≤ 21,<br>−24 ≤ l ≤ 21 |
| **Reflections collected** | 15653 | 48122 | 46335 | 45377 |
| **Independent reflections, $R_{int}$** | 8491, 0.0241 | 16059, 0.0274 | 6896, 0.0212 | 8021, 0.0368 |
| **Data/restraints/parameters** | 8491/0/394 | 16059/14/919 | 6896/0/403 | 8021/0/459 |
| **Goodness–of–fit on $F^2$** | 1.040 | 1.065 | 1.081 | 1.121 |
| **Final *R*1, *wR*2 [*I*>=2σ(*I*)]** | 0.0328, 0.0795 | 0.0348, 0.0924 | 0.0270, 0.0722 | 0.0564, 0.1432 |
| **Final *R*1, *wR*2 [all data]** | 0.0437, 0.0853 | 0.0372, 0.0943 | 0.0284, 0.0733 | 0.0566, 0.1433 |
| **Largest diff. peak/hole/ e $Å^{-3}$** | 0.78/−0.41 | 0.78/−0.44 | 0.45/−0.23 | 2.86/−0.77 |
| **Flack Parameter** | – | – | – | −0.01(2) |
| **CCDC number** | 2221285 | 2221286 | 2221288 | 2221287 |

**Table S2.** Crystal data and structure refinement details

| **Identification code** | **5** | **6** | **7** | **8** |
|---|---|---|---|---|
| **Empirical formula** | $C_{50}H_{60}CuGeN_3S$ | $C_{92}H_{102}Cu_2Ge_2N_4S_4$ | $C_{40}H_{46}CuN_3O$ | $C_{104}H_{112}Cu_2Ge_2N_6O_2$ |
| **Formula weight** | 871.20 | 1664.27 | 648.34 | 1750.25 |
| **Crystal system** | monoclinic | triclinic | orthorhombic | monoclinic |
| **Space group** | $P2_1/c$ | *P*–1 | $Pca2_1$ | *P*2/*c* |
| ***a*/ Å** | 10.22373(4) | 13.1905(5) | 16.2264(3) | 13.3558(2) |
| ***b*/ Å** | 20.07164(9) | 18.6418(7) | 10.8168(3) | 18.1417(3) |
| ***c*/ Å** | 22.27774(11) | 20.1369(10) | 20.4792(7) | 38.4583(7) |
| ***α*/ °** | 90 | 66.024(4) | 90 | 90 |
| ***β*/ °** | 98.8634(4) | 81.401(3) | 90 | 99.0988(15) |
| ***γ*/ °** | 90 | 69.363(4) | 90 | 90 |
| ***V*/ Å$^3$** | 4516.96(4) | 4233.9(3) | 3594.46(17) | 9201.1(3) |
| ***Z*** | 4 | 2 | 4 | 4 |
| **$\rho_{calc}$/ g cm$^{-3}$** | 1.281 | 1.305 | 1.198 | 1.263 |
| **$\mu$/ mm$^{-1}$** | 2.097 | 2.656 | 1.104 | 1.669 |
| ***F*(000)** | 1832.0 | 1736.0 | 1376.0 | 3664.0 |
| **Crystal size/ mm$^3$** | 0.212 × 0.186 × 0.114 | 0.098 × 0.056 × 0.044 | 0.098 × 0.05 × 0.018 | 0.099 × 0.082 × 0.016 |
| **Radiation** | Cu Kα (λ = 1.54184) | Cu Kα (λ = 1.54184) | Cu Kα (λ = 1.54184) | Cu Kα (λ = 1.54184) |
| **2θ range for data collection/°** | 8.034 to 145.16 | 7.162 to 145.254 | 8.174 to 145.636 | 7.532 to 148.838 |
| **Index ranges** | −12 ≤ h ≤ 10, −24 ≤ k ≤ 24, −26 ≤ l ≤ 27 | −13 ≤ h ≤ 16, −18 ≤ k ≤ 22, −24 ≤ l ≤ 24 | −12 ≤ h ≤ 20, −13 ≤ k ≤ 12, −25 ≤ l ≤ 23 | −16 ≤ h ≤ 13, −20 ≤ k ≤ 22, −30 ≤ l ≤ 47 |
| **Reflections collected** | 64057 | 31199 | 23418 | 41435 |
| **Independent reflections, $R_{int}$** | 8941, 0.0194 | 16439, 0.0251 | 6888, 0.0370 | 17933, 0.0395 |
| **Data/restraints/parameters** | 8941/0/516 | 16439/7/962 | 6888/91/421 | 17933/0/1061 |
| **Goodness–of–fit on F$^2$** | 1.056 | 1.015 | 1.045 | 1.014 |
| **Final *R*1, *wR*2 [*I*>=2σ(*I*)]** | 0.0288, 0.0738 | 0.0383, 0.0923 | 0.0522, 0.1377 | 0.0476, 0.1123 |
| **Final *R*1, *wR*2 [all data]** | 0.0294, 0.0743 | 0.0469, 0.0978 | 0.0615, 0.1468 | 0.0748, 0.1257 |
| **Largest diff. peak/hole/ e Å$^{-3}$** | 0.48/−0.33 | 1.60/−0.84 | 0.70/−0.36 | 0.42/−0.45 |
| **Flack Parameter** | – | – | 0.01(2) | – |
| **CCDC number** | 2221289 | 2221290 | 2221291 | 2249130 |

**Table S3.** Crystal data and structure refinement details

| Identification code | 9 | 10 | (SIMes)CuN(Ph)C(O)N(Ph)C(O)GePh$_3$ |
|---|---|---|---|
| **Empirical formula** | $C_{53}H_{57}CuGeN_2$ | $C_{53}H_{57}CuGeN_2$ | $C_{59}H_{57}CuGeN_4O_2$ |
| **Formula weight** | 858.13 | 858.13 | 990.21 |
| **Crystal system** | monoclinic | monoclinic | monoclinic |
| **Space group** | $P2_1/n$ | $P2_1/n$ | $P2_1/n$ |
| ***a*/ Å** | 14.9501(3) | 12.3095(2) | 11.4816(1) |
| ***b*/ Å** | 19.7103(3) | 19.8911(3) | 30.4535(2) |
| ***c*/ Å** | 16.1176(3) | 18.5854(4) | 14.2528(1) |
| ***α*/ °** | 90 | 90 | 90 |
| ***β*/ °** | 105.269(2) | 91.1959(18) | 94.8925(7) |
| ***γ*/ °** | 90 | 90 | 90 |
| ***V*/ Å$^3$** | 4581.73(15) | 4549.62(15) | 4965.40(6) |
| ***Z*** | 4 | 4 | 4 |
| **$\rho_{calc}$/ g cm$^{-3}$** | 1.244 | 1.253 | 1.325 |
| **$\mu$/ mm$^{-1}$** | 1.156 | 1.164 | 1.635 |
| ***F*(000)** | 1800.0 | 1800.0 | 2064.0 |
| **Crystal size/ mm$^3$** | 0.429 × 0.274 × 0.261 | 0.302 × 0.288 × 0.129 | 0.241 × 0.087 × 0.053 |
| **Radiation** | Mo Kα (λ = 0.71073) | Mo Kα (λ = 0.71073) | Cu Kα (λ = 1.54184) |
| **2θ range for data collection/°** | 5.938 to 60.668 | 5.916 to 58.26 | 6.868 to 147.15 |
| **Index ranges** | −21 ≤ h ≤ 21, −25 ≤ k ≤ 26, −21 ≤ l ≤ 22 | −16 ≤ h ≤ 16, −25 ≤ k ≤ 26, −25 ≤ l ≤ 22 | −14 ≤ h ≤ 11, −37 ≤ k ≤ 37, −17 ≤ l ≤ 17 |
| **Reflections collected** | 41691 | 44765 | 78090 |
| **Independent reflections, $R_{int}$** | 12097, 0.0261 | 11791, 0.0440 | 9988, 0.0490 |
| **Data/restraints/parameters** | 12097/0/526 | 11791/0/526 | 9988/0/610 |
| **Goodness–of–fit on F$^2$** | 1.023 | 1.099 | 1.163 |
| **Final *R*1, *wR*2 [*I*>=2σ(*I*)]** | 0.0323, 0.0711 | 0.0531, 0.1142 | 0.0512, 0.1262 |
| **Final *R*1, *wR*2 [all data]** | 0.0510, 0.0798 | 0.0950, 0.1290 | 0.0545, 0.1281 |
| **Largest diff. peak/hole/ e Å$^{-3}$** | 0.43/−0.36 | 1.33/−0.39 | 1.17/−0.47 |
| **Flack Parameter** | – | – | – |
| **CCDC number** | 2221292 | 2249131 | 2249132 |

## *In situ* reaction monitoring by NMR spectroscopy

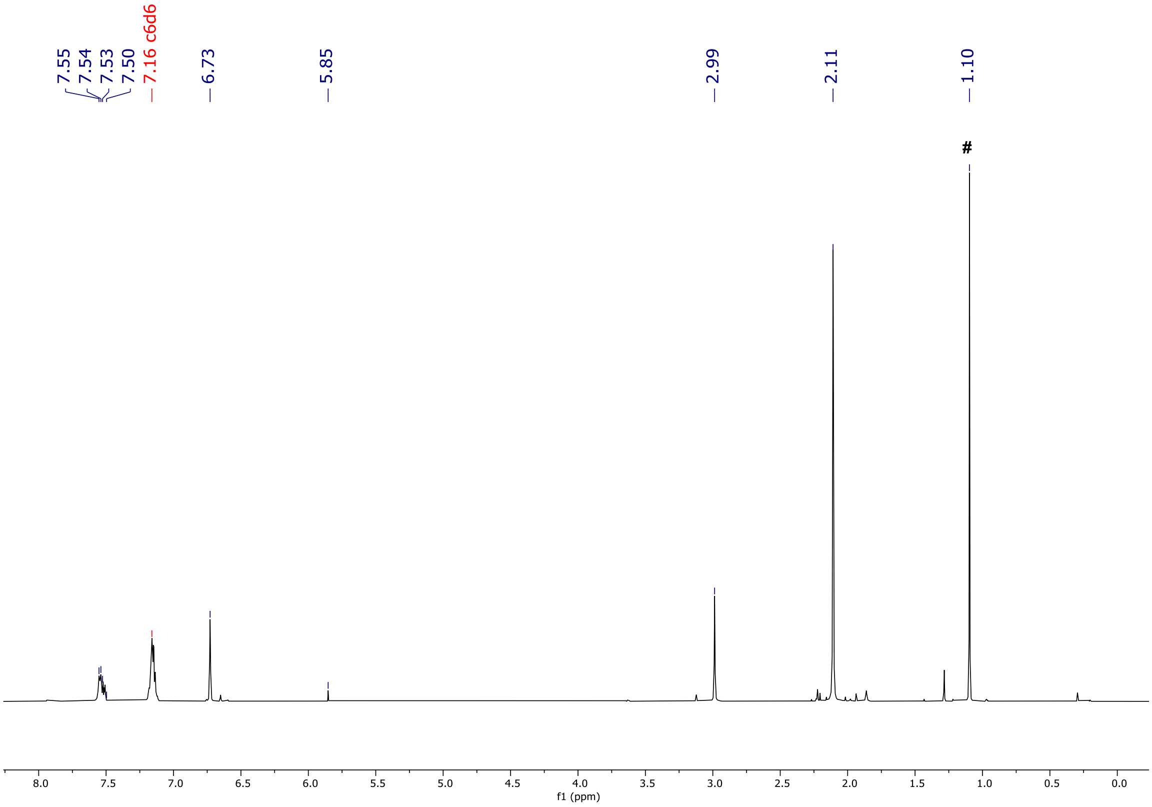


**Figure S2**. The ¹H NMR spectrum (500 MHz, $C_6D_6$) of the reaction of (SIMes)CuOtBu and $Ph_3GeH$ after heating for 3 days at 40 °C. # = HOtBu.

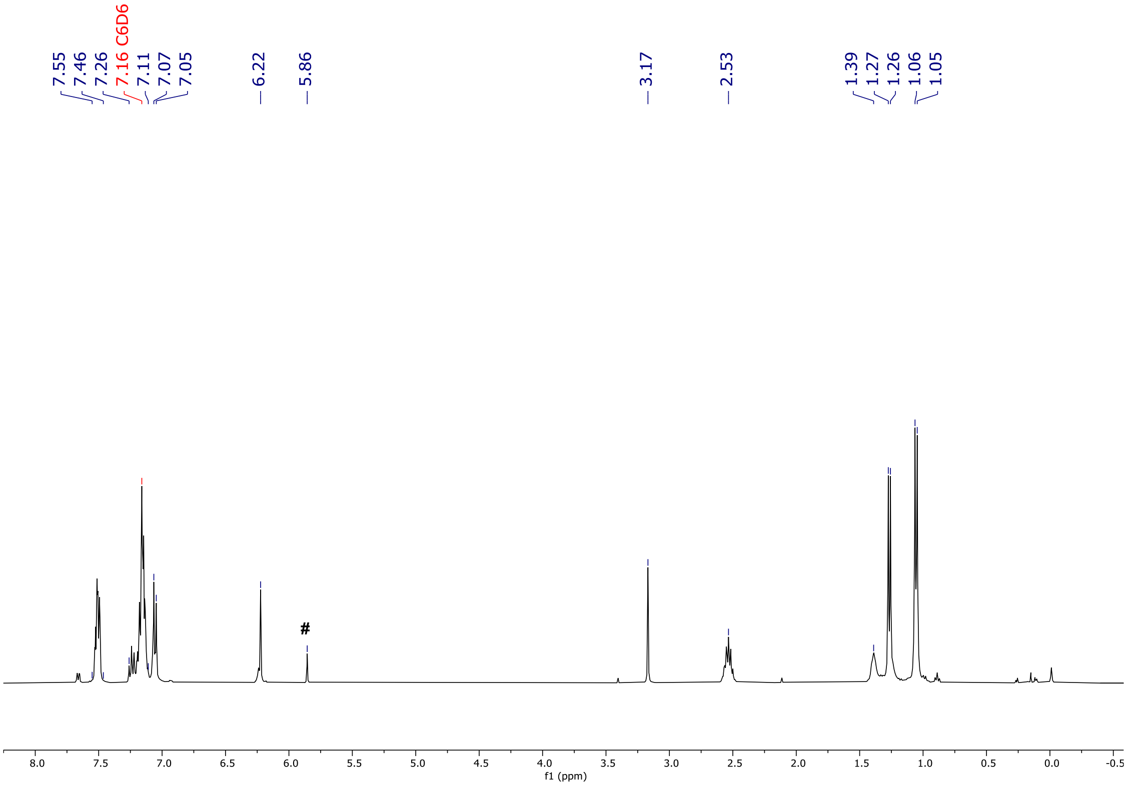


**Figure S3**. The ¹H NMR spectrum (500 MHz, $C_6D_6$) of the reaction of (IPr)CuOMe and $Ph_3GeH$ after 3 days at room temperature. # = residual $Ph_3GeH$.

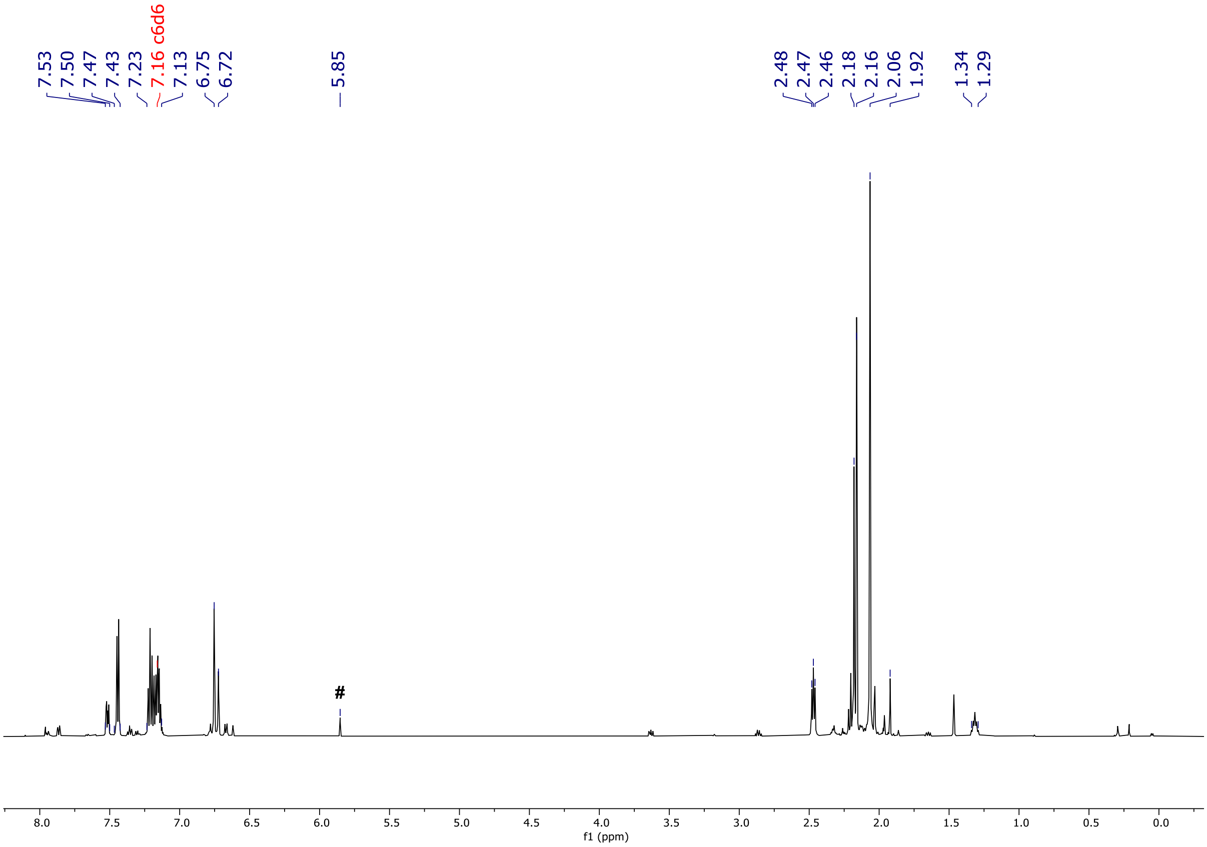


**Figure S4**. The $^{1}$H NMR spectrum (500 MHz, $C_6D_6$) of the reaction of (6-Mes)CuMes and $Ph_3GeH$ after heating for 2 days at 40 °C. # = residual $Ph_3GeH$.

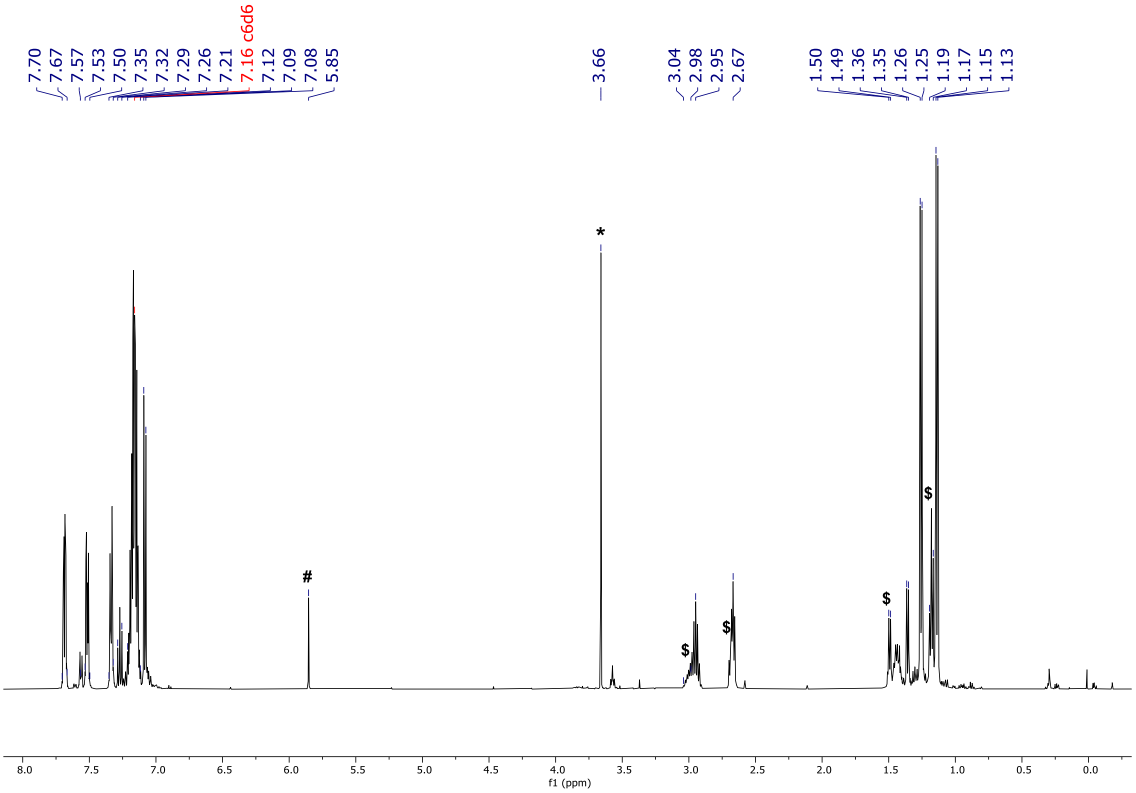


**Figure S5**. The $^{1}$H NMR spectrum (500 MHz, $C_6D_6$) of the reaction of (6-Dipp)CuOMe and $Ph_3GeH$ after heating for 3 days at 60 °C. # = residual $Ph_3GeH$; * = methanol; $ = residual (6-Dipp)CuOMe.

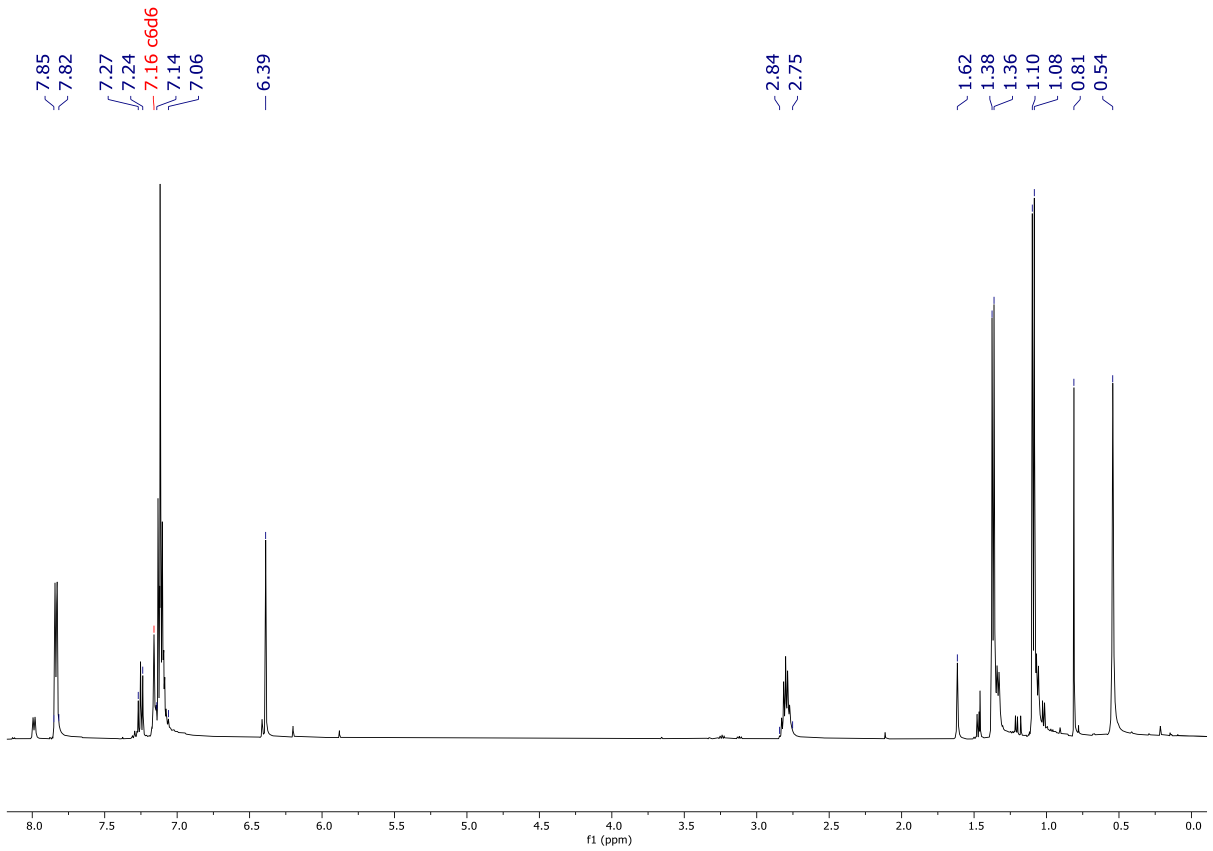


**Figure S6**. The $^1$H NMR spectrum (500 MHz, $C_6D_6$) of the reaction of (IPr)CuGePh$_3$ and tBuNCS after heating for 10 days at 40 °C.

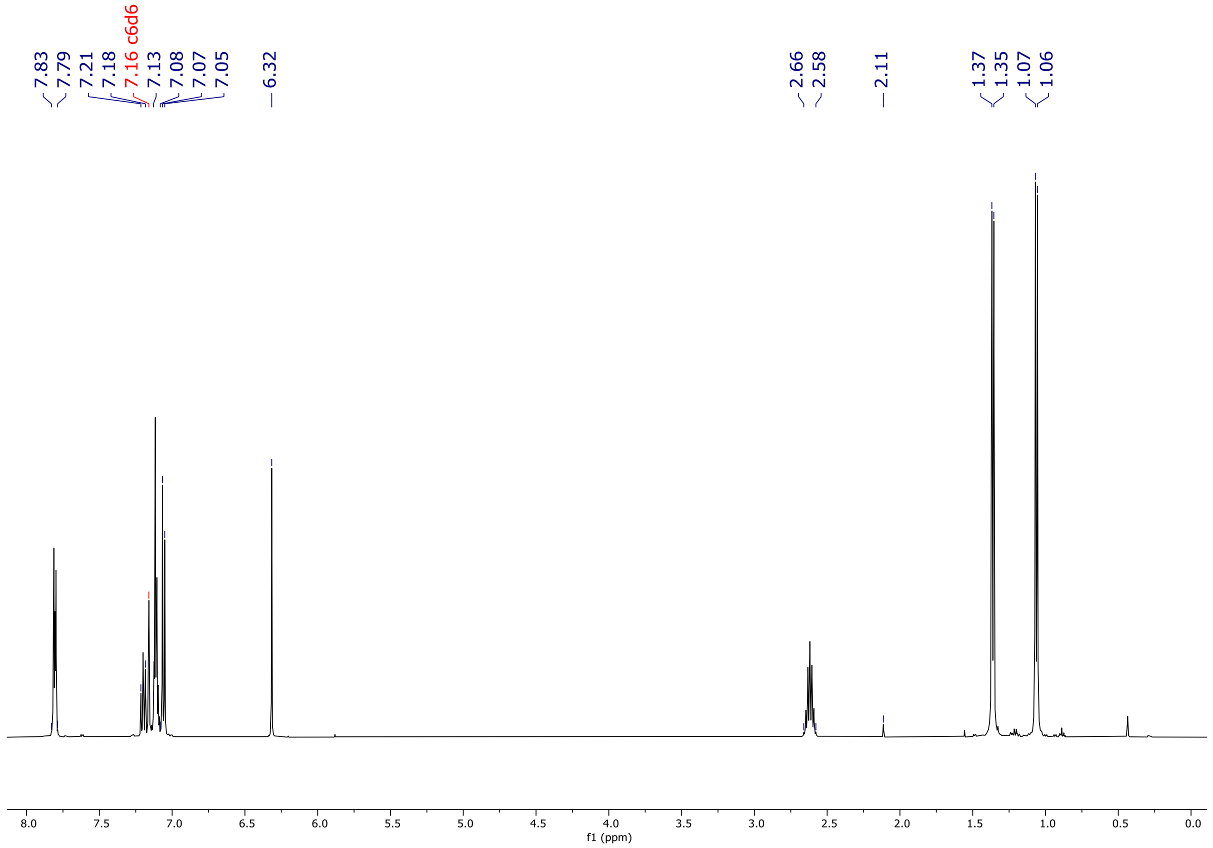


**Figure S7**. The $^1$H NMR spectrum (500 MHz, $C_6D_6$) of the reaction of (IPr)CuGePh$_3$ and $CS_2$ at room temperature.

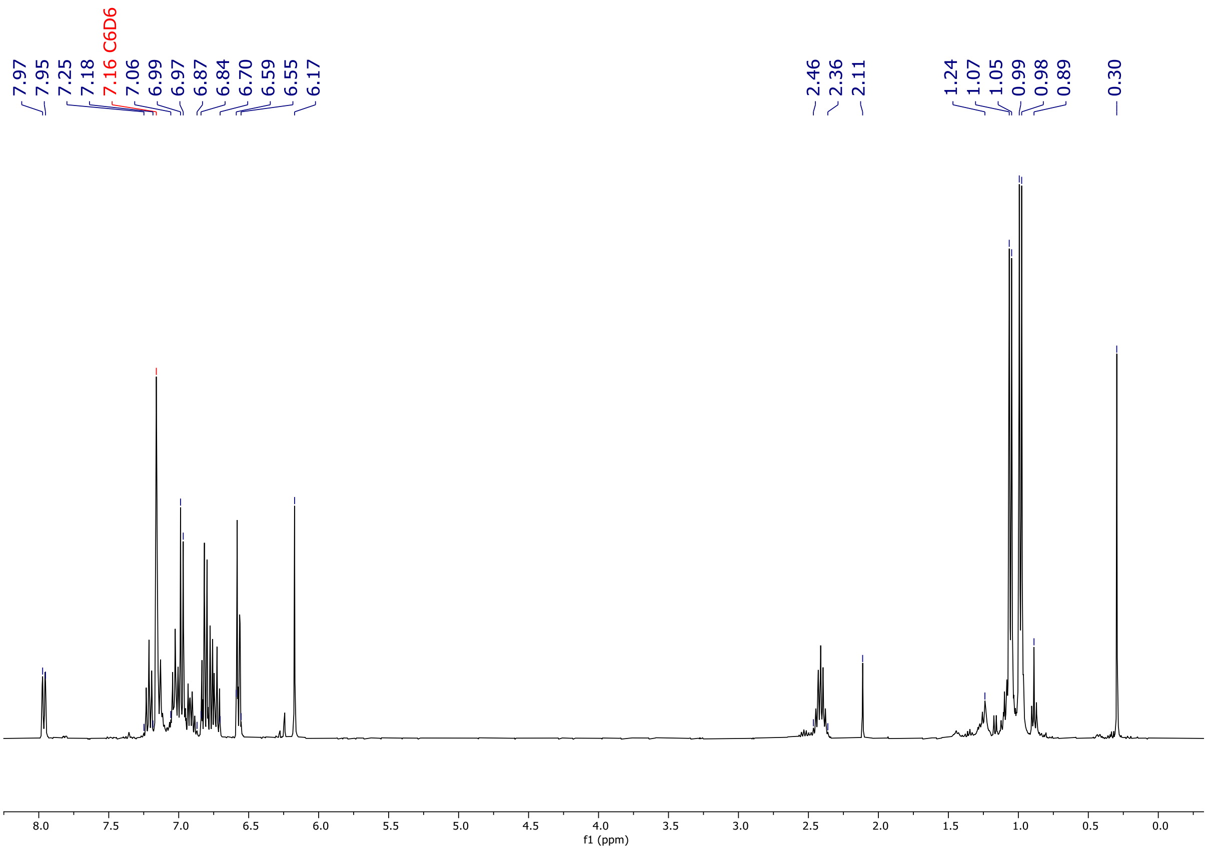

**Figure S8**. The $^{1}H$ NMR spectra (400 MHz, $C_6D_6$) of the reaction of (IPr)CuPh and PhNCO at room temperature.

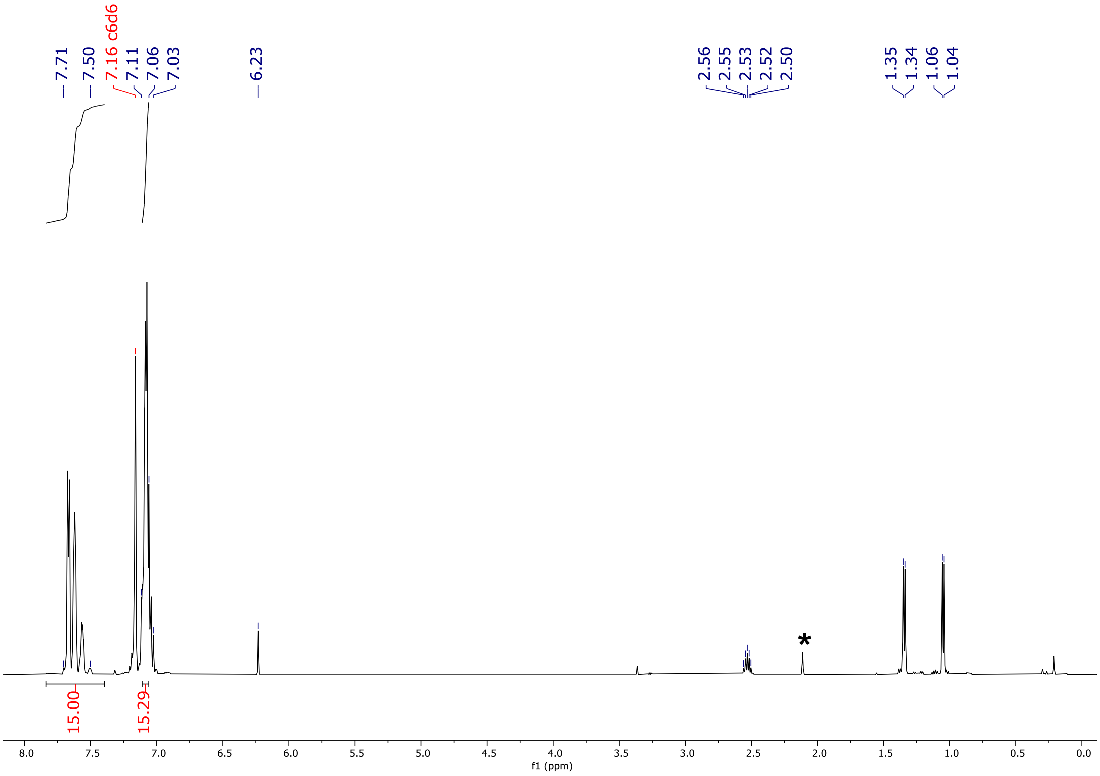

**Figure S9.** The $^{1}H$ NMR spectrum (500 MHz, $C_6D_6$) of the reaction of compound **2** and $Ph_3SnCl$. Integrated resonances correspond to $Ph_3GeSnPh_3$.[5] Non-integrated resonances correspond to (IPr)CuCl.[6] * = residual toluene.[4]

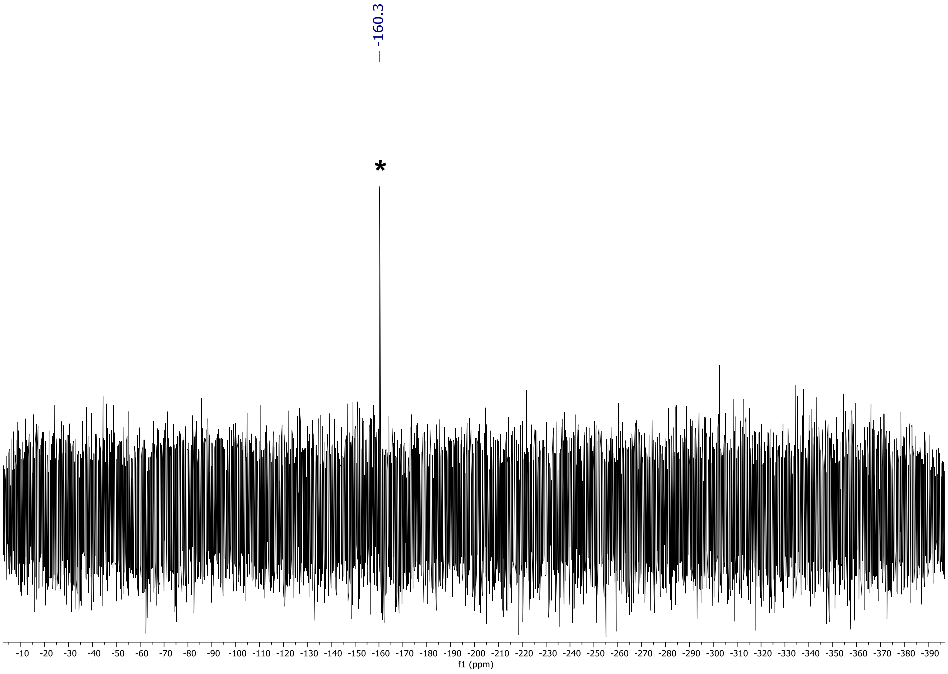


**Figure 10.** The $^{119}$Sn NMR spectrum (186 MHz, $C_6D_6$) of of the reaction of compound **2** and $Ph_3SnCl$. * = $Ph_3Ge\underline{Sn}Ph_3$.[5]

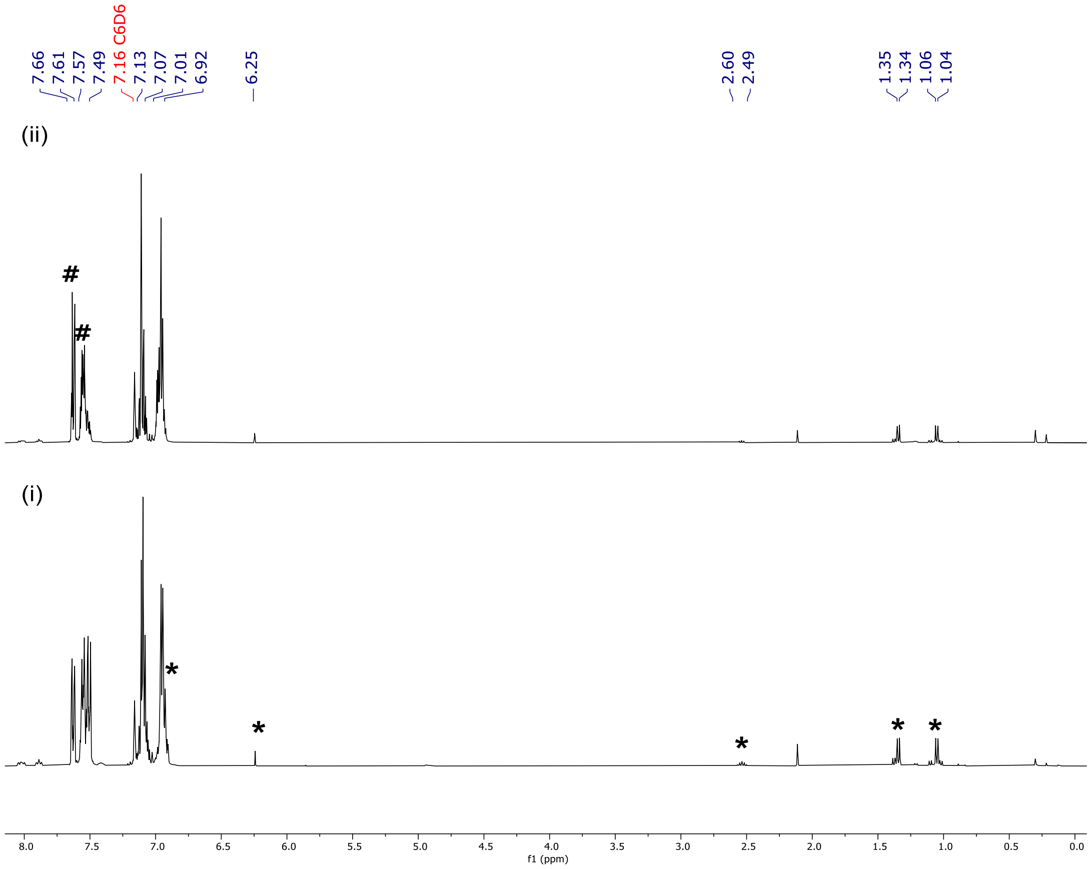


**Figure S11.** The $^{1}$H NMR spectra (400 MHz, $C_6D_6$) of the reaction of compound **2** and $Ph_2PCl$. (i) = After one equivalent of $Ph_2PCl$. (ii) = After a second equivalent of $Ph_2PCl$. * = (<u>IPr</u>)CuCl.[6] # = <u>Ph<sub></sub></u>$\underline{Ph_3}$GeCl.[7]

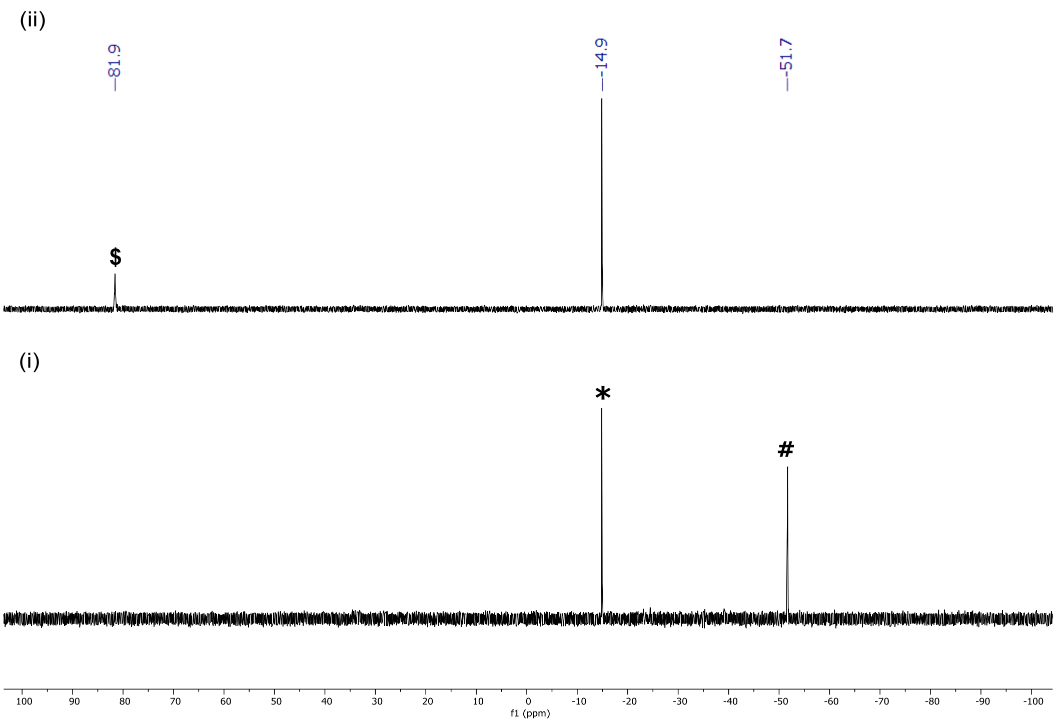


**Figure S12.** The $^{31}P$ NMR spectra (162 MHz, $C_6D_6$) of the reaction of compound **2** and $Ph_2PCl$. (i) = Addition of one equivalent of $Ph_2PCl$; (ii) = Addition of a second equivalent of $Ph_2PCl$. \$ = Excess $Ph_2PCl$.[8] * = $Ph_2PPPh_2$.[8] . # = $\underline{Ph_2}PGePh_3$.

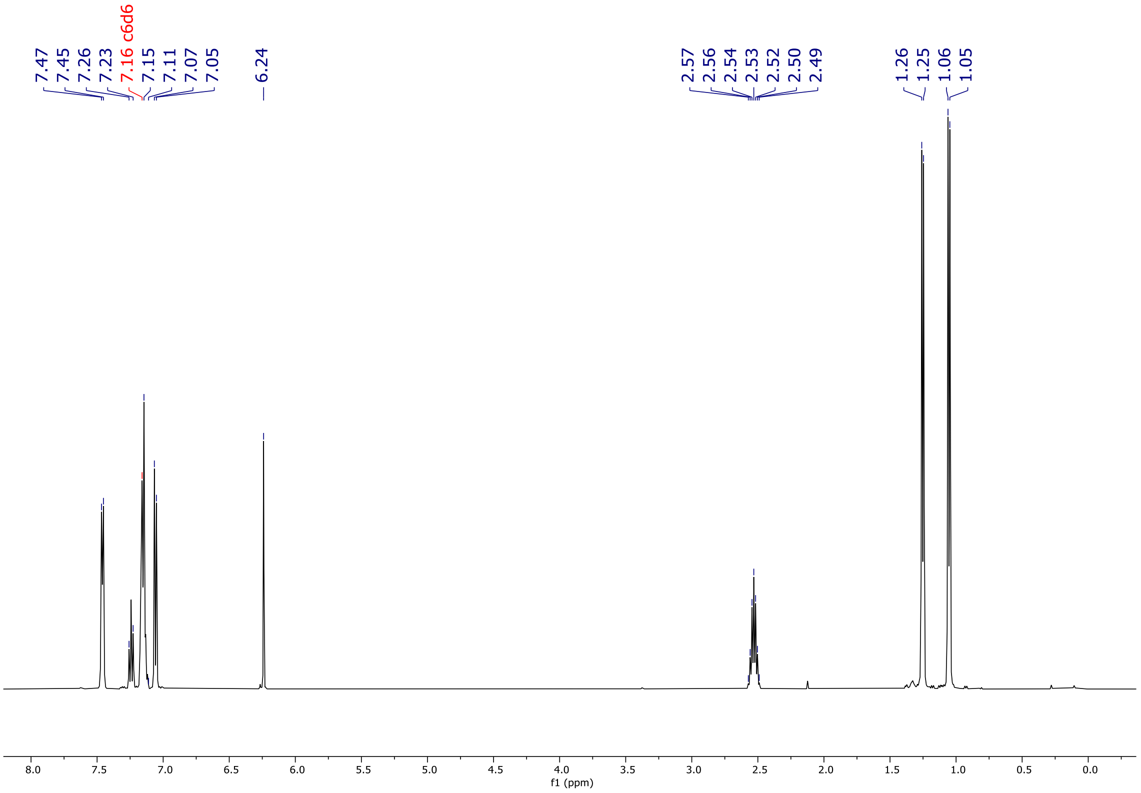


**Figure S13.** The $^1H$ NMR spectrum (500 MHz, $C_6D_6$) of the reaction of compound **2** and carbon dioxide.

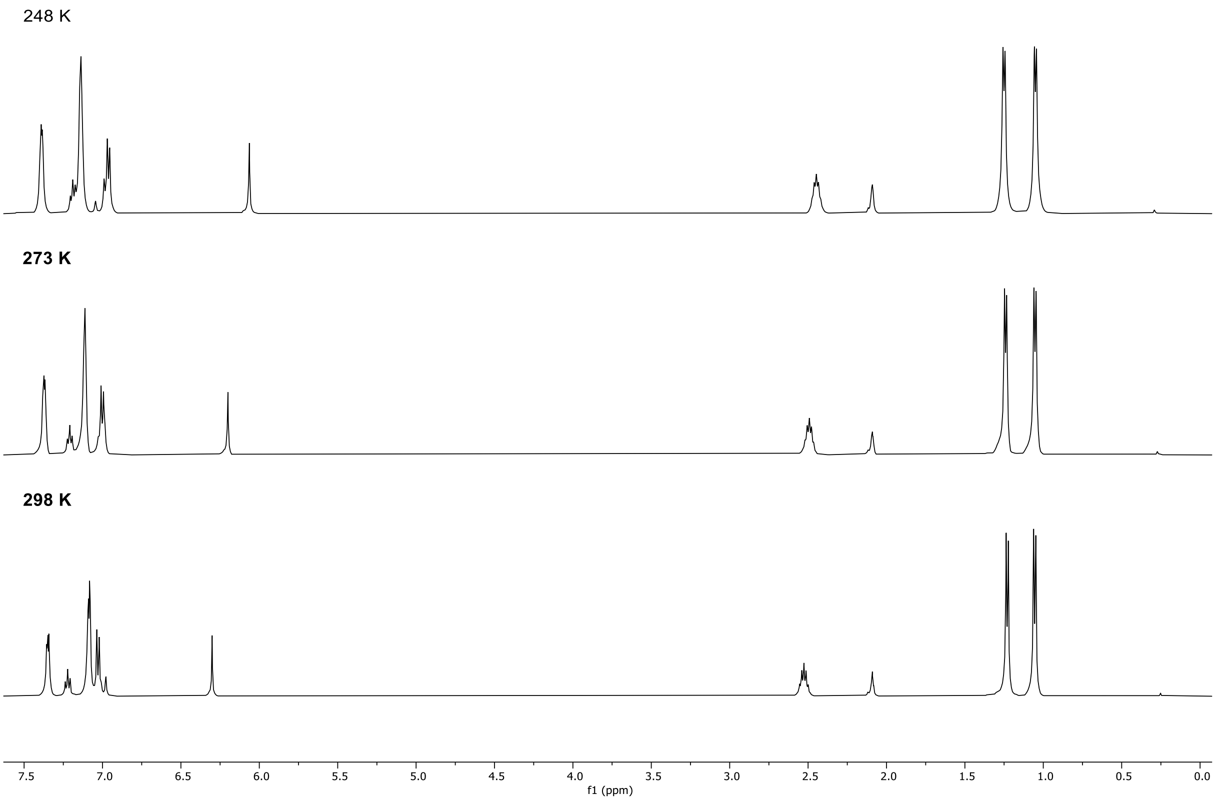


**Figure S14.** The $^{1}H$ VT-NMR spectrum (500 MHz, toluene-$d_8$) of the reaction of compound **2** and carbon dioxide.

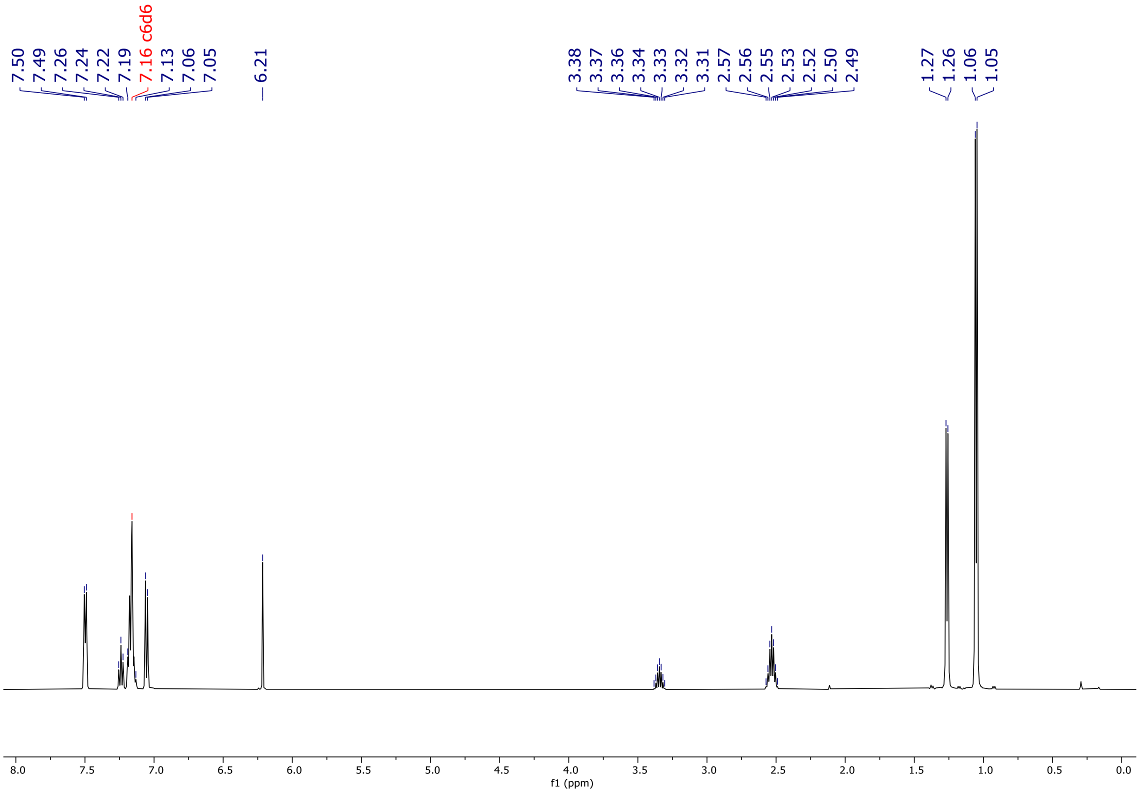


**Figure S15.** The $^{1}H$ NMR spectrum (500 MHz, $C_6D_6$) of the reaction of compound **2** and di-iso-propyl carbodiimide at room temperature.

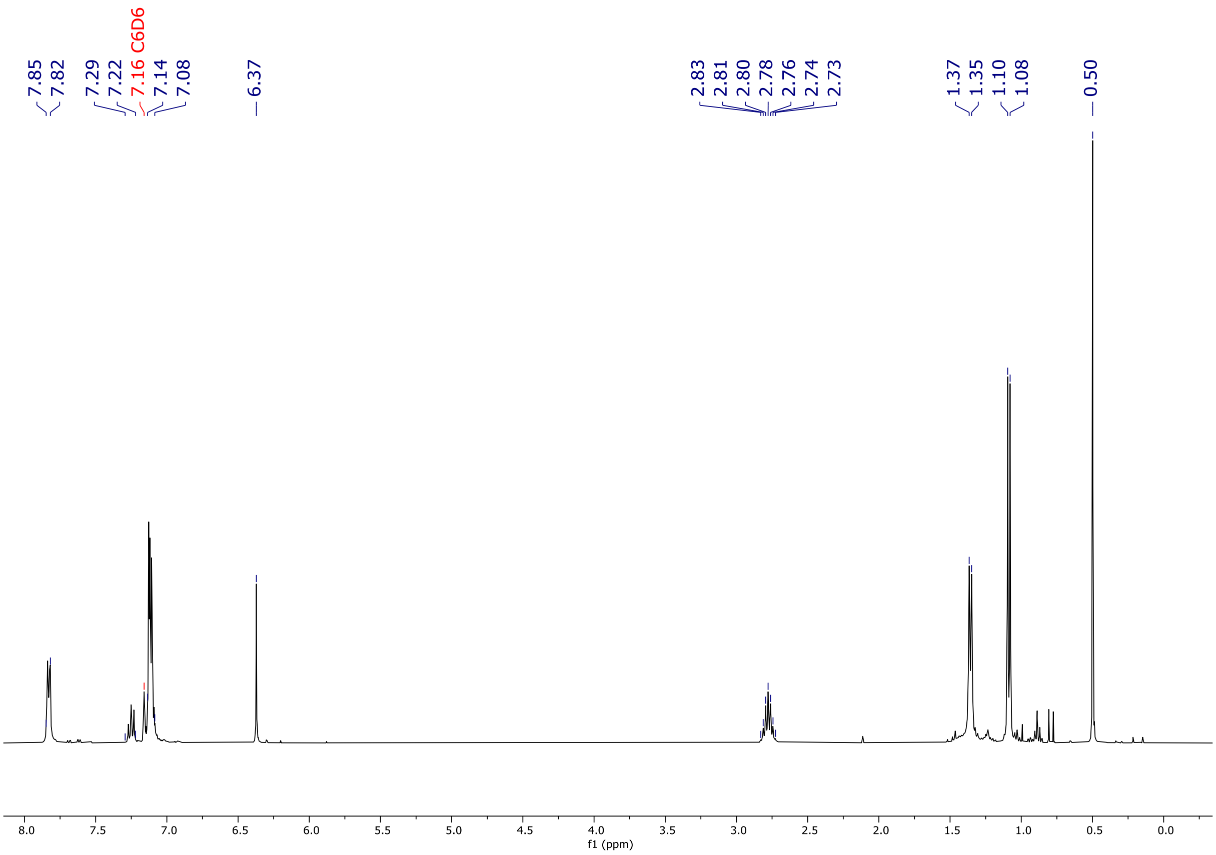


**Figure S16.** The $^{1}H$ NMR spectrum (400 MHz, $C_6D_6$) of compound **5** after heating at 120 °C overnight.

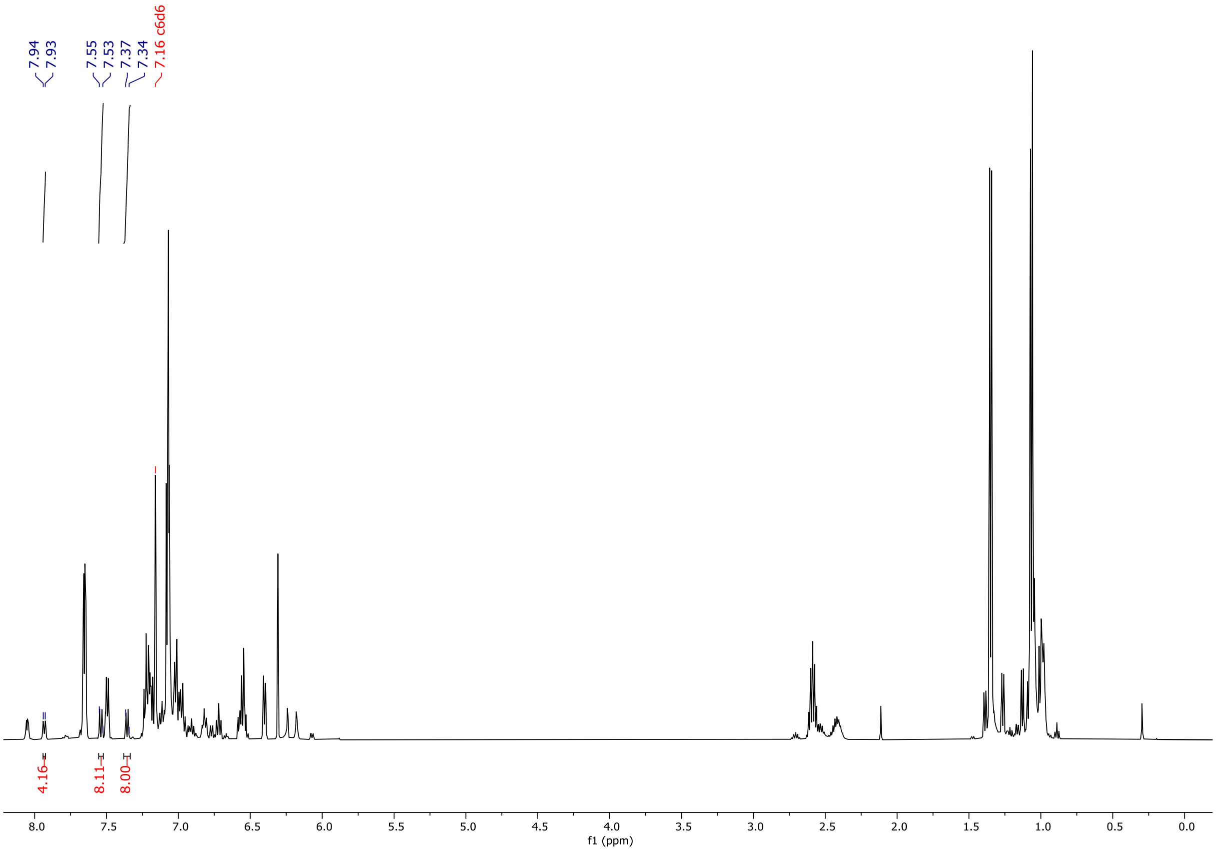


**Figure S17.** The $^{1}H$ NMR spectrum (500 MHz, $C_6D_6$) of the reaction of compound **2** and phenyl isocyanate. Integrated resonances correspond to $Ph_4Ge$.[9]

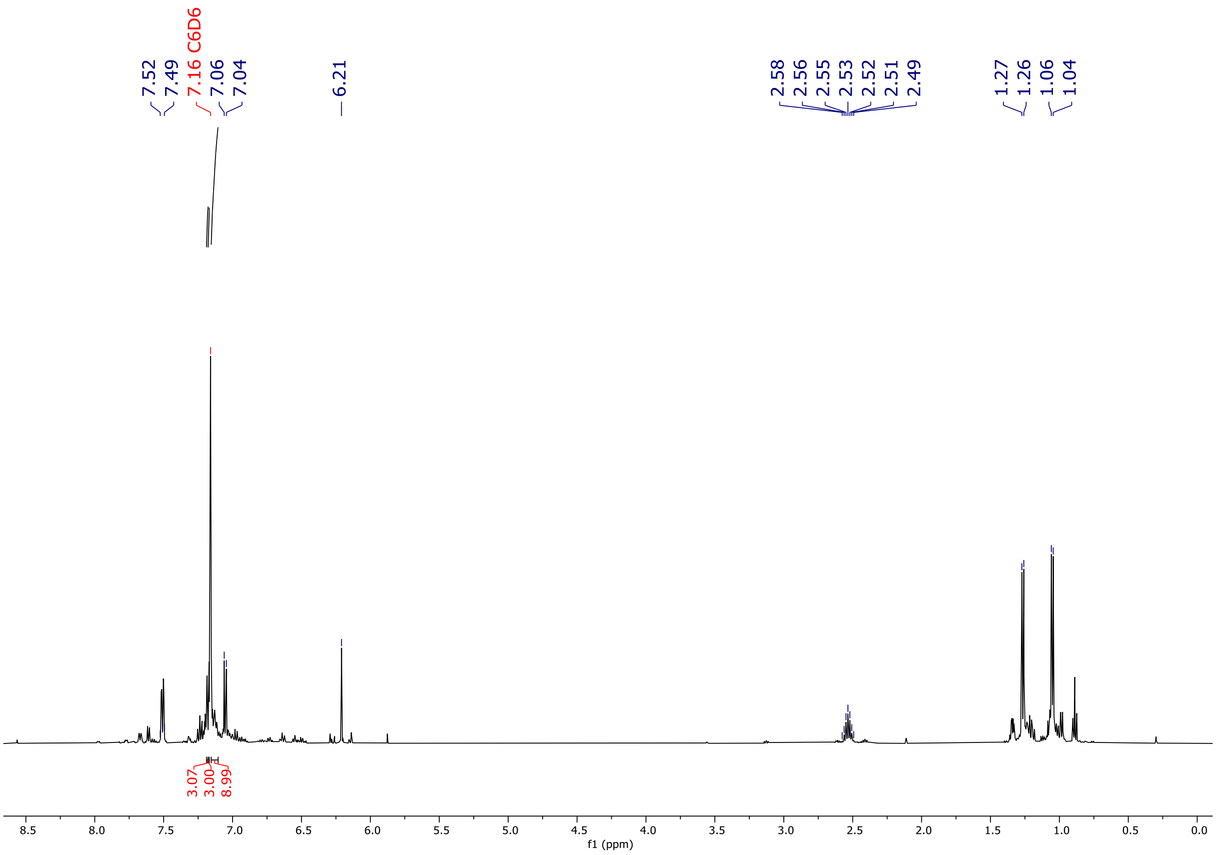


**Figure S18.** The $^1$H NMR spectrum (500 MHz, $C_6D_6$) of compound **8** after heating at 120 °C for 4 days. Integrated resonances correspond to $[PhNCO]_3$.[10]

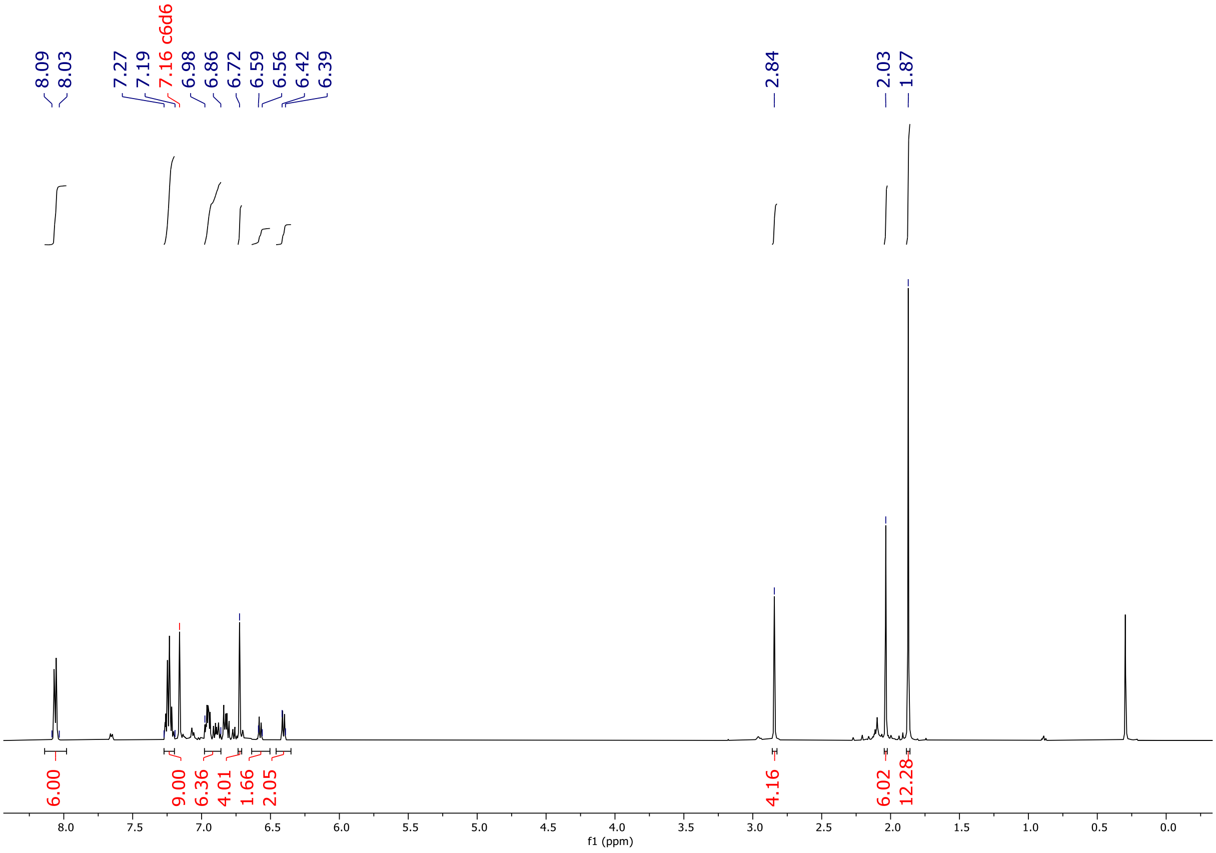


**Figure S19.** The $^1$H NMR spectrum (500 MHz, $C_6D_6$) of compound **1** and PhNCO. Integrated resonances correspond to (SIMes)CuN(Ph)C(O)N(Ph)C(O)$GePh_3$.

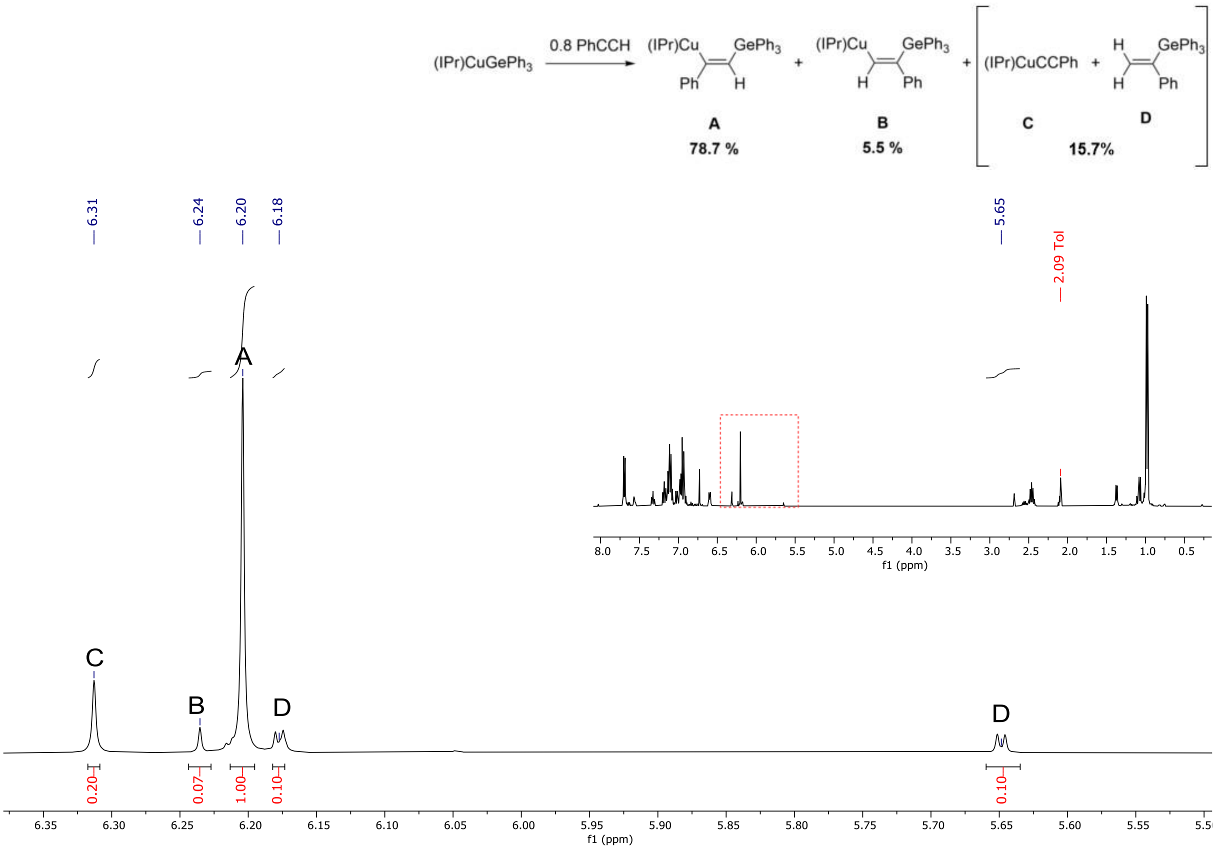


**Figure S20.** The $^{1}H$ NMR spectrum (400 MHz, Toluene-$d_8$) of the reaction of compound **2** and 0.8 equivalents of phenyl acetylene at -40 °C. Ratio of product yields are illustrated A-D.[11]

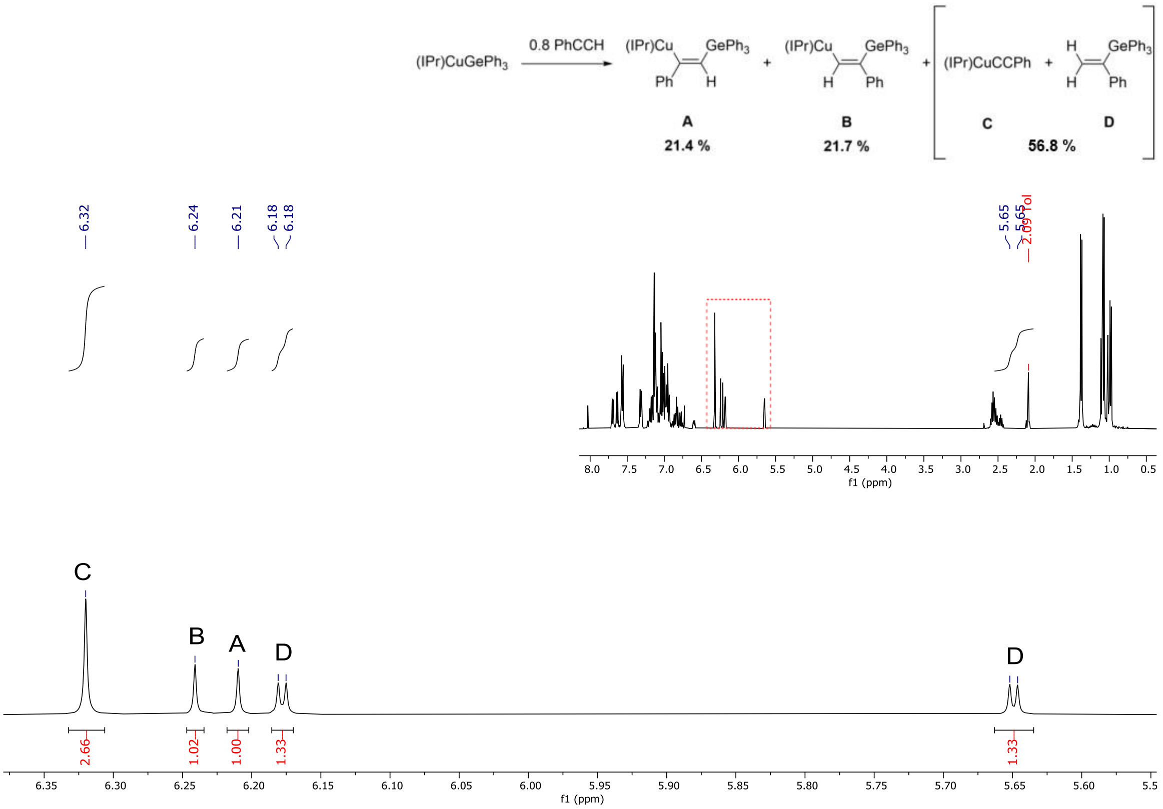


**Figure S21.** The $^{1}H$ NMR spectrum (400 MHz, Toluene-$d_8$) of the reaction of compound **2** and 0.8 equivalents of phenyl acetylene at room temperature. Ratio of product yields are illustrated A-D.[1]

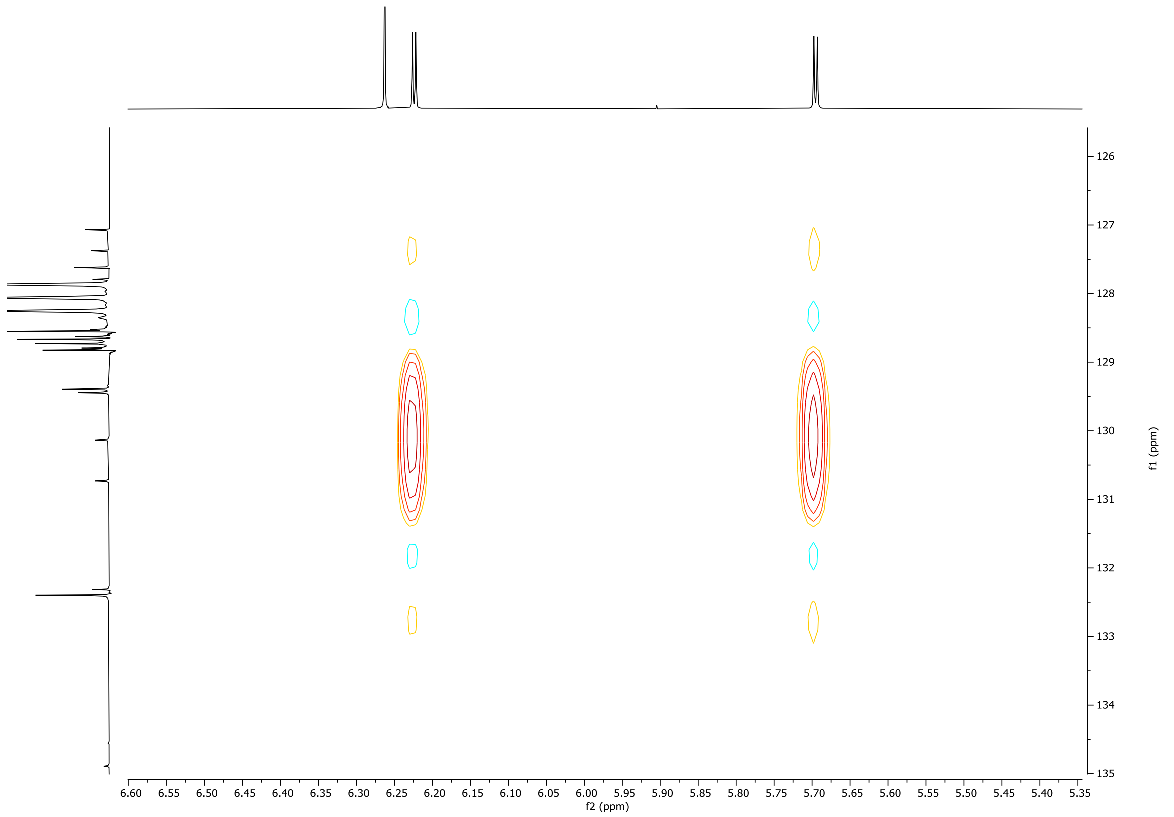

**Figure S22.** The $^{1}H$–$^{13}C$ HSQC NMR spectrum (500 MHz, $C_6D_6$) of the reaction of compound **2** and two equivalents of phenyl acetylene after 3 days at room temperature for confirmation of the geometry of compound **D**, $PhC(GePh_3)=CH_2$, figure S14.

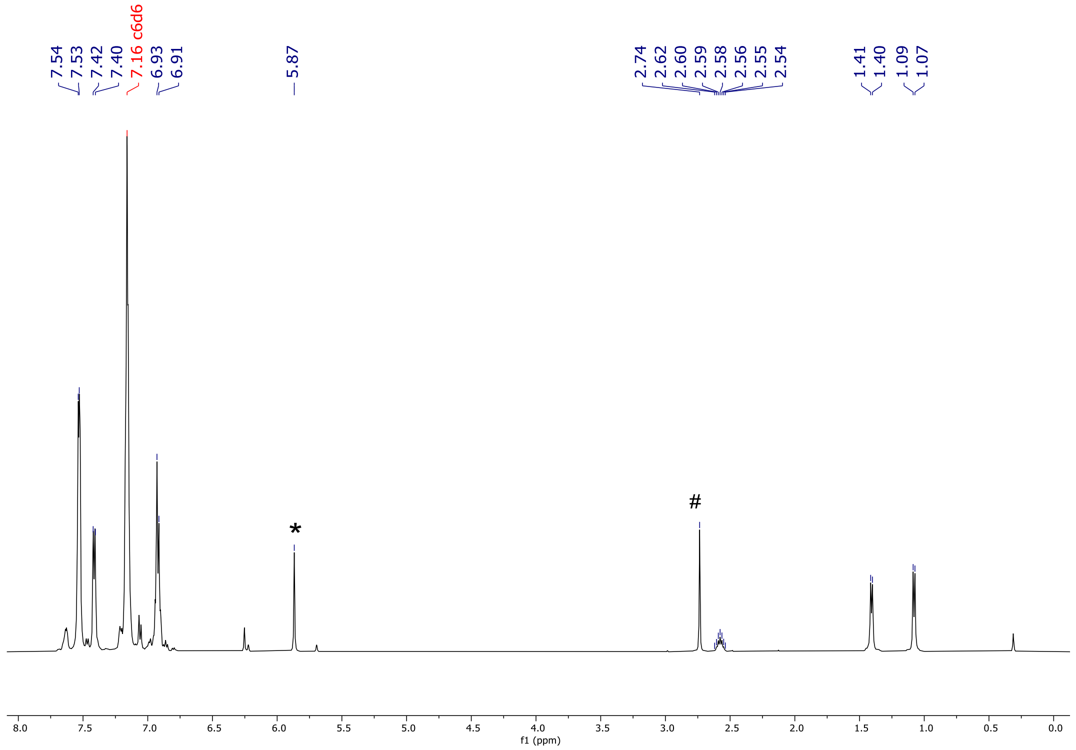


**Figure S23.** The $^{1}H$ NMR spectrum (500 MHz, $C_6D_6$) of the reaction of 10 mol% compound **2** with an equivalent of phenyl acetylene and $Ph_3GeH$ after overnight at 60 °C. # = PhCC<u>H</u>;[14] * = $Ph_3Ge$<u>H</u>.[12] Remaining labelled resonances in spectrum correspond to N,N'-bis(2,6-diisopropylphenyl)imidazol-2-ylidene(phenylacetylido)copper(I).[11]

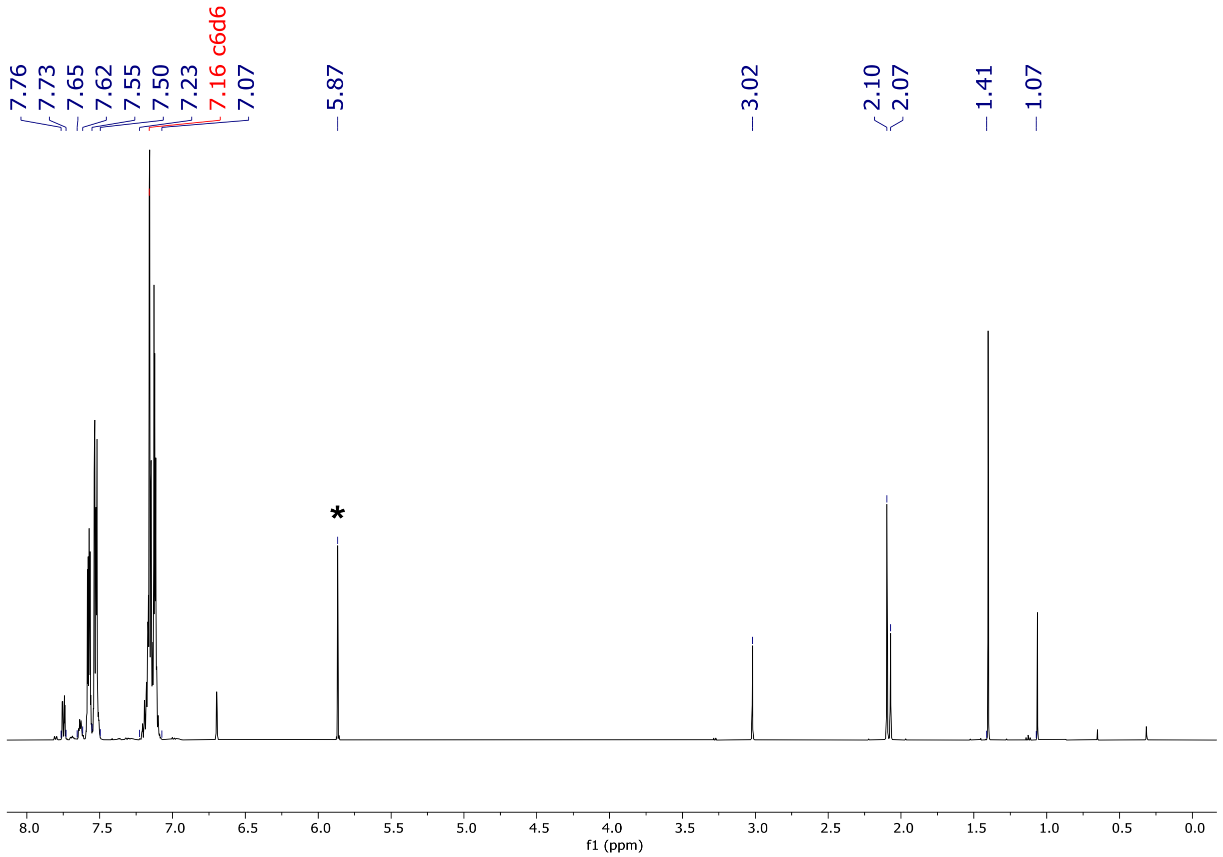


**Figure S24.** The $^{1}H$ NMR spectrum (500 MHz, $C_6D_6$) of the reaction of 10 mol% (SIMes)CuOtBu with $Ph_3GeH$ and $Ph_3SnCl$ at 60 °C overnight. * = $Ph_3Ge\underline{H}$.[12]

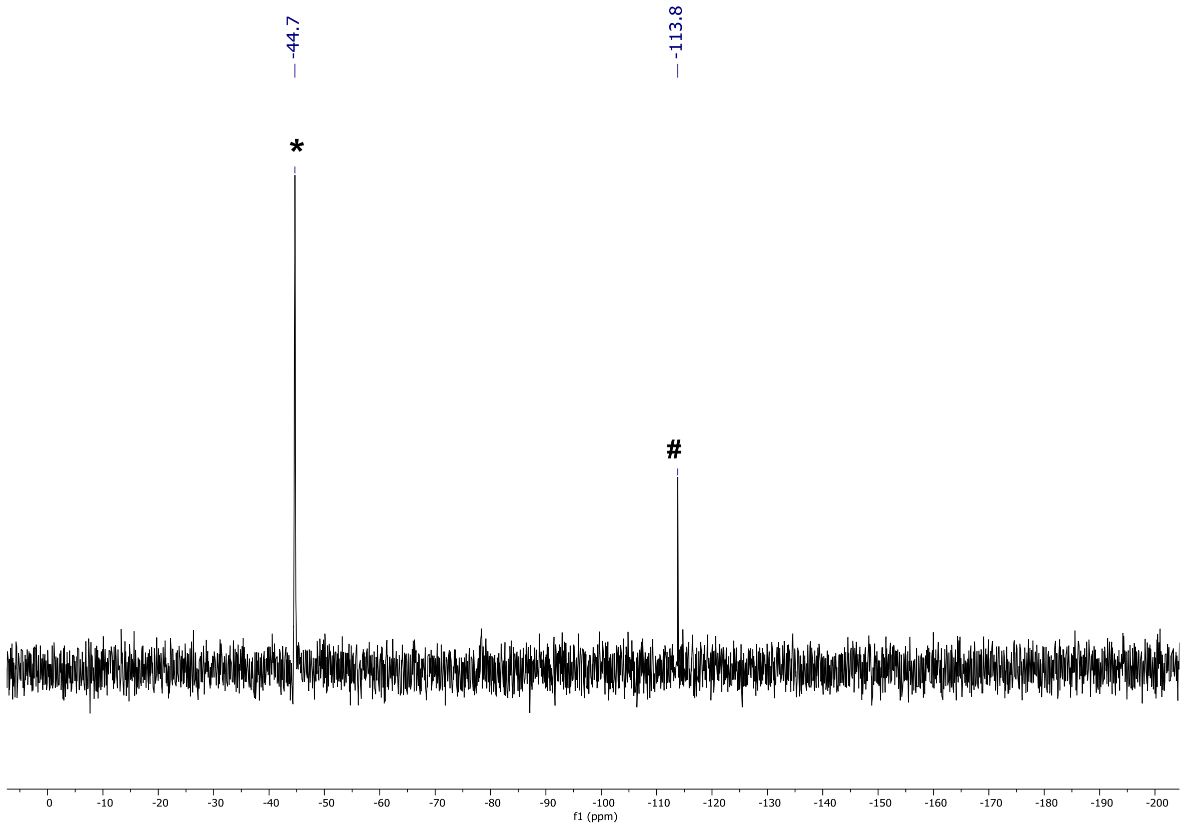


**Figure S25.** The $^{119}Sn$ NMR spectrum (186 MHz, $C_6D_6$) of the reaction of 10 mol% (SIMes)CuOtBu with $Ph_3GeH$ and $Ph_3SnCl$ at 60 °C overnight. * = $Ph_3SnCl$.[13] # = $Ph_3SnOtBu$.

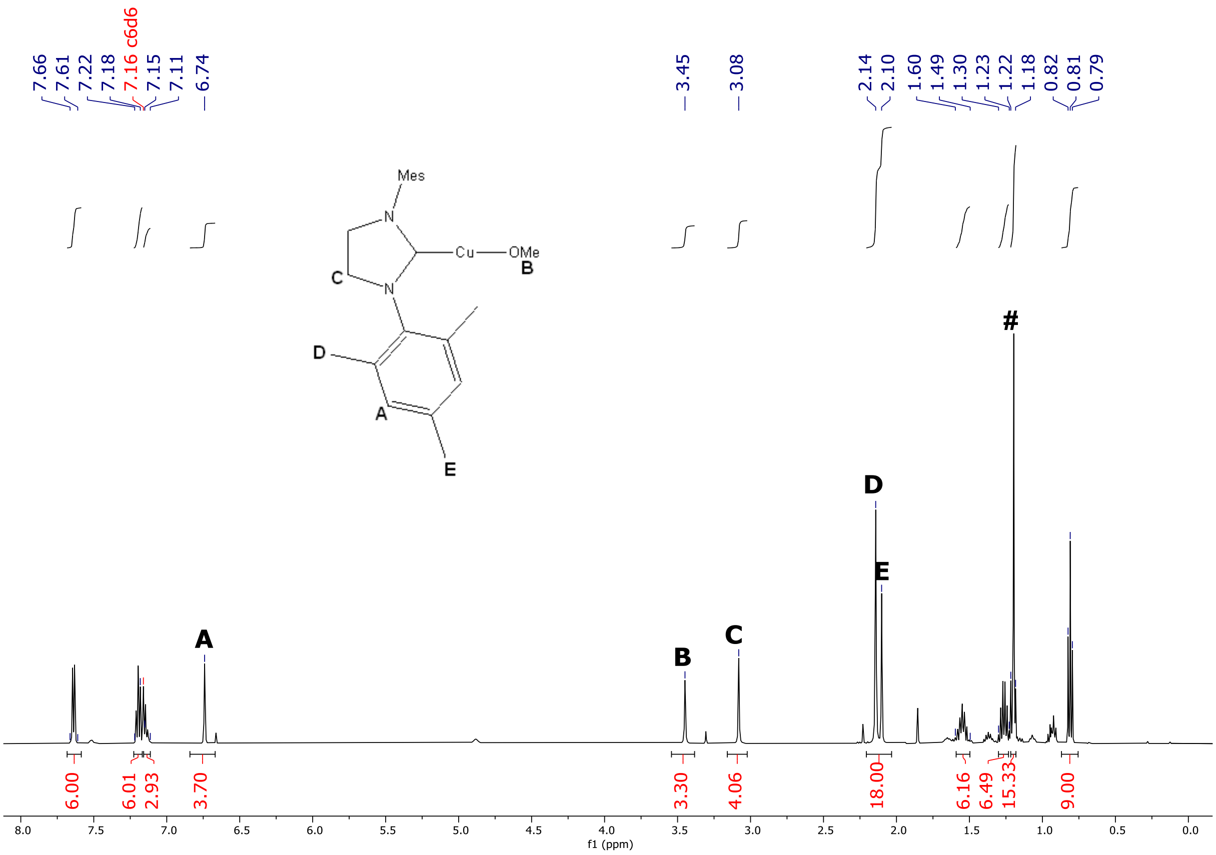

**Figure S26.** The $^{1}H$ NMR spectrum (500 MHz, $C_6D_6$) of the reaction of (SIMes)CuOtBu with $Ph_3GeH$ and $Bu_3SnOMe$ at room temperature. Unlabelled integrated resonances correspond to $Ph_3GeSnBu_3$. Labelled integrated resonances (A-E) correspond to (SIMes)CuOMe. # =tBuOH.[4]

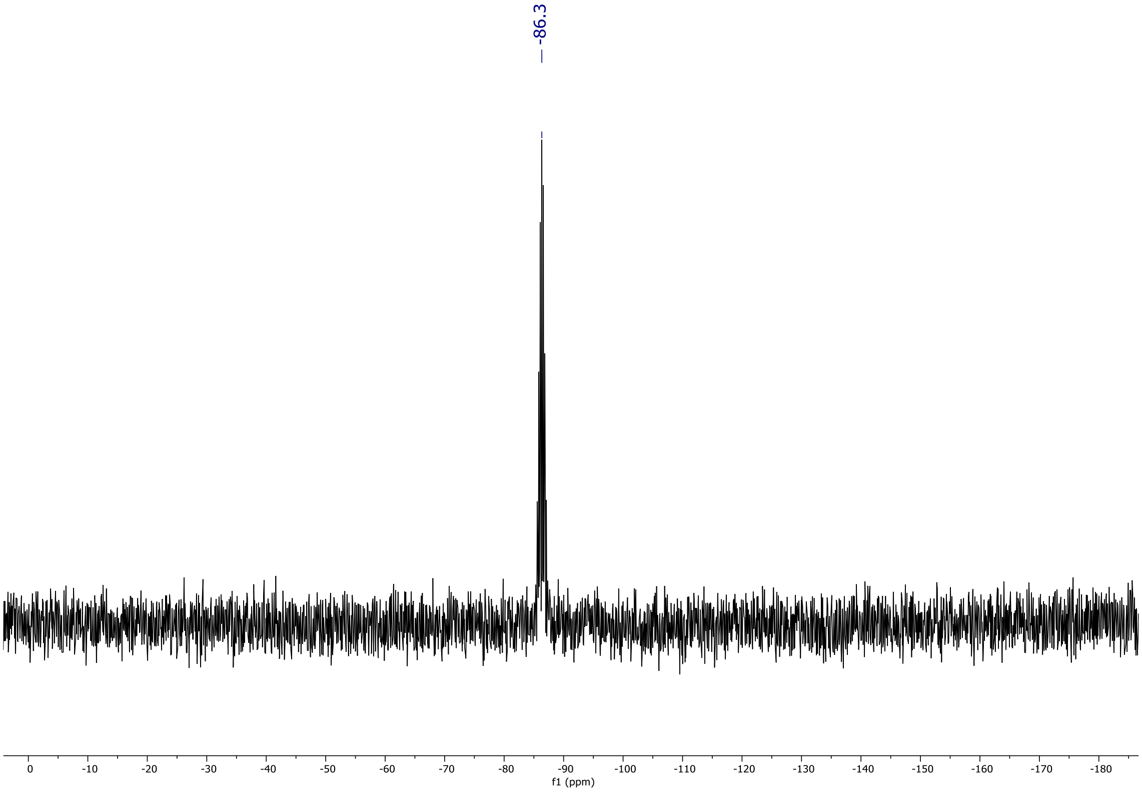

**Figure S27.** The $^{119}Sn$ NMR spectrum (186 MHz, $C_6D_6$) of reaction of (SIMes)CuOtBu with $Ph_3GeH$ and $Bu_3SnOMe$ at room temperature.

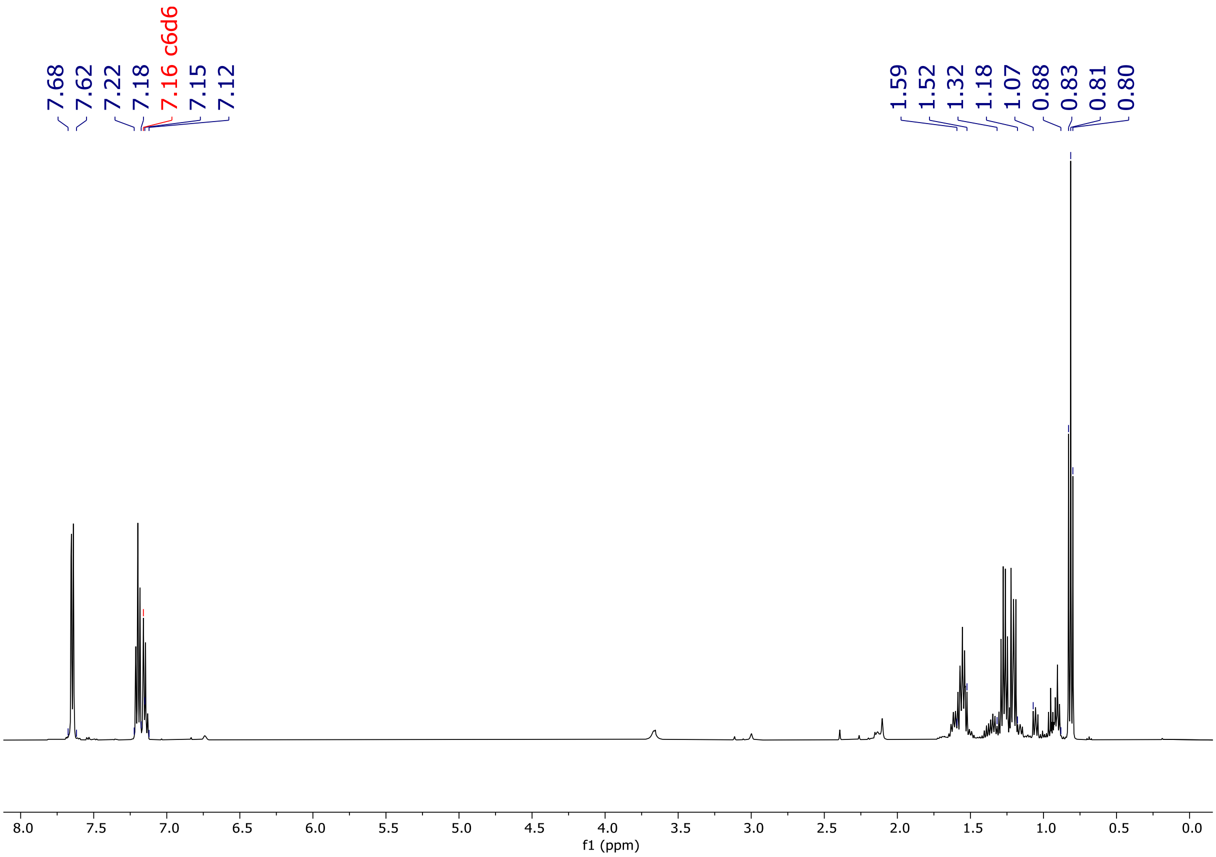


**Figure S28.** The $^{1}H$ NMR spectrum (500 MHz, $C_6D_6$) of the reaction of 10 mol% (SIMes)CuOtBu with $Ph_3GeH$ and $Bu_3SnOMe$ after volatiles were removed *in vacuo*.

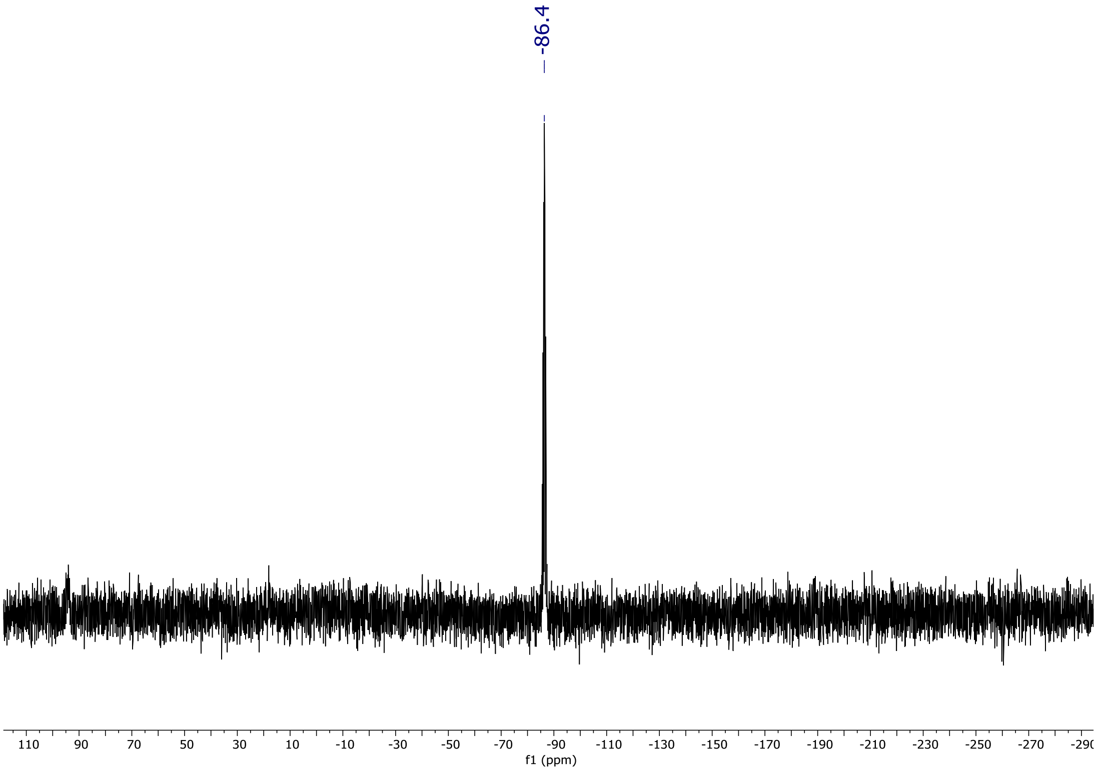


**Figure S29.** The $^{119}Sn$ NMR spectrum (186 MHz, $C_6D_6$) of the reaction of 10 mol% (SIMes)CuOtBu with $Ph_3GeH$ and $Bu_3SnOMe$ after volatiles were removed *in vacuo*.

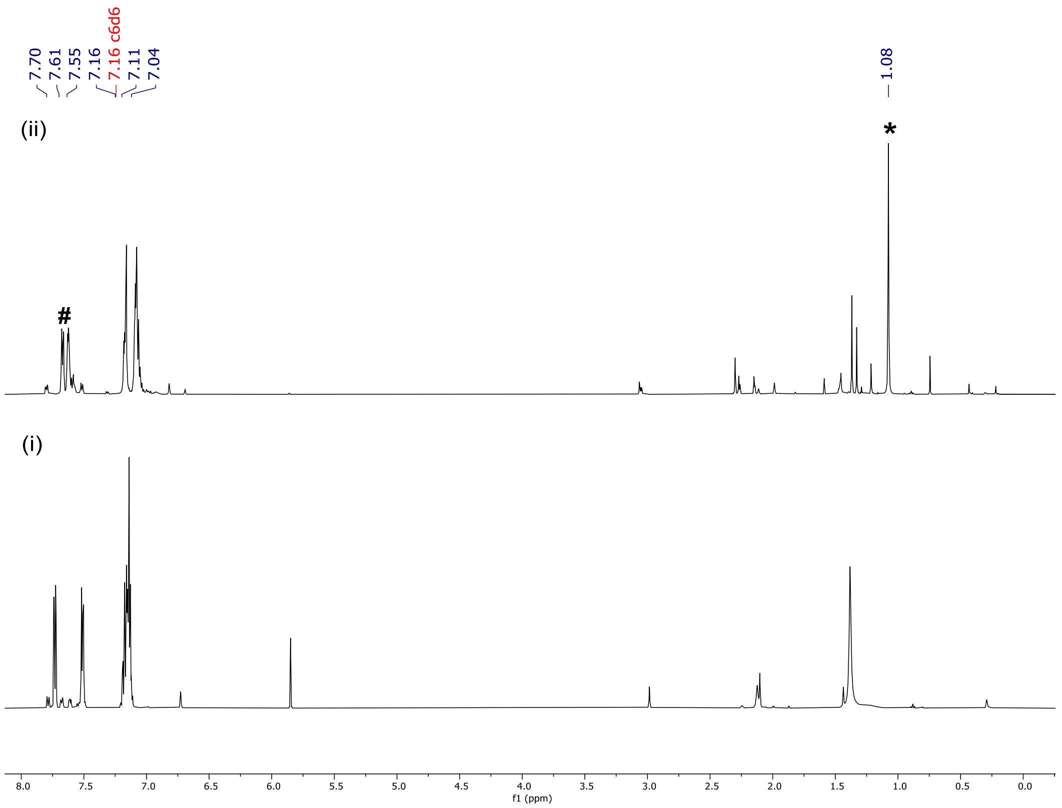


**Figure S30.** The $^{1}$H NMR spectra (500 MHz, $C_6D_6$) of the reaction of 10 mol% (SIMes)CuOtBu with $Ph_3GeH$ and $Ph_3SnOtBu$. (i) = After addition at room temperature. (ii) = After heating at 100 °C overnight. # = $Ph_3GeSnPh_3$;[5] * = tBuOH.[4]

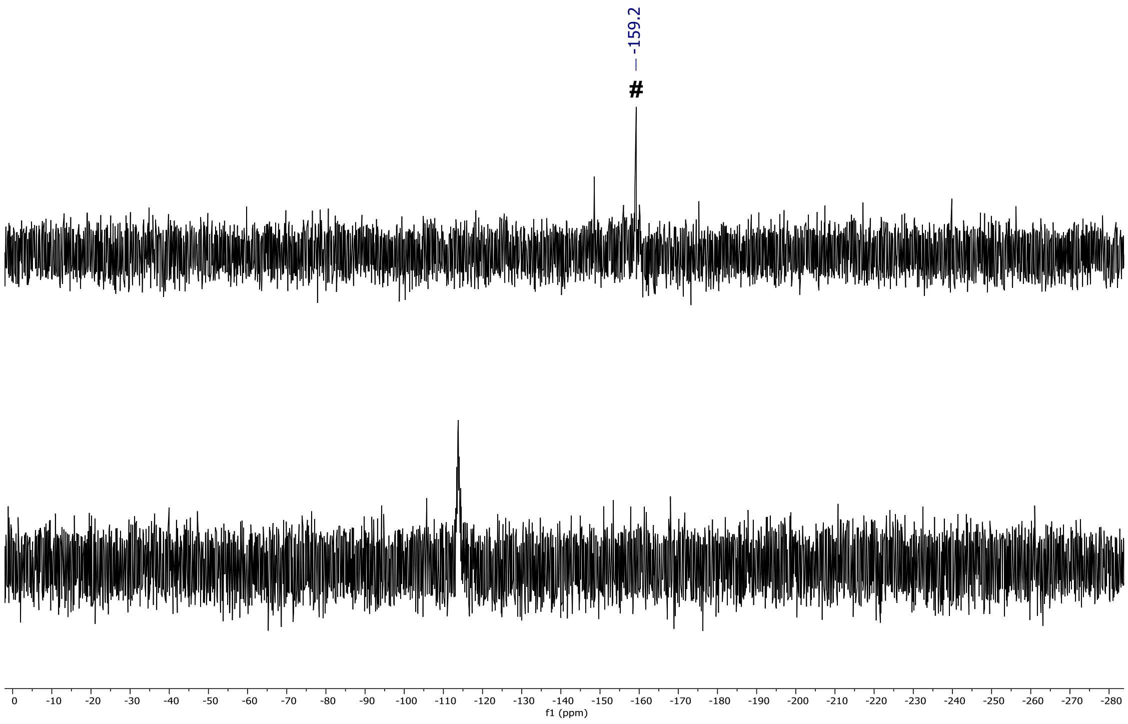


**Figure S31.** The $^{119}$Sn NMR spectra (186 MHz, $C_6D_6$) of reaction of 10 mol% (SIMes)CuOtBu with $Ph_3GeH$ and $Ph_3SnOtBu$. # = $Ph_3GeSnPh_3$.[5]

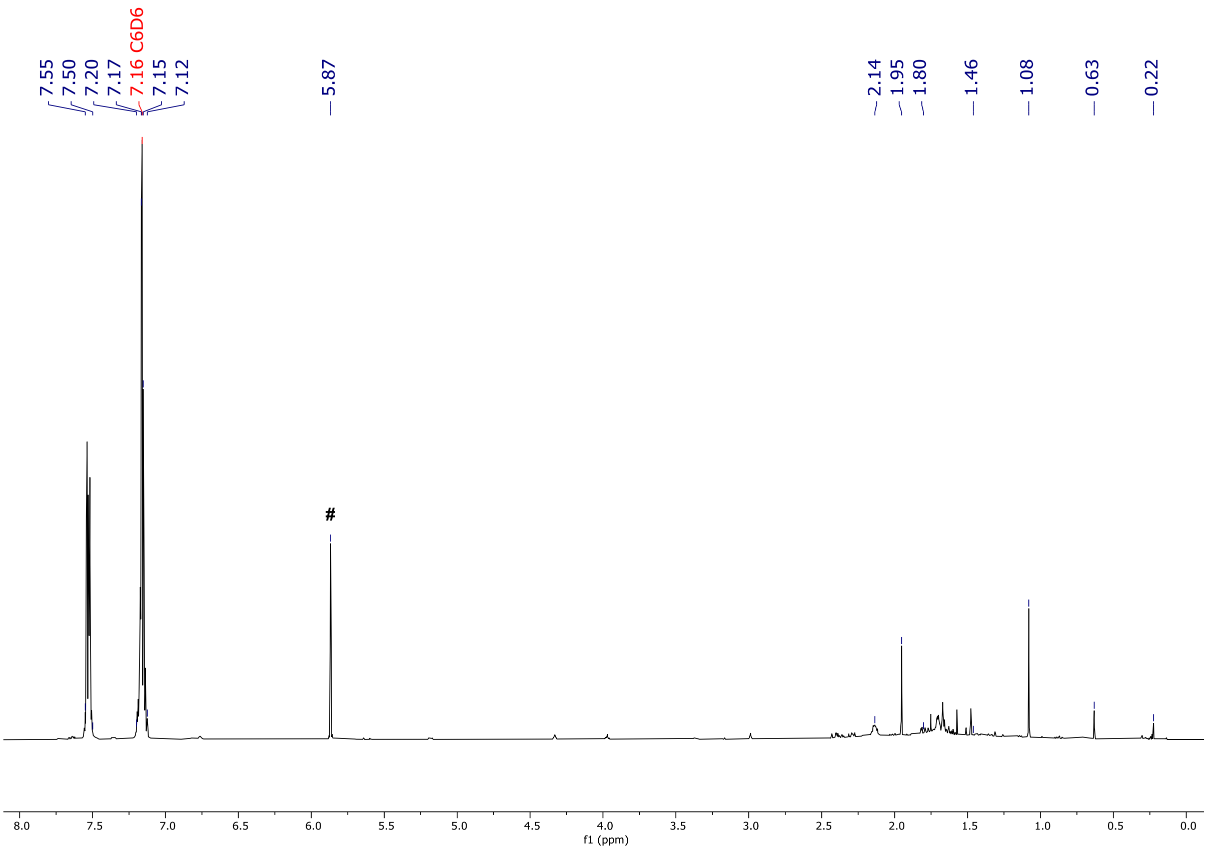


**Figure S32.** The $^{1}$H NMR spectrum (400 MHz, $C_6D_6$) of the reaction of 10 mol% (SIMes)CuOtBu with $Ph_3GeH$ and methyl vinyl ketone after heating at 80 °C for 3 days. # = $HGePh_3$.

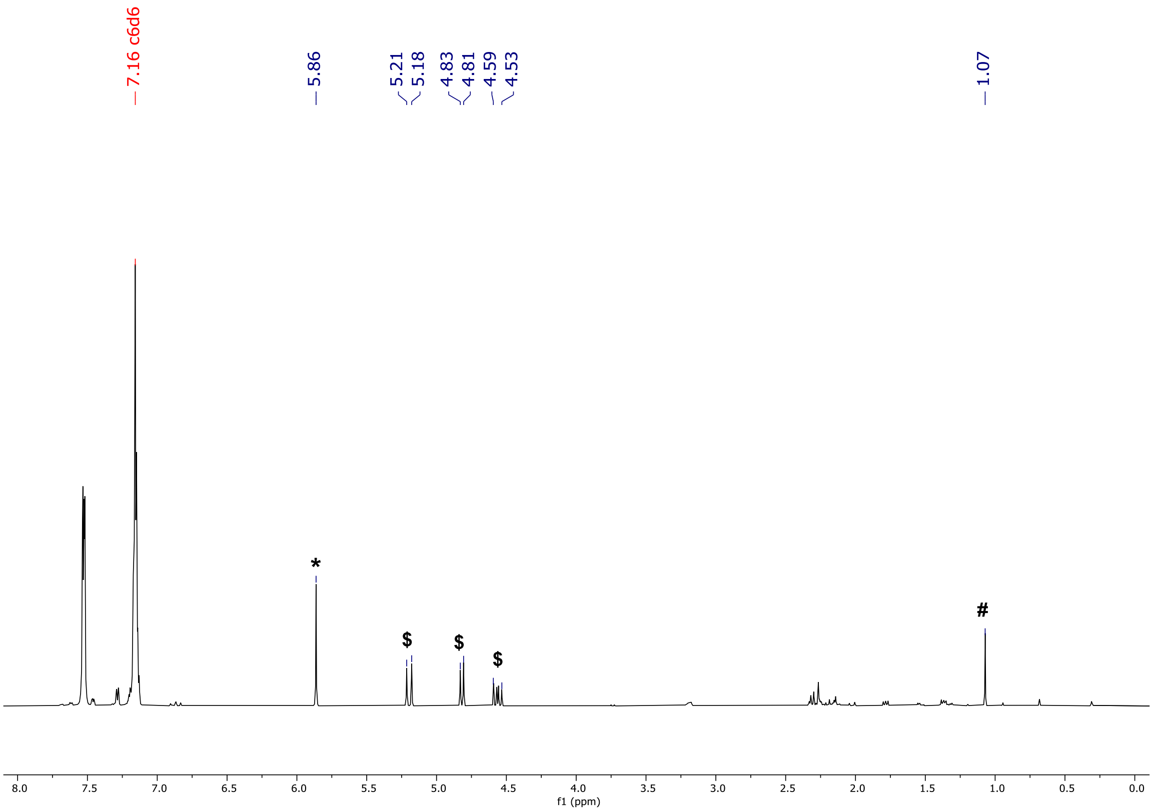


**Figure S33.** The $^{1}$H NMR spectrum (500 MHz, $C_6D_6$) of the reaction of 5 mol% (SIMes)CuOtBu with $Ph_3GeH$ and acrylonitrile after heating at 100 °C for 3 days. * = $HGePh_3$; # = *t*BuOH; $ = acrylonitrile.

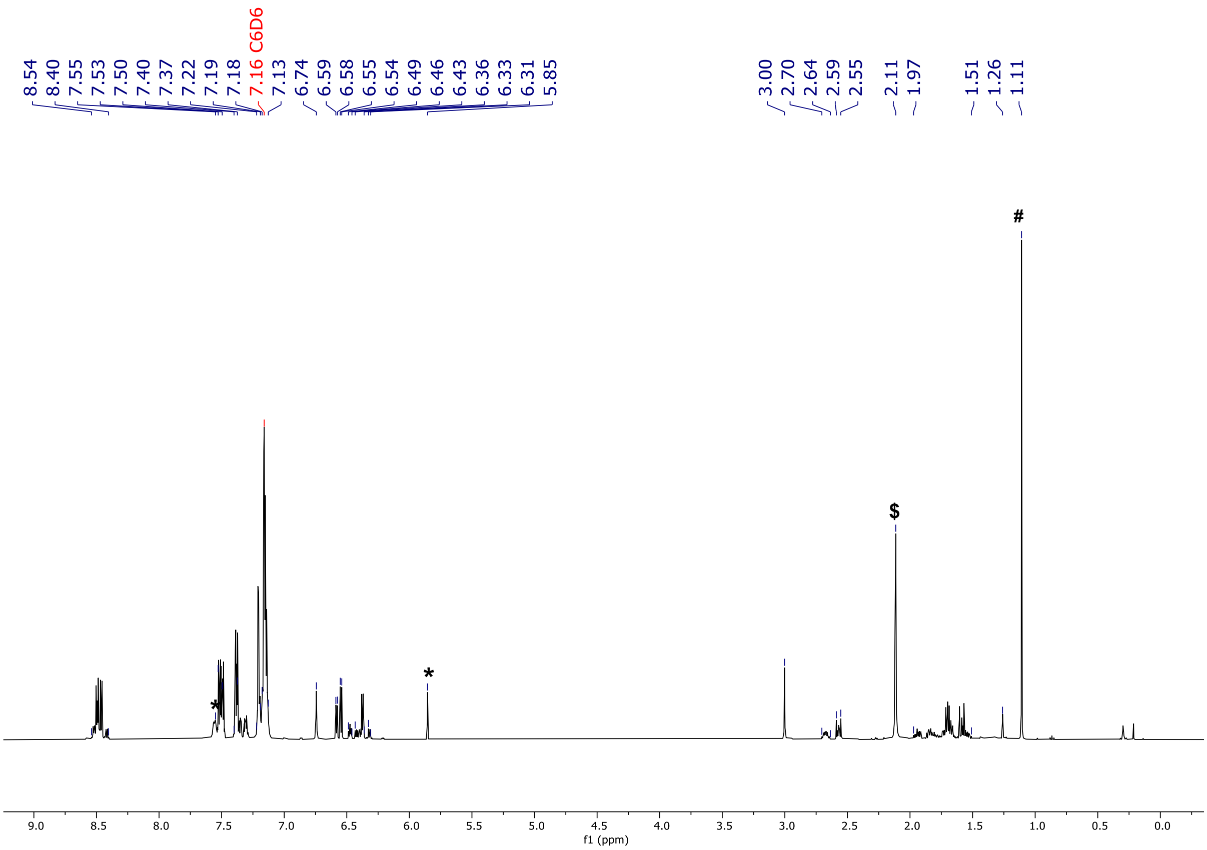


**Figure S34.** The $^{1}H$ NMR spectrum (500 MHz, $C_6D_6$) of the reaction of 10 mol% (SIMes)CuOtBu with $Ph_3GeH$ and 4-vinyl pyridine after heating at 60 °C for 3 days. * = $HGePh_3$; # = *t*BuOH; $ = toluene.

## NMR spectra of Compounds

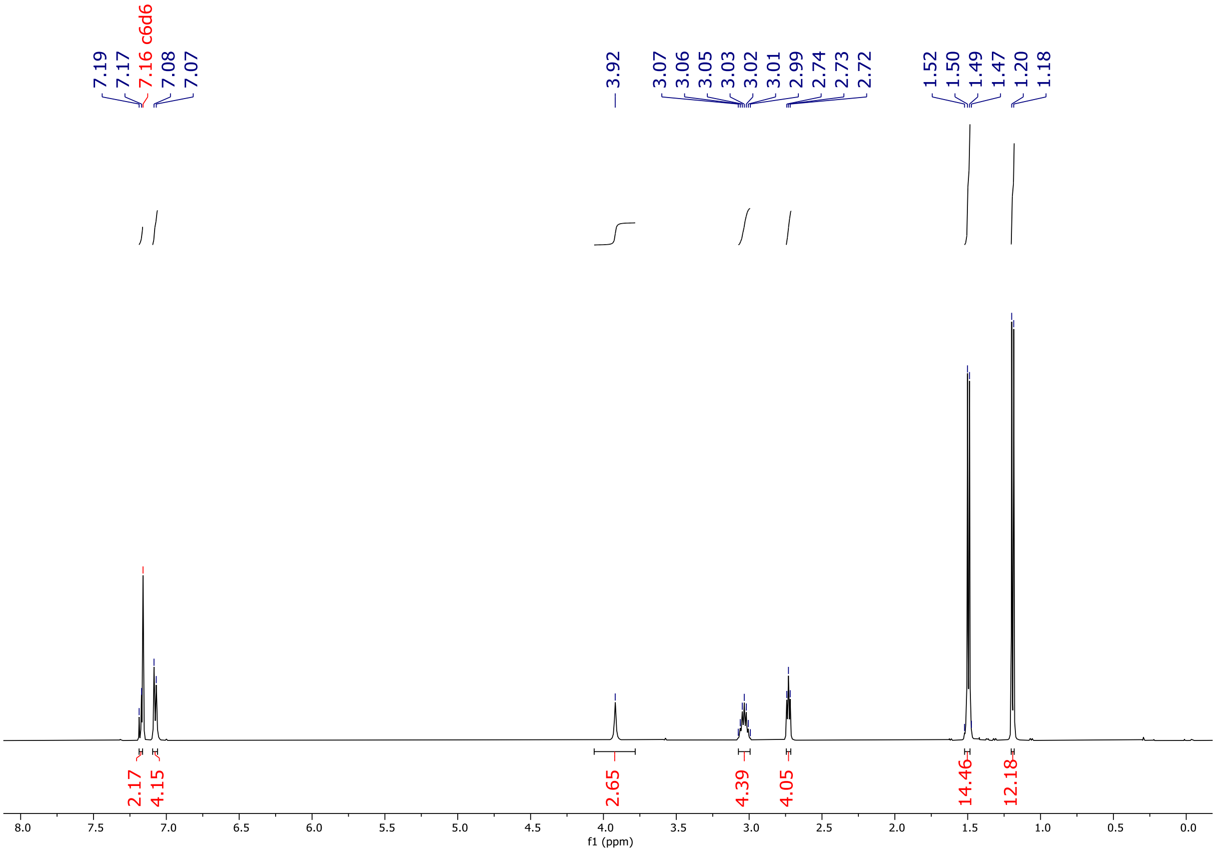


**Figure S35.** $^{1}H$ NMR spectrum (500 MHz, $C_6D_6$) of (6-Dipp)CuOMe.

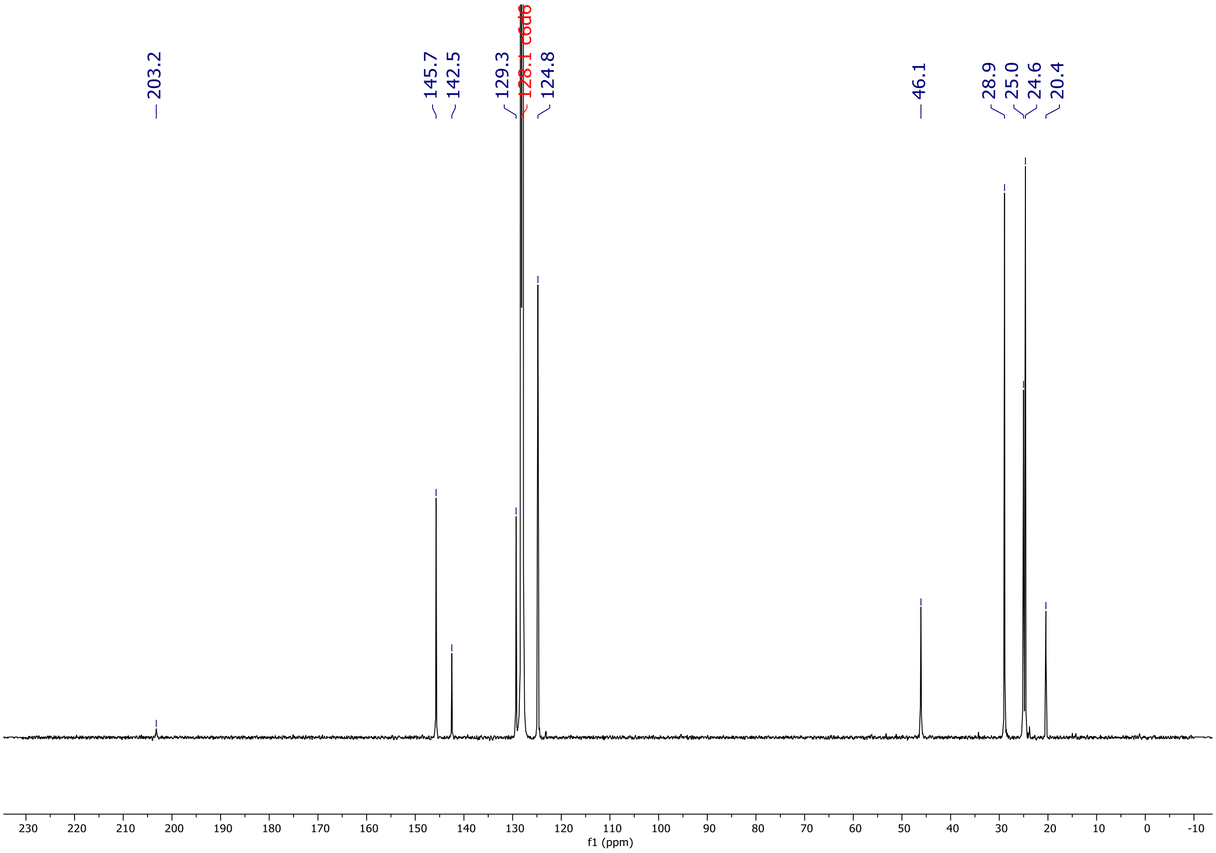


**Figure S36.** $^{13}C\{^{1}H\}$ NMR spectrum (126 MHz, $C_6D_6$) of (6-Dipp)CuOMe.

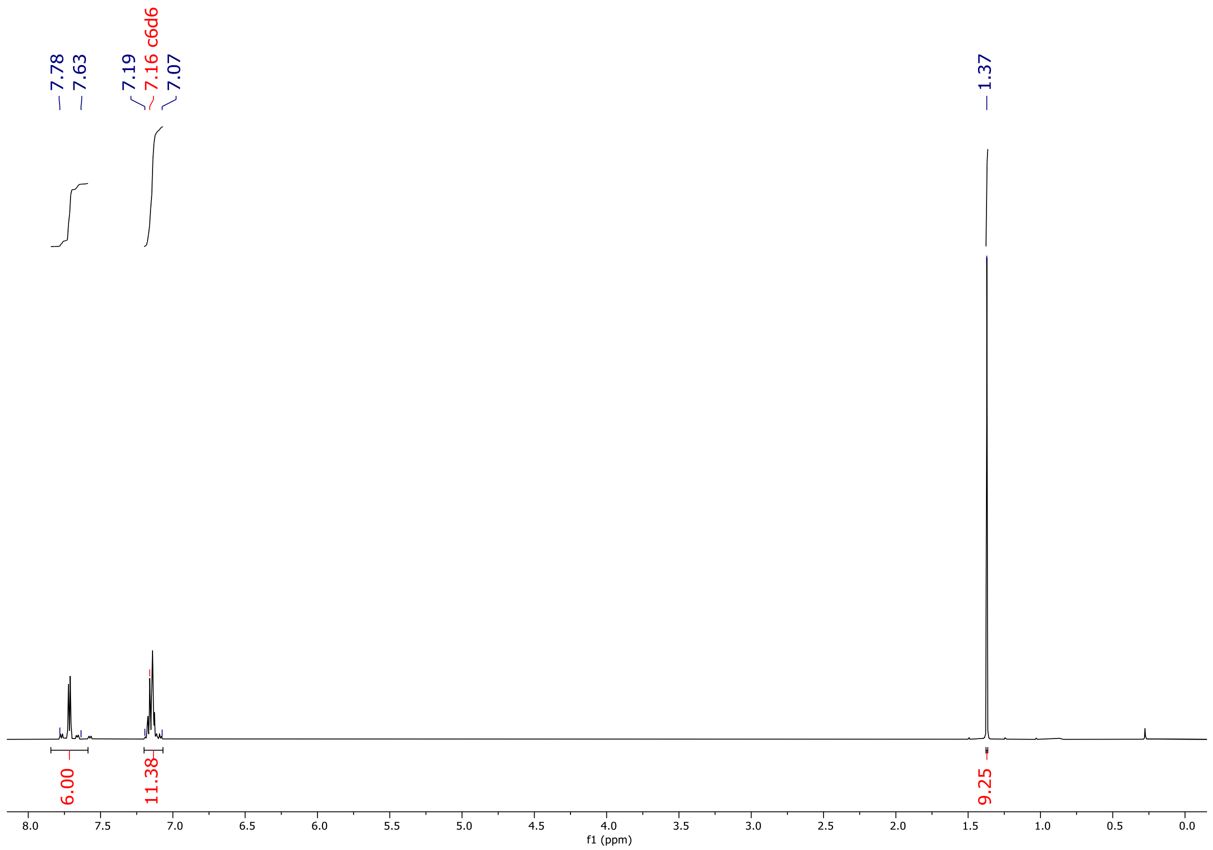


**Figure S37.** $^{1}H$ NMR spectrum (500 MHz, $C_6D_6$) of $Ph_3SnOtBu$.

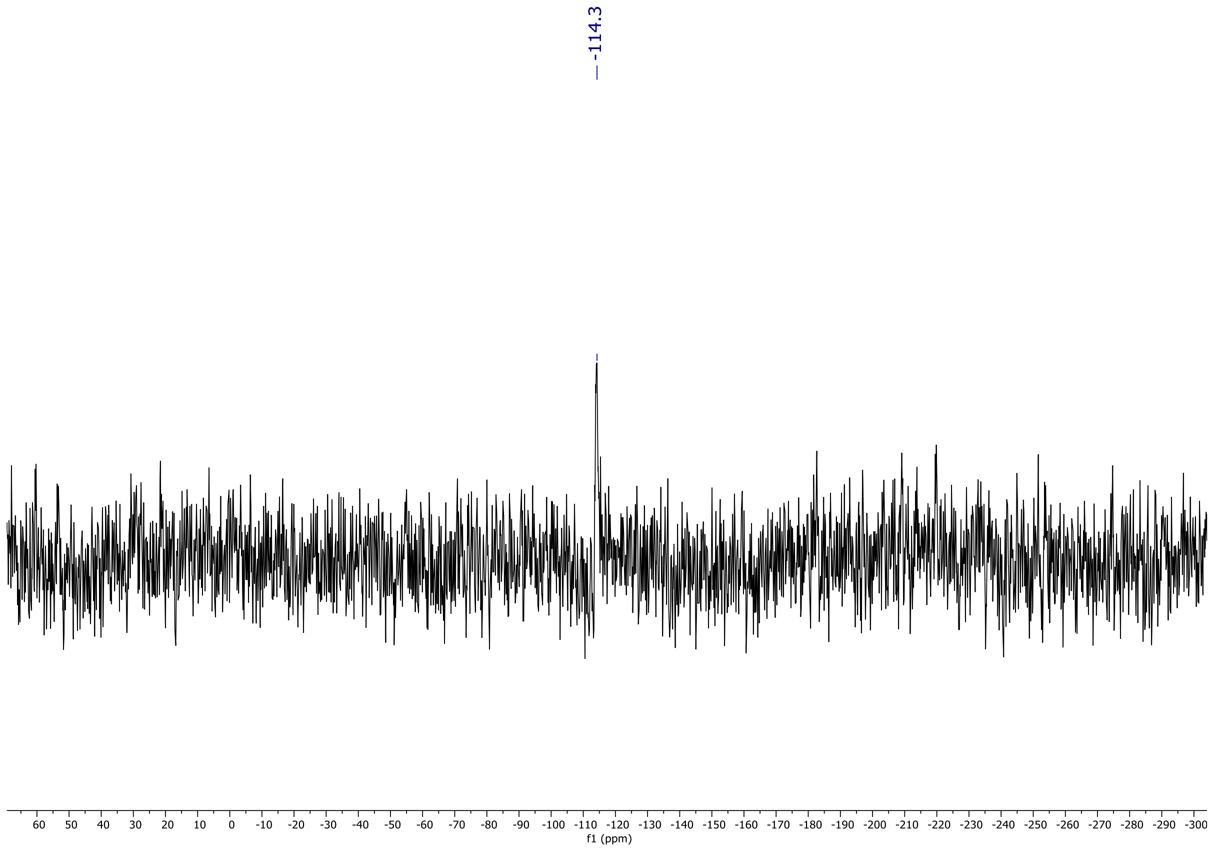


**Figure S38.** The $^{119}Sn$ NMR spectrum (186 MHz, $C_6D_6$) of $Ph_3SnOtBu$.

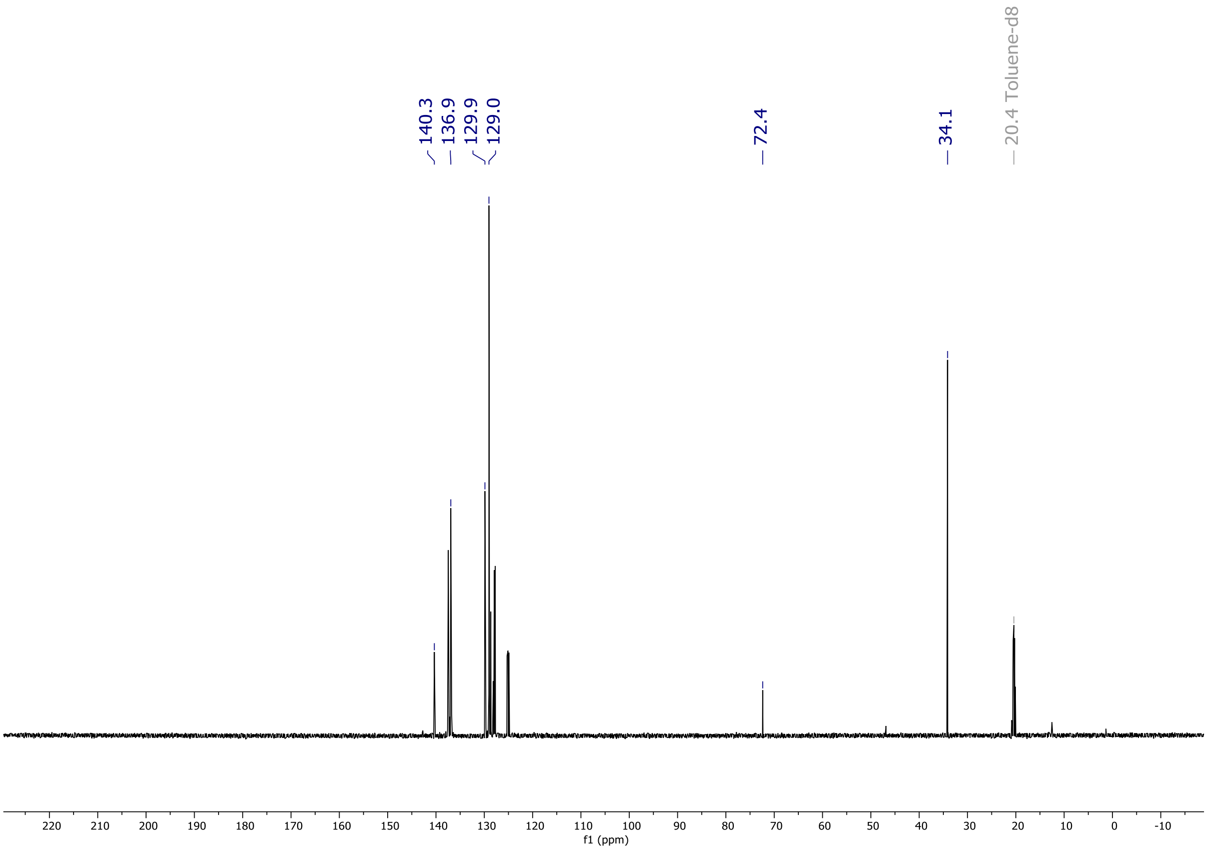


**Figure S39.** $^{13}C\{^{1}H\}$ NMR spectrum (126 MHz, $C_6D_6$) of $Ph_3SnOtBu$.

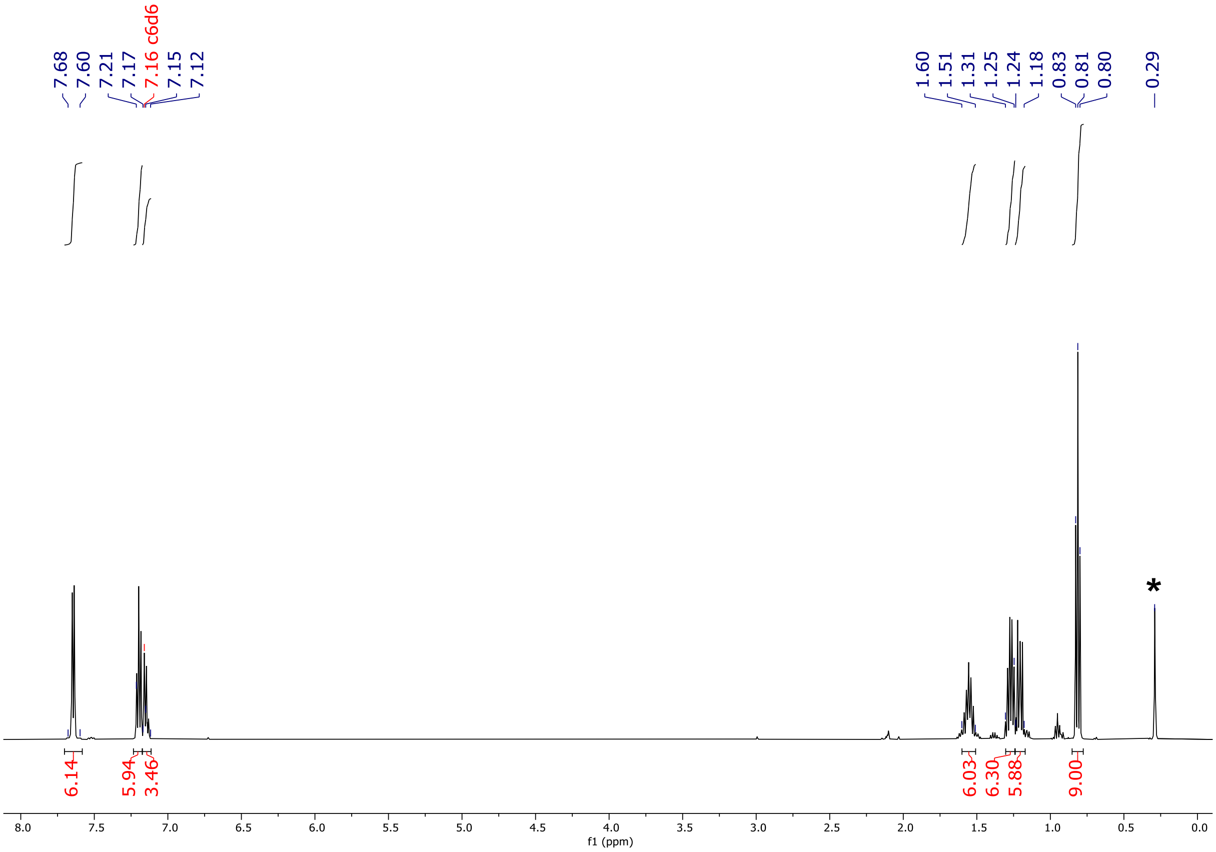


**Figure S40.** $^{1}H$ NMR spectrum (500 MHz, $C_6D_6$) of $Ph_3GeSnBu_3$. * = residual grease.[4]

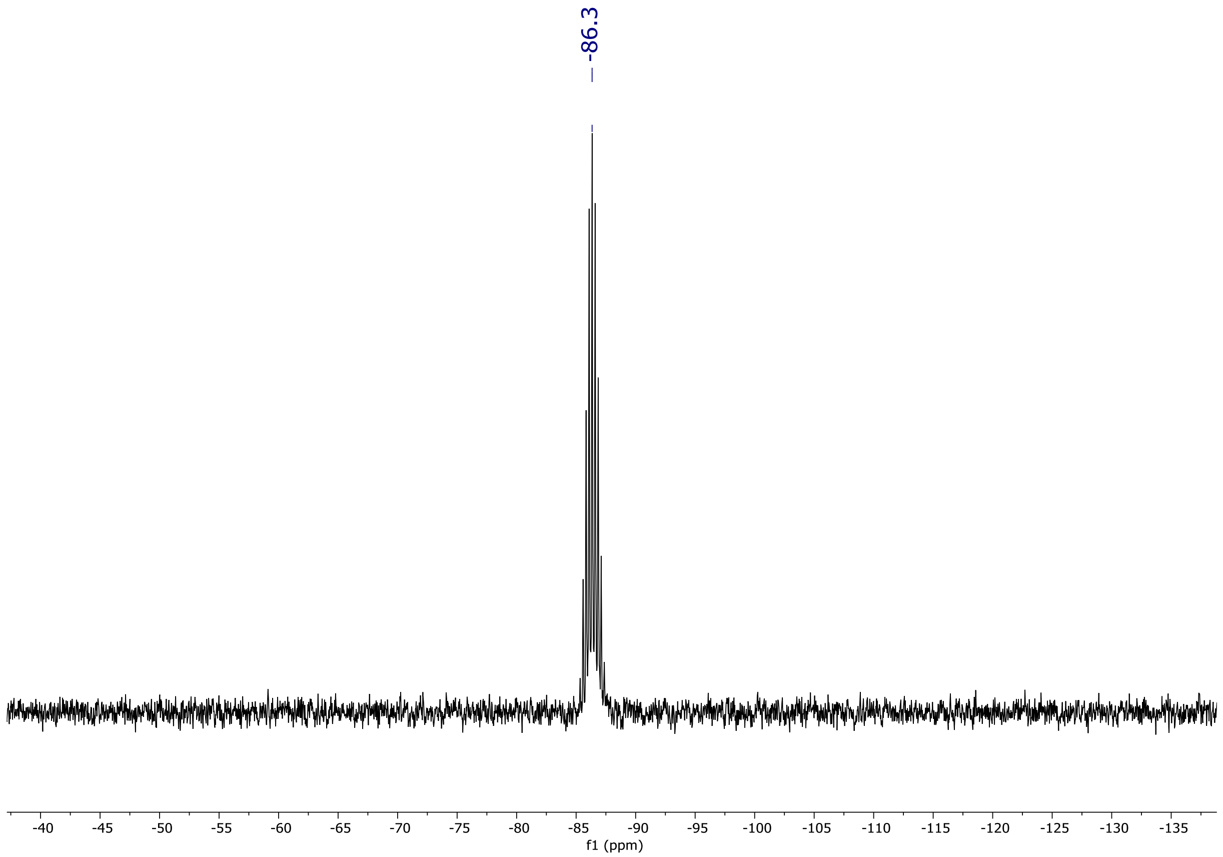


**Figure S41.** The $^{119}Sn$ NMR spectrum (186 MHz, $C_6D_6$) of $Ph_3GeSnBu_3$.

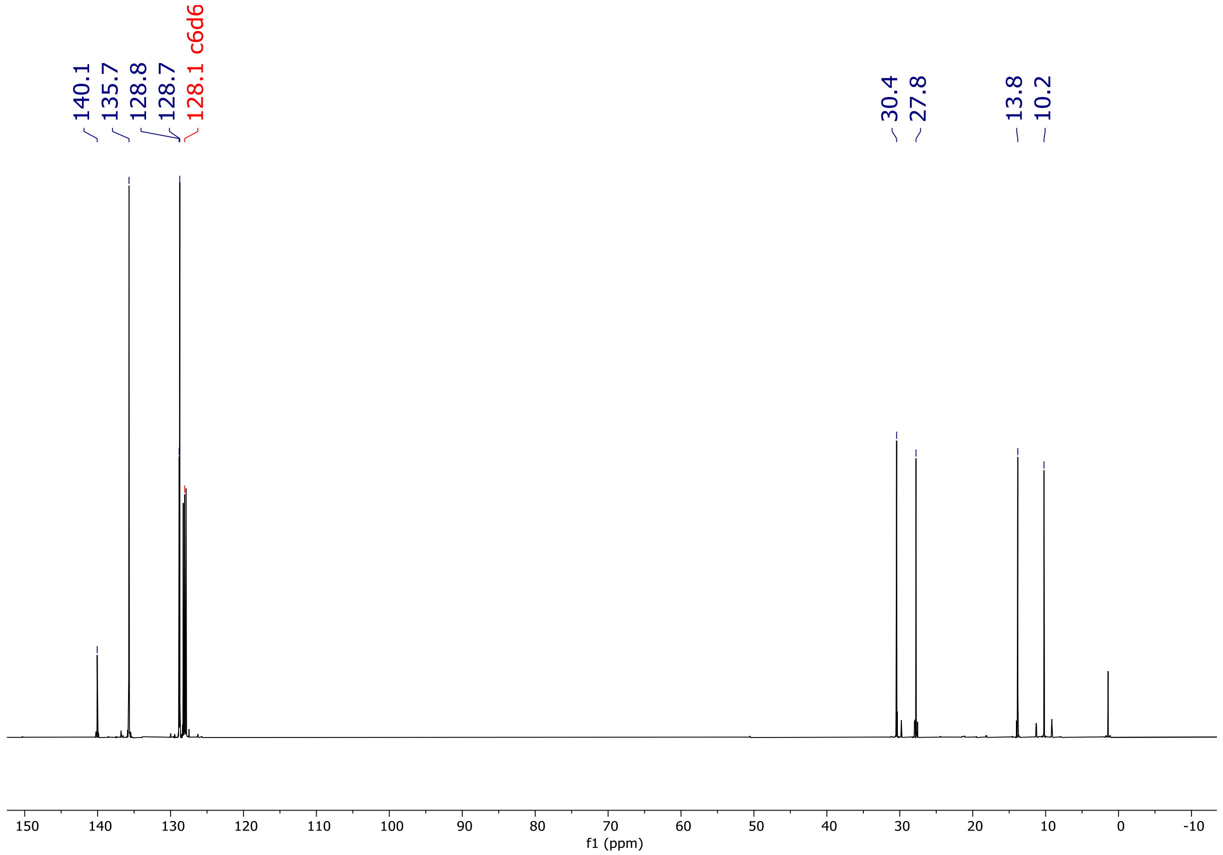


**Figure S42.** $^{13}C\{^1H\}$ NMR spectrum (126 MHz, $C_6D_6$) of $Ph_3GeSnBu_3$.

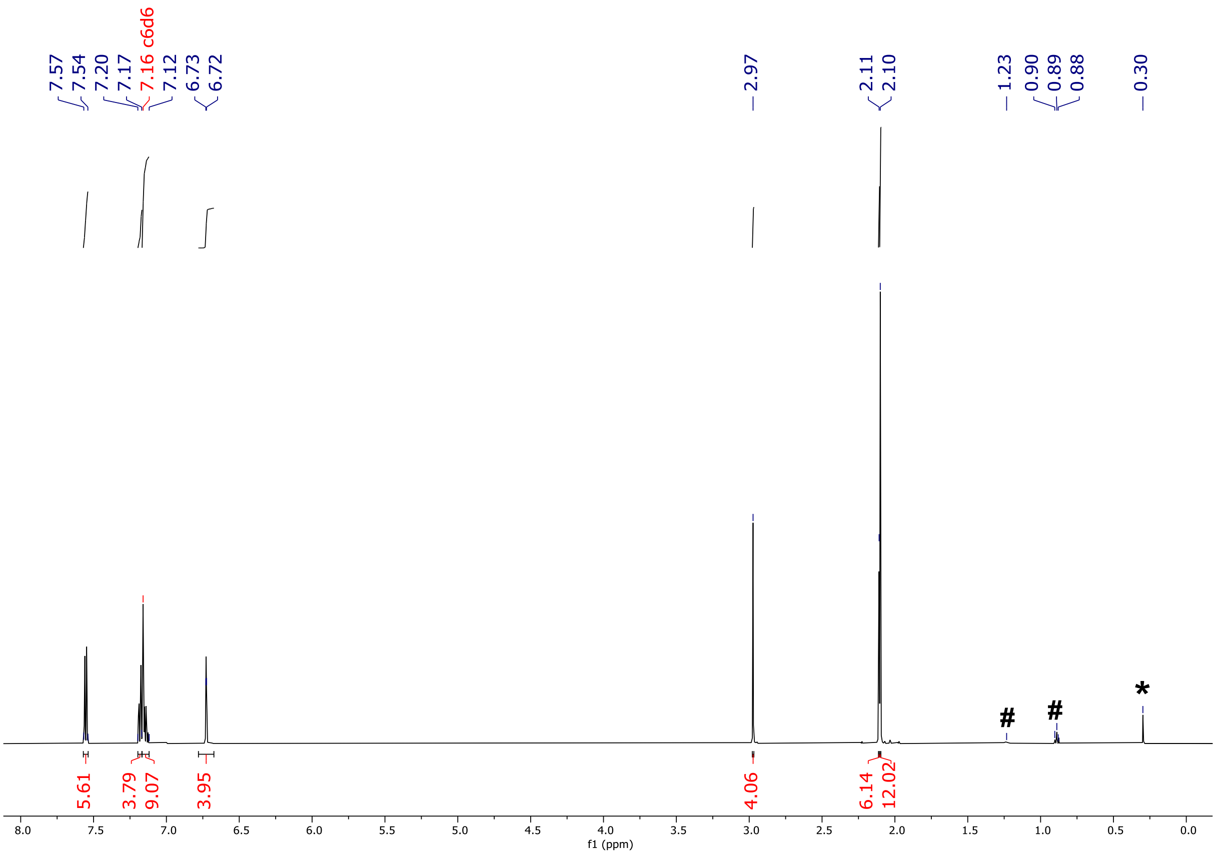


**Figure S43.** $^1$H NMR spectrum (500 MHz, $C_6D_6$) of compound **1**, (SIMes)CuGePh$_3$. # = residual hexane.[4] * = residual grease.[4]

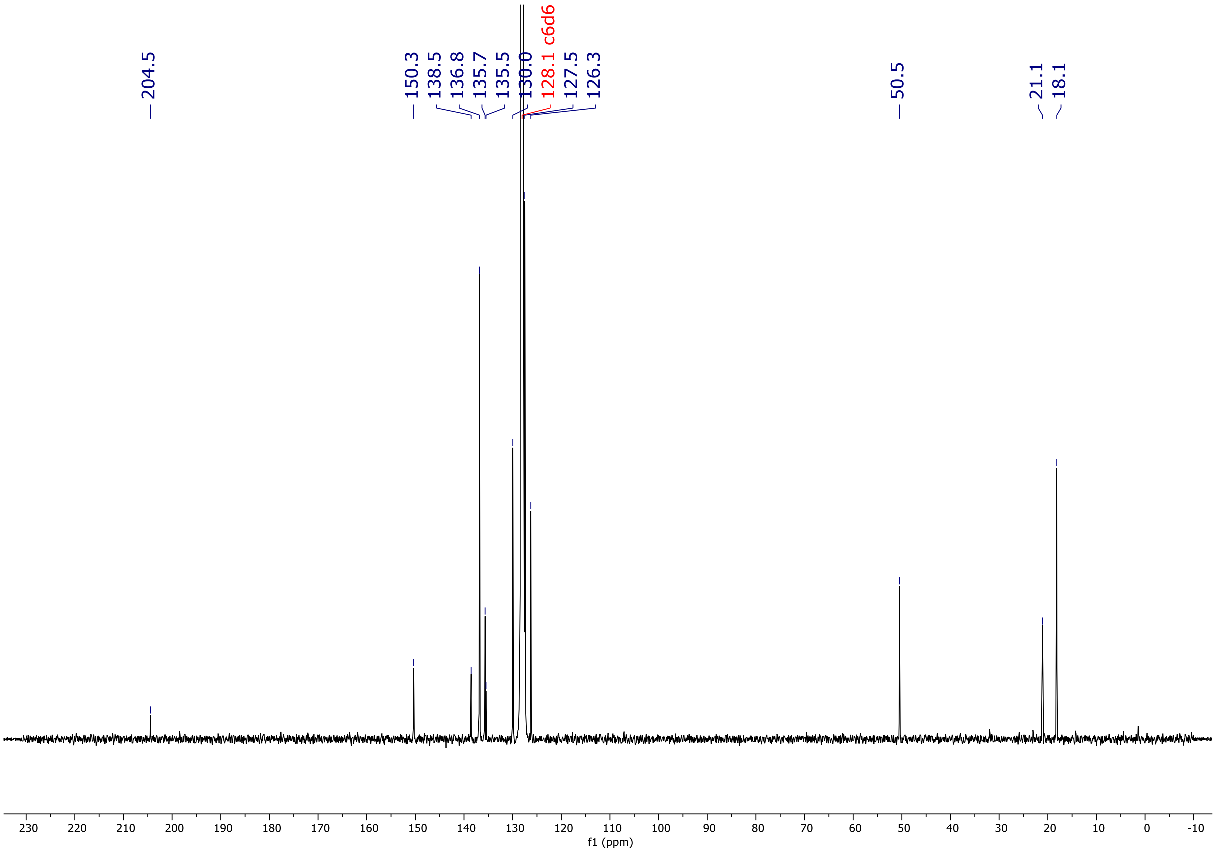


**Figure S44.** $^{13}C\{^1H\}$ NMR spectrum (126 MHz, $C_6D_6$) of compound **1**, (SIMes)CuGePh$_3$.

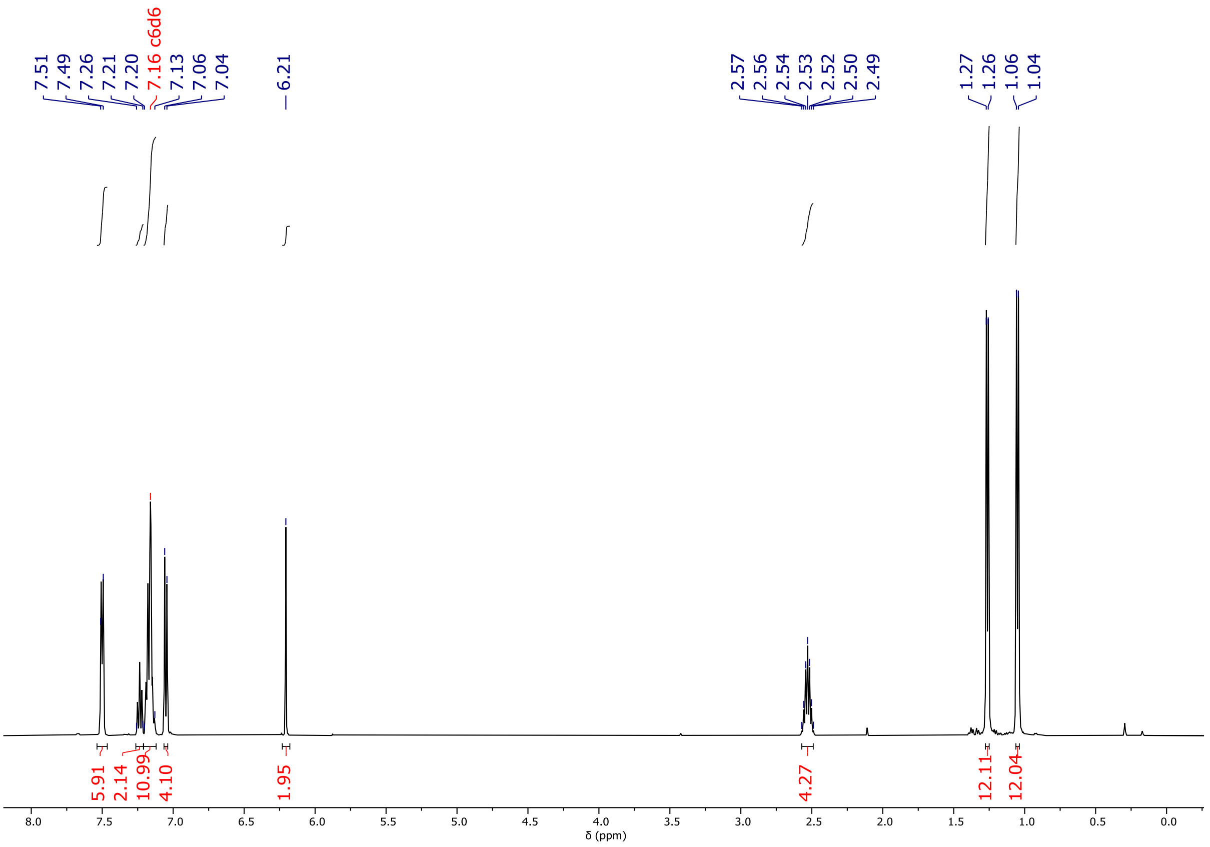


**Figure S45.** $^{1}$H NMR spectrum (500 MHz, $C_6D_6$) of compound **2**, (IPr)CuGePh$_3$.

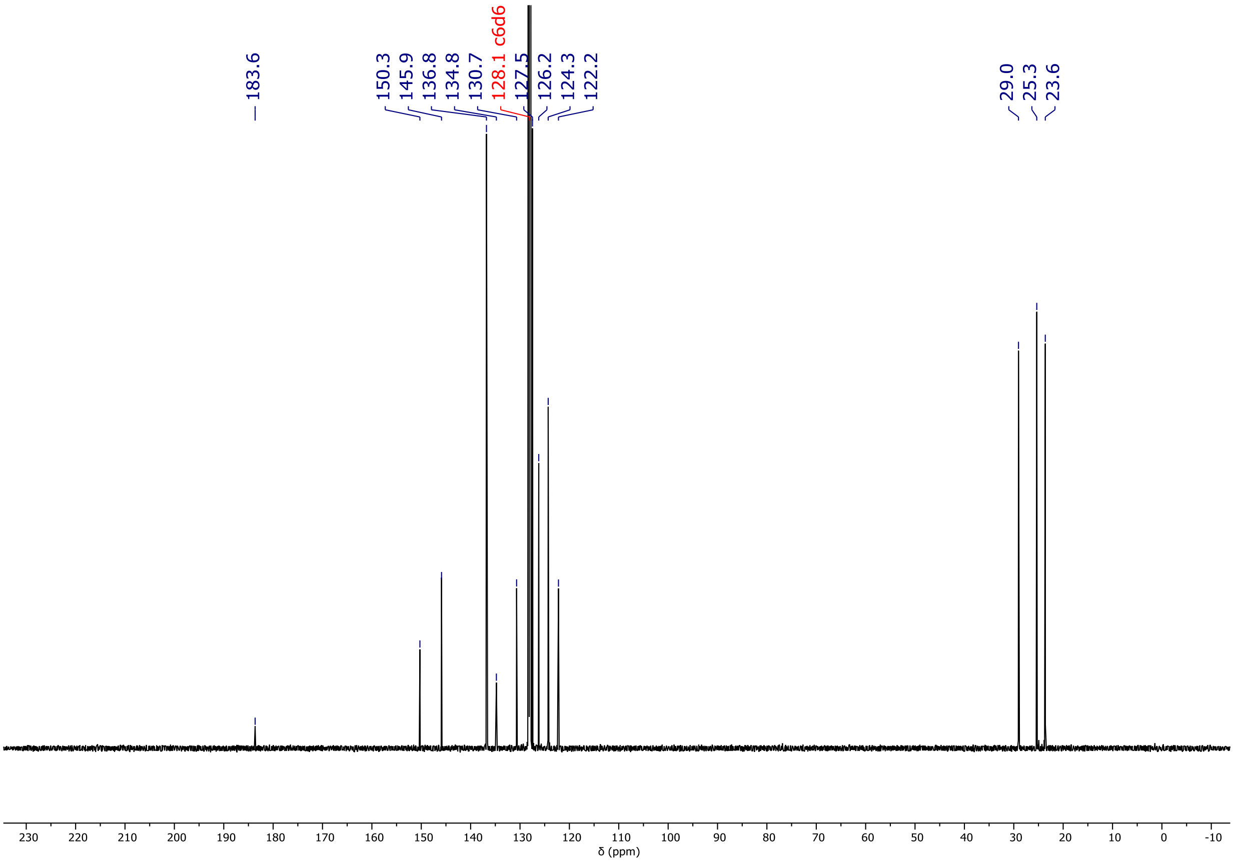


**Figure S46.** $^{13}$C{$^{1}$H} NMR spectrum (126 MHz, $C_6D_6$) of compound **2**, (IPr)CuGePh$_3$.

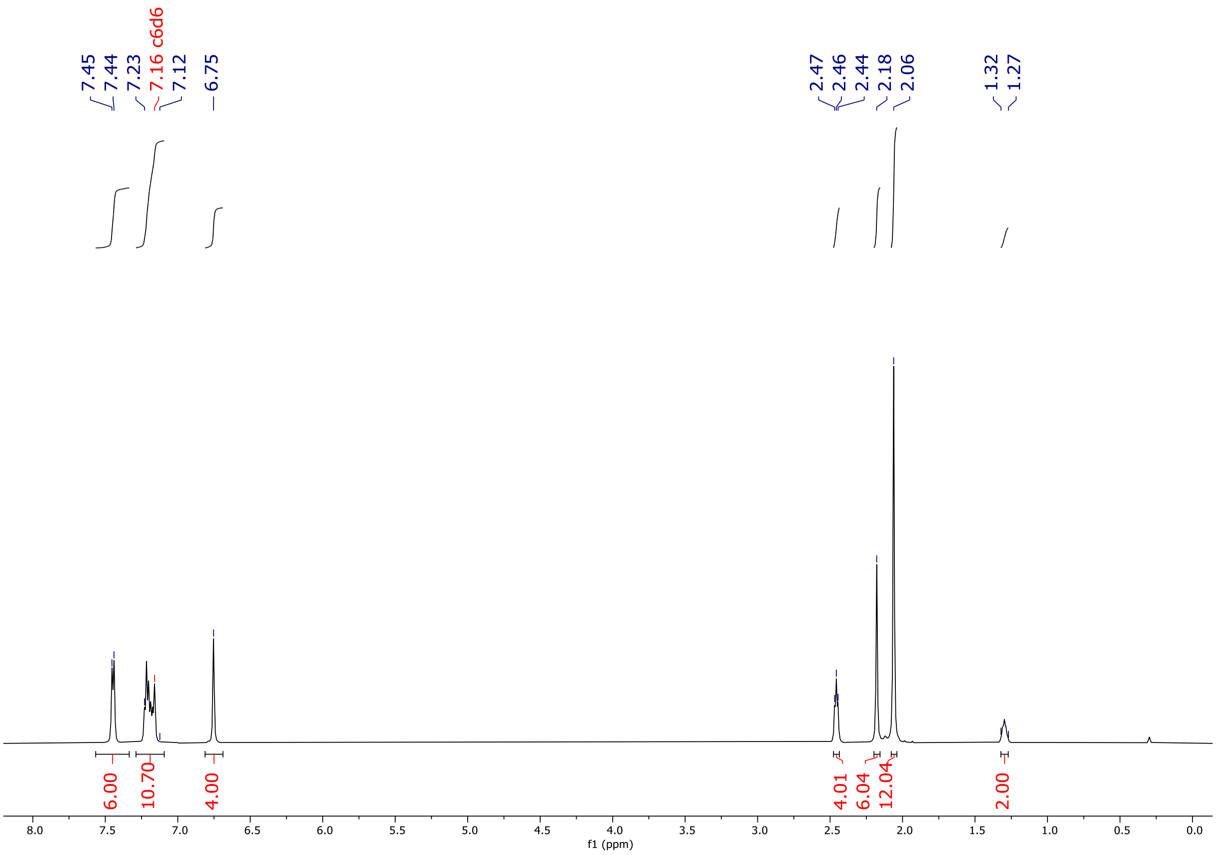


**Figure S47.** $^{1}H$ NMR spectrum (500 MHz, $C_6D_6$) of compound **3**, (6-Mes)CuGePh₃.

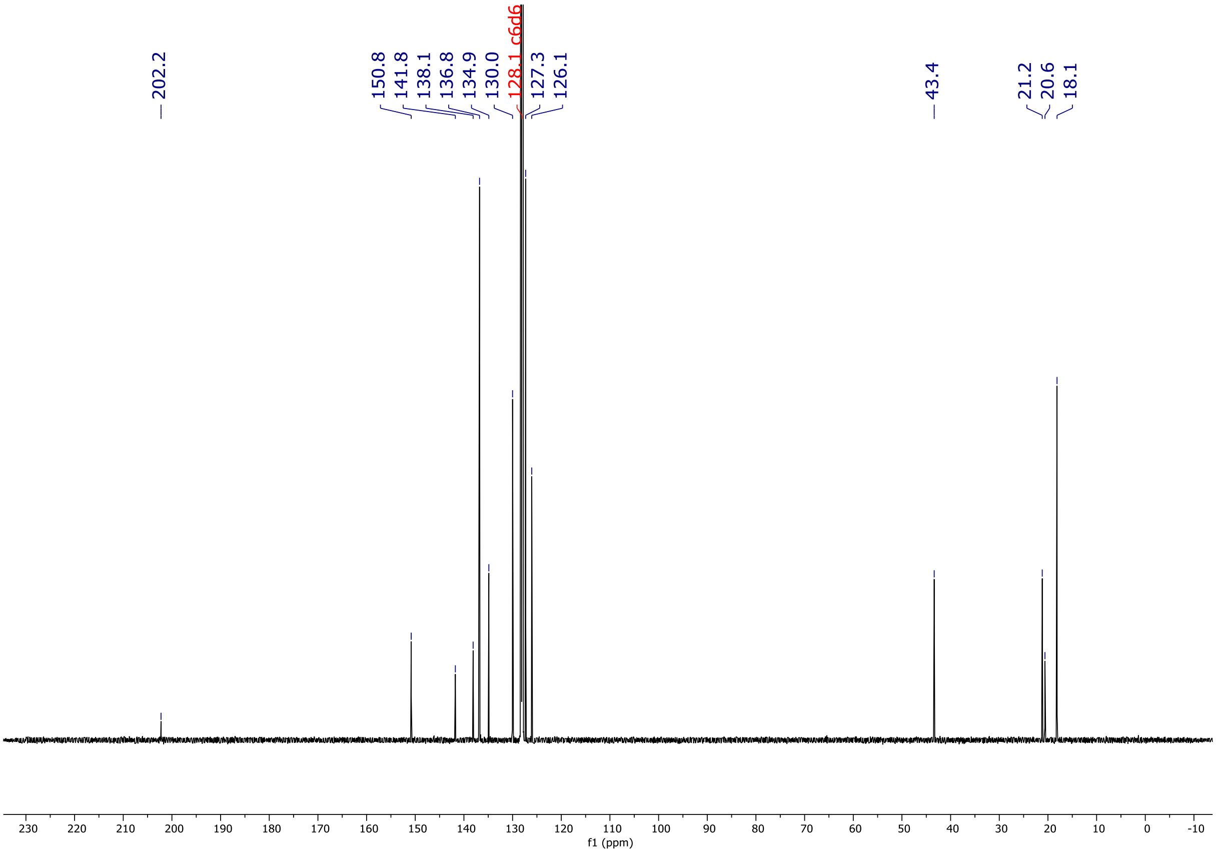


**Figure S48.** $^{13}C\{^{1}H\}$ NMR spectrum (126 MHz, $C_6D_6$) of compound **3**, (6-Mes)CuGe$Ph_3$.

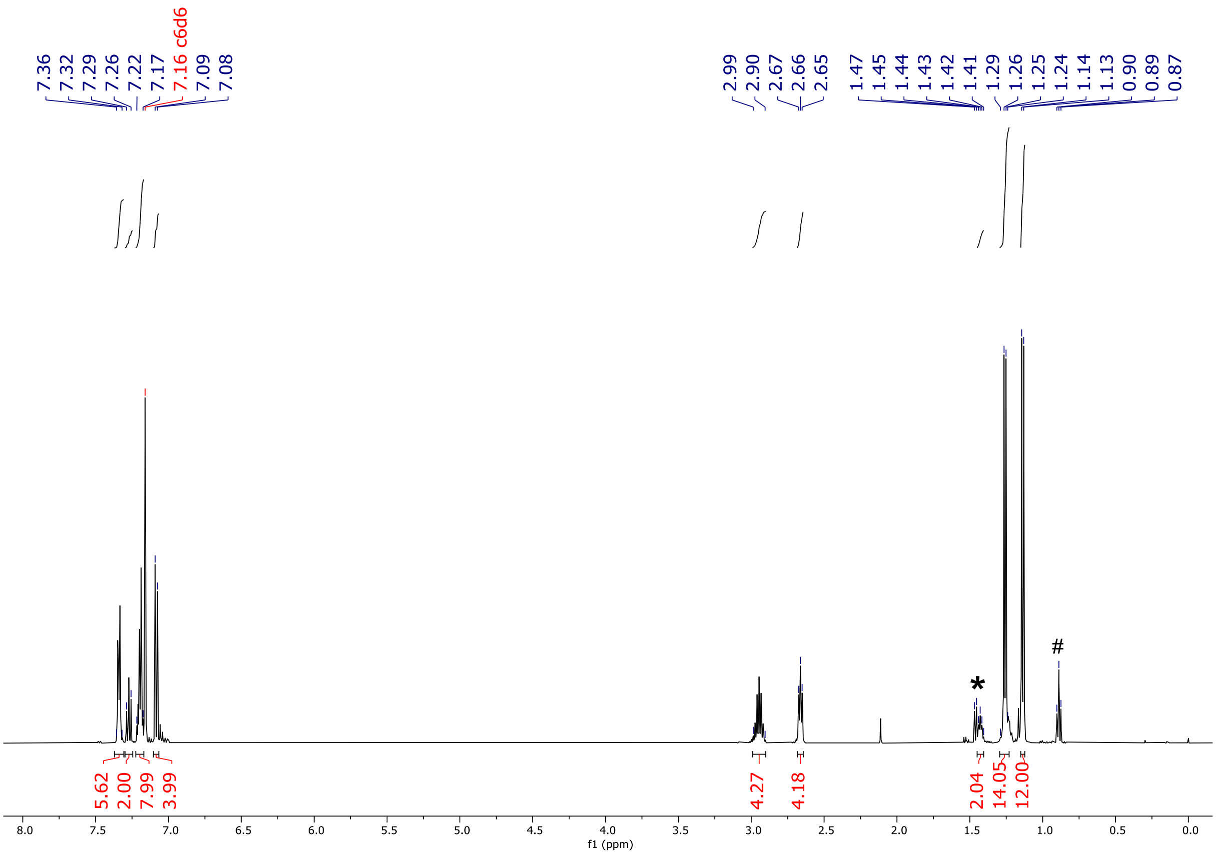


**Figure S49.** $^{1}H$ NMR spectrum (500 MHz, $C_6D_6$) of compound **4**, (6-Dipp)CuGePh$_3$. * = 5 % impurity of (6-Dipp)CuCl.[15] # = residual hexane.[4]

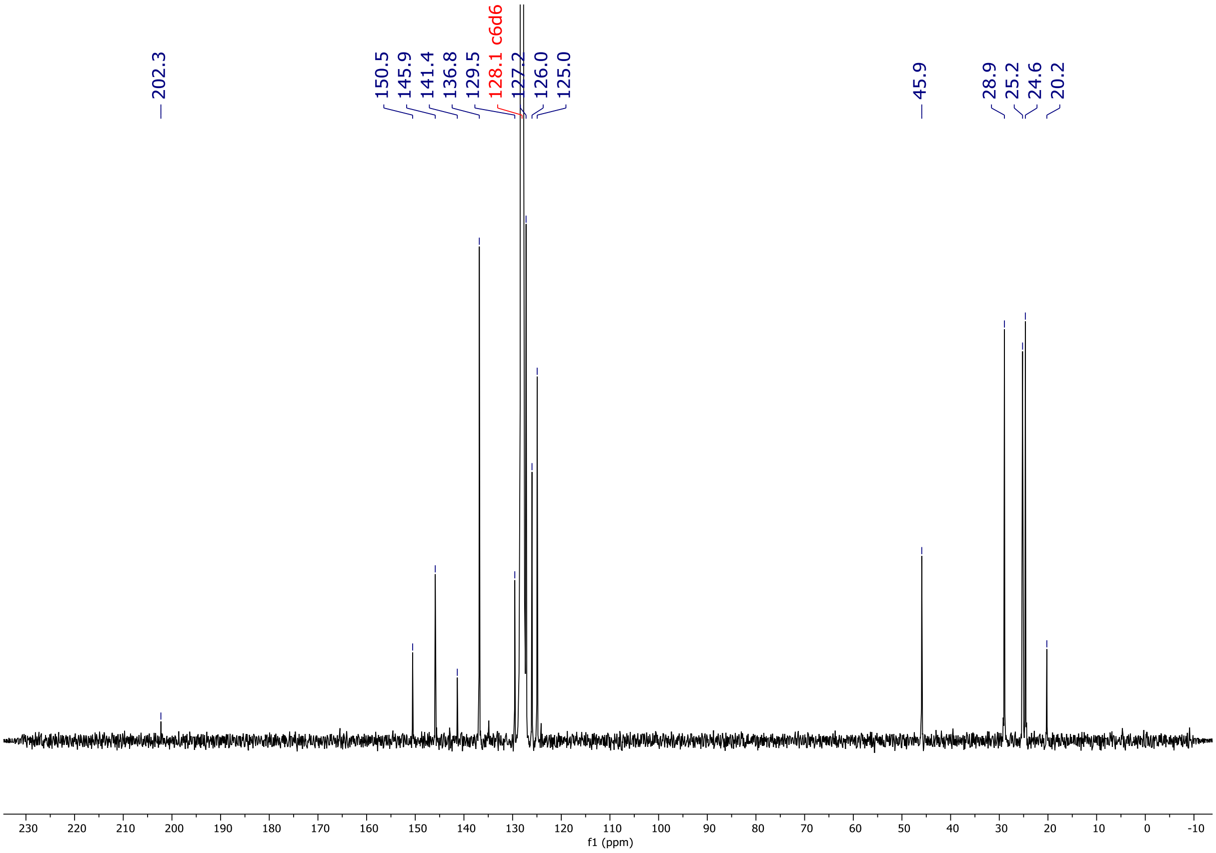


**Figure S50.** $^{13}C\{^{1}H\}$ NMR spectrum (126 MHz, $C_6D_6$) of compound **4**, (6-Dipp)CuGePh$_3$.

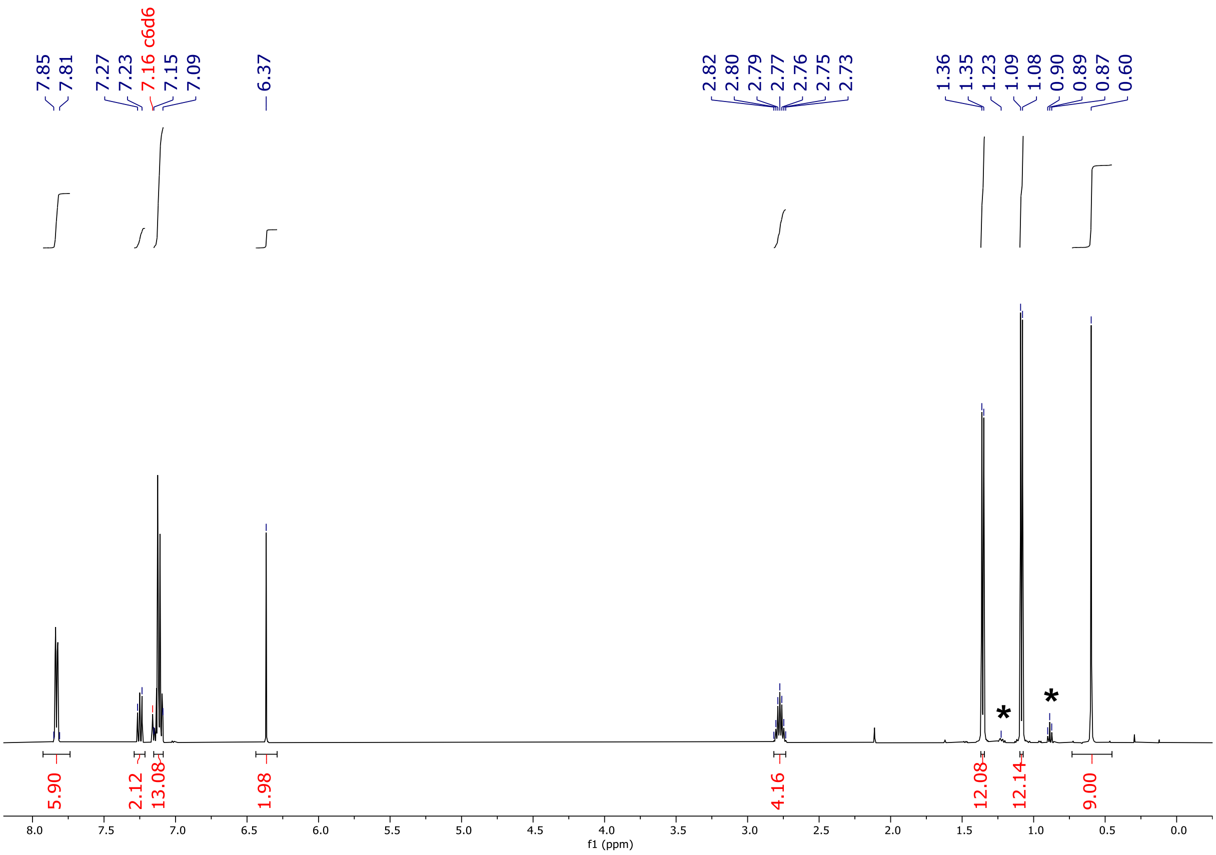


**Figure S51.** $^{1}H$ NMR spectrum (500 MHz, $C_6D_6$) of compound **5**, (IPr)CuSC(=NtBu)GePh$_3$. * = residual hexane.[4]

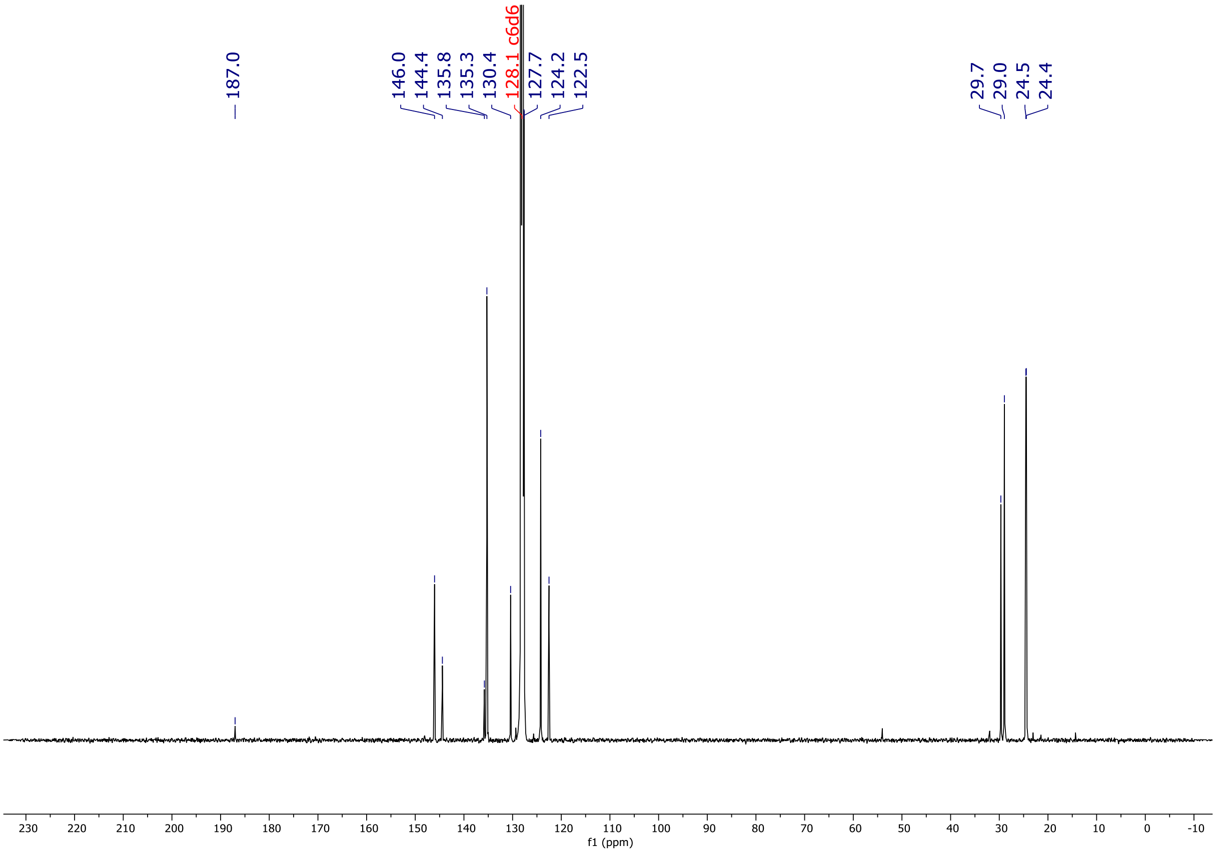


**Figure S52.** $^{13}C\{^{1}H\}$ NMR spectrum (126 MHz, $C_6D_6$) of compound **5**, (IPr)CuSC(=NtBu)GePh$_3$.

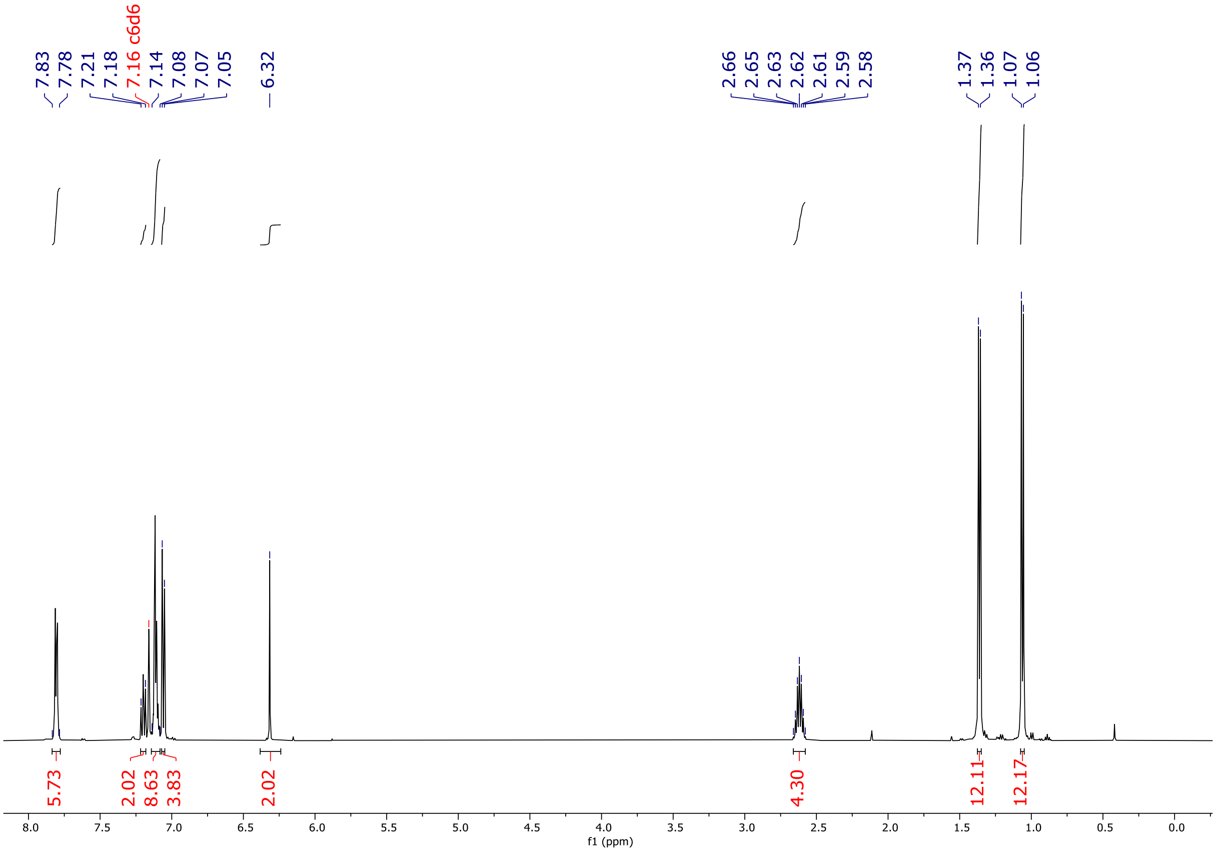


**Figure S53.** $^{1}H$ NMR spectrum (500 MHz, $C_6D_6$) of compound **6**, (IPr)CuSC(S)GePh$_3$.

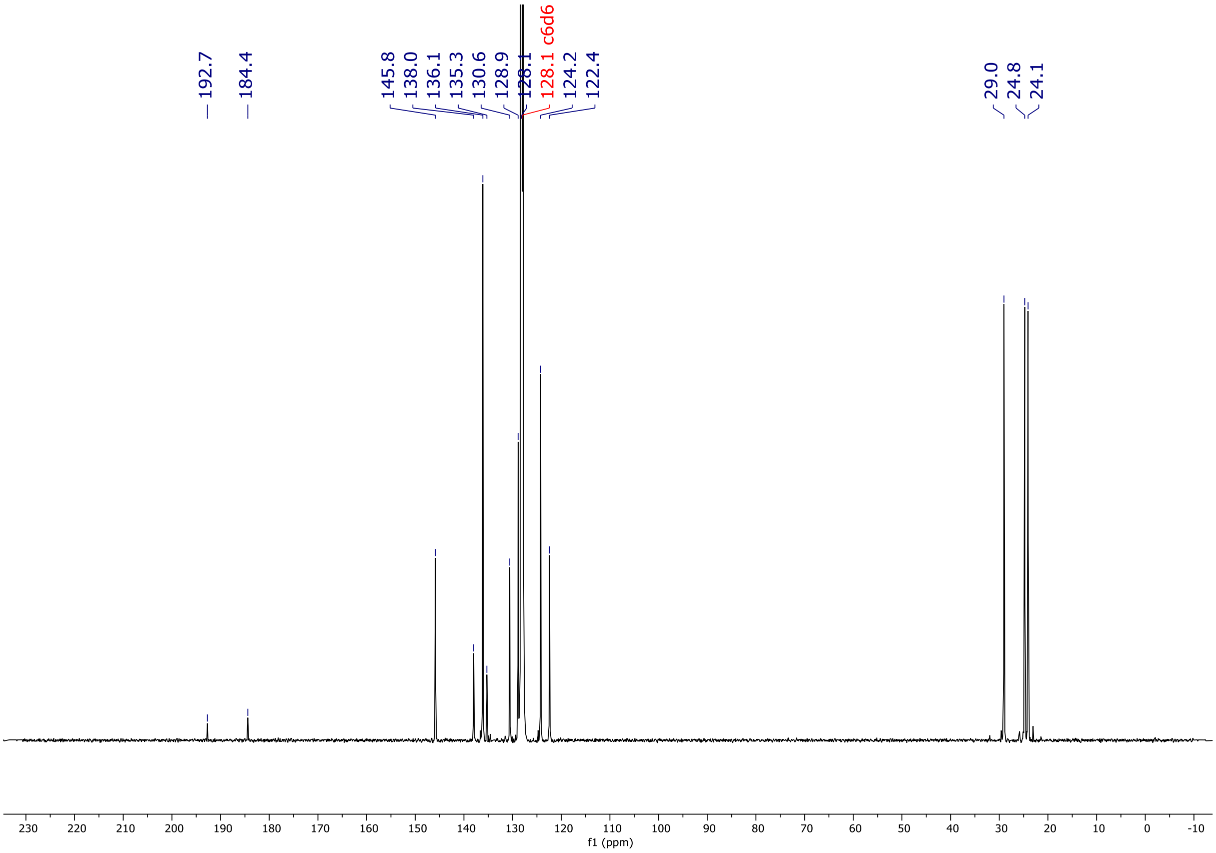


**Figure S54.** $^{13}C\{^{1}H\}$ NMR spectrum (126 MHz, $C_6D_6$) of compound **6**, (IPr)CuSC(S)GePh$_3$.

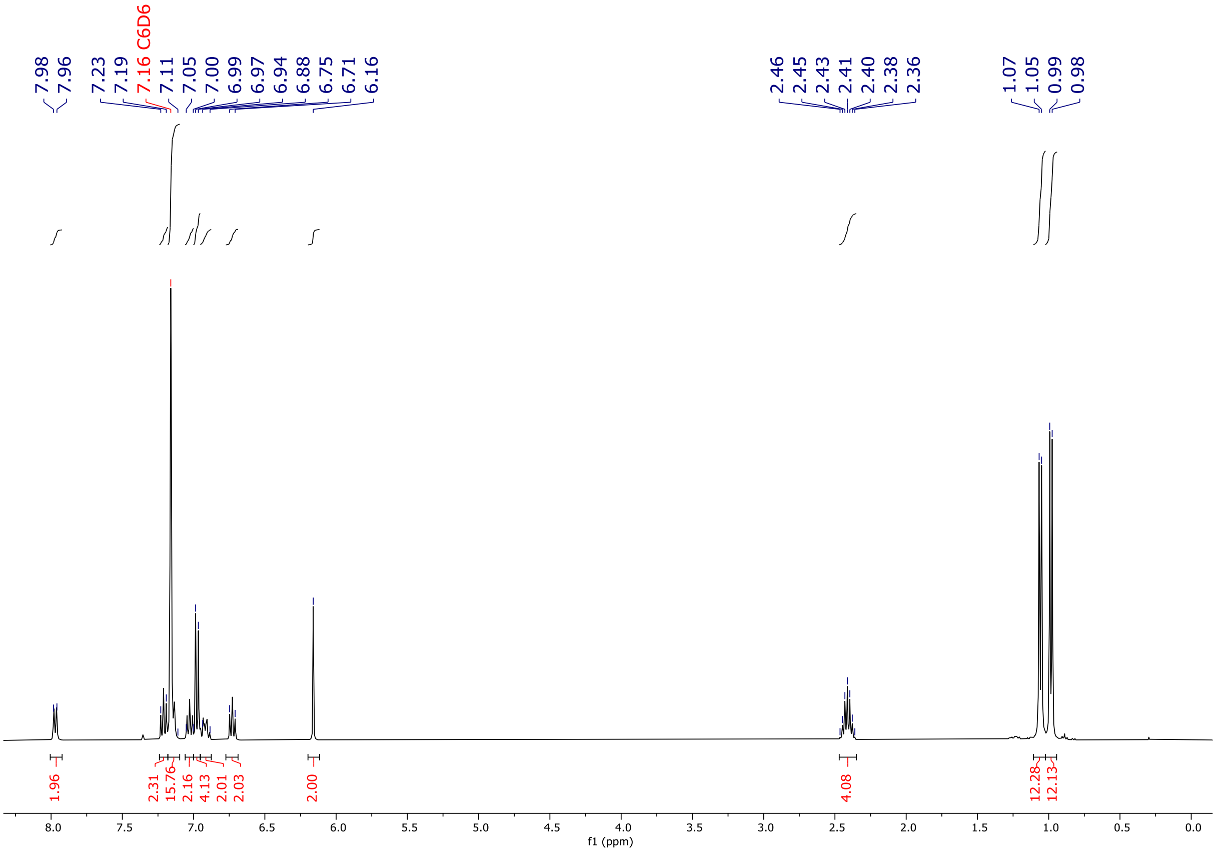

**Figure S55.** $^{1}H$ NMR spectrum (400 MHz, $C_6D_6$) of compound **7**, (IPr)CuN(Ph)C(O)Ph.

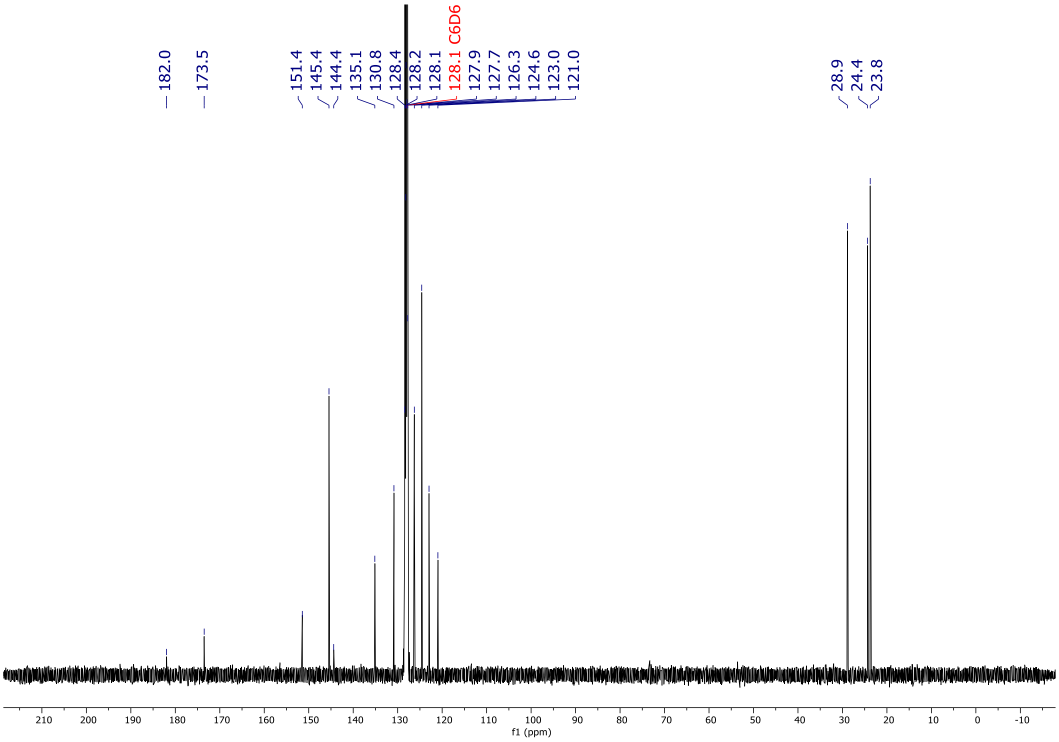

**Figure S56.** $^{13}C\{^{1}H\}$ NMR spectrum (101 MHz, $C_6D_6$) of compound **7**, (IPr)CuN(Ph)C(O)Ph.

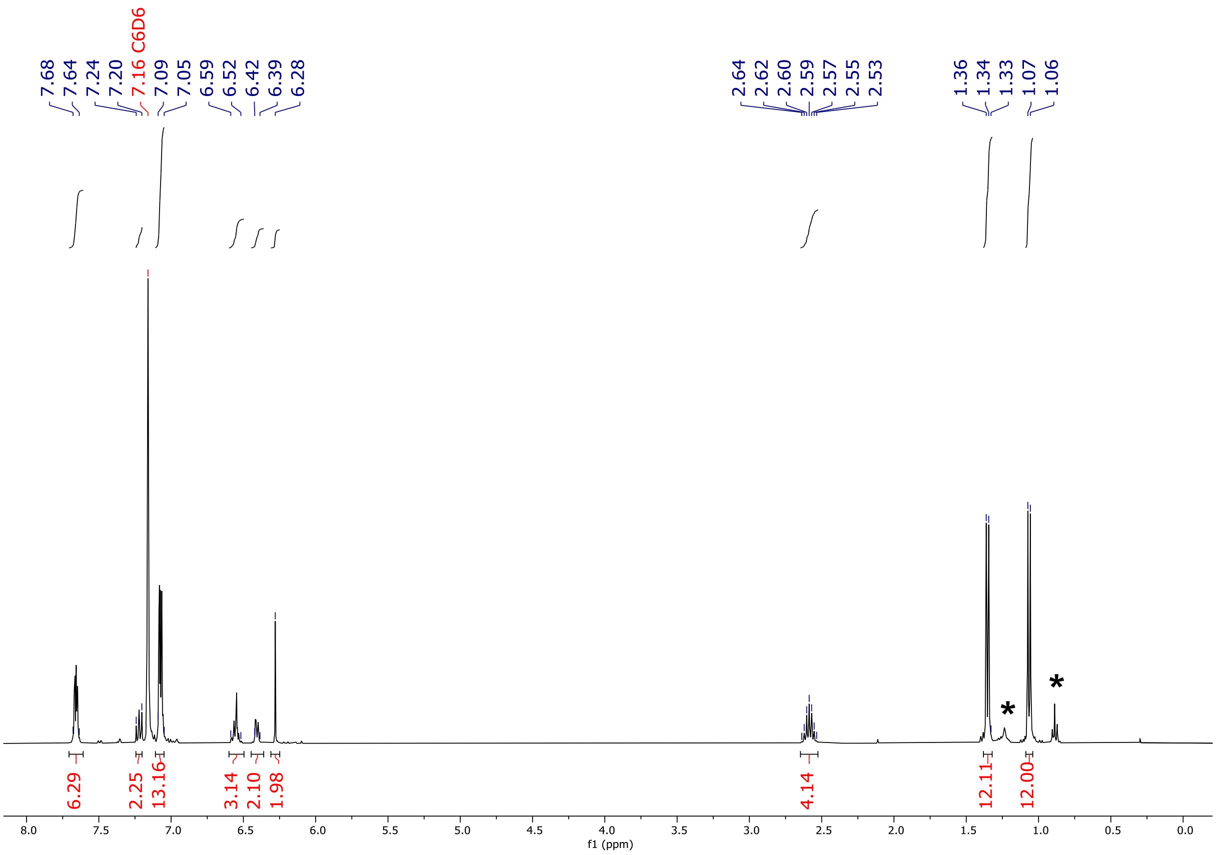


**Figure S57.** $^{1}H$ NMR spectrum (400 MHz, $C_6D_6$) of compound **8**, (IPr)CuN(Ph)C(O)GePh$_3$. * = residual hexane.[4]

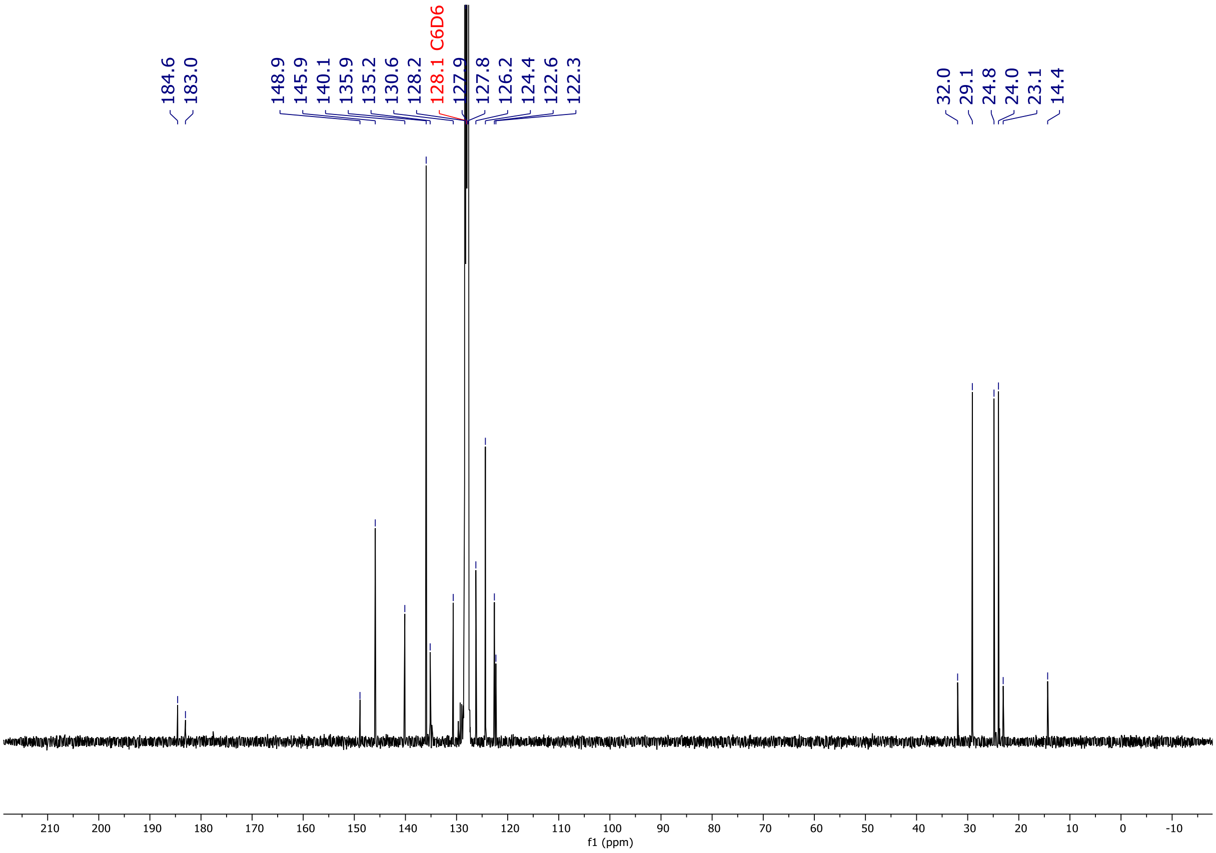


**Figure S58.** $^{13}C\{^{1}H\}$ NMR spectrum (101 MHz, $C_6D_6$) of compound **8**, (IPr)CuN(Ph)C(O)GePh$_3$.

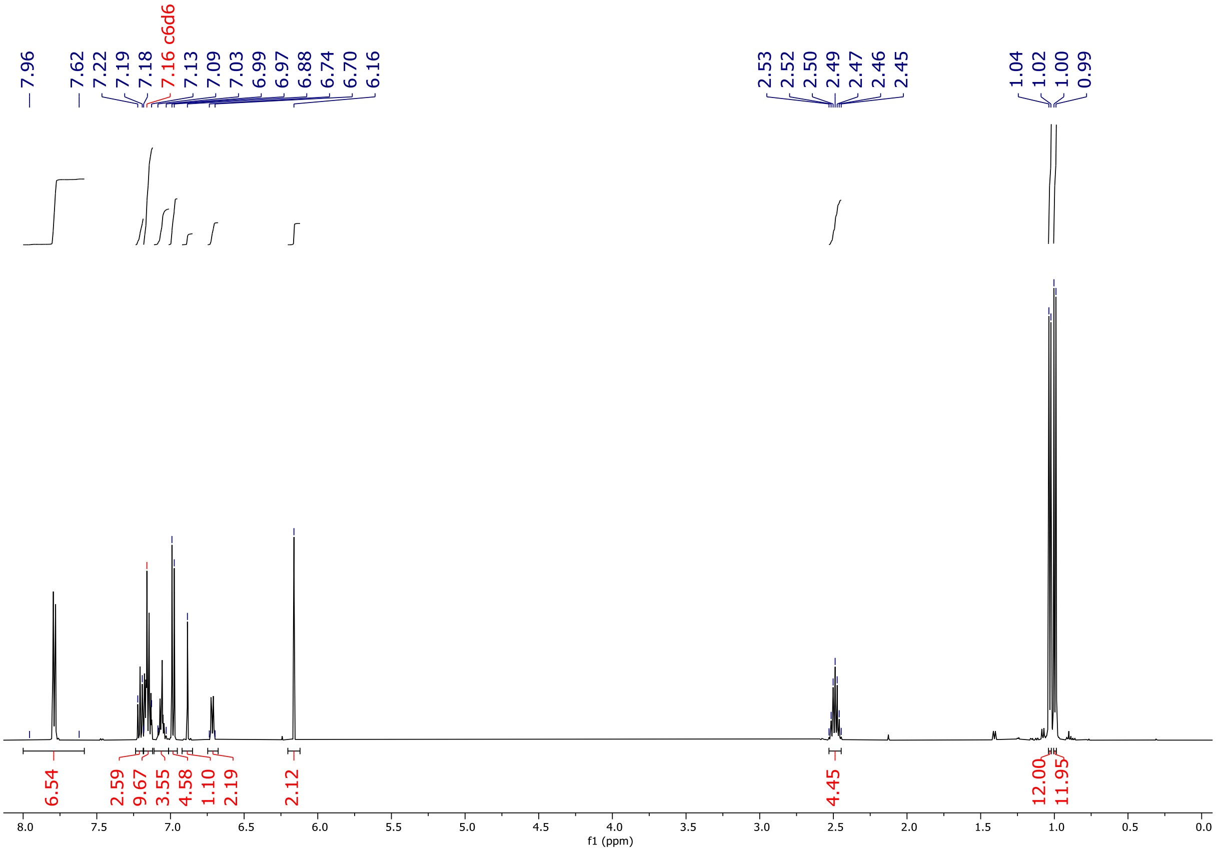


**Figure S59.** $^{1}H$ NMR spectrum (500 MHz, $C_6D_6$) of compound **9**, (IPr)CuC(Ph)C(H)GePh$_3$.

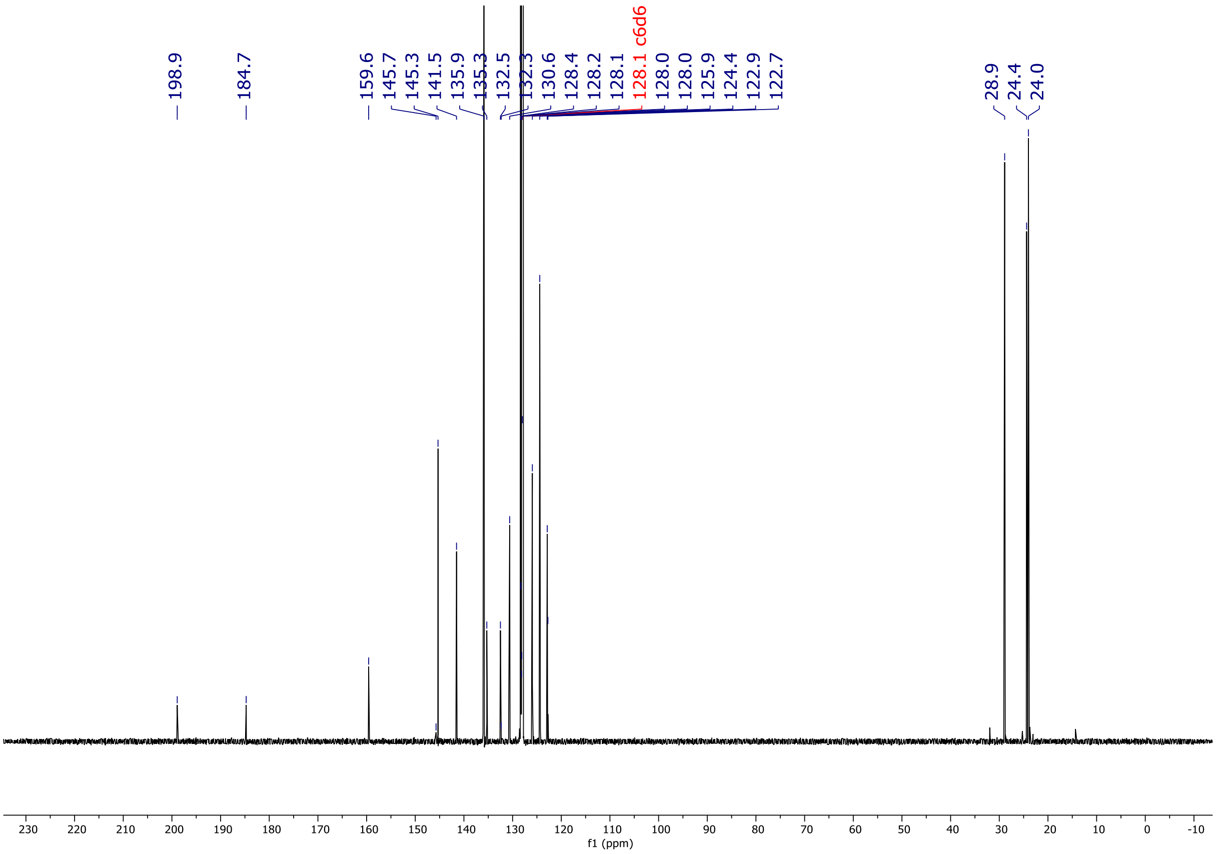


**Figure S60.** $^{13}C\{^{1}H\}$ NMR spectrum (126 MHz, $C_6D_6$) of compound **9**, (IPr)CuC(Ph)C(H)GePh$_3$.

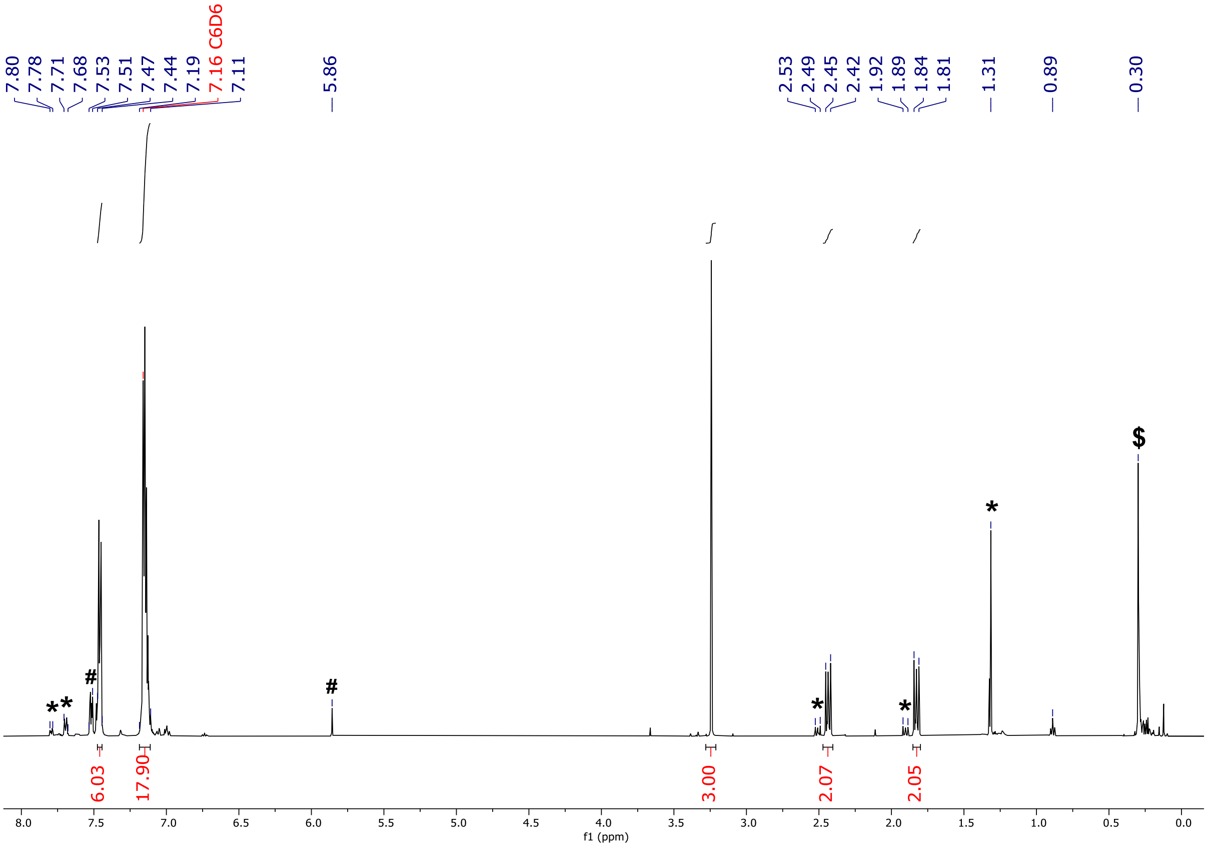


**Figure S61.** $^{1}H$ NMR spectrum (500 MHz, $C_6D_6$) of compound **11a**. # = Triphenylgermane; * = *ca*. 15 mol % contaminated with **11c** from competing transesterification reaction of **11a**; $ = grease.

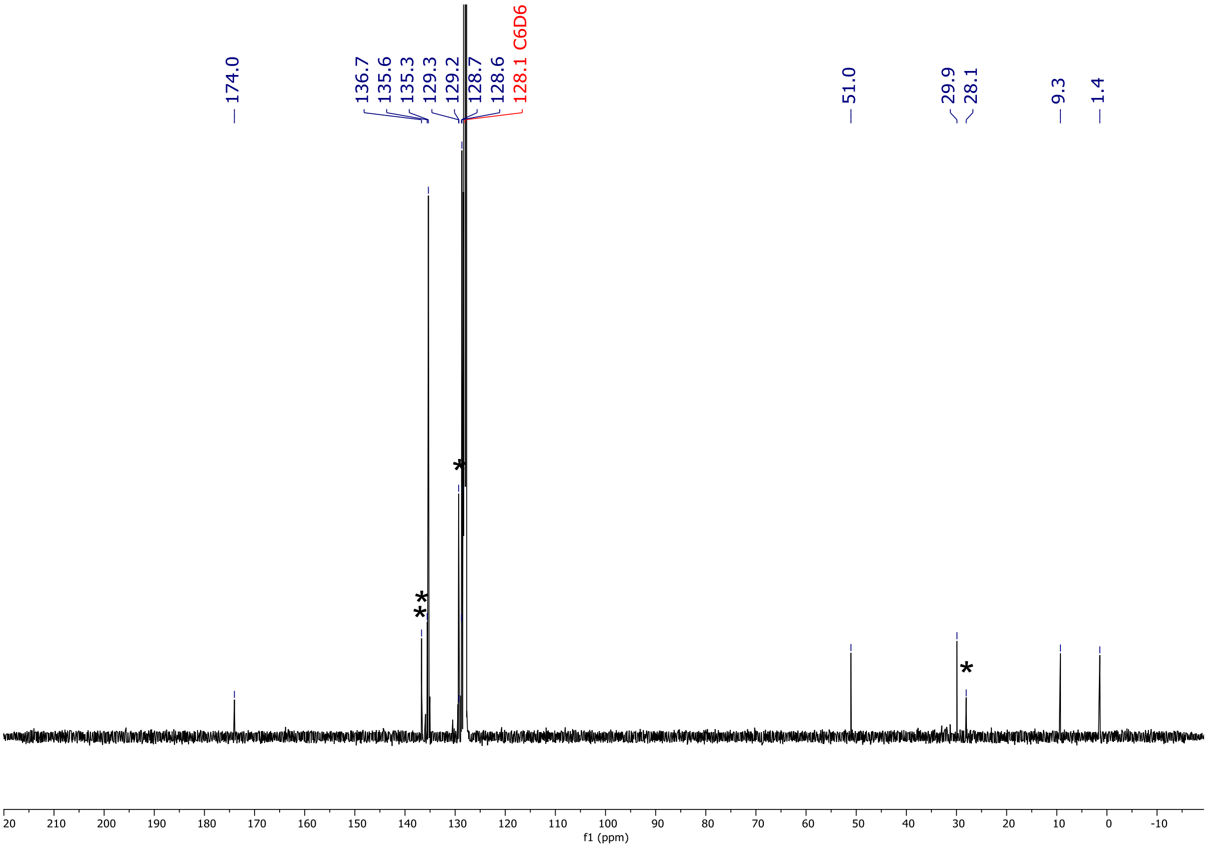


**Figure S62.** $^{13}C\{^{1}H\}$ NMR spectrum (126 MHz, $C_6D_6$) of compound **11a**, * = *ca*. 15 mol % contaminated with **11 c** from competing transesterification reaction of **11a**.

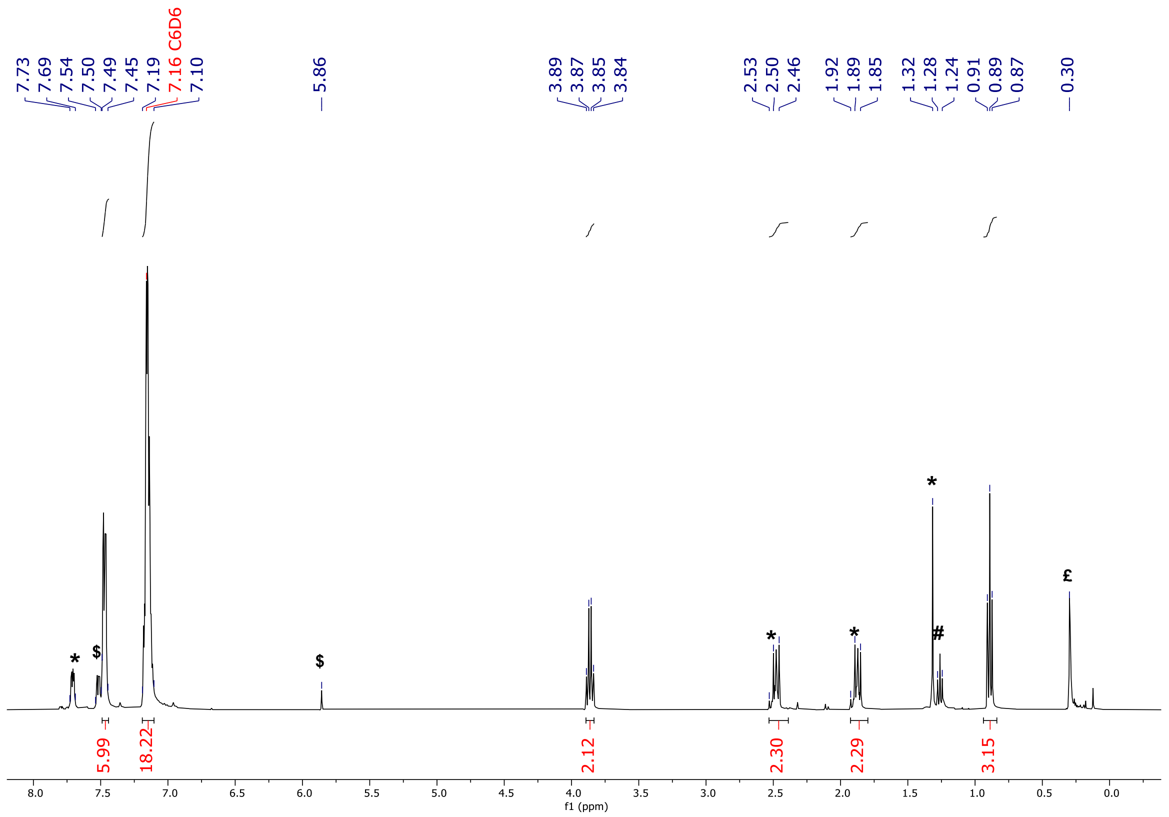


**Figure S63.** $^{1}$H NMR spectrum (400 MHz, $C_6D_6$) of compound **11b**. $ = Triphenylgermane; * *ca*. 11 mol % contaminated with **11c** from competing transesterification reaction of **11b**; # = unknown; $ = $HGePh_3$; £ = grease.

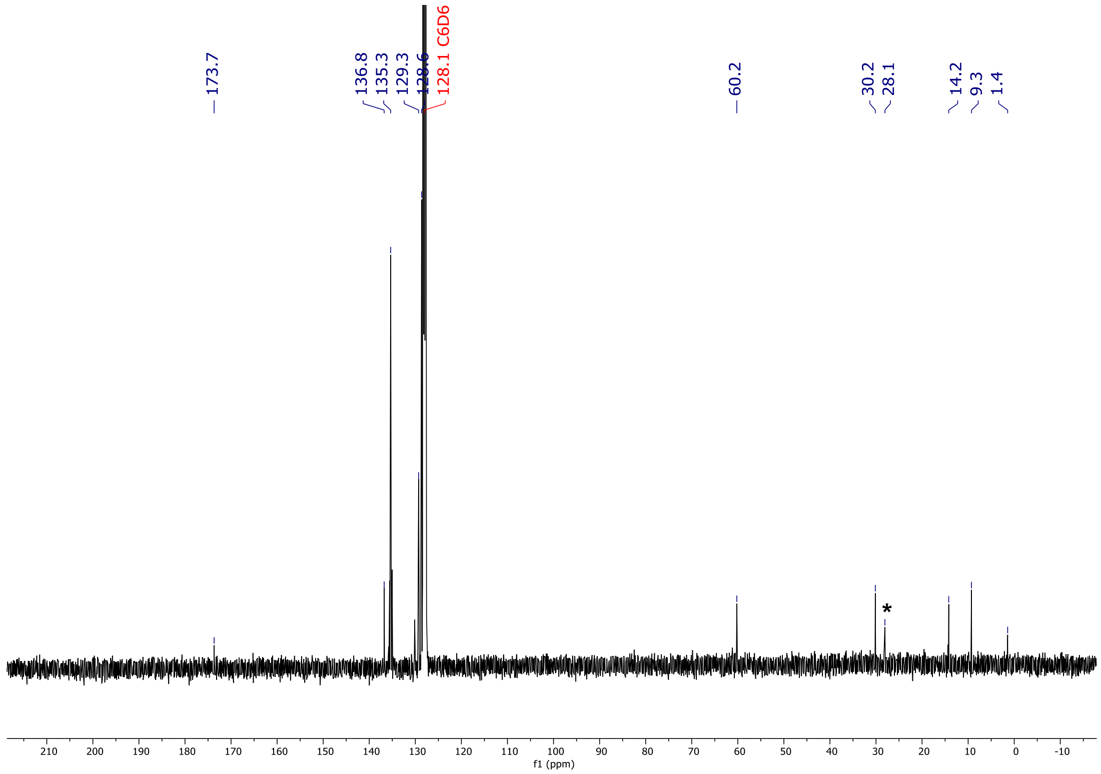


**Figure S64.** $^{13}C\{^{1}H\}$ NMR spectrum (101 MHz, $C_6D_6$) of compound **11b**, * = *ca*. 11 mol % contaminated with **11 c** from competing transesterification reaction of **11b**.

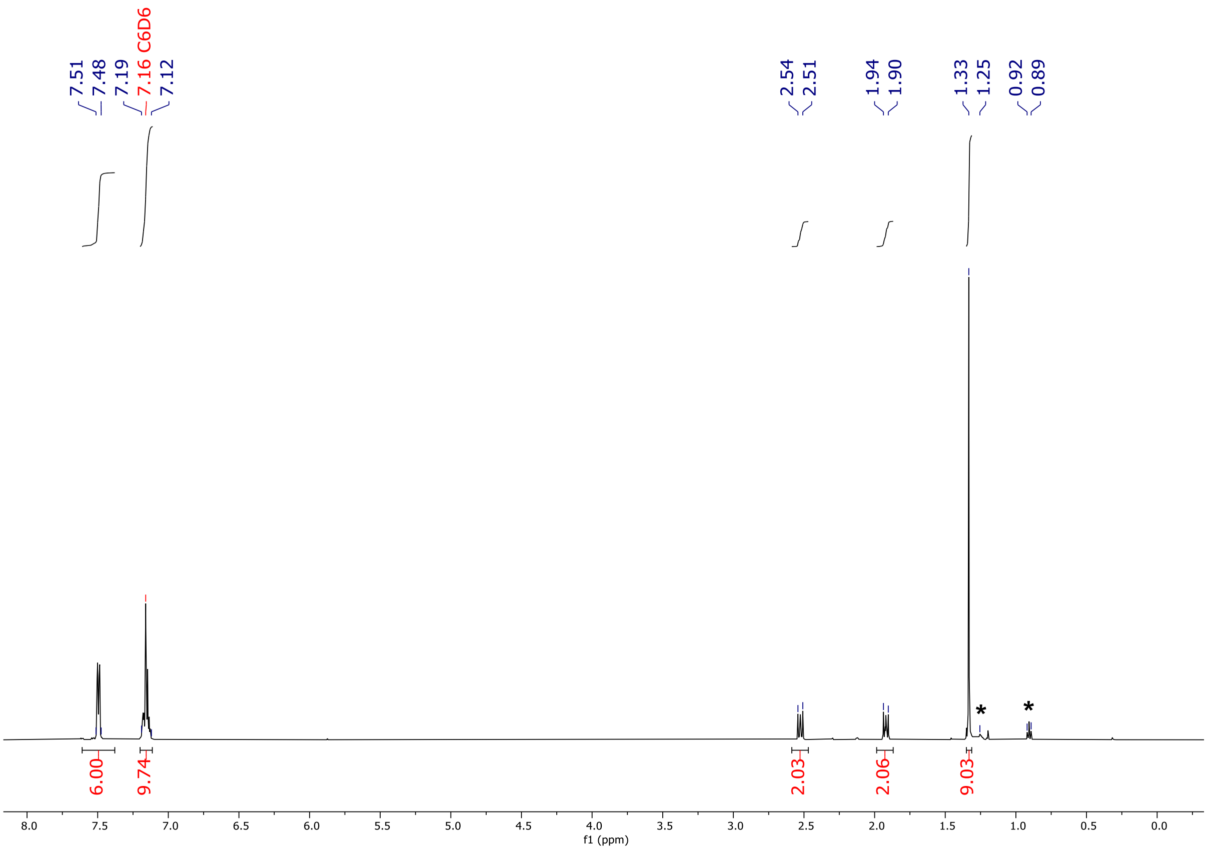


**Figure S65.** $^{1}H$ NMR spectrum (500 MHz, $C_6D_6$) of compound **11c**. * = hexane.

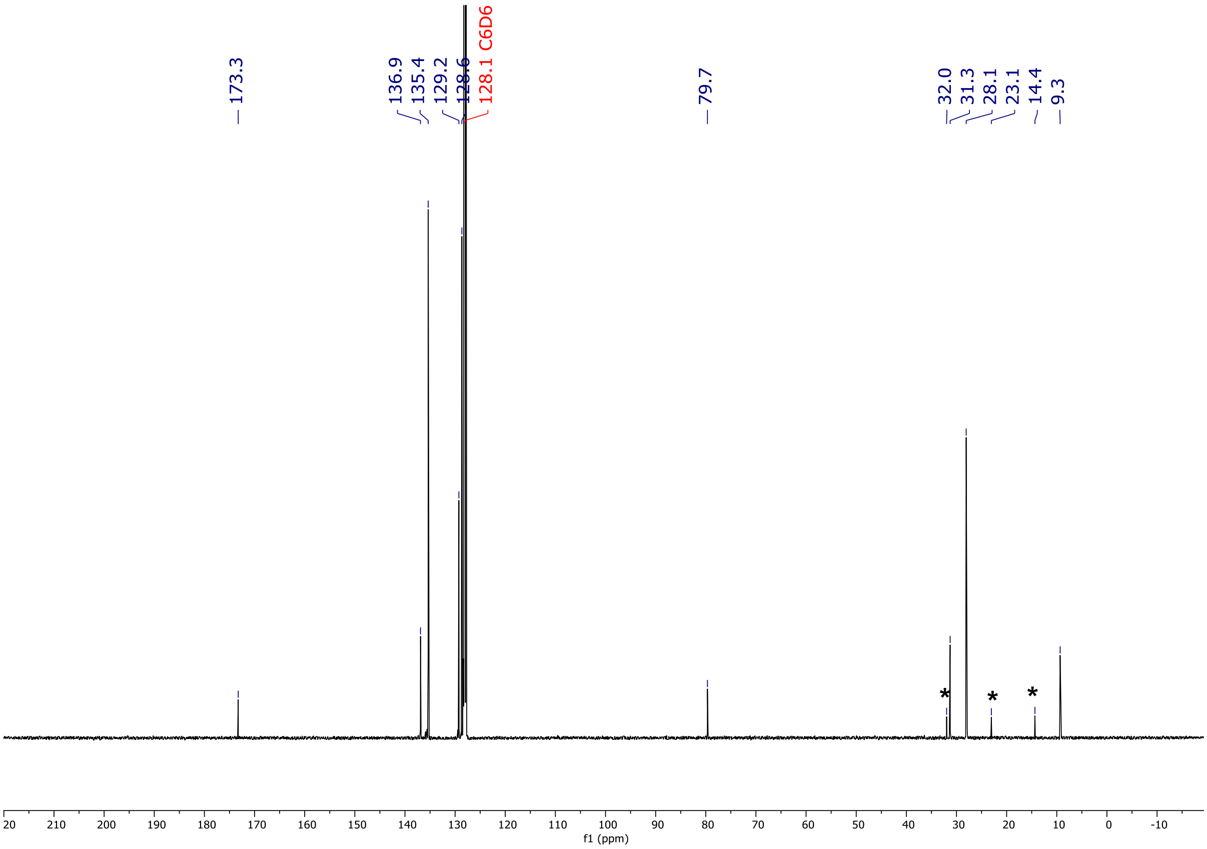


**Figure S66.** $^{13}C\{^{1}H\}$ NMR spectrum (126 MHz, $C_6D_6$) of compound **11c**, * = hexane.

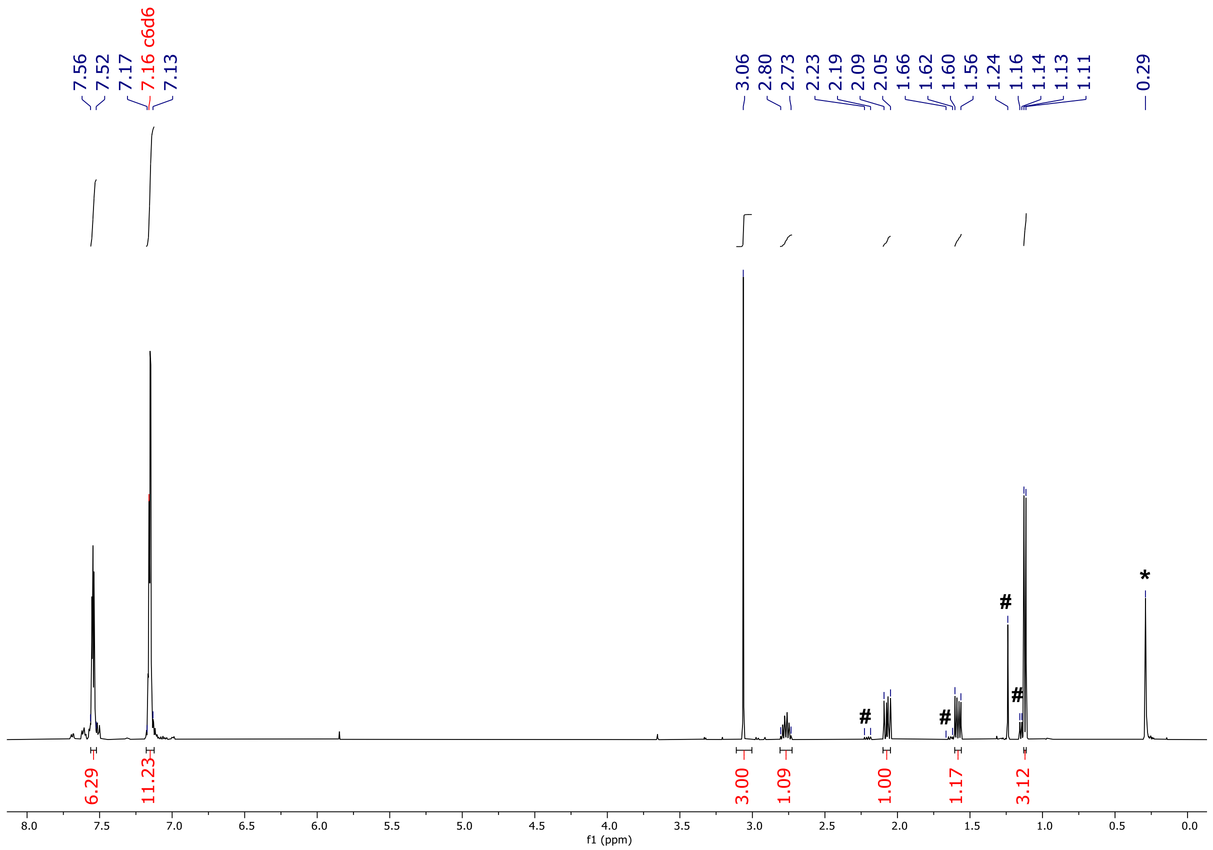


**Figure S67.** $^{1}H$ NMR spectrum (500 MHz, $C_6D_6$) of compound **11d**. * = grease; # = *ca*. 8 mol% contaminated with $Ph_3GeCH_2(CH_3)CHC(O)OC(CH_3)_3$ from competing transesterification of **11d**.

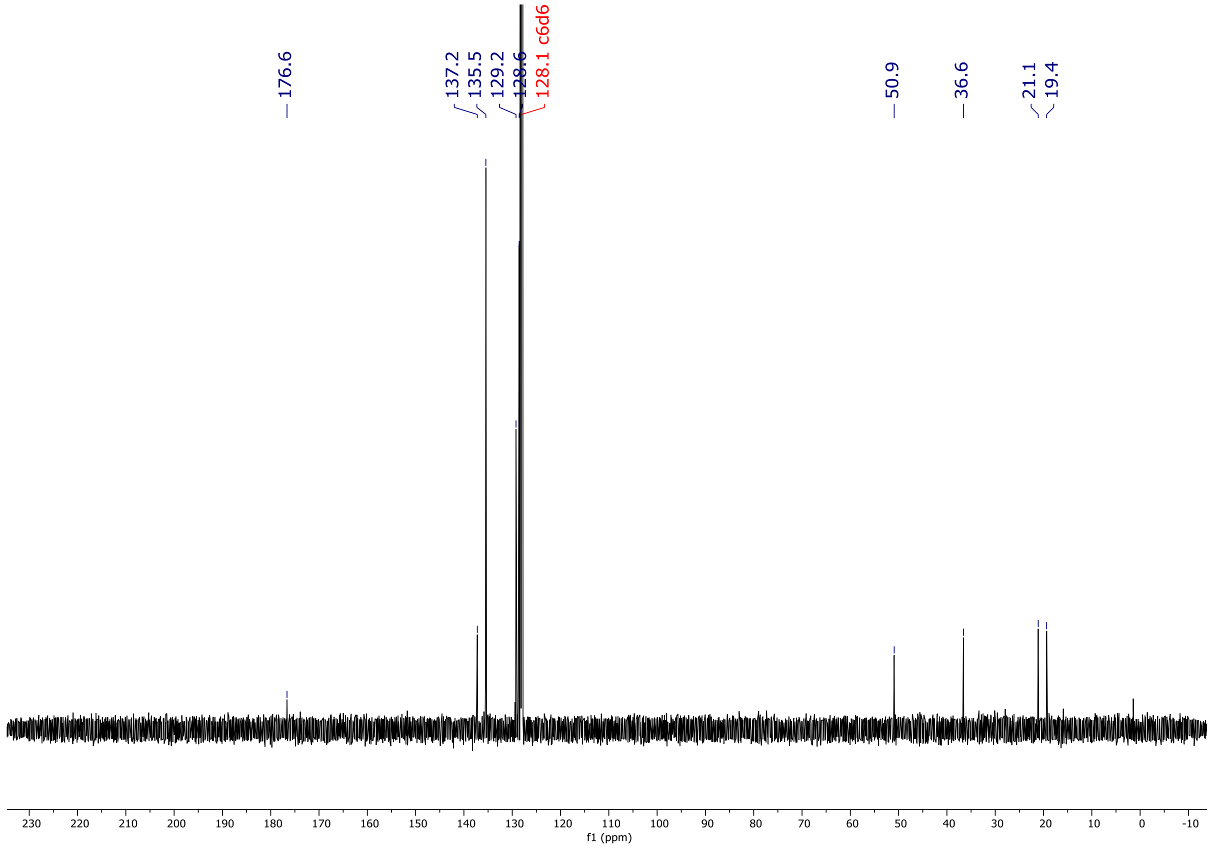


**Figure S68.** $^{13}C\{^{1}H\}$ NMR spectrum (126 MHz, $C_6D_6$) of compound **11d**.

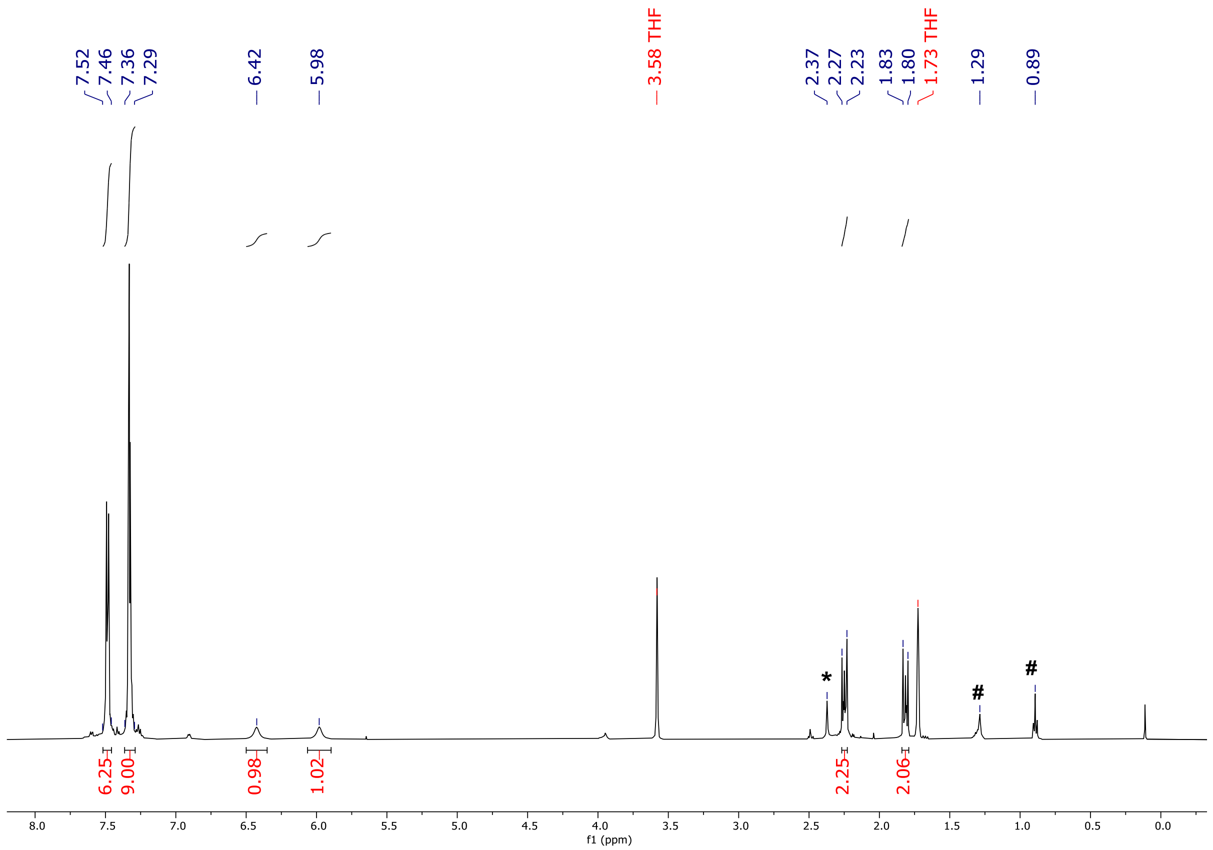


**Figure S69.** $^{1}H$ NMR spectrum (500 MHz, $C_6D_6$) of compound **11g**. * = toluene; # = hexane.

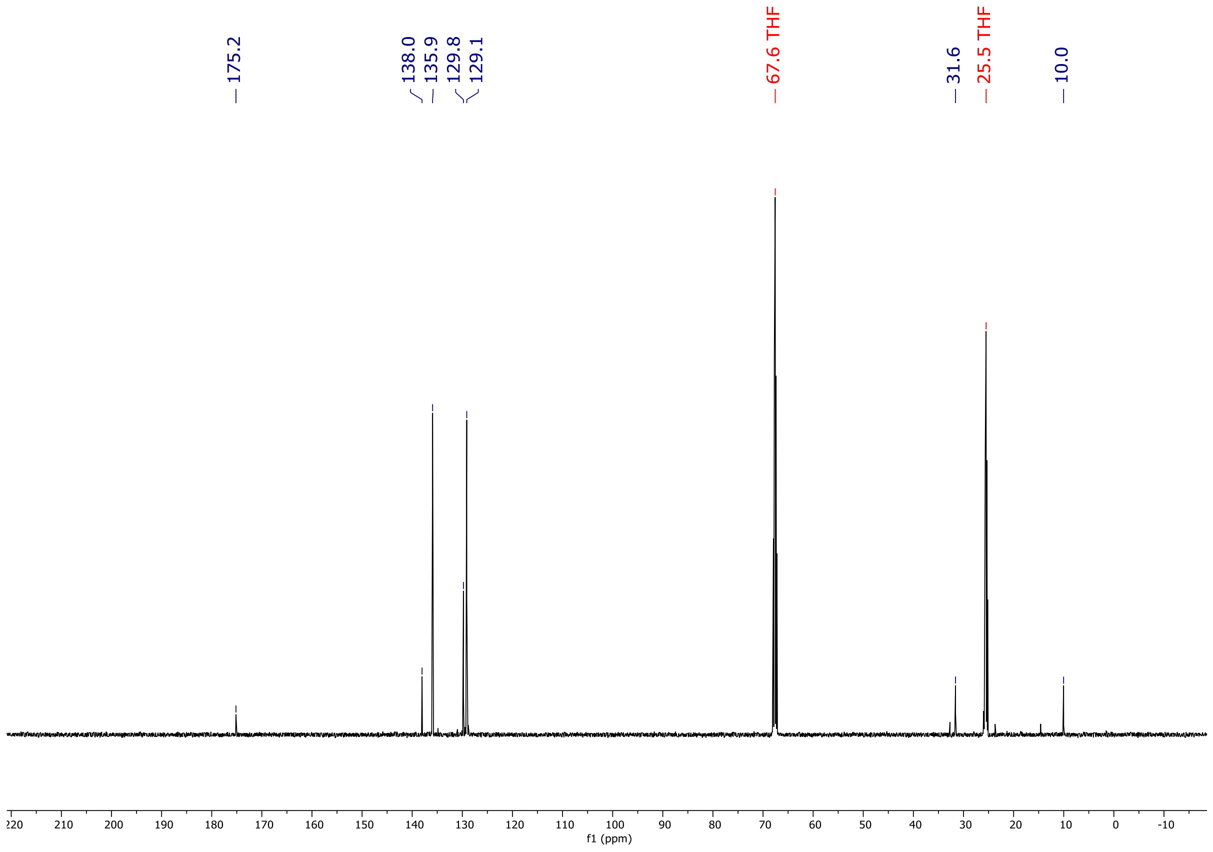


**Figure S70.** $^{13}C\{^{1}H\}$ NMR spectrum (126 MHz, $C_6D_6$) of compound **11g**.

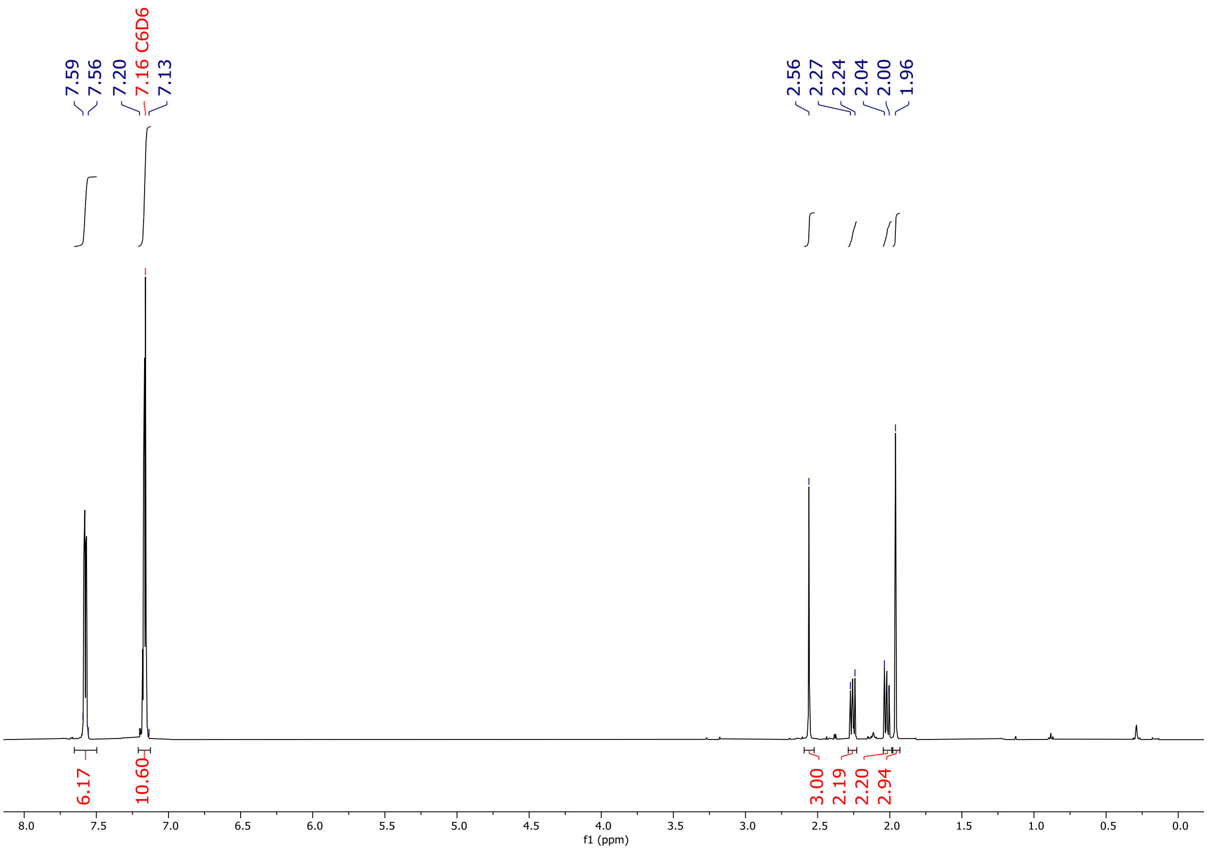


**Figure S71.** $^{1}H$ NMR spectrum (500 MHz, $C_6D_6$) of compound **11h**.

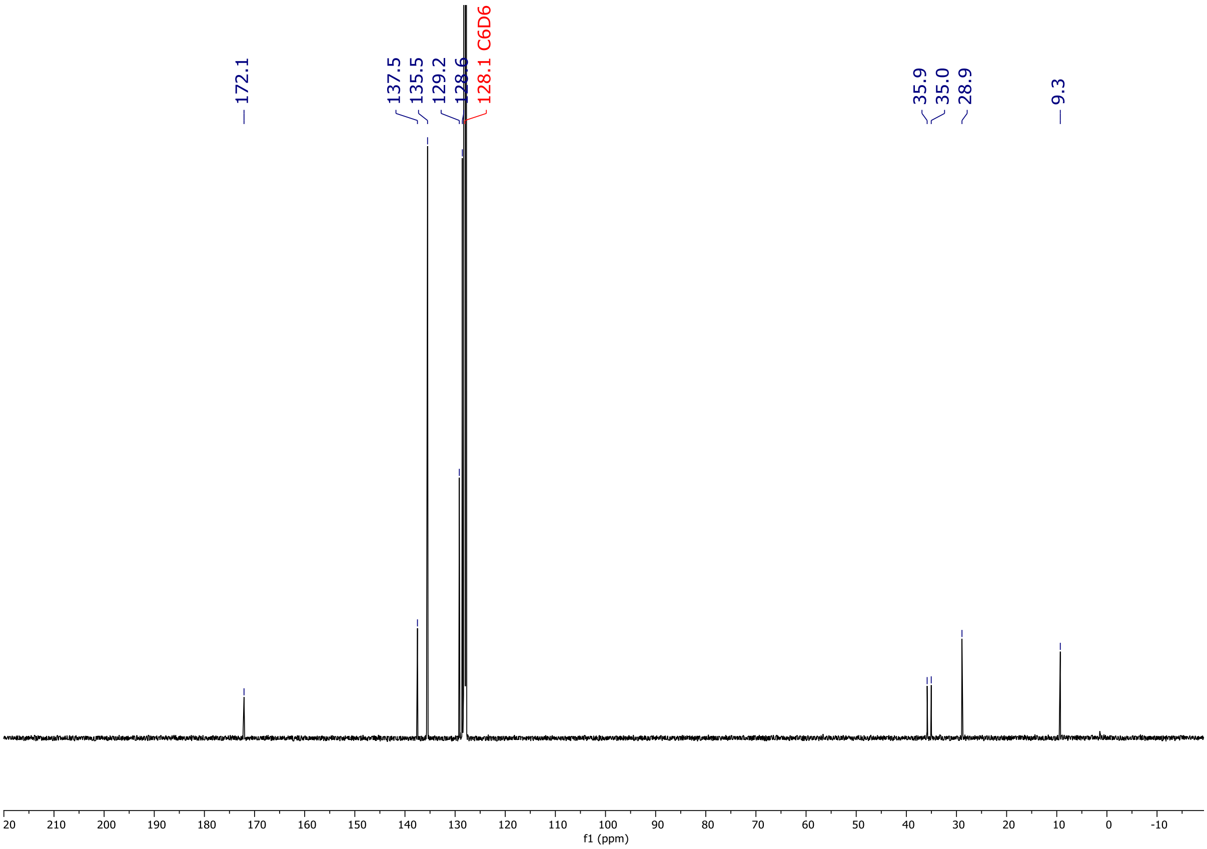


**Figure S72.** $^{13}C\{^{1}H\}$ NMR spectrum (126 MHz, $C_6D_6$) of compound **11h**.

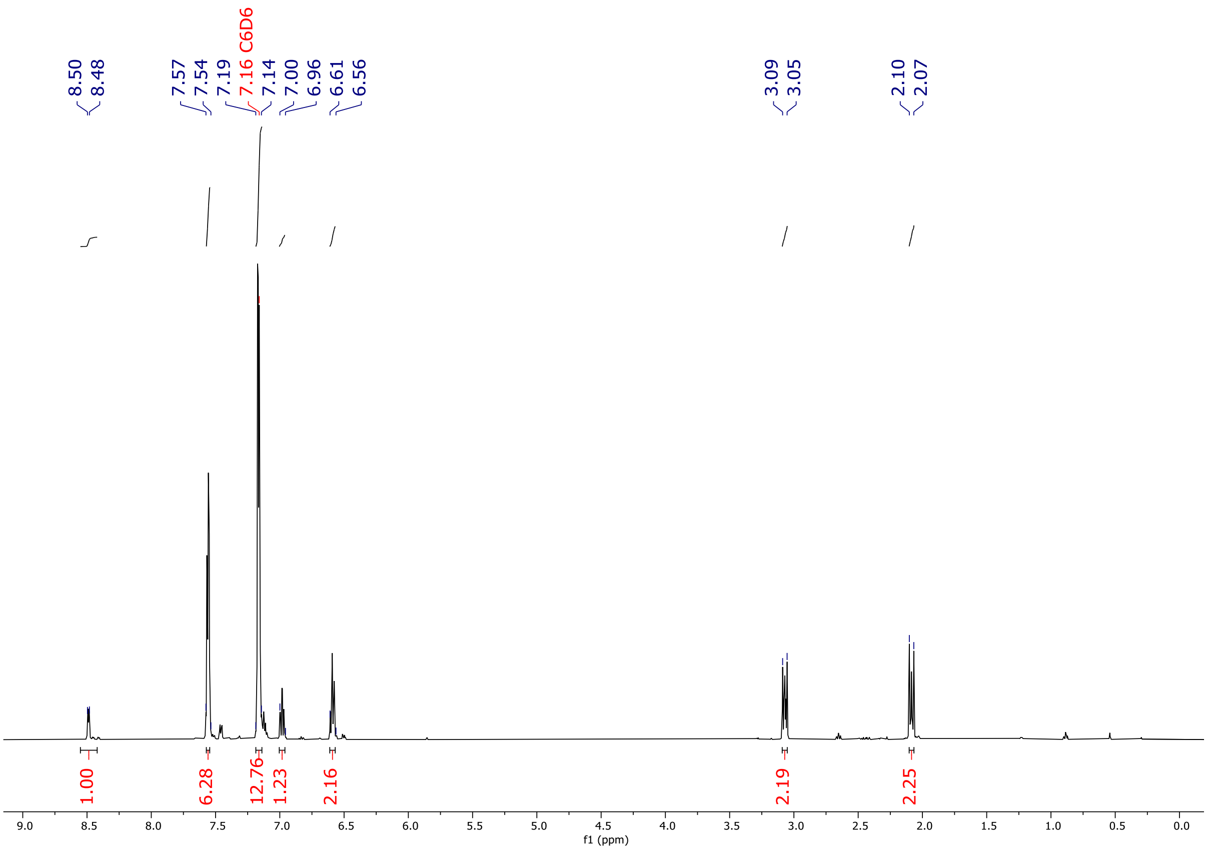


**Figure S73.** $^{1}H$ NMR spectrum (500 MHz, $C_6D_6$) of compound **11k**.

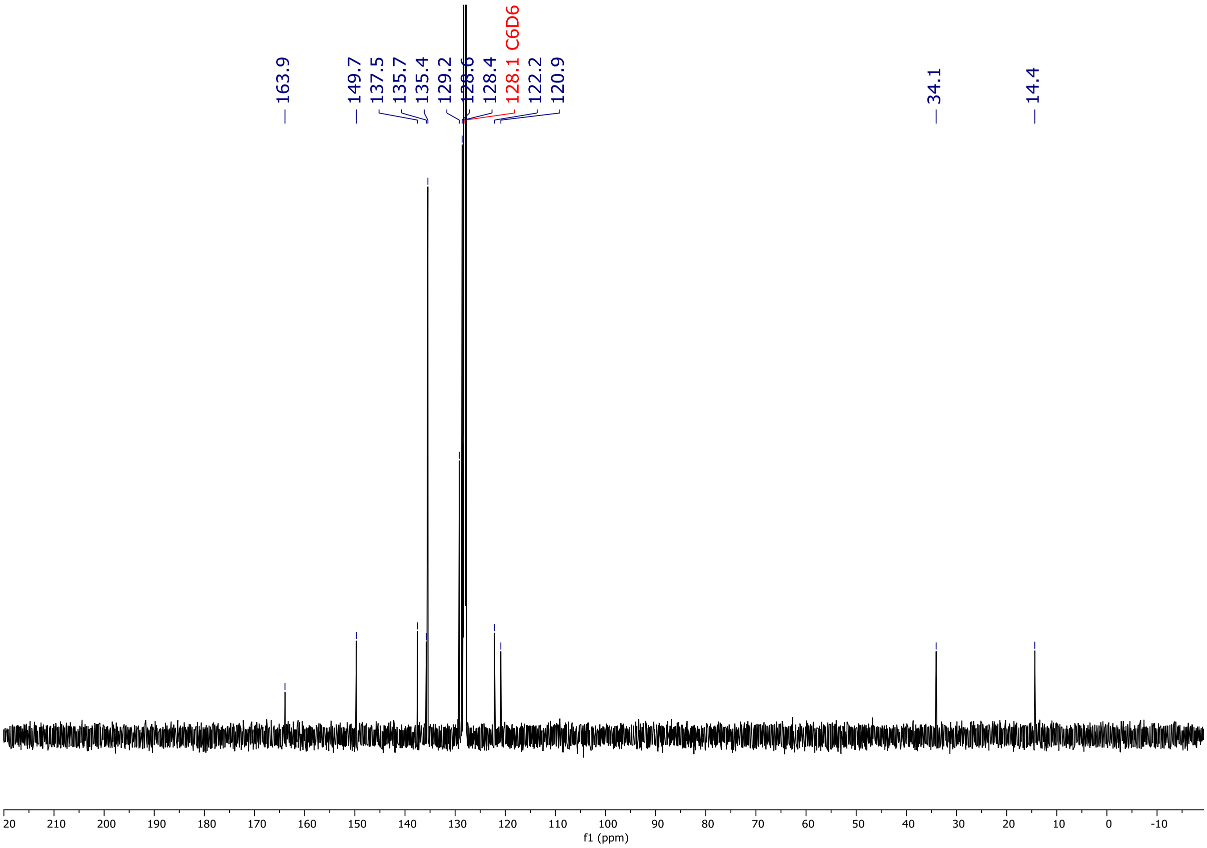


**Figure S74.** $^{13}C\{^{1}H\}$ NMR spectrum (126 MHz, $C_6D_6$) of compound **11k**.

## Computational details

DFT calculations were run with Gaussian 16 (C.01).[16] The Cu and Ge centres were described with the Stuttgart RECPs and associated basis sets,[17] and 6-31G** basis sets were used for all other atoms (BS1).[18] Initial BP86[19,20] optimizations were performed using the 'grid = ultrafine' option, with all stationary points being fully characterized via analytical frequency calculations as minima (all positive eigenvalues). All energies were recomputed with 6-311++G** basis sets for all atoms except copper and germanium, for which the cc-pvtz-pp basis set and corresponding ECP was used (BS2).[21,22] Corrections for the effect of benzene (ε = 2.2706) solvent were run using the polarizable continuum model (PCM) and BS1.[23] Single-point dispersion corrections to the BP86 results employed Grimme's D3 parameter set with Becke-Johnson damping as implemented in Gaussian.[24] Bond analyses were performed on compound **2** using NBO7 as implemented in Gaussian16 and EDA-NOCV[25] analysis was performed as implemented in the ADF program package (2022.103).[26] All EDA-NOCV calculations were performed at the PBE0-d3bj/tz2p//BP86-d3bj/BS1 level of theory (numerical quality very good, nosym).

**EDA-NOCV**

| | IPrCu• + •$GePh_3$ | **IPrCu$^+$ + $^-GePh_3$** | IPrCu$^-$ + $^+GePh_3$ |
|---|---|---|---|
| Pauli repulsion | 138.73 kcal/mol | **82.59 kcal/mol** | 220.50 kcal/mol |
| Electrostatic attraction | -144.14 kcal/mol | **-162.25 kcal/mol** | -241.06 kcal/mol |
| Orbital Interaction | -74.96 kcal/mol | **-55.28 kcal/mol** | -243.50 kcal/mol |
| Dispersion | -8.42 kcal/mol | **-8.42 kcal/mol** | -8.42 kcal/mol |
| Overall Bonding Energy | -88.79 kcal/mol | **-146.36 kcal/mol** | -272.47 kcal/mol |

Orbital interaction for IPrCu$^+$ + $^-GePh_3$ fragmentation broken down as follows:

$\Delta E_{orb1}$ = -36.87 kcal/mol (66.6 %)

$\Delta E_{orb2}$ = -2.03 kcal/mol (3.6 %)

$\Delta E_{orb3}$ = -2.52 kcal/mol (4.5 %)

$\Delta E_{orb4}$ = -2.00 kcal/mol (3.5 %)

$\Delta E_{orb(rest)}$ = -12.10 kcal/mol (21.8 %)

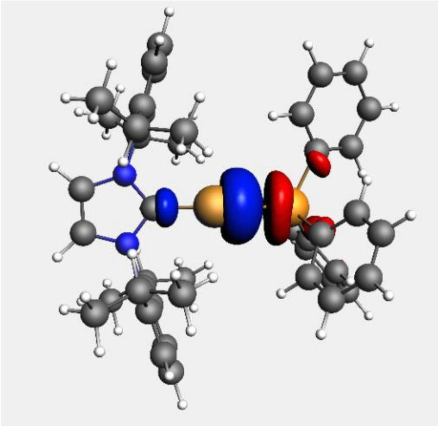

**Figure S75.** Plot of deformation density Δρ(1) of the pairwise orbital interaction between [IPrCu]+ and [$GePh_3$]- in compound 2 associated with energy term $\Delta E_{orb1}$. Charge flow from red to blue.

## Cartesian Coordinates and Computed Energies [in Hartrees]

**Phenylacetylene**
SCF (BP86) Energy = -308.376233126
Enthalpy 0K = -308.271436
Enthalpy 298K = -308.270492
Free Energy 298K = -308.308773
Lowest Frequency = 139.6291 $cm^{-1}$
Second Frequency = 150.8098 $cm^{-1}$
SCF (BP86-D3BJ) Energy = -308.406625354
SCF ($C_6H_6$) Energy = -308.386067339
SCF (BS2) Energy = -308.463935992

C -1.52035 -1.21574 0.00003
C -0.12008 -1.22150 -0.00003
C 0.59980 -0.00001 -0.00006
C -0.12006 1.22149 -0.00003
C -1.52033 1.21575 0.00003
C -2.22513 0.00001 0.00005
H -2.06507 -2.16563 0.00004
H 0.43374 -2.16498 -0.00005
H 0.43378 2.16495 -0.00005
H -2.06504 2.16565 0.00004
H -3.32000 0.00002 0.00010
C 2.02971 -0.00001 -0.00014
C 3.25258 0.00000 -0.00003
H 4.32572 0.00001 0.00096

**Compound 2**
SCF (BP86) Energy = -2056.32851158
Enthalpy 0K = -2055.456950
Enthalpy 298K = -2055.456005
Free Energy 298K = -2055.609058
Lowest Frequency = 6.1777 $cm^{-1}$
Second Frequency = 11.3824 $cm^{-1}$
SCF (BP86-D3BJ) Energy = -2056.59078181
SCF ($C_6H_6$) Energy = -2056.34486638
SCF (BS2) Energy = -2347.69568896

Ge 2.21017 0.14177 0.04930
Cu -0.15028 0.06281 -0.03342
N -3.00439 0.93417 -0.10199
N -2.82589 -1.22245 -0.11965
C -2.06457 -0.07381 -0.08224
C -4.30449 0.42790 -0.15131
H -5.17537 1.07738 -0.17311
C -4.19106 -0.93618 -0.16276
H -4.94330 -1.71944 -0.19790
C -2.28057 -2.56647 -0.09788
C -2.12432 -3.21135 1.15484
C -1.62222 -4.52716 1.14550
H -1.48949 -5.05452 2.09588
C -1.28575 -5.16686 -0.05386
H -0.89314 -6.18896 -0.03672
C -1.44504 -4.50016 -1.27442
H -1.17176 -5.00511 -2.20671
C -1.94663 -3.18494 -1.32819
C -2.48946 -2.54271 2.48028
H -2.73690 -1.48741 2.27108
C -1.30577 -2.54566 3.47255
H -1.57858 -1.99642 4.39017
H -0.41136 -2.06914 3.03904
H -1.03034 -3.57148 3.77372
C -3.74072 -3.20291 3.10536
H -3.55391 -4.26546 3.34025
H -4.60775 -3.15916 2.42423
H -4.01773 -2.69446 4.04512
C -2.11630 -2.48971 -2.67848
H -2.46474 -1.45950 -2.48930
C -0.77395 -2.38772 -3.43638
H -0.37288 -3.38506 -3.68720
H -0.01115 -1.85992 -2.84045
H -0.91336 -1.83885 -4.38390
C -3.19141 -3.19602 -3.53674
H -3.33309 -2.66198 -4.49228
H -4.16485 -3.23538 -3.01858
H -2.89665 -4.23320 -3.77401
C -2.68641 2.34851 -0.06016
C -2.59888 2.98482 1.20300
C -2.29606 4.36037 1.21347
H -2.21304 4.88162 2.17285
C -2.09281 5.06734 0.02213
H -1.85495 6.13572 0.05437
C -2.18714 4.40989 -1.21034
H -2.01726 4.96943 -2.13594
C -2.48768 3.03582 -1.28299
C -2.78582 2.23351 2.52097
H -3.11700 1.20719 2.28428
C -1.44350 2.12056 3.28013
H -0.68154 1.60955 2.66683
H -1.57473 1.54794 4.21462
H -1.05174 3.11761 3.54737
C -3.87758 2.87738 3.40441
H -3.59697 3.89678 3.72134
H -4.03011 2.27876 4.31890
H -4.84245 2.94495 2.87373
C -2.54945 2.33665 -2.64049
H -2.91986 1.30923 -2.47779
C -1.13527 2.22537 -3.25590
H -1.17409 1.68671 -4.21872
H -0.45222 1.68219 -2.57976
H -0.70215 3.22365 -3.44171
C -3.53104 3.03198 -3.60960
H -3.20093 4.05471 -3.86126
H -4.54493 3.10251 -3.18041
H -3.59728 2.46640 -4.55490
C 2.97430 -0.68803 1.73710
C 2.57321 -1.98555 2.13182
H 1.85878 -2.54439 1.51304
C 3.07575 -2.58159 3.30161
H 2.75617 -3.59216 3.58266
C 3.98543 -1.88137 4.11168
H 4.37596 -2.34064 5.02679

```
C   4.39060 -0.58933  3.73982
H   5.10188 -0.03730  4.36555
C   3.89201 -0.00308  2.56280
H   4.22424  1.00330  2.28167
C   3.14177 -0.85878 -1.45330
C   4.07425 -1.89179 -1.21586
H   4.32309 -2.16917 -0.18469
C   4.69246 -2.57352 -2.27954
H   5.41409 -3.37195 -2.06966
C   4.39388 -2.22942 -3.60760
H   4.87728 -2.75763 -4.43720
C   3.47191 -1.20082 -3.86537
H   3.23558 -0.92204 -4.89920
C   2.85271 -0.52922 -2.79766
H   2.13534  0.27236 -3.01696
C   3.04713  1.98695 -0.01755
C   4.28411  2.22364 -0.65712
H   4.80663  1.39406 -1.14802
C   4.85602  3.50777 -0.68254
H   5.81674  3.66811 -1.18591
C   4.19993  4.58414 -0.06322
H   4.64452  5.58565 -0.08212
C   2.96894  4.36919  0.57837
H   2.44937  5.20466  1.06245
C   2.40087  3.08368  0.59544
H   1.43426  2.93207  1.09287
```

**Compound 9**

SCF (BP86) Energy = -2364.73457030
Enthalpy 0K = -2363.754089
Enthalpy 298K = -2363.753145
Free Energy 298K = -2363.918566
Lowest Frequency = 6.0553 $cm^{-1}$
Second Frequency = 11.0659 $cm^{-1}$
SCF (BP86-D3BJ) Energy = -2365.05974322
SCF ($C_6H_6$) Energy = -2364.76057947
SCF (BS2) Energy = -2656.185385

```
Ge -3.10468 -0.62661 -0.09000
Cu  0.76610 -0.39675 -0.34167
N   2.37600  2.06004  0.32166
N   3.24805  0.23178  1.09723
C   2.16136  0.69623  0.37916
C   3.55560  2.42258  0.97577
H   3.89706  3.45283  1.01507
C   4.10179  1.27126  1.46964
H   5.01164  1.09292  2.03589
C   3.58196 -1.16665  1.30725
C   4.54827 -1.75093  0.44874
C   4.91773 -3.08725  0.70047
H   5.66298 -3.56476  0.05586
C   4.34231 -3.81158  1.74942
H   4.63987 -4.85062  1.92589
C   3.37811 -3.21246  2.57021
H   2.92547 -3.79256  3.37909
C   2.97488 -1.87742  2.37476
C   5.19420 -0.99888 -0.71616
H   4.68494 -0.02508 -0.81980
C   5.01607 -1.75174 -2.05355
H   5.40752 -1.14218 -2.88638
H   3.95580 -1.97765 -2.25140
H   5.56989 -2.70688 -2.05810
C   6.68876 -0.71601 -0.43655
H   7.25594 -1.65646 -0.32294
H   6.82887 -0.12916  0.48730
H   7.13823 -0.15094 -1.27144
C   1.95392 -1.22796  3.30890
H   1.41863 -0.45427  2.72939
C   0.89147 -2.21841  3.82483
H   1.31796 -2.95313  4.53085
H   0.41694 -2.76637  2.99462
H   0.09909 -1.66880  4.35787
C   2.66903 -0.52830  4.49068
H   1.93196 -0.04003  5.15169
H   3.37832  0.24254  4.14447
H   3.23330 -1.26091  5.09455
C   1.57979  3.01179 -0.43192
C   0.72880  3.90278  0.27159
C   0.02519  4.85932 -0.48471
H  -0.64381  5.55736  0.02727
C   0.15269  4.92223 -1.87686
H  -0.41220  5.66901 -2.44401
C   0.99736  4.02869 -2.54322
H   1.09197  4.08318 -3.63280
C   1.73842  3.05829 -1.84091
C   0.57447  3.87249  1.79317
H   0.96505  2.90289  2.15163
C   1.40712  4.99530  2.45843
H   2.48069  4.91592  2.21856
H   1.30157  4.95682  3.55660
H   1.06261  5.98808  2.11946
C  -0.90156  3.97444  2.23590
H  -1.32087  4.97339  2.02366
H  -0.98216  3.81237  3.32413
H  -1.53211  3.22596  1.73127
C   2.67527  2.13039 -2.61455
H   3.19425  1.48111 -1.88874
C   3.75688  2.92999 -3.37663
H   4.45429  2.24104 -3.88368
H   4.34196  3.57231 -2.69657
H   3.31191  3.57922 -4.15067
C   1.88549  1.20415 -3.56623
H   1.32985  1.78518 -4.32319
H   1.16107  0.58775 -3.00608
H   2.57280  0.52499 -4.09931
C  -4.87753 -1.54183 -0.28066
C  -4.97712 -2.93553 -0.06791
H  -4.08363 -3.51316  0.19937
C  -6.20771 -3.60162 -0.19812
H  -6.26449 -4.68311 -0.03030
C  -7.36225 -2.88191 -0.54682
H  -8.32228 -3.39952 -0.65117
C  -7.28006 -1.49712 -0.76490
H  -8.17707 -0.93154 -1.04180
C  -6.04738 -0.83514 -0.63286
H  -5.99524  0.24502 -0.81375
C  -2.81739 -0.35057  1.87796
C  -3.77776 -0.79900  2.81009
```

H  -4.66755 -1.33226  2.45633
C  -3.61325 -0.56988  4.18741
H  -4.37159 -0.92536  4.89417
C  -2.48326  0.11870  4.65623
H  -2.35721  0.30640  5.72838
C  -1.51636  0.56886  3.74101
H  -0.63227  1.10905  4.09960
C  -1.68270  0.33370  2.36497
H  -0.91485  0.68405  1.66235
C  -3.27715  1.18657 -0.91958
C  -3.92795  2.23920 -0.23884
H  -4.27874  2.08962  0.78960
C  -4.13607  3.48168 -0.86297
H  -4.64907  4.28599 -0.32321
C  -3.68985  3.68987 -2.17838
H  -3.85698  4.65552 -2.66870
C  -3.03107  2.65567 -2.86346
H  -2.67840  2.81489 -3.88874
C  -2.82736  1.41416 -2.23750
H  -2.30939  0.61120 -2.77479
C   0.40448 -3.97567 -4.05791
C  -0.23467 -3.00749 -3.26866
C   0.23718 -2.68558 -1.97066
C   1.38772 -3.37114 -1.50586
C   2.01342 -4.35586 -2.28490
C   1.52905 -4.66063 -3.56904
H   0.02133 -4.19707 -5.06090
H  -1.10959 -2.47413 -3.65641
H   1.77400 -3.13564 -0.50687
H   2.88736 -4.88451 -1.88759
H   2.02661 -5.41881 -4.18347
C  -0.44725 -1.66168 -1.11985
C  -1.80613 -1.77092 -1.02988
H  -2.30944 -2.61212 -1.54537

**TS1$_9$**

SCF (BP86) Energy = -2364.70355765
Enthalpy 0K = -2363.717708
Enthalpy 298K = -2363.716763
Free Energy 298K = -2363.881402
Lowest Frequency = -66.6942 cm$^{-1}$
Second Frequency = 10.6310 cm$^{-1}$
SCF (BP86-D3BJ) Energy = -2365.02205855
SCF ($C_6H_6$) Energy = -2364.71897370
SCF (BS2) Energy = -2656.145007

Ge -2.29469 -0.98064 -0.30089
Cu -0.06403 -0.11793 -0.11072
N   0.85971  2.53170  0.75121
N   2.02961  1.01404  1.76828
C   1.11981  1.17127  0.73448
C   1.56918  3.18539  1.75996
H   1.49863  4.25856  1.91137
C   2.29776  2.22905  2.40709
H   2.98424  2.29661  3.24631
C   2.78460 -0.18926  2.06965
C   4.16846 -0.19532  1.75110
C   4.91009 -1.34454  2.09183
H   5.97979 -1.37503  1.85980
C   4.30316 -2.44185  2.70961
H   4.89665 -3.32666  2.96395
C   2.93452 -2.40904  3.00787
H   2.46992 -3.27200  3.49214
C   2.14258 -1.28436  2.70712
C   4.89044  0.98188  1.08718
H   4.13852  1.74185  0.81485
C   5.61120  0.57151 -0.21495
H   6.07786  1.45700 -0.68088
H   4.91453  0.12374 -0.94048
H   6.41286 -0.16248 -0.02580
C   5.88516  1.64345  2.07059
H   6.67933  0.93602  2.36687
H   5.38825  1.98587  2.99441
H   6.37092  2.51615  1.60054
C   0.66874 -1.23765  3.11217
H   0.11701 -0.71274  2.30575
C   0.02044 -2.62717  3.25513
H   0.40714 -3.17832  4.13142
H   0.18617 -3.24563  2.35696
H  -1.06650 -2.50960  3.39152
C   0.48835 -0.42463  4.41716
H  -0.58096 -0.37314  4.68411
H   0.86699  0.60655  4.31805
H   1.02723 -0.90588  5.25288
C   0.03895  3.24104 -0.21169
C  -1.23400  3.72147  0.19102
C  -1.97058  4.47014 -0.74671
H  -2.95885  4.84818 -0.46859
C  -1.47222  4.72208 -2.02994
H  -2.06779  5.30091 -2.74350
C  -0.21629  4.23046 -2.40150
H   0.16852  4.43182 -3.40698
C   0.57371  3.48734 -1.50245
C  -1.81021  3.47087  1.58512
H  -1.22129  2.65932  2.04975
C  -1.67582  4.72905  2.47673
H  -0.62687  5.05157  2.58903
H  -2.07981  4.53250  3.48502
H  -2.23991  5.57468  2.04530
C  -3.28273  3.00869  1.53332
H  -3.95118  3.81857  1.19237
H  -3.62066  2.70864  2.53954
H  -3.41687  2.15097  0.85577
C   1.95753  3.00969 -1.94197
H   2.41985  2.46897 -1.09891
C   2.87724  4.20153 -2.29386
H   3.88615  3.84152 -2.56008
H   2.97502  4.90107 -1.44638
H   2.48945  4.77231 -3.15567
C   1.85909  2.01235 -3.11735
H   1.39624  2.47810 -4.00524
H   1.25844  1.13068 -2.83768
H   2.86605  1.66382 -3.40535
C  -2.47598 -2.77150 -1.25761
C  -2.06248 -3.96914 -0.62647
H  -1.68609 -3.93727  0.40404
C  -2.13505 -5.20782 -1.28667
H  -1.81483 -6.12163 -0.77260

C  -2.61722 -5.27405 -2.60436
H  -2.67279 -6.23749 -3.12317
C  -3.02559 -4.09695 -3.25243
H  -3.40493 -4.14072 -4.28006
C  -2.95500 -2.86195 -2.58451
H  -3.28783 -1.95362 -3.10110
C  -3.28074 -1.31088  1.44687
C  -4.25193 -2.32850  1.58389
H  -4.46924 -2.98693  0.73483
C  -4.94131 -2.52281  2.79461
H  -5.68922 -3.32040  2.87449
C  -4.67468 -1.69826  3.89937
H  -5.21087 -1.84831  4.84304
C  -3.71357 -0.67975  3.78554
H  -3.49986 -0.02963  4.64250
C  -3.02598 -0.49571  2.57281
H  -2.27271  0.29926  2.50121
C  -3.56973  0.20084 -1.35533
C  -4.95109  0.26064 -1.06476
H  -5.35292 -0.31566 -0.22271
C  -5.82277  1.04946 -1.83539

**$Int_9$**

SCF (BP86) Energy = -2364.71053565
Enthalpy 0K = -2363.727499
Enthalpy 298K = -2363.726555
Free Energy 298K = -2363.887526
Lowest Frequency = 10.8972 $cm^{-1}$
Second Frequency = 15.8715 $cm^{-1}$
SCF (BP86-D3BJ) Energy = -2365.04688703
SCF ($C_6H_6$) Energy = -2364.72927162
SCF (BS2) Energy = -2656.157705

Ge -2.09679 -0.89372 -0.37841
Cu  0.30295 -0.34547 -0.58873
N   0.71412  2.14986  1.05354
N   1.89580  0.49078  1.81392
C   1.04145  0.83116  0.77611
C   1.32837  2.59354  2.22959
H   1.19192  3.60503  2.59989
C   2.05944  1.54907  2.71301
H   2.68822  1.45995  3.59412
C   2.73903 -0.69150  1.87536
C   4.11846 -0.53341  1.57547
C   4.94152 -1.67214  1.68584
H   6.00924 -1.57947  1.46187
C   4.41859 -2.91278  2.06431
H   5.07584 -3.78591  2.13779
C   3.05425 -3.03776  2.35684
H   2.65477 -4.00916  2.66181
C   2.18487 -1.93214  2.28295
C   4.75038  0.80589  1.18397
H   3.94182  1.54258  1.04180
C   5.53104  0.72956 -0.14550
H   5.93642  1.72518 -0.39788
H   4.88956  0.39366 -0.97456
H   6.38562  0.03445 -0.08040
C   5.66459  1.32925  2.31792
H  -6.89115  1.08124 -1.59110
C  -5.32820  1.79261 -2.92013
H  -6.00746  2.40451 -3.52448
C  -3.95770  1.74840 -3.22338
H  -3.56252  2.32836 -4.06554
C  -3.08989  0.96464 -2.44261
H  -2.01855  0.95343 -2.68094
C   5.30909 -1.48296 -3.73402
C   3.95335 -1.38229 -3.39895
C   3.45021 -2.02890 -2.23807
C   4.34723 -2.77672 -1.42907
C   5.69878 -2.87582 -1.77936
C   6.18641 -2.23129 -2.92980
H   5.68233 -0.98192 -4.63345
H   3.26276 -0.81775 -4.03203
H   3.96453 -3.27226 -0.53263
H   6.37749 -3.46255 -1.15126
H   7.24407 -2.31426 -3.20041
C   2.05974 -1.99456 -1.93080
C   0.85199 -1.99552 -1.67780
H  -0.17586 -2.32850 -1.66746
H   6.50931  0.64063  2.49493
H   5.12071  1.43646  3.27190
H   6.08425  2.31488  2.05168
C   0.72708 -2.05734  2.71848
H   0.13703 -1.32469  2.14036
C   0.11232 -3.44158  2.43902
H   0.54180 -4.22843  3.08476
H   0.25440 -3.74161  1.38739
H  -0.97059 -3.40542  2.64250
C   0.59256 -1.69872  4.21862
H  -0.46554 -1.74885  4.52609
H   0.96211 -0.68070  4.43015
H   1.17038 -2.40447  4.84178
C  -0.05079  3.04818  0.20507
C  -1.28639  3.56048  0.67927
C  -1.96671  4.48413 -0.13939
H  -2.92673  4.88574  0.19871
C  -1.44843  4.88261 -1.37341
H  -2.00104  5.59304 -1.99693
C  -0.21683  4.37869 -1.80844
H   0.19056  4.71206 -2.76700
C   0.51882  3.46465 -1.03108
C  -1.88814  3.19800  2.03811
H  -1.33277  2.33187  2.43856
C  -1.73195  4.36880  3.03951
H  -0.67932  4.66825  3.18113
H  -2.14126  4.08924  4.02575
H  -2.28115  5.25926  2.68653
C  -3.37399  2.79107  1.93350
H  -4.00991  3.65134  1.66037
H  -3.73039  2.41096  2.90548
H  -3.52767  2.00316  1.18072
C   1.92416  3.04584 -1.47121
H   2.11306  2.02647 -1.09227
C   2.97601  3.99364 -0.84251
H   3.99602  3.68310 -1.12880
H   2.91735  3.99758  0.25902
H   2.82390  5.03049 -1.19177
C   2.10147  2.99412 -3.00107

```
H   2.06827  3.99810 -3.46024
H   1.32527  2.37237 -3.47786
H   3.08562  2.55994 -3.24297
C  -2.58521 -2.60167 -1.37472
C  -2.37090 -3.87441 -0.79646
H  -2.00422 -3.94767  0.23454
C  -2.63317 -5.05661 -1.50912
H  -2.46726 -6.03017 -1.03298
C  -3.10733 -4.99247 -2.83033
H  -3.30995 -5.91274 -3.38931
C  -3.31972 -3.74007 -3.42744
H  -3.69279 -3.67905 -4.45650
C  -3.06205 -2.56023 -2.70493
H  -3.24989 -1.59034 -3.18174
C  -2.98251 -1.19453  1.43205
C  -4.09682 -2.05544  1.56785
H  -4.46896 -2.60085  0.69308
C  -4.73379 -2.23730  2.80867
H  -5.59651 -2.90929  2.88502
C  -4.26705 -1.56203  3.94782
H  -4.76136 -1.70310  4.91536
C  -3.15872 -0.70619  3.83725
H  -2.78570 -0.17431  4.72055
C  -2.52749 -0.53124  2.59239
H  -1.65957  0.13631  2.52504
C  -3.28928  0.46326 -1.31420
C  -4.66030  0.59345 -0.99822
H  -5.09052 -0.00620 -0.18688
C  -5.48780  1.48160 -1.70692
H  -6.54891  1.56695 -1.44467
C  -4.95864  2.25434 -2.75410
H  -5.60392  2.94283 -3.31132
C  -3.59805  2.13950 -3.08141
H  -3.17640  2.74113 -3.89493
C  -2.77356  1.25595 -2.36238
H  -1.70902  1.18619 -2.61935
C   4.83094 -0.64405 -3.90320
C   3.49795 -0.48938 -3.49999
C   2.97366 -1.26358 -2.43120
C   3.83286 -2.18520 -1.77672
C   5.15875 -2.34106 -2.19926
C   5.66787 -1.57300 -3.26098
H   5.21440 -0.04389 -4.73563
H   2.84033  0.21347 -4.01936
H   3.44196 -2.77744 -0.94438
H   5.80100 -3.06880 -1.69172
H   6.70604 -1.69730 -3.58551
C   1.58232 -1.18211 -2.06716
C   0.34562 -1.46721 -2.17351
H  -0.43594 -2.03938 -2.66849
```

**TS2$_9$**

SCF (BP86) Energy = -2364.69212453
Enthalpy 0K = -2363.706178
Enthalpy 298K = -2363.705233
Free Energy 298K = -2363.866949
Lowest Frequency = -128.9081 cm$^{-1}$
Second Frequency = 10.9417 cm$^{-1}$
SCF (BP86-D3BJ) Energy = -2365.01978327
SCF ($C_6H_6$) Energy = -2364.70851773
SCF (BS2) Energy = -2656.135861

```
Ge  2.16078 -0.90209  0.01565
Cu -0.48335 -0.46215  0.19593
N  -1.32021  2.39298 -0.22041
N  -2.24405  0.98176 -1.60157
C  -1.31929  1.04731 -0.56542
C  -2.20575  3.12240 -1.02254
H  -2.34854  4.19044 -0.88861
C  -2.77693  2.23996 -1.89186
H  -3.51515  2.38156 -2.67601
C  -2.81500 -0.24509 -2.13004
C  -4.07360 -0.66176 -1.61909
C  -4.63251 -1.84017 -2.15242
H  -5.60208 -2.18597 -1.77906
C  -3.97182 -2.57292 -3.14478
H  -4.42242 -3.48953 -3.54009
C  -2.73784 -2.13132 -3.63699
H  -2.23210 -2.70534 -4.41900
C  -2.13240 -0.95720 -3.14829
C  -4.85584  0.13082 -0.56851
H  -4.19890  0.92591 -0.17687
C  -5.28678 -0.73465  0.63458
H  -5.80414 -0.10748  1.38141
H  -4.42256 -1.21185  1.12290
H  -5.99019 -1.53166  0.33585
C  -6.08553  0.81610 -1.21218
H  -6.78951  0.06670 -1.61470
H  -5.79798  1.48116 -2.04422
H  -6.62715  1.42015 -0.46371
C  -0.82551 -0.44914 -3.75016
H  -0.33026  0.18097 -2.99311
C   0.16145 -1.57680 -4.10935
H  -0.19178 -2.18339 -4.96247
H   0.32661 -2.25096 -3.25277
H   1.13417 -1.14183 -4.39136
C  -1.11033  0.44034 -4.98436
H  -0.16542  0.84241 -5.38870
H  -1.76411  1.29259 -4.73132
H  -1.60681 -0.14005 -5.78237
C  -0.65934  2.98768  0.92734
C   0.39065  3.91921  0.71320
C   0.96649  4.52551  1.84668
H   1.78189  5.24258  1.71000
C   0.52100  4.22546  3.13740
H   0.98928  4.70368  4.00397
C  -0.53180  3.32253  3.31860
H  -0.89255  3.11015  4.32984
C  -1.15654  2.69081  2.22609
C   0.88282  4.32704 -0.67701
H   0.46286  3.61594 -1.41042
C   0.37476  5.74164 -1.04862
H  -0.72601  5.81095 -1.02472
H   0.71076  6.01976 -2.06275
H   0.76754  6.49515 -0.34366
C   2.42141  4.27045 -0.79516
H   2.90306  5.04395 -0.17184
```

| | | | |
|---|---|---|---|
| H | 2.72979 | 4.45017 | -1.83905 |
| H | 2.81418 | 3.29014 | -0.48463 |
| C | -2.38869 | 1.81846 | 2.47513 |
| H | -2.58556 | 1.22920 | 1.56414 |
| C | -3.62086 | 2.71723 | 2.74145 |
| H | -4.52417 | 2.09902 | 2.88537 |
| H | -3.80890 | 3.40968 | 1.90288 |
| H | -3.47850 | 3.32518 | 3.65252 |
| C | -2.18679 | 0.80060 | 3.61553 |
| H | -2.02259 | 1.29202 | 4.59081 |
| H | -1.33282 | 0.13866 | 3.40139 |
| H | -3.08275 | 0.16518 | 3.71342 |
| C | 3.36982 | -2.52249 | 0.07034 |
| C | 3.08348 | -3.66031 | -0.71824 |
| H | 2.19937 | -3.66892 | -1.36868 |
| C | 3.91707 | -4.79146 | -0.68994 |
| H | 3.67727 | -5.66294 | -1.30956 |
| C | 5.05506 | -4.80354 | 0.13322 |
| H | 5.70559 | -5.68458 | 0.15923 |
| C | 5.35273 | -3.68238 | 0.92398 |
| H | 6.23791 | -3.68675 | 1.57014 |
| C | 4.51542 | -2.55307 | 0.89275 |
| H | 4.75798 | -1.68668 | 1.51874 |
| C | 2.66165 | -0.07666 | -1.77766 |
| C | 3.70956 | -0.63011 | -2.54661 |
| H | 4.23449 | -1.52241 | -2.18984 |
| C | 4.09184 | -0.05089 | -3.77012 |
| H | 4.90641 | -0.49760 | -4.35154 |
| C | 3.43608 | 1.09630 | -4.24498 |
| H | 3.73412 | 1.54843 | -5.19730 |
| C | 2.39478 | 1.66068 | -3.48849 |
| H | 1.87731 | 2.55697 | -3.84975 |
| C | 2.01208 | 1.07330 | -2.27023 |
| H | 1.18599 | 1.50779 | -1.69511 |
| C | 2.87212 | 0.31912 | 1.44542 |
| C | 3.94174 | 1.20092 | 1.17355 |
| H | 4.34788 | 1.26876 | 0.15705 |
| C | 4.50485 | 1.98572 | 2.19482 |
| H | 5.33938 | 2.65927 | 1.96862 |
| C | 4.00289 | 1.90181 | 3.50398 |
| H | 4.44336 | 2.51053 | 4.30144 |
| C | 2.93740 | 1.03210 | 3.78734 |
| H | 2.54289 | 0.96196 | 4.80723 |
| C | 2.37725 | 0.24662 | 2.76534 |
| H | 1.55052 | -0.43513 | 2.99550 |
| C | -2.18847 | -4.02617 | 4.24312 |
| C | -1.33175 | -3.19624 | 3.50717 |
| C | -1.63455 | -2.84199 | 2.16609 |
| C | -2.82476 | -3.36273 | 1.59431 |
| C | -3.66451 | -4.20919 | 2.33071 |
| C | -3.35995 | -4.54094 | 3.66249 |
| H | -1.93165 | -4.28114 | 5.27775 |
| H | -0.41486 | -2.80883 | 3.96412 |
| H | -3.06817 | -3.10805 | 0.55730 |
| H | -4.56860 | -4.61182 | 1.85953 |
| H | -4.02504 | -5.19270 | 4.23841 |
| C | -0.74795 | -1.96853 | 1.40899 |
| C | 0.45111 | -2.18894 | 0.88784 |
| H | 1.04996 | -3.10765 | 0.81815 |

**Compound 10**

SCF (BP86) Energy = -2364.74086034
Enthalpy 0K = -2363.752780
Enthalpy 298K = -2363.751836
Free Energy 298K = -2363.916458
Lowest Frequency = 7.4664 $cm^{-1}$
Second Frequency = 8.5196 $cm^{-1}$
SCF (BP86-D3BJ) Energy = -2365.05573728
SCF ($C_6H_6$) Energy = -2364.75936950
SCF (BS2) Energy = -2656.184351

| | | | |
|---|---|---|---|
| Ge | -2.81625 | -0.29478 | 0.46065 |
| Cu | 0.82510 | 0.01217 | -0.68485 |
| N | 3.29555 | 1.46534 | -0.04298 |
| N | 3.71492 | -0.64972 | -0.23512 |
| C | 2.66266 | 0.24713 | -0.19944 |
| C | 4.68327 | 1.32997 | 0.00765 |
| H | 5.34109 | 2.18598 | 0.12979 |
| C | 4.94818 | -0.00535 | -0.11697 |
| H | 5.88628 | -0.55287 | -0.13279 |
| C | 3.58916 | -2.09021 | -0.34148 |
| C | 3.34315 | -2.66945 | -1.61168 |
| C | 3.25783 | -4.07447 | -1.67526 |
| H | 3.06634 | -4.55243 | -2.64164 |
| C | 3.42385 | -4.86636 | -0.53317 |
| H | 3.35749 | -5.95690 | -0.60908 |
| C | 3.68192 | -4.26556 | 0.70497 |
| H | 3.81638 | -4.89192 | 1.59282 |
| C | 3.76873 | -2.86597 | 0.83314 |
| C | 3.22518 | -1.84743 | -2.89519 |
| H | 3.22223 | -0.77945 | -2.61730 |
| C | 1.90193 | -2.12482 | -3.64101 |
| H | 1.83536 | -1.49147 | -4.54247 |
| H | 1.03362 | -1.89976 | -2.99901 |
| H | 1.82976 | -3.17672 | -3.96901 |
| C | 4.44702 | -2.09206 | -3.81207 |
| H | 4.49857 | -3.14623 | -4.13661 |
| H | 5.39436 | -1.85329 | -3.29921 |
| H | 4.37984 | -1.46482 | -4.71789 |
| C | 4.08550 | -2.24443 | 2.19500 |
| H | 3.92990 | -1.15441 | 2.11559 |
| C | 3.15165 | -2.75811 | 3.31127 |
| H | 3.30508 | -3.83395 | 3.50760 |
| H | 2.09116 | -2.60867 | 3.05465 |
| H | 3.36141 | -2.22221 | 4.25323 |
| C | 5.56697 | -2.48277 | 2.57401 |
| H | 5.80051 | -2.00583 | 3.54175 |
| H | 6.25804 | -2.07397 | 1.81729 |
| H | 5.77999 | -3.56203 | 2.66910 |
| C | 2.62321 | 2.74567 | 0.06351 |
| C | 2.26578 | 3.21469 | 1.35207 |
| C | 1.65755 | 4.48259 | 1.43216 |
| H | 1.36136 | 4.87085 | 2.41193 |
| C | 1.42409 | 5.24934 | 0.28390 |
| H | 0.95624 | 6.23588 | 0.37070 |
| C | 1.78702 | 4.75703 | -0.97569 |
| H | 1.59562 | 5.36092 | -1.86891 |
| C | 2.39460 | 3.49441 | -1.11799 |

| | | | |
|---|---|---|---|
| C | 2.49813 | 2.39792 | 2.62292 |
| H | 3.07840 | 1.49966 | 2.34841 |
| C | 3.32794 | 3.18044 | 3.66552 |
| H | 4.29424 | 3.51414 | 3.25028 |
| H | 3.53313 | 2.54425 | 4.54369 |
| H | 2.79063 | 4.07482 | 4.02589 |
| C | 1.15673 | 1.91545 | 3.22049 |
| H | 0.51219 | 2.76620 | 3.50065 |
| H | 1.33712 | 1.31093 | 4.12639 |
| H | 0.59634 | 1.29846 | 2.49947 |
| C | 2.78319 | 2.98960 | -2.50719 |
| H | 3.18765 | 1.96847 | -2.39637 |
| C | 3.89228 | 3.86728 | -3.13176 |
| H | 4.18925 | 3.46896 | -4.11740 |
| H | 4.79090 | 3.90377 | -2.49242 |
| H | 3.54581 | 4.90493 | -3.28099 |
| C | 1.55261 | 2.89558 | -3.43682 |
| H | 1.10495 | 3.88833 | -3.61856 |
| H | 0.77606 | 2.24557 | -3.00076 |
| H | 1.84525 | 2.47900 | -4.41632 |
| C | -4.68264 | -1.03478 | 0.55200 |
| C | -4.89173 | -2.42838 | 0.46779 |
| H | -4.03275 | -3.10382 | 0.37632 |
| C | -6.18709 | -2.97025 | 0.51189 |
| H | -6.32774 | -4.05494 | 0.44308 |
| C | -7.29928 | -2.12418 | 0.65002 |
| H | -8.31020 | -2.54500 | 0.68742 |
| C | -7.10880 | -0.73633 | 0.74396 |
| H | -7.97174 | -0.07005 | 0.85641 |
| C | -5.81136 | -0.19893 | 0.69365 |
| H | -5.68078 | 0.88653 | 0.77629 |
| C | -1.70781 | -1.64319 | 1.44035 |
| C | -1.09190 | -2.70708 | 0.74522 |
| H | -1.10332 | -2.71265 | -0.35038 |
| C | -0.46136 | -3.75329 | 1.44137 |
| H | 0.01147 | -4.57090 | 0.88619 |
| C | -0.43578 | -3.74836 | 2.84608 |
| H | 0.04318 | -4.56964 | 3.39071 |
| C | -1.02707 | -2.68600 | 3.55061 |
| H | -1.00428 | -2.67389 | 4.64637 |
| C | -1.65658 | -1.64276 | 2.85058 |
| H | -2.11743 | -0.82223 | 3.41333 |
| C | -2.83151 | 1.44232 | 1.45733 |
| C | -3.55209 | 1.59237 | 2.66407 |
| H | -4.13551 | 0.75316 | 3.06153 |
| C | -3.55430 | 2.81545 | 3.35686 |
| H | -4.12043 | 2.91405 | 4.29011 |
| C | -2.84003 | 3.91234 | 2.84727 |
| H | -2.84804 | 4.86893 | 3.38192 |
| C | -2.12466 | 3.78007 | 1.64559 |
| H | -1.56794 | 4.63146 | 1.23915 |
| C | -2.11983 | 2.55399 | 0.95812 |
| H | -1.55947 | 2.45734 | 0.02057 |
| C | -0.80214 | -0.00909 | -1.65936 |
| C | -2.14669 | -0.06435 | -1.40823 |
| C | -3.15873 | 0.05418 | -2.49655 |
| C | -4.28926 | 0.89588 | -2.37025 |
| C | -2.99996 | -0.65320 | -3.71342 |
| C | -5.20860 | 1.04162 | -3.41891 |
| H | -4.43086 | 1.46375 | -1.44393 |
| C | -3.92166 | -0.51445 | -4.76072 |
| H | -2.14449 | -1.32930 | -3.81953 |
| C | -5.03127 | 0.33571 | -4.62019 |
| H | -6.06832 | 1.70989 | -3.29678 |
| H | -3.77886 | -1.08118 | -5.68800 |
| H | -5.75455 | 0.44144 | -5.43590 |
| H | -0.60980 | 0.14736 | -2.74688 |

**TS1$_{10}$**
SCF (BP86) Energy = -2364.70835249
Enthalpy 0K = -2363.722375
Enthalpy 298K = -2363.721431
Free Energy 298K = -2363.888799
Lowest Frequency = -55.4787 cm$^{-1}$
Second Frequency = 6.7245 cm$^{-1}$
SCF (BP86-D3BJ) Energy = -2365.02164114
SCF ($C_6H_6$) Energy = -2364.72464322
SCF (BS2) Energy = -2656.150453

| | | | |
|---|---|---|---|
| Ge | 1.69718 | -1.18586 | -0.27751 |
| Cu | -0.35550 | -0.03495 | 0.07335 |
| N | -2.75132 | 1.45266 | -0.94064 |
| N | -3.33963 | -0.24134 | 0.27476 |
| C | -2.22086 | 0.45259 | -0.14784 |
| C | -4.14407 | 1.37529 | -1.01034 |
| H | -4.73169 | 2.08057 | -1.59145 |
| C | -4.51573 | 0.30841 | -0.24037 |
| H | -5.49368 | -0.10630 | -0.01283 |
| C | -3.32086 | -1.39728 | 1.15238 |
| C | -3.42954 | -1.18834 | 2.55094 |
| C | -3.45440 | -2.32889 | 3.37709 |
| H | -3.53785 | -2.20373 | 4.46127 |
| C | -3.37283 | -3.61665 | 2.83472 |
| H | -3.38948 | -4.48967 | 3.49565 |
| C | -3.26732 | -3.79158 | 1.44970 |
| H | -3.19821 | -4.80231 | 1.03721 |
| C | -3.24128 | -2.68970 | 0.57284 |
| C | -3.56759 | 0.20582 | 3.16504 |
| H | -3.25005 | 0.94205 | 2.40381 |
| C | -2.67292 | 0.40361 | 4.40830 |
| H | -2.72006 | 1.45276 | 4.74674 |
| H | -1.61887 | 0.15654 | 4.19958 |
| H | -3.00295 | -0.22486 | 5.25344 |
| C | -5.04639 | 0.50626 | 3.50979 |
| H | -5.42692 | -0.21299 | 4.25606 |
| H | -5.69344 | 0.43976 | 2.61919 |
| H | -5.14948 | 1.52110 | 3.93179 |
| C | -3.18135 | -2.90296 | -0.93998 |
| H | -2.77917 | -1.97780 | -1.39003 |
| C | -2.24611 | -4.05730 | -1.35184 |
| H | -2.64710 | -5.03966 | -1.04526 |
| H | -1.23761 | -3.94406 | -0.92431 |
| H | -2.14373 | -4.08029 | -2.44931 |
| C | -4.60374 | -3.13182 | -1.50867 |
| H | -4.56277 | -3.26537 | -2.60358 |
| H | -5.27735 | -2.28478 | -1.29543 |
| H | -5.05580 | -4.04027 | -1.07272 |
| C | -1.97977 | 2.49132 | -1.59723 |

| | | | |
|---|---|---|---|
| C | -1.32044 | 2.18855 | -2.81731 |
| C | -0.60924 | 3.23191 | -3.44017 |
| H | -0.08065 | 3.03207 | -4.37643 |
| C | -0.56580 | 4.51694 | -2.88447 |
| H | -0.00737 | 5.31153 | -3.39062 |
| C | -1.23333 | 4.78664 | -1.68504 |
| H | -1.19525 | 5.79451 | -1.25850 |
| C | -1.95239 | 3.78093 | -1.00910 |
| C | -1.42345 | 0.81274 | -3.47752 |
| H | -1.58570 | 0.06999 | -2.67591 |
| C | -2.64600 | 0.76696 | -4.42711 |
| H | -3.58716 | 0.98992 | -3.89642 |
| H | -2.74207 | -0.23338 | -4.88381 |
| H | -2.53479 | 1.50390 | -5.24222 |
| C | -0.13972 | 0.39483 | -4.22133 |
| H | 0.04063 | 1.01202 | -5.11940 |
| H | -0.23615 | -0.64938 | -4.56399 |
| H | 0.74726 | 0.46208 | -3.57042 |
| C | -2.68530 | 4.11836 | 0.29076 |
| H | -3.06270 | 3.17532 | 0.72405 |
| C | -3.90682 | 5.02658 | 0.01375 |
| H | -4.45206 | 5.23959 | 0.94965 |
| H | -4.61270 | 4.55947 | -0.69401 |
| H | -3.59179 | 5.99176 | -0.42005 |
| C | -1.75023 | 4.76197 | 1.33801 |
| H | -1.36141 | 5.73592 | 0.99290 |
| H | -0.89258 | 4.10692 | 1.56209 |
| H | -2.30112 | 4.94441 | 2.27722 |
| C | 2.79052 | -1.54615 | 1.40029 |
| C | 2.14315 | -1.77125 | 2.63583 |
| H | 1.04718 | -1.73433 | 2.68437 |
| C | 2.87378 | -2.03265 | 3.80932 |
| H | 2.34837 | -2.20515 | 4.75622 |
| C | 4.27789 | -2.06673 | 3.76831 |
| H | 4.85164 | -2.26646 | 4.68041 |
| C | 4.94071 | -1.84329 | 2.54926 |
| H | 6.03618 | -1.87022 | 2.50879 |
| C | 4.20222 | -1.58713 | 1.38058 |
| H | 4.73520 | -1.40718 | 0.43935 |
| C | 1.55766 | -3.04621 | -1.09601 |
| C | 1.64643 | -4.21126 | -0.30137 |
| H | 1.84004 | -4.11795 | 0.77423 |
| C | 1.50676 | -5.49278 | -0.86367 |
| H | 1.58427 | -6.38065 | -0.22479 |
| C | 1.28097 | -5.63798 | -2.24238 |
| H | 1.17904 | -6.63570 | -2.68398 |
| C | 1.19461 | -4.49272 | -3.05197 |
| H | 1.02798 | -4.59555 | -4.13105 |
| C | 1.32725 | -3.21521 | -2.48102 |
| H | 1.26553 | -2.33372 | -3.13135 |
| C | 3.00477 | -0.24914 | -1.52023 |
| C | 3.93740 | -0.95425 | -2.31455 |
| H | 3.94984 | -2.05044 | -2.29120 |
| C | 4.84546 | -0.27707 | -3.14816 |
| H | 5.55942 | -0.84669 | -3.75491 |
| C | 4.83921 | 1.12617 | -3.20393 |
| H | 5.54596 | 1.65560 | -3.85305 |
| C | 3.92041 | 1.84601 | -2.42250 |
| H | 3.91008 | 2.94174 | -2.45734 |
| C | 3.01422 | 1.16248 | -1.59320 |
| H | 2.30405 | 1.73827 | -0.98687 |
| C | 0.16098 | 1.54471 | 2.01698 |
| C | 1.27083 | 2.07678 | 2.09745 |
| C | 2.58058 | 2.63583 | 2.13855 |
| C | 2.81610 | 3.95437 | 1.66731 |
| C | 3.66183 | 1.88810 | 2.67525 |
| C | 4.09953 | 4.50788 | 1.73628 |
| H | 1.98264 | 4.52848 | 1.25138 |
| C | 4.93992 | 2.45525 | 2.73643 |
| H | 3.48556 | 0.86708 | 3.02500 |
| C | 5.16448 | 3.76249 | 2.27157 |
| H | 4.27117 | 5.52553 | 1.36984 |
| H | 5.76779 | 1.86674 | 3.14448 |
| H | 6.16772 | 4.19844 | 2.32184 |
| H | -0.85296 | 1.26842 | 2.24256 |

**Int$_{10}$**

SCF (BP86) Energy = -2364.71162620
Enthalpy 0K = -2363.724440
Enthalpy 298K = -2363.723495
Free Energy 298K = -2363.884592
Lowest Frequency = 7.2788 cm$^{-1}$
Second Frequency = 14.7040 cm$^{-1}$
SCF (BP86-D3BJ) Energy = -2365.03611263
SCF ($C_6H_6$) Energy = -2364.72709140
SCF (BS2) Energy = -2656.155666

| | | | |
|---|---|---|---|
| Ge | -1.74286 | -0.37495 | 0.56134 |
| Cu | 0.21412 | 0.46111 | -0.63074 |
| N | 2.95380 | 0.81450 | 0.52107 |
| N | 2.83389 | -1.08019 | -0.52803 |
| C | 2.05098 | -0.01274 | -0.12509 |
| C | 4.24639 | 0.28001 | 0.50746 |
| H | 5.08386 | 0.79100 | 0.97351 |
| C | 4.17149 | -0.90873 | -0.15718 |
| H | 4.93133 | -1.64671 | -0.39634 |
| C | 2.36658 | -2.28547 | -1.19025 |
| C | 2.19891 | -2.28147 | -2.59860 |
| C | 1.75653 | -3.47473 | -3.20270 |
| H | 1.61212 | -3.50284 | -4.28721 |
| C | 1.51303 | -4.62546 | -2.44414 |
| H | 1.16483 | -5.54055 | -2.93479 |
| C | 1.73039 | -4.61262 | -1.06185 |
| H | 1.55608 | -5.52188 | -0.47866 |
| C | 2.16804 | -3.44846 | -0.40062 |
| C | 2.57911 | -1.08219 | -3.46681 |
| H | 2.63096 | -0.19800 | -2.80949 |
| C | 1.54172 | -0.77805 | -4.56677 |
| H | 1.83326 | 0.13259 | -5.11863 |
| H | 0.54156 | -0.61133 | -4.13640 |
| H | 1.47220 | -1.59459 | -5.30646 |
| C | 3.98397 | -1.29852 | -4.08099 |
| H | 3.99295 | -2.18325 | -4.74161 |
| H | 4.74891 | -1.45500 | -3.30153 |
| H | 4.28387 | -0.42260 | -4.68278 |
| C | 2.48000 | -3.49539 | 1.09639 |
| H | 2.53098 | -2.45614 | 1.46594 |
| C | 1.39790 | -4.22416 | 1.91650 |

| | | | |
|---|---|---|---|
| H | 1.36590 | -5.30301 | 1.68289 |
| H | 0.39753 | -3.80317 | 1.73873 |
| H | 1.62085 | -4.13401 | 2.99289 |
| C | 3.86082 | -4.15599 | 1.33533 |
| H | 4.10063 | -4.16572 | 2.41276 |
| H | 4.67590 | -3.62955 | 0.81096 |
| H | 3.85744 | -5.20150 | 0.97985 |
| C | 2.64993 | 2.09109 | 1.14322 |
| C | 2.12711 | 2.09918 | 2.46433 |
| C | 1.88531 | 3.35459 | 3.05466 |
| H | 1.46970 | 3.39782 | 4.06475 |
| C | 2.16770 | 4.54639 | 2.37580 |
| H | 1.97238 | 5.50964 | 2.85891 |
| C | 2.70263 | 4.50789 | 1.08449 |
| H | 2.92737 | 5.44445 | 0.56463 |
| C | 2.95555 | 3.28256 | 0.43616 |
| C | 1.89691 | 0.80957 | 3.25599 |
| H | 1.57820 | 0.03032 | 2.54004 |
| C | 3.21562 | 0.34139 | 3.92225 |
| H | 4.01211 | 0.15394 | 3.18379 |
| H | 3.04877 | -0.59453 | 4.48314 |
| H | 3.58195 | 1.10305 | 4.63326 |
| C | 0.79164 | 0.93539 | 4.32242 |
| H | 1.09287 | 1.60034 | 5.15166 |
| H | 0.59466 | -0.05523 | 4.76503 |
| H | -0.15004 | 1.31667 | 3.89497 |
| C | 3.58562 | 3.28165 | -0.95761 |
| H | 3.45468 | 2.27096 | -1.38361 |
| C | 5.10526 | 3.56477 | -0.87470 |
| H | 5.55921 | 3.54497 | -1.88086 |
| H | 5.62955 | 2.82160 | -0.25145 |
| H | 5.29352 | 4.56075 | -0.43693 |
| C | 2.91006 | 4.28904 | -1.91442 |
| H | 3.10880 | 5.33259 | -1.61471 |
| H | 1.81790 | 4.14766 | -1.94709 |
| H | 3.30931 | 4.16742 | -2.93664 |
| C | -3.25315 | -0.84858 | -0.72296 |
| C | -2.94018 | -1.51387 | -1.92932 |
| H | -1.88960 | -1.70870 | -2.18278 |
| C | -3.94623 | -1.93089 | -2.81893 |
| H | -3.67688 | -2.44846 | -3.74736 |
| C | -5.29481 | -1.67884 | -2.52036 |
| H | -6.08245 | -1.99743 | -3.21241 |
| C | -5.62613 | -1.01132 | -1.32989 |
| H | -6.67669 | -0.80722 | -1.09095 |
| C | -4.61483 | -0.60586 | -0.44196 |
| H | -4.89534 | -0.08583 | 0.48180 |
| C | -1.78849 | -2.05704 | 1.72241 |
| C | -2.23200 | -3.29393 | 1.20097 |
| H | -2.52110 | -3.36385 | 0.14578 |
| C | -2.34228 | -4.43451 | 2.01609 |
| H | -2.69933 | -5.37826 | 1.58702 |
| C | -2.01026 | -4.36399 | 3.37918 |
| H | -2.10432 | -5.24918 | 4.01819 |
| C | -1.56485 | -3.14522 | 3.91727 |
| H | -1.31213 | -3.07581 | 4.98218 |
| C | -1.45549 | -2.00994 | 3.09524 |
| H | -1.13508 | -1.06174 | 3.54155 |
| C | -2.51916 | 0.98850 | 1.86504 |
| C | -3.56642 | 0.66508 | 2.75964 |
| H | -3.96728 | -0.35519 | 2.77920 |
| C | -4.09556 | 1.62171 | 3.64359 |
| H | -4.91025 | 1.34564 | 4.32336 |
| C | -3.57978 | 2.92848 | 3.66052 |
| H | -3.99058 | 3.67478 | 4.34978 |
| C | -2.52954 | 3.26881 | 2.79305 |
| H | -2.11336 | 4.28294 | 2.80453 |
| C | -2.00766 | 2.30566 | 1.90968 |
| H | -1.18083 | 2.58468 | 1.24334 |
| C | 0.61201 | 1.58292 | -2.20836 |
| C | -0.65169 | 1.64791 | -2.04059 |
| C | -1.94418 | 2.12022 | -2.50627 |
| C | -3.00562 | 2.43641 | -1.63047 |
| C | -2.12149 | 2.32231 | -3.89963 |
| C | -4.20459 | 2.95906 | -2.13387 |
| H | -2.88143 | 2.27916 | -0.55640 |
| C | -3.32685 | 2.83160 | -4.39559 |
| H | -1.30163 | 2.07295 | -4.58078 |
| C | -4.37233 | 3.15611 | -3.51357 |
| H | -5.01618 | 3.20331 | -1.44102 |
| H | -3.45034 | 2.97821 | -5.47407 |
| H | -5.31496 | 3.55574 | -3.90225 |
| H | 1.51989 | 1.89223 | -2.70889 |

**$TS2_{10}$**

SCF (BP86) Energy = -2364.68229854

Enthalpy 0K = -2363.696731

Enthalpy 298K = -2363.695787

Free Energy 298K = -2363.855156

Lowest Frequency = -151.3886 $cm^{-1}$

Second Frequency = 9.5605 $cm^{-1}$

SCF (BP86-D3BJ) Energy = -2365.01392607

SCF ($C_6H_6$) Energy = -2364.69799211

SCF (BS2) Energy = -2656.125325

| | | | |
|---|---|---|---|
| Ge | 1.82440 | -0.89928 | -0.50624 |
| Cu | -0.22951 | 0.10807 | 0.80968 |
| N | -2.40245 | 1.96496 | 0.17300 |
| N | -3.20407 | -0.01922 | 0.53080 |
| C | -2.00118 | 0.64672 | 0.34881 |
| C | -3.78988 | 2.10269 | 0.25649 |
| H | -4.27938 | 3.06585 | 0.14544 |
| C | -4.29339 | 0.85384 | 0.47668 |
| H | -5.31397 | 0.50017 | 0.59134 |
| C | -3.39789 | -1.44889 | 0.68226 |
| C | -3.29826 | -2.02774 | 1.97395 |
| C | -3.57883 | -3.40342 | 2.08391 |
| H | -3.50969 | -3.88546 | 3.06349 |
| C | -3.95699 | -4.16190 | 0.96814 |
| H | -4.17819 | -5.22861 | 1.08312 |
| C | -4.05833 | -3.55882 | -0.28995 |
| H | -4.35585 | -4.15765 | -1.15692 |
| C | -3.78126 | -2.18871 | -0.46429 |
| C | -2.98507 | -1.19139 | 3.21544 |
| H | -2.37965 | -0.32845 | 2.88660 |
| C | -2.14088 | -1.94798 | 4.25864 |
| H | -1.87710 | -1.26640 | 5.08562 |
| H | -1.20422 | -2.31467 | 3.80977 |

| | | | |
|---|---|---|---|
| H | -2.68631 | -2.79880 | 4.70443 |
| C | -4.29514 | -0.66558 | 3.85203 |
| H | -4.93466 | -1.50289 | 4.18396 |
| H | -4.88048 | -0.05441 | 3.14444 |
| H | -4.07094 | -0.03975 | 4.73360 |
| C | -3.94313 | -1.55219 | -1.84570 |
| H | -3.57690 | -0.51259 | -1.78665 |
| C | -3.10191 | -2.27001 | -2.92156 |
| H | -3.43478 | -3.31169 | -3.07157 |
| H | -2.03542 | -2.29492 | -2.64972 |
| H | -3.20346 | -1.74865 | -3.88956 |
| C | -5.43437 | -1.50109 | -2.25457 |
| H | -5.54925 | -1.01080 | -3.23699 |
| H | -6.04248 | -0.94356 | -1.52174 |
| H | -5.85856 | -2.51740 | -2.33456 |
| C | -1.53114 | 3.07464 | -0.15408 |
| C | -1.48494 | 3.51035 | -1.50406 |
| C | -0.65567 | 4.61074 | -1.80151 |
| H | -0.60125 | 4.97498 | -2.83279 |
| C | 0.09516 | 5.24175 | -0.80312 |
| H | 0.74026 | 6.08945 | -1.05755 |
| C | 0.01618 | 4.79903 | 0.52394 |
| H | 0.59958 | 5.30771 | 1.29649 |
| C | -0.80675 | 3.71483 | 0.88576 |
| C | -2.33166 | 2.87299 | -2.60896 |
| H | -2.75560 | 1.93414 | -2.21246 |
| C | -3.51175 | 3.80040 | -2.98837 |
| H | -4.14951 | 4.03429 | -2.11923 |
| H | -4.14483 | 3.32545 | -3.75785 |
| H | -3.14365 | 4.75783 | -3.39723 |
| C | -1.51145 | 2.50836 | -3.86447 |
| H | -1.10633 | 3.40598 | -4.36403 |
| H | -2.15617 | 1.99220 | -4.59660 |
| H | -0.66636 | 1.84579 | -3.62116 |
| C | -0.97364 | 3.30942 | 2.35203 |
| H | -1.18732 | 2.22527 | 2.37638 |
| C | -2.18524 | 4.04989 | 2.97174 |
| H | -2.32565 | 3.74314 | 4.02281 |
| H | -3.12100 | 3.83539 | 2.42919 |
| H | -2.02499 | 5.14262 | 2.95434 |
| C | 0.28870 | 3.54120 | 3.20469 |
| H | 0.50420 | 4.61611 | 3.34145 |
| H | 1.17564 | 3.06132 | 2.76108 |
| H | 0.13839 | 3.11385 | 4.21045 |
| C | 3.53424 | -1.83794 | 0.04569 |
| C | 3.50236 | -3.16492 | 0.52574 |
| H | 2.54860 | -3.70113 | 0.58688 |
| C | 4.68163 | -3.82427 | 0.91220 |
| H | 4.63274 | -4.85592 | 1.27945 |
| C | 5.91941 | -3.16814 | 0.81904 |
| H | 6.84038 | -3.68226 | 1.11599 |
| C | 5.96869 | -1.84904 | 0.34048 |
| H | 6.92960 | -1.32752 | 0.26395 |
| C | 4.78667 | -1.19050 | -0.03825 |
| H | 4.84641 | -0.15860 | -0.40066 |
| C | 0.74185 | -2.47129 | -1.17657 |
| C | 0.04879 | -3.31393 | -0.27761 |
| H | -0.02473 | -3.04003 | 0.78115 |
| C | -0.54533 | -4.50768 | -0.72571 |
| H | -1.07659 | -5.14839 | -0.01395 |
| C | -0.45906 | -4.87555 | -2.07788 |
| H | -0.91078 | -5.81167 | -2.42447 |
| C | 0.20766 | -4.03552 | -2.98571 |
| H | 0.27440 | -4.31360 | -4.04392 |
| C | 0.79776 | -2.84058 | -2.53880 |
| H | 1.32399 | -2.20046 | -3.25665 |
| C | 2.38615 | 0.22603 | -2.08856 |
| C | 3.38122 | -0.23763 | -2.97919 |
| H | 3.85780 | -1.20960 | -2.80804 |
| C | 3.78828 | 0.54108 | -4.07695 |
| H | 4.55938 | 0.16242 | -4.75761 |
| C | 3.21720 | 1.80543 | -4.29434 |
| H | 3.54242 | 2.41806 | -5.14249 |
| C | 2.23209 | 2.28225 | -3.41461 |
| H | 1.78485 | 3.27026 | -3.57041 |
| C | 1.81788 | 1.49430 | -2.32620 |
| H | 1.04455 | 1.87681 | -1.64721 |
| C | 0.56063 | -0.40595 | 2.56177 |
| C | 1.54662 | 0.00124 | 1.77048 |
| C | 2.82869 | 0.70263 | 1.96303 |
| C | 3.22641 | 1.78783 | 1.14855 |
| C | 3.66174 | 0.32817 | 3.04498 |
| C | 4.41440 | 2.48496 | 1.41615 |
| H | 2.59346 | 2.08308 | 0.30571 |
| C | 4.85088 | 1.02167 | 3.30366 |
| H | 3.36758 | -0.52232 | 3.66808 |
| C | 5.23311 | 2.10407 | 2.49207 |
| H | 4.70152 | 3.32818 | 0.77835 |
| H | 5.48834 | 0.70990 | 4.13832 |
| H | 6.16470 | 2.64311 | 2.69451 |
| H | 0.54036 | -0.20786 | 3.64413 |

## References


1. J. W. Hall, D. M. L. Unson, P. Brunel, L. R. Collins, M. K. Cybulski, M. F. Mahon and M. K. Whittlesey, Organometallics, 2018, **37**, 3102–3110; A. J. Jordan, C. M. Wyss, J. Bacsa, and J. P. Sadighi, *Organometallics*, 2016, **35**, 613–616; A. Bonet, V. Lillo, J. Ramírez, M. M. Díaz-Requejo, and E. Fernandez, *Org. Biomol. Chem.*, 2009, **7**, 1533–1535; L. R. Collins, I. M. Riddlestone, M. F. Mahon and M. K. Whittlesey, *Chem. Eur. J.*, 2015, **21**, 14075–14084; L. A. Goj, E. D. Blue, S. A. Delp, T. B. Gunnoe, T. R. Cundari, A. W. Pierpont, J. L. Petersen, and P. D. Boyle, *Inorg. Chem.*, 2006, **45**, 9032–9045; E. Colomer, R. J. P. Corriu, C. Marzin, and A. Vioux, *Inorg. Chem.*, 1982, **21**, 368–373; M. Suzuki, H. Son, Ryoji Noyori, and H. Masuda, *Organometallics*, 1990, 9, 12, 3043–3053; J. E. Griffiths and M. Onyszchuk, Can. J. Chem., 1961, **39**, 339–347; A. Harinath, J. Bhattacharjee, S. Anga and T. K. Panda, Aust. J. Chem., 2017, 70, 724.
2. G. Sheldrick, *Acta Cryst. A*, 2015, **71**, 3–8O.
3. O. V. Dolomanov, L. J. Bourhis, R. J. Gildea, J. A. K. Howard and H. Puschmann, *J. Appl. Cryst.*, 2009, **42**, 339–341.
4. G. R. Fulmer, A. J. M. Miller, N. H. Sherden, H. E. Gottlieb, A. Nudelman, B. M. Stoltz, J. E. Bercaw, and K. I. Goldberg, *Organometallics*, 2010, **29**, 2176–2179.
5. H. Koglin, K. Behrends, and M. Draeger, *Organometallics*, 1994, **13**, 2733–2742.
6. O. Santoro, A. Collado, A. M. Z. Slawin, S. P. Nolan and C. S. J. Cazin, *Chem. Commun.*, 2013, **49**, 10483–10485.
7. K. V. Zaitsev, A. A. Kapranov, Y. F. Oprunenko, A. V. Churakov, J. A.K. Howard, B. N. Tarasevich, S. S. Karlov, G. S. Zaitseva, *J. Organomet. Chem.*, 2012, **700**, 207–213.
8. I. Elser, R. J. Andrews, and D. W. Stephan, *Chem. Commun.*, 2022, **58**, 1740–1743.
9. S. Pelzer, B. Neumann, H. Stammler, N. Ignat'ev, and B. Hoge, *Chem. Eur. J.*, 2016, **22**, 4758–4763.
10. S. R. Foley, G. P. A. Yap, and D. S. Richeson, *Organometallics*, 1999, **18**, 4700–4705.
11. L. A. Goj, E. D. Blue, C. Munro-Leighton, T. B. Gunnoe, and J. L. Petersen, *Inorg. Chem.*, 2005, **44**, 8647–8649.
12. K. V. Zaitsev, A. V. Kharcheva, K. Lam, Z. Zhanabil, G. Issabayeva, Y. F. Oprunenko, A. V. Churakov, G. S. Zaitseva, S. S. Karlov, *J. Organomet. Chem.*, 2018, **867**, 228–237.
13. A. G. Medvedev, M. Yu. Sharipov, D. A. Grishanov, A. V. Eshtukov, A. V. Churakov, I. A. Buldashov, P. A. Egorov, O. Levb and P. V. Prikhodchenko, *Mendeleev Commun.*, 2022, **32**, 57–59.
14. F. Wech, M. Hasenbeck, U. Gellrich, Chem. Eur. J., 2020, **26**, 13445–13450.
15. A. J. Jordan, C. M. Wyss, J. Bacsa, and J. P. Sadighi, *Organometallics*, 2016, **35**, 613–616.
16. Gaussian 16, Revision C.01, M. J. Frisch, G. W. Trucks, H. B. Schlegel, G. E. Scuseria, M. a. Robb, J. R. Cheeseman, G. Scalmani, V. Barone, G. a. Petersson, H. Nakatsuji, X. Li, M. Caricato, a. V. Marenich, J. Bloino, B. G. Janesko, R. Gomperts, B. Mennucci, H. P. Hratchian, J. V. Ortiz, a. F. Izmaylov, J. L. Sonnenberg, Williams, F. Ding, F. Lipparini, F. Egidi, J. Goings, B. Peng, A. Petrone, T. Henderson, D. Ranasinghe, V. G. Zakrzewski, J. Gao, N. Rega, G. Zheng, W. Liang, M. Hada, M. Ehara, K. Toyota, R. Fukuda, J. Hasegawa, M. Ishida, T. Nakajima, Y. Honda, O. Kitao, H. Nakai, T. Vreven, K. Throssell, J. a. Montgomery Jr., J. E. Peralta, F. Ogliaro, M. J. Bearpark, J. J. Heyd, E. N. Brothers, K. N. Kudin, V. N. Staroverov, T. a. Keith, R. Kobayashi, J. Normand, K. Raghavachari, a. P. Rendell, J. C. Burant, S. S. Iyengar, J. Tomasi, M. Cossi, J. M. Millam, M. Klene, C. Adamo, R. Cammi, J. W. Ochterski, R. L. Martin, K. Morokuma, O. Farkas, J. B. Foresman, D. J. Fox, Gaussian, Inc., Wallingford CT, 2016.
17. D. Andrae, U. Häußermann, M. Dolg, H. Stoll, H. Preuß, *Theor. Chim. Acta,* 1990, **77**, 123−141.

18. (a) P. C. Hariharan, J. A. Pople, *Theor. Chim. Acta,* 1973, **28**, 213−222. (b) W. J. Hehre, R. Ditchfield, J. A. Pople, *J. Chem. Phys.,* 1972, **56**, 2257.
19. (a) A. D. Becke, *Phys. Rev. A: At., Mol., Opt. Phys.,* 1988, **38**, 3098. (b) J. P. Perdew, *Phys. Rev. B: Condens. Matter Mater. Phys.,* 1986, **33**, 8822−8824.
20. M. Bühl, H. Kabrede, *J. Chem. Theory Comput.,* 2006, **2**, 1282–1290.
21. D. Figgen, G. Rauhut, M. Dolg, H. Stoll, *J. Chem. Phys.,* 2005, **311**, 227-244.
22. K. A. Peterson, C. Puzzarini, *Theor. Chem. Acc.,* 2005, **114**, 283-296.
23. J. Tomasi, B. Mennucci, R. Cammi, *Chem. Rev.,* 2005, **105**, 2999−3094.
24. S. Grimme, S. Ehrlich, L. Goerigk, *J. Comp. Chem.,* 2011 **32**, 1456-1465.
25. M. P. Mitoraj, A. Michalak, T. Ziegler, *J. Chem. Theory Comput.,* 2009, **5**, 962–975.
26. G. te Velde, F. M. Bickelhaupt, E. J. Baerends, C. Fonseca Guerra, S. J. A. van Gisbergen, J. G. Snijders, T. Ziegler, *J. Comput. Chem.,* 2001, **22**, 931–967.
